# Sterile Insects to Enhance Agricultural Development: The Case of Sustainable Tsetse Eradication on Unguja Island, Zanzibar, Using an Area-Wide Integrated Pest Management Approach

**DOI:** 10.1371/journal.pntd.0002857

**Published:** 2014-05-29

**Authors:** Marc J. B. Vreysen, Khalfan Saleh, Furaha Mramba, Andrew Parker, Udo Feldmann, Victor A. Dyck, Atway Msangi, Jérémy Bouyer

**Affiliations:** 1 Insect Pest Control Laboratory, Joint FAO/IAEA Programme of Nuclear Techniques in Food and Agriculture, Vienna, Austria; 2 Ministry of Agriculture, Natural Resources and Environment, Zanzibar, Tanzania; 3 Vector and Vector Borne Diseases Research Institute, Tanga, Tanzania; 4 Insect Pest Control Section, Joint FAO/IAEA Programme of Nuclear Techniques in Food and Agriculture, Vienna, Austria; 5 Retired, Independent Researcher, Vienna, Austria; 6 Livestock Research, Training & Extension, Ministry of Livestock & Fisheries Development, Dar es Salaam, Tanzania; 7 Unité Mixte de Recherche Contrôle des Maladies Animales Exotiques et Emergentes, Centre de Coopération Internationale en Recherche Agronomique pour le Développement (CIRAD), Montpellier, France; 8 Unité Mixte de Recherche 1309 Contrôle des Maladies Animales Exotiques et Emergentes, Institut national de la recherche agronomique (INRA), Montpellier, France; 9 Institut Sénégalais de Recherches Agricoles, Laboratoire National d'Elevage et de Recherches Vétérinaires, Dakar-Hann, Sénégal; International Centre of Insect Physiology and Ecology, Kenya

## Tsetse and Trypanosomosis on Unguja and Earlier Control Efforts

In 1908, African animal trypanosomosis (AAT) was first reported on the island of Unguja (Zanzibar), but circumstantial evidence of its presence is reported as far back as 1880 [1]. There are no reports of the presence of human African trypanosomosis (HAT). Tsetse are vectors of trypanosomes, and *Glossina austeni* Newstead was only discovered in 1945 on Unguja Island, which is a testimony to its elusive behaviour. Subsequent surveys demonstrated that *G. austeni* was the only species present, widespread over the island but more abundant in the thickets of the eastern part of the island [1]. The annual losses due to the disease, in terms of meat and milk production, mortality of calves, cost of disease control, and the importation of animals to satisfy the demand for protein, were estimated at 2 million USD [2]. Screening of cattle showed an overall trypanosomosis infection rate of 17%, being predominantly *Trypanosoma congolense* and to a lesser extent *T. vivax.* Local infection rates at the herd level could be as high as 46% (Supporting Information S1). Therefore, the Government of Tanzania requested assistance from the International Atomic Energy Agency (IAEA) in 1983 to assess whether the sterile insect technique (SIT) could be integrated with other proposed control tactics for the eradication of *G. austeni* from Unguja Island. A colony was initiated at the Tsetse and Trypanosomiasis Research Institute (TTRI) (now the Vector and Vector-Borne Diseases Research Institute), located in Tanga on the mainland of Tanzania, from pupae collected in the Jozani forest (now the Jozani-Chwaka Bay National Park). Flies were originally maintained on goats and rabbits, but the in vitro feeding technique was introduced in 1984 [3]. By mid-1990, the colony had reached a size of 40,000 females, and pilot release studies were implemented that confirmed the feasibility of transporting and deploying sterile males on the island.

The efficacy of using residual synthetic pyrethroids as pour-on treatments on livestock was assessed in 1987 during a pilot trial in Mangapwani (Supporting Information S1). Application of the insecticide on 700 head of cattle, 200 goats, and a few donkeys in five cycles separated by 15 days resulted in a decrease of the trap catches from one fly/sticky trap/day before the trial to zero within 37 days. None of the sentinel animals became reinfected.

## The Eradication Strategy

The pour-on trial in Mangapwani prompted the Government of Zanzibar to attempt an island-wide eradication effort (1988–1993) using this technique in areas with abundant livestock, complemented by the use of blue, insecticide-impregnated cloth screens (IIS) in those areas where livestock was absent [4]. As a result, the disease prevalence in the northern half of the island decreased to very low levels (<5%), but little impact was noticed in the cattle residing around the forests in the southern part of the island as domestic animals could not penetrate these areas [4]. The use of IIS proved to be very challenging in the dense Jozani forest ecosystem as they had to be deployed at densities of 40–70 per km^2^ for a period of 18 months to suppress the female and male fly populations by 80% and 98%, respectively [5].

In 1994, the strategy was modified, and an area-wide integrated pest management approach with an SIT component was adopted [6]. From mid-1994 to mid-1996, sterile male flies were dispersed by air over the entire southern part of the island, while, in the northern half of the island, the fly population continued to be suppressed using pour-on applications of deltamethrin on livestock and cattle dipping in strategic areas. The release area was expanded to include the middle and northern parts of the island in mid-1996, and 12 small offshore islands were included from January 1997 onwards [7].

## The Implementation

**Figure 1:**
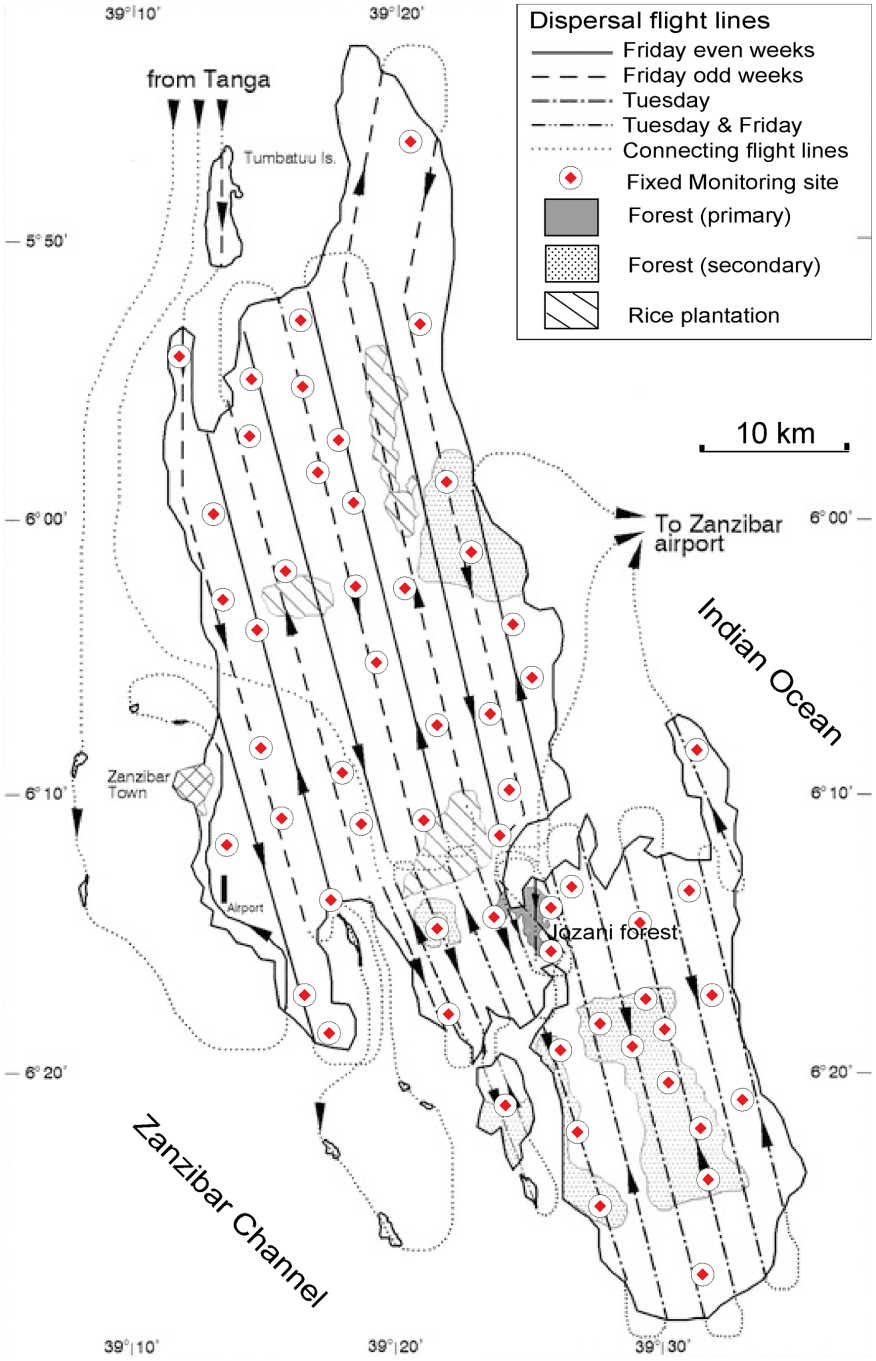
Flight lines and monitoring sites during the eradication campaign on the island of Unguja. The location of the 55 fixed monitoring sites is presented (red points).

The sterile flies for the operational release phase of the project were produced at the TTRI in Tanga, Tanzania [8]. Whereas in 1994 the *G. austeni* colony remained below 50,000 producing females, improvements in rearing methods and use of better quality blood for feeding the flies allowed colony expansion to 400,000 and 900,000 by the end of 1995 and 1996, respectively [9]. The sterile flies were released twice a week by light aircraft at an altitude of 210–270 m over fixed release paths that were separated by 1–2 km (Figure 1). The pilots used a Global Positioning System (GPS) for accurate navigation and sterile fly dispersal. The sterile flies were marked with Day Glo fluorescent powder (Radiant Color, Belgium) that enabled discrimination from the wild fly population in the trap catches.

The entire operational eradication phase was monitored with more than 500 sticky panel traps [5], [7] that were checked from once a day to once a week depending on the importance of the area (Figure 1). In addition, sentinel animals were being screened every 2–5 months for AAT transmission in 38 arbitrarily assigned blocks [10].

## The Results

**Figure 2:**
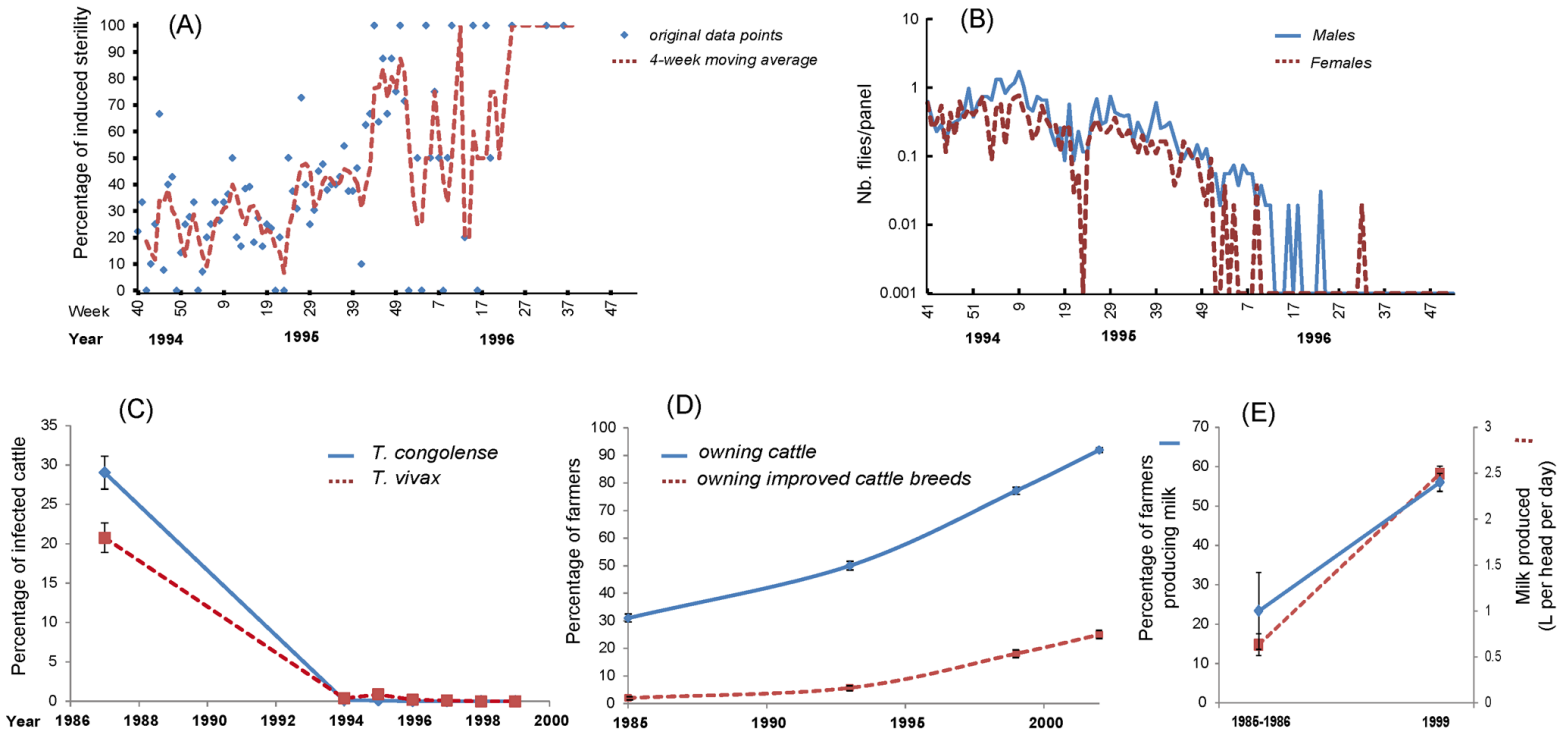
Entomological, epidemiological, and socioeconomic indicators of the eradication campaign on the island of Unguja. Graph (A) presents the rate of induced sterility (4-week moving average). Graph (B) shows the density of tsetse flies sampled with the sticky panels in fixed monitoring sites. The trap catches (+0.001) of indigenous female (dotted red line) and male (solid blue line) *G. austeni* are presented on a logarithmic scale. Graph (C) presents the decrease in trypanosome prevalence as a result of tsetse eradication. Graph (D) presents the percentage of farmers owning cattle and improved cattle breeds, and graph (E) presents the increase in milk production per cattle head (in liters) and in the percentage of farms producing milk.

During the operational sterile male release phase (August 1994–December 1997), a total of 8.5 million sterile male *G. austeni* were released over the island. Different release densities were applied over different ecosystems in relation to the suitability of that habitat to harbour *G. austeni*, i.e., from <50 sterile males/km^2^ over unsuitable habitats to >300 sterile males/km^2^ over the primary forest. During the first months of the releases, the average sterile-to-wild-male ratios remained below 10∶1. As of week 13 of 1995, more than 25,000 sterile males were released per week and as a result, the average sterile-to-wild-male ratios increased gradually and reached >100∶1 by the end of 1995. The mating frequencies of the released males as evidenced by the rate of induced sterility in the trapped wild female flies were used as a direct indicator of the efficiency of the sterile male release programme. Whereas the natural abortion rate before the release of the sterile males averaged 3.5%, the mating frequencies of wild female flies with sterile males (i.e., the rate of abortion) increased from 19% in the first period of the releases to 32%, 48%, and 72% during the 2nd, 3rd, and 4th quarters of 1995, respectively. The adequate quality of the sterile males was epitomised by an analysis of the trap catches in the Jozani forest reserve that showed that sterile males, despite being released by air in a uniform way, aggregated in the same areas as the wild male flies, ensuring adequate sterile-to-male overflooding ratios [11]. Another indicator of the success of the programme was the shift in age distribution of the female fly population, i.e., as the programme advanced, the proportion of young females in the population was gradually reduced due to the reduction in population replacement rate with each generation. As a result of the gradual increase in the rate of induced sterility in the female fly population, the fly population density as evidenced by trap catches decreased very rapidly as of mid-1995 (Figure 2). The last indigenous fly was trapped in week 36 of 1996 [7].

The transmission rate of trypanosomes in sentinel cattle was used as an indirect indicator of the progress of the programme (Figure 2). The incidence of AAT in northern Unguja was already very low in 1994 (<1%) due to the suppression of the fly population using the pour-on technique in previous years. Whereas *T. congolense* was still detected in 1994, it was not found any more in the period of 1995–1997. In southern Unguja, the incidence of AAT was higher, but *T. congolense* was not detected any more in 1996 and the incidence of *T. vivax* was 0.19% (i.e., one animal was positive out of the 520 sampled) [12]. After the releases were stopped, more than 3,000 cattle were screened for AAT in 1998–1999 [13]. None of the animals were found positive for AAT, and this has remained so ever since. AAT is occasionally reported in imported cattle from the mainland, but transmission to indigenous animals never occurs as the vector is absent.

## The Socioeconomic Impact

Two economic surveys implemented two and five years after the completion of the eradication operations (Supporting Information S2 and S3) [14] concluded that the proportion of small farmers (1) holding indigenous cattle increased from 31% to 94% between 1985 and 2002, (2) selling milk from indigenous cattle increased from 11% to 62% between 1985 and 1999, and (3) using oxen for ploughing increased to 5% in 2002 (but was expected to increase thereafter) (Figure 2). There was high demand for improved livestock breeds, and the percentage of farmers holding improved cattle breeds increased from 2% to 24% in the period of 1985–2002. In addition, from 1985 to 1999, milk production nearly tripled (Figure 2). In general, the average monthly income of farming households increased by 30% in the period of 1999–2002, and the proportion of households with a monthly income of over 25 USD and over 50 USD increased from 69% to 86% and from 22% to 36%, respectively. This could be associated with tsetse and trypanosomosis eradication since a strong correlation was observed between household income and milk yields, milk sales, and the use of manure and animal power for cultivation and transport. There was a clear acceleration of the innovation trajectories of farmers, as can be seen by increased slopes in panel D of Figure 2.

The eradication of the tsetse fly from Unguja in 1997 followed by the disappearance of AAT thereafter [12], [13] enabled farmers to integrate livestock keeping with cropping in areas where this had been impossible before. The increased livestock and crop productivity and the use of animals for transport and traction significantly contributed to an increase in the quality of people's lives.

## Lessons and Perspectives

The successful and sustained eradication of the *G. austeni* population from Unguja Island, the subsequent disappearance of the AAT disease, and the positive impact of the programme on raising the socioeconomic standards of the human population on the island incited a renewed interest from African governments. This culminated in the establishment of the Pan-African Tsetse and Trypanosomosis Eradication Campaign (PATTEC) at the African Summit in 2000 under the auspices of the African Union Commission. This political initiative provided encouragement to tsetse-affected African states for increased and renewed efforts in their struggle against tsetse and trypanosomosis and to develop and implement similar campaigns in their infested territories [14].

The success of the eradication programme on Unguja Island was possible due to the many technical and managerial prerequisites that were in place during the planning and implementation of the programme [15]. Beyond the complete isolation of the target population, the programme on Unguja Island benefitted from an extensive planning phase based on a strong set of baseline data that allowed a good strategic choice of control tactics that are very effective at high population densities and very effective at low population densities, such as the SIT; sterile flies of adequate quality available in adequate numbers and released throughout the campaign without interruption; routine quality control procedures in place; an extensive monitoring component with permanent feedback between field teams and managers, allowing the making of adequate strategic choices based on sound scientific principles rather than on process-oriented bureaucracies or political wishes; adequate national and international expertise; sufficient financial resources and adequate logistics; and regular independent programme reviews.

Failing to meet these prerequisites will increase the probability of failure for area-wide integrated pest management (AW-IPM) programmes, and in those cases, it is wise to encourage local IPM on a field by field basis that combines vector control and trypanocidal treatments aiming at suppression rather than eradication.

## Supporting Information

Supporting Information S1 Pilot trial for the control of *G. austeni* on the island of Zanzibar. Food and Agriculture Organization of the United Nations expert report by A. H. Schönefeld, in the framework of project TCP/URT/6758, “Assistance in Trypanosomiasis Control in Zanzibar, United Republic of Tanzania,” Rome, July 1988.
(PDF)

Supporting Information S2 Livestock and agriculture development in Zanzibar, post-tsetse eradication: A follow-up socioeconomic study. International Atomic Energy Agency confidential report by N. S. Y. Mdoe, Vienna, June 2003.
(PDF)

Supporting Information S3 Livestock and agriculture development in Zanzibar, pre- and post- tsetse eradication. International Atomic Energy Agency confidential report by N. E. Tambi, W. O. Maina, and S. Y. N. Mdoe, in the framework of project RAF5040, Vienna, December 1999.
(PDF)
