# Supplementary material for: Sterile Insects to Enhance Agricultural Development: The Case of Sustainable Tsetse Eradication on Unguja Island, Zanzibar, Using an Area-Wide Integrated Pest Management Approach
Source: PLoS Negl Trop Dis. 2014 May 29;8(5):e2857. doi: 10.1371/journal.pntd.0002857 (PMC4038469; doi:10.1371/journal.pntd.0002857)
Supplement: Supporting Information S1 — Pilot trial for the control of G. austeni on the island of Zanzibar. Food and Agriculture Organization of the United Nations expert report by A. H. Schönefeld, in the framework of project TCP/URT/6758, “Assistance in Trypanosomiasis Control in Zanzibar, United Republic of Tanzania,” Rome, July 1988. (PDF) [file pntd.0002857.s001.pdf]

110

AG:TCP/URT/6758

TECHNICAL COOPERATION PROGRAMME

Pilot Trial for the Control of *Glossina austeni* on the Island of Zanzibar

Report prepared by the Consultant
A.H. Schönefeld, Dr. med. vet.

Project
TCP/URT/6758
Assistance in Trypanosomiasis Control
Zanzibar, United Republic of Tanzania

FOOD AND AGRICULTURE ORGANIZATION OF THE UNITED NATIONS

Rome, July 1988

TABLE OF CONTENTS

		Page
LIST OF ABBREVIATIONS		iii
ABSTRACT		iv
1.	INTRODUCTION	1
1.1	Background information	1
1.2	Specific objectives of the technical assistance and terms of reference	3
1.3	Project staff	4
1.4	Equipment and facilities	4
2.	WORK PROGRAMME	4
2.1	Summary of project activities	4
2.2	Choice and description of the experimental area	5
2.3	Target design	7
2.4	Tsetse survey	7
2.5	Mobilization of farmers and trypanosomiasis survey	7
2.6	Organisation of tsetse control	9
2.7	Monitoring of the effects of tsetse control	10
2.8	Monitoring of the tick infestation	10
3.	RESULTS AND CONCLUSIONS	10
3.1	Distribution of Glossina austeni in the trial area	10
3.2	Reduction of tsetse during the control operation	13
3.3	Participation of farmers	15
3.4	Trypanosomiasis survey, treatment and control	17
3.5	Tick control	19
3.6	General conclusions	20
4.	RECOMMENDATIONS	20
5.	ACKNOWLEDGEMENTS	22
6.	REFERENCES	23
APPENDIX: List of supplied equipment and material		23

LIST OF ABBREVIATIONS

AHPC	Animal Health and Production Centre
HCT	Haematocrit Centrifuge Technique
IAEA	International Atomic Energy Agency
LDD	Livestock Development Department
PCV	Packed Cell Volume
SIT	Sterile Insect Technique
TCP	Technical Cooperation Programme of FAO
TTRI	Tsetse and Trypanosomiasis Research Institute, Tanga, Tanzania

ABSTRACT

Recent surveys have shown that approximately one quarter of the cattle population on Unguja Island, Zanzibar is kept in areas with endemic trypanosomiasis. One third of the island is estimated to offer suitable habitat for Glossina austeni, the sole existing cyclical vector, but its detection, particularly when in low density, is difficult. Previously bait-oxen were the only effective means of attraction, while traps and targets were largely inefficient. At the request of the Government of Zanzibar, the Food and Agriculture Organization of the United Nations (FAO) provided six months' technical assistance in 1987/88, under its Technical Cooperation Programme to test the feasibility and identify a method for the control and possible eradication of tsetse and trypanosomiasis from a defined trial area. As precursor to its eventual island-wide application, the technique selected involved the treatment of livestock, mainly cattle, with sequential applications of the residual pour-on acaricide deltamethrin.

The report describes the implementation and the results of the pilot study. Mangapwani in northwestern Zanzibar was chosen as a suitable, relatively isolated area. A modified type of glue-impregnated target was developed for surveying tsetse in the experimental zone. The extent of infested area, some 20 sq km, was larger than originally thought. Glossina austeni was caught in 26 different locations at a mean rate of one tsetse fly per target per day, the apparent fly density ranging between 0.14 and 3.66. Screening for trypanosomiasis in the area disclosed that 46 percent of the cattle were infected with Trypanosoma congolense and/or T. vivax. Thirty-eight percent of 150 cattle were either reinfected or newly infected within less than two months after curative treatment with diminazene aceturate. The negative correlation between trypanosomiasis and the mean haematocrit-value was also demonstrable.

During the control operation, insecticide was applied to nearly 700 resident cattle, 200 goats and a few donkeys in five consecutive cycles at 15 to 18 day intervals. The effects were monitored entomologically by means of sticky targets and parasitologically by repeated examination of sentinel cattle. The apparent tsetse fly density dropped to zero within 37 days after the start of the insecticide treatments. No tsetse was caught inside the trial zone of approximately 33 sq km during the following observation period of two months. None of 144 sentinel cattle was found reinfected within a similar period and their mean haematocrit-value rose from 24.2 to 29.1 percent. A good acaricidal effect of the pour-on formulation against Rhipicephalus, Amblyomma and Boophilus spp. was also observed. The promising results of the control trial against G. austeni are discussed and recommendations for larger island-wide application on Zanzibar through the recently initiated UNDP/FAO project "Animal Disease Control" are made.

1. INTRODUCTION

1.1 Background information

A key factor in the development of the livestock sector in Zanzibar is improved disease control. Bovine trypanosomiasis has long been identified as a major constraint on the cattle and dairy industry over much of Unguja Island, in particular at the important smallholder level. The introduction of more productive exotic cattle breeds or the udpgrading of the indigenous East African Zebu is inhibited due to the increased susceptibility of such animals to clinical trypanosomiasis and tick-borne disease, such as East Coast fever. Although Pemba Island is free of tsetse, Unguja harbours one species, Glossina austeni, the cyclical vector of Trypanosoma congolense and Trypanosoma vivax, both pathogenic to cattle. Human sleeping sickness does not exist on Zanzibar. The prospects for tsetse control, with sustainable results are attractive since Unguja is separated from the mainland and protected from reinvasion by some 30 km of ocean.

The detection of G. austeni has always proved difficult. Whilst animal trypanosomiasis has been recorded since the beginning of the century, the first tsetse was only caught in 1945. Surveys were conducted using either bait-oxen fly-rounds or pupal searches. All past attempts to capture adult G. austeni for the purpose of accurately determining tsetse distribution as a necessary prerequisite to undertaking a successful control campaign failed. Bait-oxen were the only partially effective means of attraction, while the effectiveness of traps and targets was generally very low. During 1985-87, the UNDP/FAO Livestock Development Project, URT/81/017, in conjunction with the tsetse and trypanosomiasis control unit of the Livestock Development Department (LDD) under the Ministry of Agriculture and Livestock, comprised a trypanosomiasis detection and diagnosis component which, in the absence of other means, broadly delimited the present-day distribution of tsetse through a trypanosomiasis prevalence survey (URT/81/017: Field Document No. 7, 1986 and No. 10, 1987; Terminal Report 1987). Figure 1 shows areas of Unguja where trypanosomiasis has been detected. About 24 percent (6430 head) of the total cattle population and 20 percent (4850) of the goats were estimated to live in areas with endemic trypanosomiasis. Over one third of the island was believed to offer a seasonal or permanent tsetse-habitat (Bouvry Stratford, 1986, Hafidh et al., 1987).

Concurrently, the Joint FAO/IAEA Division provided technical assistance, through the project "Tsetse Fly Eradication by the Sterile Insect Technique" to the Tsetse and Trypanosomiasis Research Institute (TTRI) in Tanga, which has undertaken some tsetse survey and investigative work in the Jozani forest in southern Zanzibar. Turner (1984) attempted to assess the island-wide location and density of G. austeni based on puparial searches. Hall (1986) studied cattle and goats as bait animals for the capture of flies as well as bioconical and F3 traps and, with more success, glue-impregnated sticky targets.

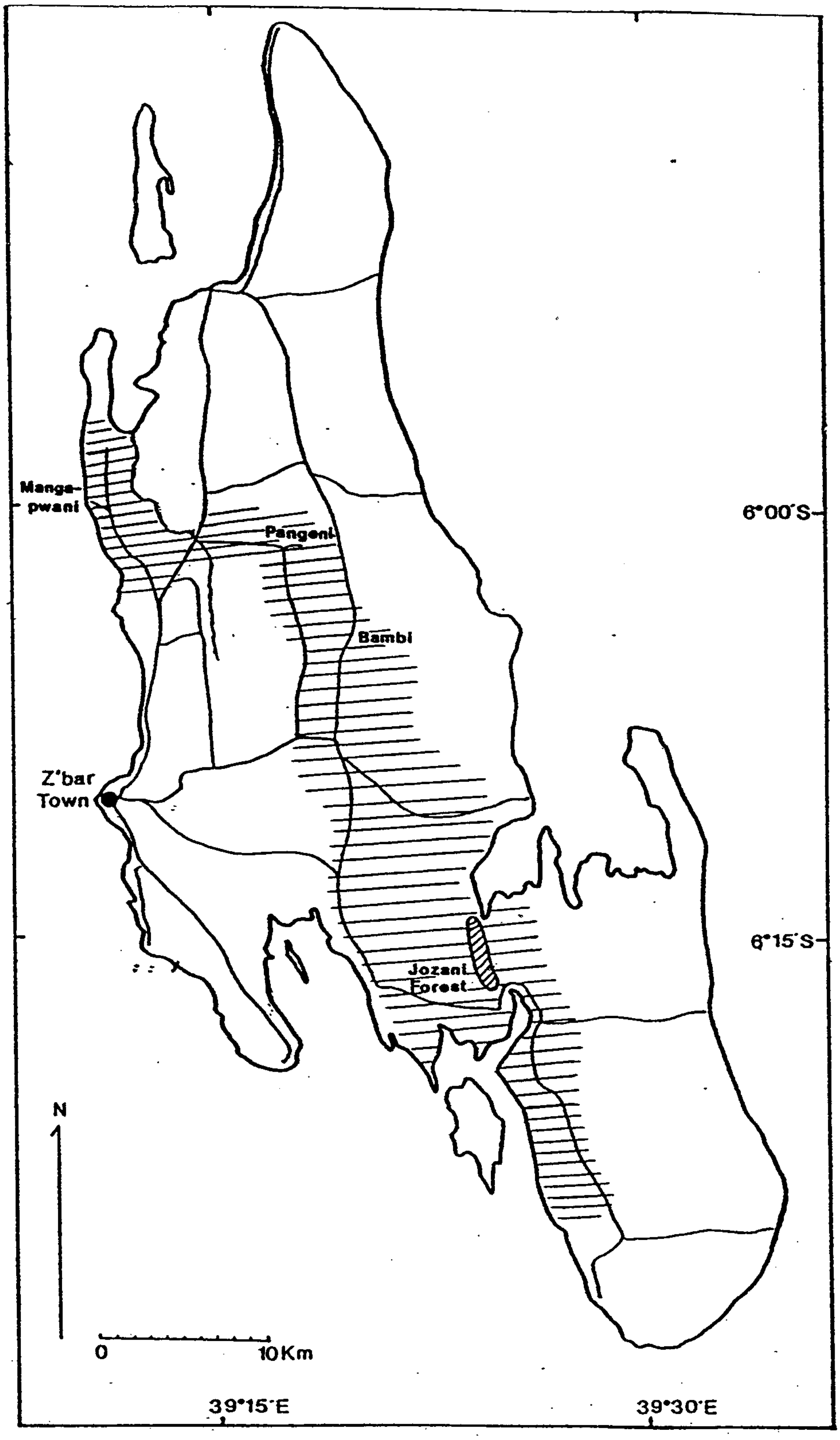

FIGURE 1: Occurrence of bovine trypanosomiasis on Unguja Island, Zanzibar (hatched area), based on findings of the 1985-87 survey (Bouvry Stratford,1986; Hafidh et al.,1987)

In support of the national development strategy to increase crop and livestock production to self-sufficiency levels, one immediate objective of the current UNDP/FAO Animal Disease Control Project, URT/86/022, is to develop and apply a method for tsetse and trypanosomiasis control which, if not entirely successful in achieving eradication, could be integrated with the Sterile Insect Technique (SIT) to produce the desired result. The proposed control technique involves the repeated block treatments of livestock (mainly cattle) in tsetse-infested areas with a pour-on formulation of a residual synthetic pyrethroid insecticide, following the successful trial of the technique undertaken in Zimbabwe (Hursey et al., in press). The formulation spreads evenly over the body surface and persists on hair-coat and skin without acting systemically. This environmentally safe approach is a modification of the modern target technique, but using cattle as moving attractants instead of stationary screens, the insecticide also acts as an effective acaricide.

The Government of Zanzibar requested FAO technical assistance to test the feasibility of this technique under local conditions on the island. Assistance was provided under the FAO Technical Cooperation Programme (TCP) project TCP/URT/6758. The project had a duration of six months and began with the arrival of the consultant in Zanzibar on 5 December 1987.

1.2 Specific objectives of the technical assistance and terms of reference

The specific objectives of the technical assistance were to broaden the existing data base on tsetse ecology, distribution and trypanosomiasis prevalence, and to field test the proposed vector control technique in a defined area for eventual application island-wide, if necessary to be integrated with the SIT in order to achieve eradication.

The terms of reference of the tsetse and trypanosomiasis control specialist were to assist the Livestock Development Department in:

- locating a suitable area for conducting a pilot tsetse control trial using insecticide-treated cattle where the activities foreseen would not interfere with on-going tsetse ecological studies and East Coast fever control;
- mobilizing support from farmers in the trial area;
- conducting a pre-treatment trypanosomiasis survey and tick count;
- preparing a detailed programme of work for the duration of the project;
- implementing the trial and monitoring the effects on trypanosomiasis and tick-borne diseases and tick infestation.

1.3 Project staff

The national counterpart staff were all members of the tsetse control unit of the Livestock Development Department:

Mr. Hassan S. Hafidh, Tsetse Field Officer (Head)
Mr. Issa S. Khamis, Tsetse Field Officer
Mr. Khalfan M. Saleh, Tsetse Field Officer
Mr. Suleiman H. Salum, Tsetse Field Assistant
Mr. M.K. Masoud, Tsetse Field Assistant

1.4 Equipment and facilities

The TCP project was accommodated in the LDD premises at Maruhubi in Zanzibar Town where an office and the laboratory facilities of the Veterinary Investigation Centre were made available. Some blood examinations were also carried out at the Animal Health and Production Centre (AHPC), Mangapwani.

Two vehicles, a Suzuki SJ 410 4WD Station Wagon and, from March a Pick-up of the same make were used. On the completion of the project, the imported equipment and materials - one microscope, one microhaematocrit centrifuge, a portable generator, the remaining pour-on pyrethroid insecticide, diminazene aceturate and other veterinary consumables - were handed over to the continuing project URT/86/022. The major items are listed in detail in the Appendix. The fibreglass panels for construction of targets, the glue isopolybutylene, and diluent were provided by the LDD.

2. WORK PROGRAMME

2.1 Summary of project activities

In the preparatory phase, information obtained from the LDD, and through field visits, led to the identification of Mangapwani as a suitable trial area; a modified type of glue-impregnated target was tested and manufactured in sufficient numbers for surveying the experimental zone. During the survey phase, approximately 30 sq km were systematically monitored for tsetse flies; resident livestock owners were mobilized by means of posters and meetings, their cattle were examined for trypanosomiasis and curative treatment given when necessary. In the control phase, all available cattle, goats and donkeys were treated in five consecutive cycles at two week intervals. An area of approximately 33 sq km was thus covered by "living targets" for about three months. During the control operation, the changes in the apparent density of *G. austeni*, the trypanosomiasis incidence and the tick infestations on cattle were monitored and recorded.

2.2 Choice and description of the experimental area

The area of Mangapwani, in northwestern Zanzibar, was chosen for the pilot trial being a permanent focus of G. austeni with a large cattle population living under a high trypanosomiasis risk. It is relatively protected from reinvasion due to the large Mahonda plains and associated sugar cane plantation to the east and the sea to the west and is readily accessible from Zanzibar town (Figure 2). The experimental zone extended from the densely populated area of Misufini in the north to the Mahonda main road in the southeast and was demarcated by the road south of Kama-village in the south. The following eight administrative communities are included: Misufini (partly), Mangapwani, Zingwezingwe, Fujoni, Kiomba Mvua, Mkadini, Mfenesini (partly) and Kama. The flat open grasslands east of Zingwezingwe river around Panga Tupu, a popular grazing ground during the dry season and in all probability not tsetse-habitat, were not included.

Mangapwani is situated in the more humid western part of Zanzibar and receives between 1500 and 2000 mm annual rainfall, most during the rainy seasons from March to May and November to December. The rainfall from December 1987 to May 1988, 729 mm were recorded at the Mahonda sugar factory, was well distributed. No month was completely dry, but the rains were below normal during the main wet season, especially during March and May: 82 mm over 10 days in March, 388 mm (25 days) in April and 90 mm (15 days) in May.

Mangapwani is situated in the tree crop area and is intensively cultivated with a variety of traditional crops, such as coconuts, cloves, citrus and other fruit-trees, with an intercropping mainly of bananas and cassava. Rice cultivation is practised on the low-lying clearings. Abandoned plantations and bush fallowing have created an extensive patchwork of dense undergrowth and thickets of varying density and height, forming a suitable habitat for G. austeni. After lying fallow for usually eight to ten years, small plots of land are cleared, burnt and recultivated by the villagers until the soil fertility again declines. Bush is also an important source of firewood, charcoal and building material.

The dominant cattle breed is the small East African zebu, owned by smallholders who seldom hold more than five animals. Friesian and Jersey crosses are present in small numbers. Cattle are kept traditionally in an integrated crop and livestock farming system. They graze, usually tethered in small groups or separately, on native grasses and forages under tree crops, often near or inside the thickets, along roads and tracks, on fallow land between cassava plantations or other food crops and on the few cleared natural pastures. Conditions are generally favourable for frequent contact between cattle and tsetse. Bulls are also used as draught animals. Goats are owned by a few people, normally kept near their houses and restricted in movement. The few donkeys, probably not more than 40 in the trial area, play an important transport role in the harvesting of coconuts. Mangapwani is situated in a densely populated part of the island, with over 200 inhabitants per square kilometre.

2.3 Target design

The target designed for surveying G. austeni was based on the model used by Hall (1986), but its shape, size, finish and glue-impregnation were further modified. The shape was changed from a two-dimensional flat screen, standing on two "legs", into a hanging and freely rotating three-dimensional design composed of three screens with three legs (Fig. 3). The screens were assembled from fibreglass-panels of 0.28 x 0.45 m each, originally supplied for the manufacture of William-traps formerly used for the control of stable-flies. The three screens, each composed of two panels for the body and a half panel for the leg, were supported by three metal poles which fitted together horizontally at an angle of 120 degrees. All parts were interconnected with nylon thread, making the target foldable and easy to transport. The overall surface area was nearly doubled from 0.5 sq m for the two-dimensional model to 0.95 sq m for the new target. The white colour was retained but distemper was replaced by white oil paint with a trace of blue, which made the surface more brilliant and resistant to frequent cleansing.

After suspension on a suitable branch or twig, the targets were coated with a thin layer of isopolybutylene, a transparent and odourless glue which made the panels extremely sticky for one week or longer, and with effective persistence following rainfall. Before use, the glue was diluted with 15 percent (v/v) of "Heavy distillate" (Product 28673, BDH Chemicals Ltd., England), a less unpleasantly smelling, and presumably less repellent, substitute for the previously used kerosene.

2.4 Tsetse survey

During the tsetse survey from December 1987 to February 1988, up to ten targets were used simultaneously, deployed in over 60 different locations in and around the trial area. Generally, the targets remained in place for 2x2 days and in areas of negative captures for up to one week. The positions were chosen as close to a potential tsetse habitat as possible, i.e. in the semi-shade of patchy thickets or dense undergrowth, often at the edges of small clearings, cultivation plots and tracks. Ground clearance of the targets was between 10 and 15 cm; grass and weeds, both underneath and around them, were cut short to improve visibility. After use, they were cleaned with kerosene, soap and clear water.

2.5 Mobilization of farmers and trypanosomiasis survey

Much attention was paid to the mobilization, instruction and encouraging of the regular participation of all livestock owners in the trial area. The strategy included an introductory letter from the LDD to all administrative communities involved, a meeting with their leaders at the North B District Office under the chairmanship of the Administrative Officer, the support of the local Animal Health Assistant, the distribution of FAO posters on African Animal Trypanosomiasis in Swahili and the exchange of information through daily personal contacts with the farmers.

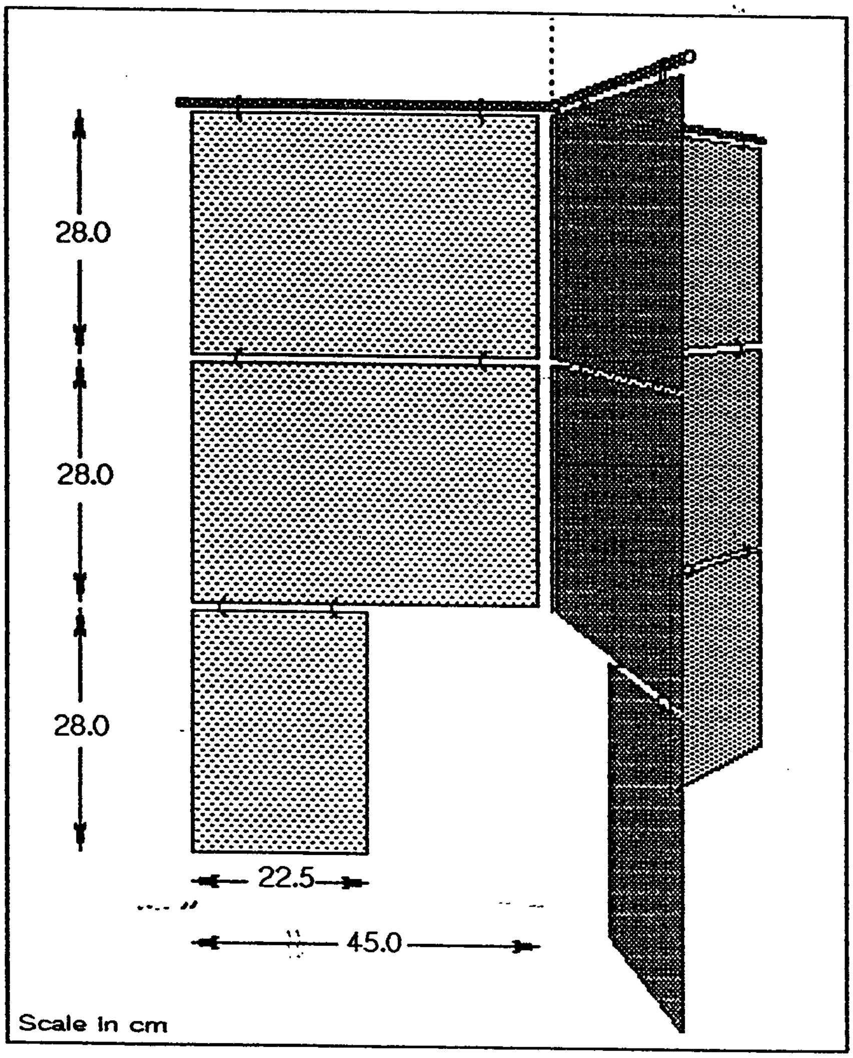

FIGURE 3: Design of the three-dimensional target, used for the monitoring of *G. austeni*.

It soon became clear that many of the cattle owners would only collaborate and allow the bleeding of their animals if treatment was provided at the earliest opportunity. Accordingly, local wound treatment and in most confirmed cases also curative trypanocidal treatment with diminazene aceturate (TRYPAZEN[R])* at 3.5 mg per kg body weight, plus 10 percent contingency, (girth weight, determined with a cattle weighing tape) were offered. Repeated blood examinations made possible the calculation of trypanosomiasis period prevalence and incidence rates.

Farmers were requested to present their stock at one of the 20 regular meeting points, normally less than one kilometre away from their home. The cattle, tethered and usually rather docile, were tied to the trunk of a coconut tree and bled from the jugular vein. Details of each owner, his cattle and other stock were recorded with the following particulars: date of examination, name of owner, location, breed of animals, sex, age, colour, examination results and further treatment. The registration made it possible to monitor the animals individually throughout the field trial.

The blood (with EDTA as anticoagulant) was kept cool and was processed within three hours. It was examined for trypanosomes and the packed cell volume (PCV) by the haematocrit centrifuge technique (HCT), using microcapillary tubes of 75 mm standard lengths. After four minutes of centrifugation at 12 000 rpm the capillary tubes were checked directly at x200 magnification using the maximum contrast. The identification of T. congolense and T. vivax was routinely made by their different movement behaviour; in doubtful cases recourse was made to Giemsa-stained thin blood film preparations. Trypanosoma brucei was not found.

2.6 Organisation of tsetse control

The tsetse control operation, i.e. the application of the pour-on insecticide, began with the first treatment on 23 February and ended on 4 May 1988. A deltamethrin (1 percent w/v) pour-on formulation (SPOTON[R])** was applied to all available cattle older than six months, goats and donkeys in five consecutive cycles at 15 to 18 days interval. The intended dose rate was 10 ml per 100 kg of estimated body weight. On cattle, SPOTON[R] was applied with a T-bar applicator gun, adjusted to deliver 5 ml, to each side of the body along a line from the shoulders to the haunch. Smaller quantities were applied between the base of the ears, on top of the hump, at the tail base, on large dewlaps and on the scrotum of adult bulls. The intention was to reach primarily the lower parts of the body without neglecting predilection sites for ticks and for skin wounds. Donkeys were treated only on the fore and hindlegs, goats along the middle line of the back. Calves below six months were omitted purposely in order to maintain their natural exposure to endemic tick-borne infections.

Under the conditions of the pilot study, a full application cycle took about one week. This included the repetition days necessary to include the "no show" farmers. For the first two applications, the tsetse control unit operated in one group. Thereafter, two teams, each equipped with a 4WD vehicle, were formed.

* Registered trademark of Virbac, France.
** Registered trademark of Cooper Ltd., Zimbabwe.

2.7 Monitoring of the effects of tsetse control

The effects of the control operation were monitored entomologically by tsetse targets, and parasitologically by blood examinations of sentinel animals. The reduction in the apparent density of G. austeni, i.e. the number of tsetse per target per day, was regularly recorded by four targets placed in positions of highest tsetse density prior to intervention. In order to increase the probability of fly catches, additional targets were positioned also at other selected sites throughout the area.

During the second pour-on cycle, when tsetse was already considerably reduced, one third of the cattle from the trial zone and a number from neighbouring Mwakaje area were randomly chosen as sentinel animals. They were checked for PCV levels and trypanosome infections; thereafter all were treated with diminazene aceturate at 5.25 mg/kg body weight, and in rotation re-examined three, five and eight weeks later.

2.8 Monitoring of the tick infestation

East Coast fever and other tick-borne diseases are among the main causes of poor animal health on Zanzibar. The vector-ticks are omnipresent. To investigate the acaricidal effects of pour-on deltamethrin on the tick burden of cattle, tick counts were made on 20 randomly chosen zebus of different age and sex from four locations immediately before the first, third, fourth and fifth application cycle. For Rhipicephalus spp., the mean adult tick count for both ears, and for Amblyomma spp. and Boophilus spp., the average total tick counts were recorded.

3. RESULTS AND CONCLUSIONS

3.1 Distribution of Glossina austeni in the trial area

Past attempts to capture G. austeni in Mangapwani with traps or bait animals resulted in the detection of only a few flies mainly in or around the small shoreline conservation zone (LeRoux,1981; Turner, 1984; Hall, 1986). This probably gave misleading information on the extent of the infested area. The use of three-dimensional sticky targets resulted in confirmation that a much larger area was infested. It did not result in a statement of the true area density of this species.

The targets were used to assess the distribution and the relative abundance of G. austeni. Figure 4 illustrates the positions of and results obtained from all targets placed in and around Mangapwani. Between December 1987 and February 1988, 127 tsetse flies were caught in 24 sites inside the trial zone. The mean capture rate was 1.02 flies per target per day. The sex-ratio was well balanced with 65 female and 62 male flies. The apparent fly density varied considerably between target-positions and ranged from 0.14 (one fly in seven days) to 3.66 (11 flies in three days). The highest yield of one target in 24 hours was seven flies. Table 1 shows the results of six target-positions with capture rates above one tsetse per target per day.

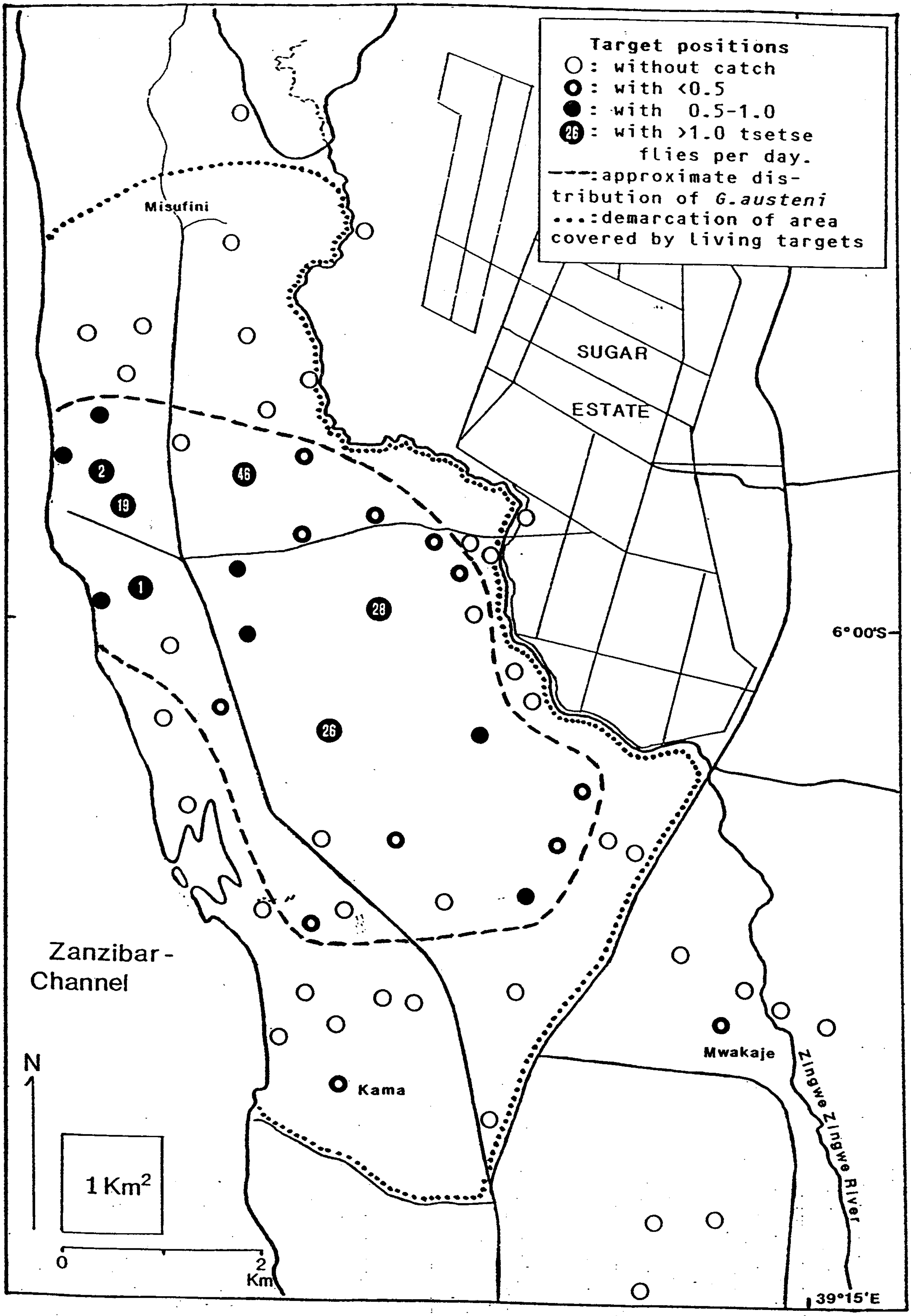

FIG.4: Approximate distribution and apparent density of *Glossina austeni* in the Mangapwani trial area; demarcation of area covered by living targets (pour-on treated livestock) during tsetse control.

TABLE 1. Capture rates and apparent density of G. austeni in target-positions 1, 2, 19, 26, 28, 46 prior to tsetse control

Target No.	Month	Target days	Total catch	Female flies	Male flies	Apparent density
1	12/87	4	13	9	4	3.25
1	2/88	3	11	3	8	3.66
2	12/87	4	8	4	4	2.00
19	1/88	6	19	7	12	3.17
19	2/88	4	13	7	6	3.25
26	1/88	4	8	6	2	2.00
28	2/88	4	7	5	2	1.75
28	2/88	6	9	5	4	1.50
46	2/88	3	8	6	2	2.66

The tsetse-infested area of some 20 sq km extended from the seashore conservation zone of Mangapwani eastwards to the edges of the Mahonda plains and sugar estate, and covered the communities of Mangapwani, Zingwezingwe, Fujoni, Kiomba Mvua and parts of Mkadini and Kama. In view of the extent of suitable all-season tsetse-habitat, i.e. evergreen thickets in all stages of regeneration on fallow land under coconut, clove and fruit trees, it is very doubtful, whether bush-clearing on a limited scale would result in any significant tsetse reduction.

Targets set up on an average of five days in 39 other, mainly more peripheral, positions caught no tsetse. Glossina austeni was not detectable north of Mangapwani towards Misufini, an area with larger plots of cassava plantations, nor in the dense riverine vegetation along Zingwezingwe River. However, two pregnant female flies were caught southeast of the experimental zone in Mwakaje, thus corroborating an earlier conclusion drawn on the basis of the trypanosomiasis disease survey in the Project Findings of URT/81/017 (1987), that Mangapwani could not be considered an isolated endemic area.

The results of the tsetse distribution surveys were used to decide the zone to be covered by the control operation. The area of Kama was included from the second pour-on cycle due to the later capture of one male tsetse near Kama-village.

Three-quarters (73 percent) of the tsetse captured preferred to land on the legs of targets, the others were recorded on the lower side of the body. The landing heights observed were very rarely more than 50 cm above ground, which made coating of the upper half of the targets with glue unncessary. The determination of age, although feasible in principle with flies captured on sticky targets, was not done, because the targets were not usually checked often enough to enable examination of the specimen in a fresh state and with minimum glue contamination.

3.2 Reduction of tsetse during the control operation

The regular pour-on applications on the majority of the resident livestock had an immediate and marked effect on the tsetse population. The reduction of G. austeni was monitored through regular placement of targets at four sites in the centre of the trial area, which were highly infested before intervention. Until immediately before the start of the control operation, the four targets in sites 1, 19, 28 and 46 caught a total of 80 tsetse in 30 target-days. During the early control phase they caught only 24 flies, of which 13 were classified as teneral. The apparent density decreased progressively from 2.67 flies per target per day prior to control to 0.62 during the second week after the first pour-on treatment, 0.29 during the fourth week after the second treatment, 0.04 during the third cycle and in the sixth week, to zero. Table 2 summarizes the fly catches and the apparent fly density recorded by the targets in the four standard monitoring positions.

The last two flies recorded, both non-teneral males, were caught on 29 March in site 28 and on 31 March, 37 days after the start of the operation, in site 26. During April and May, up to six additional targets were placed in various other sites. Up to the end of May when the Project was terminated, no tsetse fly had been caught or seen at any of the target-positions.

It is of interest to compare the negative catching result in Mangapwani with results obtained by two targets in Jozani forest between 21 and 27 April, positioned 60 metres apart on the upper forest floor. In total, 34 tsetse, 14 males and 3 females on each target, were caught, of which 13 males landed while the targets were being checked and within 20 minutes thereafter, at about 11.00 a.m. during heavy rainfall (at 26°C). This observation clearly demonstrated the efficacy of the targets under adverse climatic conditions and also a sex-specific activity of G. austeni in response to man and targets.

TABLE 2. Average capture rates and apparent density of *Glossina austeni* at target-positions 1, 19, 28, 46 before and after implementation of tsetse control in Mangapwani

Period	Target-days	Total catch	Female flies	Male flies	Apparent density
Before treatment	30	80	42	38	2.67
<Pour-on cycle 1> Day 8 - 15	26	16	10	6	0.62
<Pour-on cycle 2> Day 22 - 29	24	7	2	5	0.29
<Pour-on cycle 3> Day 33 - 40	28	1	0	1	0.04
Day 47 - 54	28	0	0	0	0
<Pour-on cycle 4> Day 60 - 67	28	0	0	0	0
<Pour-on cycle 5> Day 73 - 81	32	0	0	0	0
Day 89 - 95	24	0	0	0	0

The rapid reduction of *G. austeni* in the Mangapwani area was promoted by the following factors: the high number and uniform distribution of cattle that could be treated as "living targets" (see 3.3); low or non-availability of alternative host animals; minimal reinvasion pressure; relatively favourable climatic conditions during the period of the trial; and a smooth progression of the planned work programme. So far, the results indicate that tsetse eradication may have been achieved in the trial area. To substantiate this, sustained monitoring and protection of the zone from reinvasion will be necessary.

The decision on the number of insecticide treatments, the dose rate and the intervals of application was made with due regard to the two objectives of tsetse and tick control. The manufacturer's recommendation for tick control was followed as far as the treatment intervals were concerned. However, in order to control tsetse, the dose rates were increased by 50 percent and nonetheless were well tolerated by the animals. If the objective is limited to tsetse control, longer treatment intervals may also be considered, but at the expense of optimal tick suppression. The number of treatment cycles was determined on the basis of the reproduction biology of tsetse and the actual recorded reduction in the apparent fly density, extended by a safety margin of about one month. As the fifth pour-on cycle was carried out during week 10, the period of full insecticidal cover over the trial area is estimated at three months. The overall insecticide consumption was 81 litres for 3 112 cattle treatments plus 975 and 80 treatments of goats and donkeys respectively. The mean application per bovine amounted to 24.9 ml, 20 percent above the intended dose rate (the average body weight of the zebu cattle being 200 kg).

The potential of a new and relatively simple control technique for G. austeni has been demonstrated in a limited trial area. The persistent and rainfast pour-on insecticide formulation offers flexibility in treatment and requires a minimum of technical equipment. The potential for tsetse control is optimal where livestock are evenly distributed and are the sole or major food resource for the fly. The efficient application is also dependent on willing community participation.

A survey technique for the detection of G. austeni has been developed and improved. A reliable tsetse detection method is regarded as an essential prerequisite to any future control operation. Although an acceptable approach to an island-wide tsetse control campaign might simply be to treat cattle in all areas where trypanosomiasis occurs, a reliable detection method for low tsetse populations is indispensable, particularly with regard to the planned implementation of the density dependent SIT. The targets have shown a potential which has not yet been fully exploited. Further improvements in efficiency can be expected through the addition of olfactory attractants, as has been demonstrated for other tsetse species, or through modifications of design. Insecticide-impregnated stationary targets may also be deployed as control devices where livestock are scarce. If game, and certainly bushpigs in parts of the island, should compete with livestock as host animals, a higher number of pour-on application cycles or the temporary movement of treated cattle as decoys into critical zones would be advisable. Limited persistent groundspraying with a synthetic pyrethroid insecticide might also be considered where environmentally acceptable, e.g. as a temporary barrier between two treatment blocks.

3.3 Participation of farmers

The average number of cattle per farming family in Mangapwani is three. Large numbers of farmers in the trial area were kept regularly informed on control activities and were mobilized as described under 2.5. In the initial survey, 153 farmers with their 472 cattle actively participated. During the control phase, the number increased to 238 farmers with 695 cattle, about 200 goats and a few donkeys (Table 3).

TABLE 3. Participation of farmers with their livestock during the trypanosomiasis survey and the pour-on application cycles 1 to 5

COMMUNITY	SURVEY		CYCLE 1		CYCLE 2		CYCLE 3		CYCLE 4		CYCLE 5	
Mangapwani	*71	**29	89	38	76	31	85	36	74	30	83	33
Misufini	48	17	66	28	77	33	102	38	97	32	147	48
Zingwe-zingwe	113	26	98	22	102	24	100	26	98	24	104	24
Fujoni	85	35	85	38	82	39	97	43	83	37	91	40
Kiomba Mvua	80	23	80	30	69	23	80	26	73	27	77	29
Mkadini/ Mfenesini	75	23	99	29	112	34	124	38	121	36	107	34
Kama	-	-	-	-	79	27	82	24	77	25	86	30
TOTALS	472	153	527	185	597	211	670	231	623	211	695	238
Cattle per farmer	3.08		2.85		2.83		2.90		2.95		2.92	
TOTALS Goats	-		183		215		195		198		184	
Donkeys	-		21		14		18		12		15	

* number of cattle
** number of farmers

The number of treated cattle per sq km ranged between 18 and 21 per cycle. It can be assumed that practically all cattle owners and stock in the experimental zone were registered and that the participation per cycle was above 85 percent. A greater degree of fluctuation in the number of willing participants was common in the border areas. Cattle owners depend mainly on other activities for their livelihood, such as cultivating, fishing, trading or casual employment. Therefore, they did not always keep to scheduled appointments. The necessary high degree of participation was only obtained through the repeated mobilization and meticulous registration of cattle owners and stock, and forewarning of all intended treatment dates. Goats and donkeys were included because of their likely role as secondary tsetse hosts (see under 3.4), but treatment of all individuals was not insisted on. In view of the numerous livestock owners that would need to be included in an island-wide tsetse control operation, the availability of a personal computer, with suitable standard software, would greatly facilitate and improve the efficiency of the registration and recording system.

3.4 Trypanosomiasis survey, treatment and control

Before the introduction of tsetse control, trypanosomiasis morbidity was high and stock losses in the area were probably more often caused by this infection than by tick-borne diseases. The blood examination of most cattle during the survey confirmed the magnitude of the problem. Out of 472 bovines sampled, 217 or 46 percent were positive by the HCT, either with *Trypanosoma congolense* (55 percent), *T. vivax* (37 percent) or both. The ratio fluctuated slightly, depending on the time of examination during the study and sample size. In a second examination of 248 cattle, 120 or 48.4 percent were positive, of which 66.7 percent were diagnosed as *T. congolense*-infections (Table 4). *T. vivax*, formerly not reported in such proportions on Unguja Island, was the dominant species in some localities.

TABLE 4. Trypanosomiasis infection rates and packed cell volumes (PCV, mean and standard deviation) of 248 cattle in the Mangapwani trial area before tsetse control

	Cattle	Percent	PCV (%)
Sample size	248		24.7 +/- 5.6
Negative HCT-result	128	51.6	27.1 +/- 5.0
Positive HCT-result	120	48.4	22.0 +/- 5.3
**Infected with:**			
Trypanosoma congolense	80	66.7	22.3 +/- 5.2
Trypanosoma vivax	21	17.5	24.3 +/- 4.2
T. congolense/T.vivax	19	15.8	18.4 +/- 5.3

While no difference in the trypanosomiasis infection rates of light or dark coloured animals was observed, an age-related difference was noted. Infection rates were higher in one to two year-old cattle (57 percent) than in those under one year (47 percent) or above two years (44 percent).

The role of goats as trypanosome carriers was also investigated: 65 blood samples were collected randomly in Mangapwani and Zingwe-zingwe. Fourteen (21.5 percent) were found positive with T. congolense (11) or T. vivax (3). However, the animals affected were confined to only a few of the flocks examined. Trypanosoma congolense infections were also diagnosed in two obviously sick donkeys both with PCV-values below 15 percent, one of which died whilst the other responded to treatment.

An estimate of the trypanosomiasis incidence rate in Mangapwani was made on the basis of 150 individual cattle which were examined and, if positive, immediately treated and re-examined within less than two months, though at slightly differing intervals (Table 5). During this period, 38 percent were either reinfected or newly infected, 32 percent were cured and 30 percent remained negative.

TABLE 5. Trypanosomiasis incidence and packed cell volumes (PCV) in 150 cattle in the Mangapwani trial area, examined during the survey, treated if positive with diminazene aceturate and re-examined after mean intervals of 34 to 49 days.

Cattle	n	%	T.congolense	T.vivax	Both spp.	Interval (days)	1st PCV %	2nd PCV %
Cured	48	32	32 -	15 -	1 -	34+-6*	21.5+-4.0*	27.0+-3.9*
Reinfected	17	11.3	11/12**	5/2	1/3	40+-5	21.3+-2.7	23.1+-4.3
Newly infected	40	26.7	- 30	- 5	- 5	49+-13	25.9+-4.5	23.5+-5.6
Remained negative	45	30	- -	- -	- -	45+-12	25.5+-4.7	26.6+-3.9

* Mean and standard deviation
** Number of cases during first and second examinations

These results reflect an effective break in the transmission of trypanosomiasis through the application of the pour-on insecticide in the Mangapwani trial zone. They contrast sharply with observations made during the same period in the adjacent untreated area of Mwakaje where every second bovine, i.e. 19 of 37 cattle examined, was either re-infected or newly infected either with T. congolense (7 cases), T. vivax (10) or both.

The situation in the Mangapwani trial zone changed following tsetse control operations. The risk of trypanosomiasis transmission and infection seemed virtually eliminated. None of the 144 cattle in the sentinel herd was found positive when examined three, five and eight weeks after curative trypanocidal treatment.

The negative correlation between trypanosomiasis and PCV-value was also demonstrated. The PCV of positive animals was generally lower than that of uninfected cattle. Although the individual data fluctuated considerably from mean values, the trends were clearly demonstrated. Table 4 shows the particularly depressing effect on the PCV of mixed T. congolense and T. vivax infections. Table 5 shows the dependence of the PCV-values on the status of infection in a sample of 150 cattle before tsetse control had been achieved. While the mean PCV rose by 5.5 percent in the cured animals, the opposite trend was recorded in newly infected cattle. Analogous results were obtained in 144 sentinel cattle after the start of tsetse control. Within eight weeks after curative treatment, their mean PCV rose from 24.2 to 29.1 percent. Not only did the anaemic state of the animals return to normal, but their general appearance also improved.

3.5 Tick control

Generally, the tick burden on cattle in the Mangapwani trial area was low to moderate before intervention. Tick control was only practised by a few farmers and then at irregular intervals. The bi-monthly application of pour-on deltamethrin controlled ticks well (Rhipicephalus spp.) to very well (Amblyomma spp., Boophilus spp.). On twenty sentinel cattle, the average count of adult Rhipicephalus spp. on the ears dropped from an initial 16.5 to 4.5, 1.8 and 1.2 when checked before the third, fourth and fifth applications respectively. The mean total of adult Amblyomma spp. dropped from 8.8 to 1.7, 0.5 and zero, while Boophilus spp. with a one-host-life-cycle fell quickly from a mean of 4.9 adults to none. Combined with very beneficial effects on the healing of skin wounds, which were found mainly on the hump of about 20 percent of the zebu cattle (probably caused by ticks and maintained by irritating flies and birds), the good acaricidal properties were quickly visible and convincing to farmers. This played an important role in ensuring their cooperation. A sustained effect, after the regular applications within the pilot trial have ceased, cannot be expected. The average cost of 0.6 US$ per dose, as applied in this trial, makes the continuous use of SPOTON[R] for tick control in the traditional sector economically unjustifiable, unless eradication can be achieved.

3.6 General conclusions

The pilot control trial can be regarded as the first step in an island-wide operation leading to the ultimate eradication of tsetse and trypanosomiasis on Unguja Island. The feasibility of the method for the effective control and possibly eradication of G. austeni has been demonstrated in a defined area. This relatively simple and environmentally safe technique, which was also generally acceptable to the farmers, offers

good prospects for successful application in other parts of the island. It will, therefore, provide the basis for a similar technical approach within the objectives of the recently established project URT/86/022. However, in view of its intended large-scale use, the possible limitations of the method and the practical implications have not yet been clearly defined with regard to (a) a final assessment of whether eradication can be achieved by this method alone, and (b) the possible need to integrate with other control methods under varying ecological conditions.

An island-wide tsetse control operation requires the preparation of a detailed programme and the coordination of activities between the LDD under the Ministry of Agriculture as executing agency, the UNDP-funded FAO project URT/86/022 - "Animal Disease Control" and the IAEA-supported project "Tsetse Fly Eradication by SIT" at TTRI, Tanga. It can be anticipated that the necessary equipment, vehicles, consumables and operational funds will be procured by the projects involved. The staff situation is regarded as more critical. The tsetse and trypanosomiasis control personnel within the LDD is at present insufficient to fulfill all medium and long-term technical responsibilities required by an island-wide operation. A senior national tsetse and trypanosomiasis control specialist is not yet available.

Two, or preferably three operational teams are considered to be the minimum staff requirement for the tsetse and trypanosomiasis surveys and for the control operation. Each should consist of one specialized field officer, two assistants and one driver, under the guidance of a tsetse and trypanosomiasis control specialist and a national counterpart. To cover large areas during the control operation, local help could be sought at community level to assist in the insecticidal treatment of cattle providing good supervision is ensured.

4. RECOMMENDATIONS

4.1 In order to achieve a final evaluation of the pilot trial, as to whether control or eradication of tsetse has been achieved, it is recommended that the regular monitoring of the tsetse population and the search for trypanosomiasis in the sentinel animals should continue until a definite conclusion is possible. The supposed absence of G. austeni from the peninsula north of Misufini has to be confirmed. Protection of the area against re-invasion of tsetse should be provided through a barrier consisting of treated cattle maintained across possible reinvasion routes, mainly in the southeast towards Mwakaje and Kitope. Any tsetse catches made in this vicinity would justify immediate concerted control efforts. The future capture of tsetse inside the Mangapwani zone would certainly justify a repeat of the original trial, probably of longer duration.

4.2 It is recommended that the tsetse and trypanosomiasis survey be extended systematically into adjacent areas and other parts of North B District. Exact distribution maps and quantitative data on apparent tsetse density and trypanosomiasis prevalence should be produced for areas at risk. The accurate northern limit of the distribution of G. austeni should be defined, from which any intended large-scale operation should start and

proceed progressively in a southerly direction. Results of the recent island-wide trypanosomiasis survey suggest the presence of tsetse in parts of Mahonda, Kitope, Mgambo and Pangeni of North B District. These communities and the untreated area of Mfenesini and Mwakaje, adjacent to the Mangapwani trial zone, could successively be incorporated into the phased tsetse control operation. For assessment of the control efforts in all areas, direct and indirect detection methods, such as the glue-impregnated targets, fly-rounds with bait-animals, and sentinel cattle herds should be employed.

4.3 It is recommended that goats and donkeys be included in future insecticide applications, and that the role and relationship of domestic animals and game in the epidemiology of trypanosomiasis under different ecological conditions on Zanzibar be further investigated.

4.4 It is recommended that trypanocidal curative treatments in livestock generally be restricted to confirmed emergency cases and block treatments within the control operation. They should be carried out by qualified personnel, to avoid indiscriminate drug use and to minimize the risk of development of drug-resistant strains of trypanosomes.

4.5 It is recommended that the organisation of future survey and control operations be based on a computer-assisted livestock and livestock owners' register. It should process all data on livestock density, distribution, migration, grazing pattern, productivity, disease prevalence, participation of the farmers and other factors relevant to the smooth running of the operation. It may also facilitate the production of a final cost-benefit analysis.

4.6 It is recommended that emphasis be put on further development and improvement of the target-technique, which has already become useful in the detection and monitoring of G. austeni. A promising potential is the addition of tsetse attractive odours. As a stationary toxic device, the same type of target with insecticidal impregnation could also play a direct role in tsetse control where cattle are scarce and other hosts such as bushpigs are present. Efforts to improve traps for both survey and control of G. austeni should continue.

4.7 It is also recommended that the persistence of the deltamethrin pour-on formulation be tested under Zanzibar conditions. Longer intervals between applications could then be considered accordingly. The necessary bioassay tests could be done in collaboration with TTRI, Tanga. The institute could also assist in a mark-release-recapture exercise to assess more precisely the true population density of G. austeni and the efficiency of various survey techniques.

4.8 It is strongly recommended that the existing national tsetse control unit be strengthened to an adequate level as suggested under 3.6. The training of personnel, both on the job and through external courses, should be given priority. In appreciation of the practical demands and hardship conditions inherent in the execution of tsetse control operations, the provision of field allowances for the compensation of overtime and hardship in the field should also be considered.

4.9 It is finally recommended that regular meetings be initiated and held at senior technical level of the national and international institutions and organizations involved, in order to coordinate and streamline the various activities and encourage further efforts aimed at the elimination of tsetse and trypanosomiasis in Zanzibar.

5. ACKNOWLEDGEMENTS

I am grateful to Mr. Kassim M. Biwi, Director, and Dr. Iddi A. Shambwana, Deputy Director of the Livestock Development Department, Zanzibar, for their kind support and the provision of facilities. I wish to thank all staff members of the tsetse control unit for their continuous assistance, often outside office hours, in particular Mr. Hassan S. Hafidh, whose professional knowledge, local experience and diligence were great assets during the implementation of the pilot trial. I am also indebted to Mr. Said Kassim, Veterinary Assistant, AHPC Mangapwani, for the help rendered in the field, and to many other members of the LDD, of the North B District Administration and the local communities for their support of this work.

6. REFERENCES

Bouvry Stratford, M. 1986. Cattle trypanosomiasis situation on Zanzibar. FAO Livestock Development Project, URT/86/017, Field Document No. 7, Zanzibar.

Hafidh, S.H., Khamis, I.S., Salum, S.H., & B. Stratford, M. 1987. Background data for the control of cattle trypanosomiasis on Zanzibar, Tanzania. FAO Livestock Development Project, URT/86/017, Field Document No. 10, Zanzibar.

Hall, M.J.R. 1986. A study of methods for the survey of the tsetse fly, Glossina austeni Newst., on Zanzibar Island. Final report to the International Atomic Energy Agency.

Hursey, B.S., Whittingham G.W., Chadenga V. The integration of insecticidal techniques for the control and eradication of Glossina morsitans in North-east Zimbabwe. In press.

LeRoux, J.G. 1981. The status of tsetse and trypanosomiasis on Zanzibar Island. FAO Mission Report (AGAH), Rome.

Tatchell, R. 1976. Report of the tick control consultant, AGO/URT/73/025. UNDP/FAO Project: Livestock Production, URT/73/025, Zanzibar. Rome.

Turner, D.A. 1984. The distribution of the tsetse fly, Glossina austeni Newstead on Zanzibar (Unguja) Island, Republic of Tanzania: a report of a survey carried out between November 1983 and February 1984. IAEA Report.

UNDP/FAO 1987. Project URT/81/017, Livestock Development - Phase III, Zanzibar. Project Findings and Recommendations, Terminal Report, AG:DP/URT/81/017, Rome.

APPENDIX: List of supplied equipment and material

The list specifies important equipment and material which was supplied by FAO for the execution of the pilot trial:

1 Microscope LEITZ Laborlux K with objectives EF 10/0.25, Plan 20/0.45, EF 40/0.65, 100/1.25 oel, eyepieces PERIPLAN x 10/18; mains supply 110-220 V and 12 V car adaptor (Fed. Rep. of Germany).

1 HAWKSLEY microhaematocrit centrifuge 01500, 220/240 V, with reader 01502, heparinised capillary tubes 01603 and sealant 01503 (UK).

1 HONDA EG-1400XR 1.2 KVA generator (Japan).

110 litres of deltamethrin (1% w/v) pour-on, SPOTON®, manufactured by Cooper (Zimbabwe) Ltd., with T-bar applicator gun (adjustable).

150 x 10.5 g diminazene aceturate, TRYPAZEN®, manufactured by Virbac (France).

Cattle and pig weighing tape, Dalton Supplies Ltd. (UK).
