# Supplementary material for: Sterile Insects to Enhance Agricultural Development: The Case of Sustainable Tsetse Eradication on Unguja Island, Zanzibar, Using an Area-Wide Integrated Pest Management Approach
Source: PLoS Negl Trop Dis. 2014 May 29;8(5):e2857. doi: 10.1371/journal.pntd.0002857 (PMC4038469; doi:10.1371/journal.pntd.0002857)
Supplement: Supporting Information S2 — Livestock and agriculture development in Zanzibar, post-tsetse eradication: A follow-up socioeconomic study. International Atomic Energy Agency confidential report by N. S. Y. Mdoe, Vienna, June 2003. (PDF) [file pntd.0002857.s002.pdf]

LIVESTOCK AND AGRICULTURE DEVELOPMENT IN ZANZIBAR, POST-TSETSE ERADICATION: A FOLLOW-UP SOCIO-ECONOMIC STUDY

Draft Report prepared for IAEA by:

Ntengua S.Y. Mdoe

Department of Agricultural Economics and Agribusiness
Sokoine University of Agriculture, Morogoro, Tanzania.

June , 2003

SUMMARY

The primary purpose of this follow-up survey was to collect data on the current livestock and agriculture situation in Unguja, Zanzibar for assessing changes that have occurred since the last economic assessment survey in 1999. The approach adopted consisted of a review of the relevant recent documents on livestock and agriculture development in Zanzibar, collection of data on relevant parameters from these documents; informal discussions with key informants including government officials, design of a farm level survey using a formal questionnaire and design of a market level survey using check lists. The data collected were coded, entered in access database and analysed using the Stata software.

Based on the results of the 2002 follow-up survey and the results of the 1999 economic assessment survey, there is the general conclusion that the livestock and agriculture situation in Unguja, Zanzibar has improved since the eradication of tsetse and trypanosomosis in 1997. Whereas the 1999 economic assessment survey showed that the livestock and agriculture situation in Unguja has improved significantly from what it was before the initiation of tsetse/trypanosomosis control/eradication in 1985/86, the follow-up survey of 2002 indicate that livestock and agriculture production have continued to improved during the past three years (1999-2002). This suggests that eradication of tsetse and trypanosomosis has opened opportunities for increased livestock and agricultural production in Unguja, Zanzibar.

At the macro-level, improvement in livestock and agricultural production is evidenced by increased growth in livestock and agriculture. The contribution of agriculture to overall GDP increase from 34% in 1999 to 39% in 2001. The relative increase in the agricultural sector contribution to the overall GDP during the last three years is due to an increase in both crop and livestock production. Whereas production of crops such as paddy, cassava and bananas has respectively increased by 57%, 14% and 91%, production of milk, beef and chicken have increased by 6%, 7% and 8% respectively between 1999 and 2002.

At the farm (micro-) level, land areas cultivated for most crops in 2002 and 1999 do not vary significantly from those cultivated in 1999 although relatively more cultivated land in 2002 was allocated to the crops given top priority as cash crops and food crops. With the exception of coconut, cloves, rice and yams whose land areas declined, land areas under cassava, sweet potatoes, yams and maize increased slightly or remained the same while land areas for vegetables and plantain/banana increased substantially.

Whereas yields achieved by farmers in 2002 for cassava, rice, maize, coconut and vegetables increased from their 1999 levels, yields for bananas, sweet potatoes, yams and cloves declined from their 1999 levels. This suggests that productivity of cassava, rice, maize, coconut and vegetables has increased while that of bananas, sweet potatoes, yams and cloves has declined during the past three years. The increase in the productivity of cassava, rice, maize, coconut and vegetables may be attributed to the use of improved seed varfieties and manure for crop production. Although most of the farmers interviewed used local seed varieties for most of the crops during the 2002 cropping season, improved seeds were used for maize, rice, vegetables, fruits and coconuts. With regard to livestock, not only has the relative proportion of farms raising cattle and small ruminants increased in

2002 compared to 1999, the number of farms with improved cattle breeds has also increased. Evidence for increased intensification of livestock and agricultural activities in Unguja is provided by the increased crop-livestock integration through more farmers using manure for their crop production while in turn using crop by-products to feed their animals. The use of animal power for ploughing and transport activities has also increased during the past three years.

Although it is currently inappropriate to use criteria such as benefit-cost ratio to assess the impact of tsetse control/eradication, comparison of the performance of livestock and crop production before tsetse control/eradication with the post-tsetse (1997-2002) performance suggest that the eradication of tsetse and typanosomosis in 1997 has resulted in significant gains including increased milk production, manure and power for ploughing and transport activities. Of particular importance is the fact that some of the milk produced is consumed at home. On average, about 20% of the milk produced per farm is consumed at home as raw milk and about 1% is consumed as fermented milk. This suggests that the increase in milk production following tsetse and typanosomosis eradication has also contributed to improvement of the nutritional status of rural households in Unguja, Zanzibar. Another evidence of the positive socio-economic impact of tsetse and trypanosomosis eradication is the increase in the average household income and the proportion of farmers who moved from low-income to high-income strata since 1999. The average household income has increased by almost 30% from 41,232 TSHS in 1999 to 53,502 TSHS in 2002. Although it is difficult to isolate the effects of tsetse eradication from other factors since several factors may have contributed to the increase in household income, it is still logical to attribute the increase in household income to tsetse and trypanosomosis eradication. A strong correlation was observed between household income and milk yields, milk sales, use of manure and animal power for cultivation and transport. This correlation suggests that the increase in the household income between 1999 and 2002 is associated with tsetse eradication in the Unguja island.

Although no attempt is made in this report to use criteria such as benefit-cost ratio to assess the economic impact of tsetse eradication in Unguja, Zanzibar, the use of such criteria economic impact assessment in future would require accurate data on total population of important livestock species (cattle, goats, sheep and donkeys). Therefore a livestock census needs to be undertaken to obtain accurate information that will be used to assess economic impact.

Despite the observed increase in livestock production and productivity following eradication of tsetse and trypanosomosis, assessment of the aggregate production and demand for livestock products indicate that demand falls short of supply of livestock products such as milk. Comparison of current (2002) production as well as projections with current and future demand indicates that the domestic consumption needs are significantly higher than domestic supply. This suggests that Zanzibar will continue to experience a deficit of milk and will continue to rely on imports unless concerted efforts are made to increase domestic production. However, continued reliance on food imports cannot be sustained and could impact negatively to both livestock and agricultural production. Therefore deliberate efforts must be made by Zanzibar authorities in collaboration with private sector and other stakeholders to tackle constraints which are affecting the livestock and agricultural sector. These constraints include poor livestock and

crop extension services, processing and marketing of crop and livestock products, diseases and low usage of inputs and improved crop and livestock varieties. Although the IAEA has intervened by promoting a heifer in trust scheme and establishment of improved fodder, these efforts need to be extended to other villages in the island. Further interventions by the government and other parties interested in livestock development that would be required to improve and sustain livestock production include:

- Promoting the use of AI and bull services
- Strengthening animal health service in the rural areas by involving the private sector in the provision of veterinary services together with AI services.
- Establishment of a revolving fund for small loans to private veterinarian to purchase basic facilities, veterinary drugs and vaccines.
- Promoting the use of botanicals to treat some of the diseases
- Farmer training on proper feeding management including the importance of supplementary (concentrates, mineral) feeding
- Low cost feed formulation
- Feed conservation techniques for use during the dry season when natural grass supply is inadequate
- Promoting farmers' organizations
- Farmer empowerment.
- Establishment of milk collection centres in rural areas
- Training on hygienic handling of milk including promoting use of aluminium containers for milking and marketing milk
- Promotion of small scale milk processing in rural areas.

TABLE OF CONTENTS

SUMMARY ii
TABLE OF CONTENTS v
LIST OF TABLES vii
1.0 INTRODUCTION 1
1.1 Objectives 1
2.0 METHODOLOGY 3
2.1 Study area 3
2.2 Data collection 3
2.2.1 Secondary data 3
2.2.2 Farm level data 3
2.2.2.1 Development of the questionnaire 3
2.2.2.2 Selection of sample farm households 4
2.2.2.3 Questionnaire administration 4
2.2.3 Market level data 5
2.3 Data compilation and analysis 5
3.0 RESULTS 7
3.1 Household socio-economic characteristics 7
3.1.1 Age and sex distribution 7
3.1.2 Education and religious affiliation 8
3.1.3 Household size and structure 8
Table 3 shows the household structure by age and sex category. The average household size was 6.9 persons with a maximum of 36 persons and a minimum of one person. North-A District had the largest average household size of 8.2 persons while Central and South Districts had the smallest size of 6.2 persons each. Each household had an average of 2 adults and 4 children. These results suggest that the average household size obtained in this study is lower than that obtained in 1999. 8
3.1.4 Occupational distribution 9
3.1.5 Household income 9
3.2 The Status of Livestock and Crop production in Unguja and changes that have occurred since 1999 10
3.2.1 Livestock production 11
3.2.1.1 Livestock species raised 11
3.2.1.1.1 Cattle 11
3.2.1.1.2 Sheep and goats 12
3.2.1.1.3 Chickens 12
3.2.1.1.4 Donkeys 12
3.2.1.2 Dairy production 13
3.2.1.2.1 Milk production with indigenous cattle 13
3.2.1.2.2 Milk production with improved cattle 14
3.2.2 Crop production 14
3.2.2.1 Types of crops produced and their importance 14
3.2.2.2 Acreage cultivated 16
3.2.2.3 Quantities of crops harvested and crop yields 17

3.3 Intensification of livestock and crop production 18
3.3.1 Availability and farmers preference for improved livestock breeds 18
3.3.2 Adoption of improved livestock breeds and crop varieties................................ 19
3.3.3 Access to land resources 21
3.3.4 Use and access to improved seeds 22
Table 23: Availability of improved seed varieties in Unguja, 2002 23
Table 24: Average prices paid by farmers for improved seed varieties in Unguja, 2002 23
3.3.5 Integration of crops and livestock 23
3.3.6 Adoption of animal power 25
3.3.7 Dairy intensification 25
3.3.7.1 Dairy cattle productivity 26
3.3.7.2 Dairy cattle feeding practices 26
3.3.7.3 Dairy cattle breeding practices 28
3.3.8 Milk processing and marketing at the farm level 28
3.3.8.1 Milk processing 28
3.3.8.2 Milk marketing 29
3.4 Animal disease and other constraints to dairy intensification in Unguja, Zanzibar .. 30
3.5 Assessment of overall domestic production, consumption needs and current market for dairy products in Zanzibar 32
3.5.1 Domestic milk production 32
3.5.2 Assessment of total consumption needs (demand) for milk in Zanzibar 32
3.5.3 Assessment of the current market for milk and milk products in Unguja, Zanzibar 33
3.5.3.1 Dairy products purchased and consumed 33
3.5.3.2 Preferences for milk and milk products 34
3.5.3.3 Quantities of milk and milk products consumed 35
4.0 Socio-economic Impact of Tsetse Eradication in Unguja, Zanzibar 37
6.0 Documents Reviewed 46
Type of animal 49
Type of animal 50
Type of animal 51
Milk marketing and processing 53
Milk Products 59
1. Sources of milk /milk products; 59
Milk Products 59
A 5.3. QUESTIONNAIRE FOR HOUSEHOLD CONSUMERS 60
Milk and milk products 60
Local products; 61
(a) Highly preferred …….. (b) Moderately preferred …… (c) Less preferred …… 61
Raw products; 61

LIST OF TABLES

Table 1: Sex and age distribution of household heads.. 8
Table 2: Educational level and religious affiliation of household heads.............................. 8
Table 3: Household size and structure by age and sex category... 9
Table 4: Distribution of household heads by occupation... 9
Table 5: Average household income and frequency distribution of household income..... 10
Table 6: Proportion (%) of farms with livestock in Unguja, 2002 12
Table 7: Average herd /flock sizes for cattle, chickens, sheep and goats in Unguja, 2002 13
Table 8: Milk production within the traditional sector in Unguja, Zanzibar, 2002........... 13
Table 9: Milk production within the improved dairy sector in Unguja, Zanzibar, 2002... 14
Table 10: Food crops considered as important by farmers 15
Table 11: Cash crops considered as important by farmers 15
Table 12: Land area (acres) cultivated for important food and cash crops after tsetse intervention in 1999 in Unguja, Zanzibar 16
Table 13: Changes in average land areas cultivated before and after tsetse eradication (1999 and 2002) in Unguja, Zanzibar 17
Table 14: Quantities of food and cash crops harvested from cultivated areas after tsetse eradication in 2002 in Unguja, Zanzibar... 17
Table 15: Food and cash crop yields obtained after tsetse intervention in Unguja, Zanzibar (Kg/acre), 2002.. 18
Table 16: Farmers views on the availability of improved livestock species in Unguja, Zanzibar.. 19
Table 17: Farmers' preference for improved livestock breeds and reasons 19
Table18: Sources of improved livestock species in Unguja, Zanzibar............................. 20
Table 19: Important constraints to the acquisition of improved livestock breeds in Unguja, Zanzibar.. 20
Table 20: Average prices paid by farmers for improved livestock breeds in Unguja, Zanzibar, 2002.. 21
Table 21: Change in land area cultivated during the last three years (1999-2002)............ 22
Table 22: Use of Improved seeds in Unguja in 2002... 22
Table 25: Feed stuffs fed to livestock in Unguja, Zanzibar, 2002..................................... 24
Table 26: Use of animal manure for crop production.. 24
Table 27: Use of farm animals for traction and transport, 2002.. 25
Table 28: Production and reproduction parameters for dairy cattle in Unguja, Zanzibar, 1999.. 26
Table 29: Feeding systems and feed stuffs fed to dairy cattle in Unguja, Zanzibar, 2002 27
Table 30: Cost of feed staffs used, 2002.. 27
Table 30: Breeding methods for dairy cattle and sources of breeding materials in Unguja, Zanzibar, 2002.. 28
Table 31: Quantities of milk sold in litres per farm per day, 2002.................................. 30
Table 32: Proportion of milk passing through different marketing channels in Unguja, Zanzibar.. 30
Table 33: Prices of milk received by farmers during wet and dry season, 2002 30
Table 34: Farmers' ranking of the importance of animal diseases currently affecting livestock in Unguja.. 31
Table 35: Effects of East Coast Fever (ECF) in Unguja, 2002.. 32

Table 36: Projections for milk production per capita in Unguja, Zanzibar, 1986-2015.... 33
Table 37: Reasons given by sample households for and against local and imported dairy products 35
Table 38: Zanzibar: Quantities of major dairy products consumed in hotels 35
Table 39: Zanzibar: Quantities of dairy products consumed by households during wet and dry season 36
Table 40: Zanzibar: Quantities of dairy products consumed by households during wet and dry season 36
Table 41: Comparison of average household income and proportion of households by income categories 39

LIST OF ANNEXES

Annex 1 47
Annex 2 48
Annex 3 56
Annex 4 57
Annex 5 59
Annex 6 62
Annex 7 63

1.0 INTRODUCTION

Agriculture is the most important sector of the economy of Zanzibar. In 1997, the year when tsetse flies were eradicated in Unguja, agriculture contributed 38% (37,997 Million TShs) to the GDP (99,208 Million TShs) (measured at current market prices) and 75% of foreign exchange earnings (FAO, 1999; Zanzibar, URT, Department of Statistics, 1999). Although both agricultural GDP and overall GDP increased from 37,997 Million TShs and 99,208 Million TShs in 1997 to 47,255 Million TShs and 139,833 Million TShs in 1999 respectively, the share of agriculture in the GDP declined from 38% in 1997 to 34% in 1999. Thereafter, the contribution of agriculture increased to 39% (81,812 Million TShs) of the overall GDP of 208,085 Million TShs in 2001. During the same period, agriculture's contribution to foreign exchange earnings increased from 75% in 1997 to 79% of foreign exchange earnings in 2001 (MFEA, 2002). The relative increase in agricultural sector contribution during the past three years is due to an increase in both crop production (especially food crop production) and livestock production. For example, production of paddy, cassava and bananas increased by 57%, 14% and 91% respectively between 1999 and 2001. On the other hand, production of milk, beef and chicken has increased by 6%, 7% and 8% respectively between 1999 and 2001 (Ministry of Agriculture, Livestock and Natural Resources, 2002). The increase in crop and livestock has increased domestic food production and consequently decline in food imports. Expenditure on food imports has declined from 39,158 Million TShs. in 1999 to 25,308 Million TShs. in 2001 (MFEA, 2002). Although expenditure on food imports has declined, food imports still form more than 40% of total imports. In 1999, food imports formed 44% of total imports, declining slightly to 42% of the total imports in 2001 (MFEA, 2002). This reflects the need to improve and intensify crop and livestock production in Zanzibar in order to increase domestic production.

Prior to the eradication of tsetse flies in 1997, trypanosomosis was the most important disease constraining livestock production in Zanzibar. Following eradication in 1997 a study was undertaken in 1999 to assess the economic impact of the intervention on livestock and agriculture development (Tambi *et al.*, 1999). Tambi et al. (1999) observed increases in productivity of livestock and some crops as well as increased crop-livestock integration at the farm level. The present study is a follow-up to the 1999 study to assess changes that have occurred in livestock and agriculture development since 1999. The study reports information collected from secondary sources and from a sample survey conducted in December 2002-January 2003.

1.1 Objectives

The primary purpose of this study is to assess changes in the livestock and agriculture development that have occurred since the last socio-economic study in 1999 to get a measure of the impact of the tsetse eradication. Specific objectives of the study are to:

(i) summarise the status of livestock and agriculture development in 2002, highlighting indicative key parameters that show changes in livestock numbers, productivity and performance; quantifying and qualifying the changes that have occurred since 1999;

(ii) evaluate the potential for the development of the dairy sector on Unguja taking into account the economics and availability of feed resources on the island and on the mainland;

(iii) identify other constraints for the development of the dairy sector;

(iv) analyse the current market for dairy products on Unguja and the potential development of a local market for dairy products taking into account the situation of the dairy sector in East Africa; and

(v) make an estimate of the impact of disease control on the dairy sector and assess its sustainability.

2.0 METHODOLOGY

2.1 Study area

Zanzibar comprises 10 districts of which 6 districts are in Unguja island and the rest are in Pemba island. This study was carried out in the five districts of Unguja, namely North A, North B, Central, South and West (Figure 1). These were the same districts that were covered during the 1999 socio-economic survey, which was the first socio-economic study to assess the livestock and agriculture situation after tsetse eradication in 1997.

To be able to assess changes that have occurred since 1999, the present study purposely sampled all the 50 villages surveyed during the 1999 socio-economic survey. However, 5 villages which are part of the recent heifer in trust project were purposely added to capture recent developments in the dairy sector. Thus, a total of 55 villages were selected for the survey (Annex 1).

2.2 Data collection

Upon arriving in Unguja in mid December 2002, the consultant met with the Director for Agriculture and Livestock and other senior staff of the Department of Agriculture and Livestock (DAL) to discuss the logistics and facilitation of the data collection exercise. The data collection exercise began after discussions with the staff and training of enumerators. Three types of data were collected, namely secondary, farm level and market level data.

2.2.1 Secondary data

Following the discussions with the Agriculture and Livestock Department staff and training of enumerators, a series of visits were made to the Ministry of Agriculture, Natural Resources and Cooperatives, the Department of Statistics, Ministry of State Planning and Investment, and the Ministry of Trade, Industries and Marketing. During these visits, the consultant examined a wide range of recent documents and studies related to the general economy of Zanzibar and the institutional and policy environment governing livestock and agricultural production, the agricultural and the livestock situation in Zanzibar, and dairy products trade including imports and exports.

2.2.2 Farm level data

The farm level data collection exercise involved questionnaire development, selection of sample farm households and administration of the questionnaire as described in subsequent sections.

2.2.2.1 Development of the questionnaire

The questionnaire used during the 1999 Economic Assessment Survey formed a basis for the development of the questionnaire used in the follow-up survey of 2002. Unlike the 1999 questionnaire, however, this questionnaire had fewer questions designed to facilitate recall of past information. While the 1999 questionnaire was designed to seek information

on events that occurred in 1985/86, this questionnaire was designed to capture recent changes in agriculture and livestock development that have occurred since 1999. This is a shorter time period than the period between 1985 and 1999.

While the 1999 questionnaire contained a total of 66 questions, the questionnaire used in the follow-up survey contained 70 closed and open ended questions to enable respondents report freely and give reasons for certain answers (Annex 2). It covered all the aspects covered during the 1999 survey, namely the general socio-economic characteristics of the respondents; the status and evolution of livestock and agricultural development at the farm level; livestock and crop production systems; land use practices; crop-livestock interactions; the animal disease situation, feeding, breeding and management practices; the post-tsetse eradication livestock and crop productivity parameters; changes in livestock and crop production since 1999, and the future potential of livestock and agricultural development. Because dairy offers more possibilities for increased intensification, both the 1999 and this questionnaire included a section devoted to the dairy enterprise. Unlike the 1999 questionnaire, however, the questionnaire used in the follow-up survey comprises additional sections devoted to marketing and processing of milk at the farm level.

2.2.2.2 Selection of sample farm households

Since the aim of this study was to assess the developments in agriculture and livestock that have occurred since 1999, it was deemed important to follow-up the same farm households sampled and interviewed during the July-August 1999 survey. The list of the farm households was extracted from the 1999 questionnaires at the Department of Agriculture and Livestock headquarters. The availability of the farmers was confirmed during field visits and those who could not be found were replaced by new farmers. Seven hundred sixty two (63%) of the total sample of 1203 farmers interviewed in the follow-up survey were farmers who were also interviewed during the 1999 survey. The remaining 441 (37%) were new farmers.

2.2.2.3 Questionnaire administration

The questionnaire was administered by 16 enumerators and two supervisors (Annex 3). Like the 1999 survey, the enumerators were all employees of the Ministry of Agriculture, Livestock and Natural Resources. Deliberate efforts were made to use those who were enumerators during the 1999 survey. However, only 14 out of the 18 who participated in the 1999 survey were available. Thus, 2 new enumerators were recruited by the consultant with the assistance of the senior staff from the Department of Agriculture and Livestock. The whole team of enumerators was trained for 2 days prior to commencement of field visits. In addition to training, instructions and explanations accompanying the questionnaire were provided to each enumerator to remind them during the fieldwork (Annex 4). During field implementation, the enumerators were organized into four groups and each was assigned a group leader. Interviews began on December 18, 2002 and ended on January 8, 2003. In each village, farmers were informed through the *Shehas* (village leaders) one or two days before the proposed date of interviews. Most interviews took place in the morning period to enable farmers attend to their farm duties. Other interviews took place in the evenings, upon the farmer's return from the farm. On average, each questionnaire was completed within 2 hours. Farmers who had been raising livestock for a

long time took longer to be interviewed as was the case with those who were growing several crops

During the fieldwork, farmers co-operated well and were willing to spend more time with the enumerators. Their hopes and expectations were quite high upon learning of the survey. Unlike the 1999 interviews, which in most cases required farmers to recall information for events that occurred long time ago, very few problems of recalling from memory were encountered during the follow-up survey because the recall period was shorter. Therefore none of the questionnaires was discarded during screening because of lack of responses resulting from failure of the respondents to recall information from memory.

2.2.3 Market level data

One of the objectives of this study was to analyse the current market for dairy products in Unguja and the potential development of a local market for dairy products. To be able to do this, it was essential to collect data from various market participants in addition to the dairy products trade data from secondary sources and milk marketing data collected from the sampled farmers as indicated in section 2.2.2.2. Market participants interviewed include household consumers, owners and managers of hotels and restaurants, and kiosks/milk bar owners and operators. These were considered to be current and potential buyers of locally produced and imported dairy and dairy products.

A total of 30 randomly selected household consumers were interviewed. With regard to hotels/restaurants and kiosks/milk bars, 15 owners/managers of hotels and restaurants and 20 kiosks and milk bars owners/operators were talked to, respectively. The interviews were guided by semi-structured checklists prepared for each category of market participant (Annex 5). The checklist for household consumers contained 7 questions while those for owners/managers of hotels and owners/operators of kiosks and milk bars contained 6 questions each.

The interviews with household consumers were conducted at their homesteads. On the other hand, interviews and discussions with owners/managers of hotels and owners/operators of kiosks and milk bars were carried out at their business premises.

2.3 Data compilation and analysis

Prior to data extraction, questionnaires used to collect data from farmers were coded by administrative district. Access computer software forms were prepared and farmers responses were coded. During data extraction, unanticipated problems that arose from enumerator-introduced errors such as entering the value “zero” where “no response - not applicable” should have been entered were rectified after verification with the enumerators. Problems associated with quantity measurements were resolved by converting them into a single unit. For example, milk reported in 750 cc bottles was converted into liters. Quantities of crops (e.g. cassava, rice, maize, tania, sweet potatoes, cloves) reported in bags, *polos* or *gunias* were multiplied by 50 kg. Quantities reported in *pakachas* (local basket made of palm leaves) were multiplied by 15 kgs while quantities of crops (e.g. tomatoes and other vegetables) reported in *susus* (local container made of palm

leaves) were multiplied by 5 kg. For coconuts, two pieces were assumed to be equivalent to one kg. Crop yields were estimated by converting quantities harvested into kilograms per acre. After screening the data for possible outliers, the data were analysed using the Stata software.

On the other hand, the information from household consumers, hotels and restaurants, and kiosks and milk bars was coded, entered and analysed using the excel computer software.

3.0 RESULTS

This Chapter is organized into four main parts. The first part presents reports results of the analysis of the information obtained from secondary sources and from interviews conducted at the farm and market levels. This part is further divided into five sections. The first section presents the socio-economic characteristics of the sample farm households. The second section describes the current (2002) status of livestock and agriculture development and compares it with the situation in 1999 using the key parameters that show changes in livestock productivity and performance. The third section specifically deals with intensification of livestock and crop production in Unguja, Zanzibar while the fourth section describes the constraints to dairy intensification. The fifth section combines the dairy trade information from secondary sources with results of analysis of information collected from market participants to describe the current market for dairy products on Unguja and the potential development for local market for dairy products. The second part two assesses the socio-economic impact of tsetse and trypanosomosis control/eradication in Unguja, Zanzibar. Implications of the results for agriculture and livestock development in Unguja, Zanzibar are discussed in the third part. The last part presents the summary and conclusions.

3.1 Household socio-economic characteristics

3.1.1 Age and sex distribution

About 94% of the sample households were headed by males while only 6% were headed by females (Table 1). Compared with the results of the 1999 survey, the sample households headed by females have increased by 2%. West District had the largest percentage of female household heads although the percentage is smaller than 1999, when almost 13% of the households in that district were female headed. The average age was 42 years with a maximum of 84 years and a minimum of 15 years.[1] Average age varied from 40 years in North-A and South to 46 years in North-B. Three percent of the households fell within the age category of less than 21 years while 44% fell within the category of 36 to 55 years. About one fifth of the household heads were older than 55 years. These estimates indicate that household heads in Zanzibar are generally of middle age with 79% of them falling within the 21 to 55 age bracket.

[1] The person with 15 years old lost both parents

Table 1: Sex and age distribution of household heads

Sex and age category	District					All districts
	Central	North-A	North-B	South	West	
Sex (%): Male	94.6	98.0	95.0	97.6	89.4	94.3
Female	5.4	2.0	5.0	2.4	10.6	5.7
Total number	501	51	260	165	226	1,203
Average age (Years)	39.7	42.3	45.5	39.7	45.0	42.4
Std. de.	13.6	11.1	15.0	12.3	14.2	13.8
Maximum	95.0	70.0	90.0	78.0	89.0	95.0
Minimum	15.0	17.0	18.0	19.0	18.0	15.0
% within this age group						
Less than 21 years	3.8	2.0	1.9	3.0	0.9	2.7
21 to 35 years	40.7	36.0	27.5	39.0	26.5	34.8
36 to 55 years	42.1	40.0	44.2	45.1	48.2	44.0
Greater than 55 years	13.4	22.0	26.4	12.8	24.3	18.5
Total number	501	51	260	165	226	1,203

Source: Follow-up Socio-economic Survey, 2002.

3.1.2 Education and religious affiliation

About 16% of the sample household heads had no formal education, 7% had acquired some form of adult education. Household heads with primary level education made up 42% while one 36% had secondary level education. These results suggest that in general, the level of education in Zanzibar is low, with about two thirds having at best, primary level education. The household heads are predominantly Muslims (97%) with only 3% being Christians. Those with other religious affiliations are almost negligible.

Table 2: Educational level and religious affiliation of household heads

% households within this level of education	District					All districts
	Central	North-A	North-B	South	West	
No formal education	10.5	21.6	22.4	4.3	18.7	15.5
Adult education	4.1	9.8	4.8	6.2	7.8	6.5
Primary	49.8	41.2	37.6	39.5	40.2	41.7
Secondary	34.6	27.4	34.8	49.4	31.0	35.5
High School	1.0	0.0	0.4	0.6	1.4	0.7
University	0.0	0.0	0.0	0.0	0.9	0.2
Total number	501	51	260	165	226	1,203
Religious affiliation (%)						
Muslim	96.8	100.0	98.1	100.0	88.1	96.6
Christian	3.2	0.0	1.9	0.0	11.5	3.3
Others	0.0	0.0	0.0	0.0	0.5	0.1
Total number	501	51	260	165	226	1,203

Source: Follow-up Socio-economic Survey, 2002.

3.1.3 Household size and structure

Table 3 shows the household structure by age and sex category. The average household size was 6.9 persons with a maximum of 36 persons and a minimum of one person. North-A District had the largest average household size of 8.2 persons while Central and South Districts had the smallest size of 6.2 persons each. Each household had an average of 2

adults and 4 children. These results suggest that the average household size obtained in this study is lower than that obtained in 1999.

Table 3: Household size and structure by age and sex category

Household Size (Number)	District					All districts
	Central	North-A	North-B	South	West	
Average	6.2	8.2	7.1	6.2	6.7	6.9
Std.dev.	3.3	3.4	3.9	3.0	3.5	3.4
Max.	21.0	17.0	36.0	16.0	19.0	21.8
Min.	1.0	1.0	1.0	1.0	1.0	1.0
Less than 12 years	2.4	2.4	2.8	2.3	2.6	2.5
12 to 18 years	1.4	2.2	1.7	1.5	1.6	1.7
Adult Males	1.2	1.3	1.4	1.2	1.2	1.3
Adult Females	1.2	1.3	1.3	1.2	1.2	1.3

Source: Follow-up Socio-economic Survey, 2002.

3.1.4 Occupational distribution

Farming is the major occupation of the people of Unguja Island. About 89% of the sample household heads reported farming as their major occupation (Table 4). By definition, farming includes livestock keeping and crop agriculture. Wage employment in the public service was an occupation for only 8% of the household heads with 3% of them involved in business and other income earning activities. Among the secondary occupations, farming was reported by about 41% of the household heads followed by 28% in business and 12% in the public service. Other secondary occupations such as fishing were reported by only 1% of the household heads.

Table 4: Distribution of household heads by occupation

Major occupation: (%)	District					All districts
	Central	North-A	North-B	South	West	
Farming	91.7	86.0	89.6	85.8	89.7	88.6
Business	2.4	10.0	1.2	1.2	1.3	3.2
Public service	5.9	4.0	9.2	13.0	8.9	8.2
Other	0.0	0.0	0.0	0.0	0.0	0.0
Total number	501	51	260	165	226	1,203
Secondary occupation: (%)						
Farming	42.7	47.1	43.3	33.3	37.3	40.7
Business	37.1	47.1	15.6	10.6	31.3	28.3
Public service	7.7	5.9	7.8	19.7	20.9	12.4
Other	0.0	0.0	0.0	6.7	0.0	1.3
Total number	501	51	260	165	226	1,203

Source: Follow-up Socio-economic Survey, 2002.

3.1.5 Household income

Household income averaged TShs 53, 502 (US$ 53.5) per month with a maximum monthly income of TShs 930,000 (US$ 930) and a minimum of TShs 4,500 (US$ 4.5) (Table 5). Income is highest in West District despite the widest variation in income distribution. In 1999, the average household income was estimated at TShs. 41, 232 (US$

51.5) with a maximum monthly income of TShs. 850,000 (US$ 1,063) and a minimum of only TShs. 2,000 (US$ 2.5) (Tambi *et al*., 1999). Overall the income per household in Tanzanian Shillings has increased since 1999 although the increase is not significant in dollar value due to depreciation of the Tanzanian Shilling. Approximately 93% the farm households reported an average monthly income of less than TShs 100,000 (US$ 100). In the 1999 survey, 97% of the farm households reported an average monthly income of less than TShs. 100,000 (US$ 100) (Tambi *et al*., 1999). Although the percentage of households with monthly income below TShs. 100,000 has declined during the past three years, the fact that more than 90% of households still reported incomes below TShs. 100,000 is a reflection of the generally low income level of farm households in Zanzibar.

Table 5: Average household income and frequency distribution of household income

	District					
Household income (TSHS/Month)	Central	North-A	North-B	South	West	All districts
Average	48,384<br>(48.4)	50,373<br>(50.4)	55,298<br>(55.3)	41,909<br>(41.9)	71,545<br>(71.5)	53,502<br>(53.5)
Standard deviation	29,322<br>(29.3)	24,226<br>(24.2)	34,856<br>(34.8)	23,956<br>(23.9)	75,748<br>(75.7)	37,622<br>(37.6)
Maximum	300,000<br>(300.0)	100,000<br>(100.0)	300,000<br>(300.0)	200,000<br>(200.0)	930,000<br>(930.0)	930,000<br>(930.0)
Minimum	5,000<br>(5.0)	10,000<br>(10.0)	5,000<br>(5.0)	4,500<br>(4.5)	5,000<br>(5.0)	4,500<br>(4.5)
% within this income group:						
Less than 25,000 TSHS (31.3)	13.0	9.8	8.8	14.5	12.8	12.1
25,000 to 50,000 TSHS<br>(31.3) to (62.5)	54.1	52.9	45.0	61.8	35.4	49.6
50,001 to 100,000 TSHS<br>(62.5) to (125)	27.9	37.3	40.0	19.4	34.5	31.0
100,001 to 200,000 TSHS<br>(125) to (250)	3.0	0.0	3.5	1.2	14.2	4.8
Greater than 200,000 TSHS<br>(250)	02	0.0	0.8	0.0	229	0.7
Total number	501	51	260	165	226	1,203

Remarks: Numbers in parentheses are values in US$ (1 US$ = 1000 TShs)
Source: Follow-up Socio-economic Survey, 2002.

From the preceding estimates, farm households in Unguja can be characterized as being predominantly headed by middle-age Muslim males with less than secondary level education. The households are relatively large with more children than adults and derive a very meagre income from farming activities. These characteristics have important implications for livestock and agriculture development on the Island, particularly for the adoption of production systems that require human, financial and other physical resources necessary for increasing productivity.

3.2 The Status of Livestock and Crop production in Unguja and changes that have occurred since 1999

This section examines the current (2002) status of livestock and agriculture development in Unguja taking into account the key parameters that show changes in livestock productivity and performance since the 1999 Economic Assessment Survey.

3.2.1 Livestock production

3.2.1.1 Livestock species raised

Currently, livestock producers in Zanzibar raise cattle, goats, sheep, chickens, ducks, Guinea fowls, donkeys, rabbits and pigs. As was the case in 1999 and before tsetse intervention in 1985/86, cattle, goats and chickens are the most important species in terms of numbers and the proportion of farms raising them.

3.2.1.1.1 Cattle

About 94% of the sample farmers reported raising indigenous cattle, with proportions varying from 92% in South District to 98 % in North-A Districts (Table 6). The results indicate substantial increase in the percentage of farm households raising indigenous livestock during the past three years especially in North-A and North-B Districts. In the 1999 survey, 77% of the sample farmers reported raising indigenous cattle with proportions varying from 20% in North-A to 95% in Central and South Districts (Tambi *et al.*, 1999). Farms with improved cattle, mainly crossbred cattle make up 24% of the sample, with proportions ranging from 12% in North-A District to 35% in West District. In 1999 the proportion of farms raising improved cattle was estimated at about 18%. When the 1991 situation is compared to the current (2002) estimate, it can be said that more farmers have become involved in improved cattle production (intensive cattle production) as well as indigenous cattle production. However, the increase in the number of farms raising indigenous cattle outweigh the increase in the number of farms raising improved cattle.

The average number of indigenous cattle per farm is estimated at 5.49 (± 4.59 sd) indigenous cattle (Table 7). Farms in Central District have more indigenous cattle (6.7 cattle per farm) compared to farms in West District (4.2 cattle per farm) even though the proportion of farms with indigenous cattle is greater in the West than in the Central. The current number of indigenous cattle per farm is approximately more than twice the number in 1999. In 1999, the average number of indigenous cattle per farm was estimated at 2.28 (± 4.36 sd) with farms in Noth-B District having more indigenous cattle (2.6 cattle per farm) compared to farms in South District (1.9 cattle per farm) (Tambi *et al.*, 1999). Regarding improved cattle, the average number per farm household is estimated at 0.41 (± 1.12 sd) crossbred cattle (Table 7). On average, farms in West and Central Districts have significantly more improved cattle than farm households in the remaining districts with North-A District raising the least number of improve cattle per farm household (Table 8). While the average number of indigenous cattle per farm household has increased significantly during the last three years (1999 to 2002), the average has declined slightly from an average of 0.59 cattle per farm household in 1999 to 0.41 cattle per farm household in 2002.

Table 6: Proportion (%) of farms with livestock in Unguja, 2002

Livestock species:	Central (n=392)	North-A (n=65)	North-B (n=255)	South (n=186)	West (n=240)	All Districts (n=1,138)
Indigenous cattle	90.4	98.0	95.8	92.1	92.5	93.8
Improved cattle	40.3	11.8	15.8	14.5	35.0	23.5
Sheep and goats	17.8	7.8	16.5	26.1	21.7	18.0
Local poultry	63.9	74.5	71.5	50.9	77.4	67.7
Improved poultry	5.2	5.9	4.2	1.8	4.4	4.3
Donkeys	1.0	5.9	3.5	0.0	3.1	2.7

Source: Follow-up Socio-economic Survey, 2002.

3.2.1.1.2 Sheep and goats

The proportion of farms with sheep and goats is estimated at 18% (Table 6), which is higher than 13% estimated during the 1999 socio-economic survey (Tambi *et al.*, 1999). In general farms raising sheep and goats have increased during the past three years. Like in 1999, most of the farm households with goats are in West (22%), Central (18%) and South (26%) Districts (Table 6). The proportions of farm households raising sheep and goats in 19999 were West (20%), Central (14%) and South (13%) (Tambi *et al.*, 1999). The average number of sheep and goats per farm household is 0.5 (±2.47sd) with farm households in North-A District having fewer numbers (0.14) than West District (0.71) (Table 7). Farms in the North-A District had also the least proportion of farm households raising sheep and goats (Table 6).

3.2.1.1.3 Chickens

About 68% of the sample farmers raise indigenous chickens while 4% raise improved chickens (Table 6). The proportions of farm households raising local chicken and improved chickens are higher than the proportions of farm households raising the same three years ago (1999). In 1999, 48% and 2% of farm households were raising local and improved chicken, respectively (Tambi *et al.*, 1999). Currently, each farm has an average of 11.5 (± 16.1 sd) and 6.1(± 45.4 sd) indigenous and improved chickens respectively (Table 7) compared with 1999 when each farm had an average of 9 ((± 12 sd) and 7 (± 198 sd) indigenous and improved chickens respectively (Tambi *et al.*, 1999). The results generally indicate that while the proportion of farms raising improved chickens has increased, the number of improved chickens per farm household has declined slightly during the past three years.

3.2.1.1.4 Donkeys

Approximately 3% of the sample farm households are raising donkeys with each household raising an average of 0.1 ((± 0.6) donkeys (Table 6). The proportion of farm households raising donkeys is higher by 1% compared with the estimated proportion of 2% of farm households raising the same livestock species in 1999 (Tambi *et al.*, 1999).

Table 7: Average herd /flock sizes for cattle, chickens, sheep and goats in Unguja, 2002

	Average number of animals per farm				
District	Indigenous cattle	Crossbred cattle	Goats and sheep	Indigenous chickens	Improved chickens
Central	6.70 (6.83)*	0.80 (1.84)	0.58 (4.08)	12.28 (25.96)	7.77 (60.88)
North-A	6.33 (5.75)	0.08 (0.23)	0.14 (0.49)	11.37 (12.46)	0.65 (3.12)
North-B	5.32 (3.81)	0.16 (0.55)	0.43 (1.88)	12.53 (15.08)	7.24 (80.28)
South	4.92 (3.46)	0.15 (0.50)	0.62 (2.27)	8.18 (11.20)	0.68 (7.82)
West	4.20 (3.12)	0.85 (2.45)	0.71 (3.61)	13.15 (15.73)	13.89 (74.69)
Average	5.49 (4.59)	0.41 (1.12)	0.50 (2.47)	11.50 (16.09)	6.05 (45.36)

Source: Follow-up Socio-economic Survey, 2002.
* Standard deviations in parentheses.

3.2.1.2 Dairy production

As indicated in section 3.2.1.1.1 above, 94% of the sample farmers reported that they were raising indigenous cattle and only 24% were raising improved cattle, mainly crossbred cattle. This clearly shows that the dairy sub-sector in Unguja is still largely based on indigenous cattle.

3.2.1.2.1 Milk production with indigenous cattle

The results of this survey indicate that 74% of the sample farms keeping indigenous cattle milked their cattle in 2002. On average each farm produced about 3.4 litres with a minimum of 0.5 litres and a maximum of 55 litres per farm per day (Table 8). Production per cow per day averaged 2.5 litres. According to the 1999 socio-economic survey, 58% of the farming households studied were milking indigenous cattle and produced an average of 2.4 liters/cow/day with a standard deviation of 2.5 litres (Tambi *et al*., 1999). Comparison of the current estimates with the 1999 estimates indicates that some changes have occurred in the traditional dairy sub-sector in Unguja. First, the relative proportion of farms milking indigenous cattle has increased from 58% in 1999 to 74% in 2002, suggesting that more farmers have increasingly become involved in traditional dairy production during the past three years. Second, while the proportion of farms milking indigenous cattle has increased significantly, average milk production has increased slightly from 2.4 litres/cow/day in 1999 to 2.5 litres/cow/day.

Table 8. Milk production within the traditional sector in Unguja, Zanzibar, 2002

District	% of farms producing milk	Quantity of milk produced per farm per day (litres/farm/day)		
		Average	Minimum	Maximum
Central	59.9	3.5(4.6)	0.5	35.0
North-A	88.2	3.8 (4.1)	0.5	17.0
North-B	82.7	3.7 (5.6)	0.5	55.0
South	79.4	1.7 (2.5)	0.5	12.0
West	66.4	4.3 (4.5)	0.5	27.0
All districts	74.3	3.4 (4.3)	0.5	55.0

Source: Follow-up Socio-economic Survey, 2002.
* Standard deviations in parentheses

3.2.1.2.2 Milk production with improved cattle

As pointed out in section 3.2.1.1.1, 24% of the sample farms (289 farms) raised improved cattle in 2002. Most (93%) of the farms raising improved cattle reported that they were milking their cattle. The average milk production per farm was 10.6 litres/day with a minimum of 1 litre per farm per day and maximum production of 74 litres per farm per day (Table 9). On average, farms raising improved cattle were producing significantly more milk per day than farms raising indigenous cattle (Tables 8 and 9).

Table 9: Milk production within the improved dairy sector in Unguja, Zanzibar, 2002

District	Quantity of Milk Produced per farm (Liters/farm)		
	Average	Minimum	Maximum
Central	12.0 (10.6)	2.0	74.0
North-A	10.0 (3.3)	3.0	10.0
North-B	10.8 (5.2)	5.0	24.0
South	3.6 (3.1)	1.0	10.0
West	16.6 (12.7)	4.0	50.0
All districts	10.6 (7.6)	1.0	74.0

Source: Follow-up Socio-economic Survey, 2002.
* Standard deviations in parentheses

3.2.2 Crop production

3.2.2.1 Types of crops produced and their importance

As pointed out earlier, agriculture is the dominant sector of the economy of Zanzibar. Besides livestock, farmers in Unguja, Zanzibar grow a wide range of crops for food and income generation. These crops include cloves, coconuts, cassava, sweet potatoes, bananas, yams, rice, maize, vegetables, beans, peas, pineapples, oranges, tomatoes and eggplant. When asked to rank the major food crops in their declining order of importance, most farmers interviewed reported cassava, banana/plantain, sweet potatoes, maize, vegetables, yams and rice as important food crops (in declining order of importance) (Table 10). Slight differences exist across the five districts in the ranking of the major food crops. While cassava and banana/plantain were reported by most farmers interviewed in all five districts as the first and second important food crops respectively, there are differences between districts in the third, fourth, fifth, sixth and seventh important food crops. For example, most farmers in Central, North-A and North-B Districts ranked potatoes as the third important food crop. On the other hand, farmers in South District and West District reported vegetables and maize as the third important food crop, respectively.

Important cash crops (in declining order of importance) as considered by most of the interviewed farmers are cassava, banana/plantain, sweet potatoes, coconut, vegetables, fruits, yams, rice and cloves (Table 11). Like the ranking of major food crops, there are slight differences between districts in the order of importance for the third up to the eighth important cash crop (Table 11). For example, most farmers in Central, South and West Districts reported sweet potatoes as the third important cash crop. In North-A and North-B, most farmers reported coconuts and vegetables as the third important cash crop, respectively (Table 11). Almost all the food crops considered as important by the farmers

are important sources of cash income. Cassava and banana/plantain appear to be the most important crops not only as food crops but also as the major cash crops in the household economy of Unguja, Zanzibar.

When compared with the situation in 1999, slight changes have occurred in the relative importance of both the major food and cash crops in the economy of Zanzibar. According to the 1999 socio-economic survey, rice was reported as the fourth important food crop and maize was reported as the sixth important food crop. According to the results of the follow-up survey, maize was reported as the fourth important food crop while rice was reported as the seventh important food crop (Table 11). Regarding cash crops, the relative importance of most crops as important sources of cash income has changed during the past three years. For example vegetables which ranked fifth as an important source of cash income in 1999 (Tambi et al., 1999) is currently considered as the second major source of cash income (Table 11). According to the 1999 socio-economic survey, cloves which is traditionally the major export and cash crop of Zanzibar was ranked below cassava, banana, sweet potatoes, coconut, vegetables and yams but was considered to be more important than maize, fruits and rice (Tambi, *et al*., 1999). The relative importance of this crop as a major cash crop appears to have declined further compared with the situation in 1999. The results in Table 11 indicate that cloves is now ranked below fruits and rice as an important sources of cash income for the interviewed farm households.

Table 10: Food crops considered as important by farmers

Farmers who consider these as important (%)	District					All districts (n=1,203)
	Central (n=501)	North-A (n=51)	North-B (n=260)	South (n=165)	West (n=226)	
Cassava	44.6	38.2	46.3	42.1	44.1	43.1
Banana/plantain	41.0	28.7	35.4	30.7	40.5	35.2
Sweet potatoes	15.4	23.5	20.4	10.1	15.8	17.0
Maize	7.1	22.1	17.4	1.3	21.0	13.8
Vegetables	11.3	6.6	5.7	19.0	7.5	10.0
Yams	12.5	1.5	4.7	23.3	5.1	9.4
Rice	5.7	10.3	13.3	2.9	9.0	8.2
Other food crops	10.3	7.9	4.3	13.2	4.9	7.7

Source: Follow-up Socio-economic Survey, 2002.

Table 11: Cash crops considered as important by farmers

Farmers who consider these as important (%)	District					All districts (n=1,203)
	Central (n=501)	North-A (n=51)	North-B (n=260)	South (n=165)	West (n=226)	
Cassava	13.0	19.8	21.8	19.8	20.1	18.9
Vegetables	16.8	15.2	18.2	18.8	13.1	16.4
Banana/plantain	15.7	18.6	15.7	14.1	13.5	15.5
Fruits	13.4	12.7	9.6	11.0	12.7	11.9
Yams	8.2	9.3	12.9	6.3	10.8	9.5
Sweet potatoes	10.7	3.5	6.8	13.5	13.1	9.5
Coconuts	8.2	10.5	8.9	7.3	10.8	9.1
Rice	7.5	3.5	2.5	3.6	3.1	4.0
Cloves	3.4	4.7	2.5	2.1	2.7	3.1
Maize	1.8	2.3	1.1	3.6	0.0	1.8

Source: Follow-up Socio-economic Survey, 2002.

3.2.2.2 Acreage cultivated

Average land area cultivated in 2002 varied from 0.9 acres for vegetables to 2.0 acres for coconuts with the West District having the largest amount of land (2.8 acres) devoted to coconuts (Table 12). When compared with the situation before tsetse intervention in 1985/86 and after tsetse intervention in 1999 (Table 13) the average land areas cultivated do not vary significantly for most crops. However, relatively more land in 2002 is allocated to the crops given top priority as cash crops and food crops. Comparing the areas cultivated in 1999 and 2002, land areas cultivated for coconut, cloves, rice and yams declined with area under cloves declining more than the other crops (Table 13). Land areas cultivated for cassava, sweet potatoes, tania (cocoyams) and maize increased slightly or remained the same while land areas cultivated for vegetables and plantain/banana increased substantially (Table 13).

Table 12: Land area (acres) cultivated for important food and cash crops after tsetse intervention in 1999 in Unguja, Zanzibar

Type of crop	District					All districts
	Central	North-A	North-B	South	West	
Coconut	1.6	1.5	2.5	1.7	2.8	2.0
Cassava	1.5	1.6	1.8	1.4	1.5	1.6
Banana	1.3	1.0	0.8	1.3	1.0	1.1
Sweet potatoes	1.2	1.6	1.7	0.9	1.0	1.3
Yams	1.5	0.7	1.1	1.7	1.3	1.3
Cloves	1.4	1.2	2.3	n.a	1.8	1.7
Rice	1.5	1.0	1.3	1.0	1.2	1.2
Maize	1.2	n.a	0.5	1.1	1.2	1.0
Vegetables	1.3	0.6	0.8	1.0	0.7	0.9

Source: Follow-up Socio-economic Survey, 2002.

Although the increase in land areas cultivated for some of the crops may be attributed to factors other than influence of tsetse eradication, tsetse eradication appears to have increased access to land for crop production. A large proportion (70%) of the sample farmers who acquired new land after tsetse eradication did so in tsetse cleared areas. The Central district has the largest proportion of farmers who grow crops on land acquired in tsetse cleared areas. Moreover, some farmers (46% of total sample) intend to acquire new crop land in tsetse cleared areas in future. Again, most of the farmers who intend to acquire crop land in tsetse cleared areas are in the Central District.

Table 13: Changes in average land areas cultivated before and after tsetse eradication (1999 and 2002) in Unguja, Zanzibar

Type of crop	Acreage cultivated (acres)			% Change from 1999 to 2002
	Before tsetse intervention in 1985/86[a)]	After tsetse eradication		
		1999[a)]	2002[b)]	
Coconut	2.0	1.9	2.0	5
Cassava	1.4	1.4	1.6	14
Banana/plantain	1.3	2.1	2.2	5
Sweet potatoes	1.0	1.1	1.3	18
Yams/cocoyams	1.8	1.8	1.7	-5.5
Cloves	1.3	1.0	1.7	70
Rice	1.1	1.0	1.2	20
Maize	0.9	0.9	1.0	11
Vegetables	0.5	0.7	0.9	29

Source:
[a)]Tambi et al. (1999)
[b)] Follow-up Socio-economic Survey, 2002.

3.2.2.3 Quantities of crops harvested and crop yields

Tables 14 and 15 respectively provide information on quantities of crops harvested and crop yields obtained in 2002 (5 years after tsetse eradication). Except for cloves, quantities harvested in 2002 of the other crops increased from their 1999 levels (Table 14). In terms of yields, 2002 yields for cassava, rice, maize, coconut and vegetables increased from their 1999 levels while yields for bananas, sweet potatoes, yams and cloves declined from their 1999 levels (Table 15).

Table 14: Quantities of food and cash crops harvested from cultivated areas after tsetse eradication in 2002 in Unguja, Zanzibar

Type of crop	District					All Districts	
	Central	North-A	North-B	South	West	Average 2002 survey (n=1203)	Average 1999 survey (n=1,138)
Cassava	756	1,062	1,171	605	1,113	949	790
Rice	659	451	574	579	580	569	443
Maize	786	n.a	490	300	820	599	392
Banana/plantain*	63	42	43	56	49	54	52
Sweet potatoes	744	1,462	1,412	301	850	954	837
Yams	1,099	630	1,123	681	751	857	471
Cloves	120	180	265	n.a	132	174	249
Coconut**	1,956	805	1,319	3,213	744	1,608	1,124
Vegetables	646	210	440	633	135	413	197

*Banana bunches (*Mikungu*)
** Coconut fruits *(nazi)*
Source: Follow-up Socio-economic Survey, 2002.

Table 15: Food and cash crop yields obtained after tsetse intervention in Unguja, Zanzibar (Kg/acre), 2002

Type of crop	District					All Districts	
	Central	North-A	North-B	South	West	Average	% of farmers responding (n=1,138)
Cassava	504	664	651	432	742	599	559
Rice	439	451	442	579	483	474	443
Maize	655	n.a	980	273	683	599	456
Banana*	49	42	54	43	49	49	53
Sweet potatoes	620	914	831	334	850	733	756
Yams	733	900	1,121	401	578	659	687
Cloves	86	150	201	n.a	73	102	252
Coconut	1,223	537	528	1,889	266	804	608
Vegetables	496	350	550	633	193	456	285

* Bunches

Source: Follow-up Socio-economic Survey, 2002.

3.3 Intensification of livestock and crop production

Indicators of intensification in livestock production include adoption of high yielding animal breeds accompanied by improved nutritional and health management practices. On the other hand, intensification in crop production requires the adoption of high yielding crop varieties, use of inorganic and organic fertilizers and other inputs that can raise output per unit area. How well farmers adopt these practices clearly depends on the availability of opportunities and their willingness to take advantage of such opportunities. The eradication of trypanosomosis in 1997 has provided a more favorable environment for raising improved livestock species and has opened up new opportunities for a more optimal use of land resources through integration of crop and animal agriculture as well as the availability of improved animal breeds and crop varieties. The 1999 socio-economic survey (Tambi *et al.*, 1999) has provided evidence for increasing intensification of livestock and crop production following tsetse eradication in Unguja, Zanzibar. The study has shown increasing trends in the use of animal manure for crop production, use of crop by-products as livestock feed and use of animal power for ploughing and transportation after tsetse eradication in 1999. However, the extent however, to which farmers will continue to intensify their production after tsetse eradication depends on their resource endowment, availability and increasing level of awareness of different opportunities.

3.3.1 Availability and farmers preference for improved livestock breeds

When farmers were asked about their views on the availability of improved livestock breeds now when compared with 1999, 62% of the sample farmers reported that exotic cattle breeds are readily available now than three years ago (1999) whereas 40% indicated that cross bred cattle are readily available now than 1999. About 61% reported that improved poultry breeds are readily available now than 1999 (Table 16). When information was sought on farmers' preferences for livestock breeds, 61% reported that they preferred improved breeds while 38% preferred local breeds (Table 17). The major reason given for preferring improved breeds were their high level of productivity (94%). However, the remaining 6% preferred them because of their ability to mature early. Also

two major reasons were given for preference of local breeds. These are the fact that they are easy to manage (55 %) and they are disease resistant (45%).

Table 16: Farmers views on the availability of improved livestock species in Unguja, Zanzibar

Farmers views on relative availability of improved livestock species now and 1999:	District					All districts (n=1,203)
	Central (n=501)	North-A (n=51)	North-B (n=260)	South (n=165)	West (n=226)	
Pure-bred cattle:						
More available now	64.3	60.0	69.6	43.0	10.6	62.2
Same as 1999	7.9	20.0	11.8	11.2	70.2	11.6
Less available now	27.8	20.0	18.6	45.8	19.2	26.2
Crossbred cattle:						
More available now	32.8	63.6	40.5	52.6	30.0	40.0
Same as 1999	46.6	18.2	47.6	15.8	10.0	39.7
Less available now	20.6	18.2	11.9	31.6	60.0	20.3
Improved sheep and goats:						
More available now	32.8	63.6	40.5	52.6	10.6	40.0
Same as 1999	46.6	18.2	47.6	15.8	70.2	39.7
Less available now	20.6	18.2	11.9	31.6	19.2	20.3
Improved poultry:						
More available now	80.4	0.0	72.4	71.1	80.3	60.8
Same as 1999	10.1	90.5	10.6	7.8	10.9	26.0
Less available now	9.5	9.5	17.0	21.1	8.8	13.2

Source: Follow-up Socio-economic Survey, 2002.

Table 17: Farmers' preference for improved livestock breeds and reasons

Farmers who prefer:	District					
	Central (n=501)	North-A (n=51)	North-B (n=260)	South (n=165)	West (n=226)	All districts (n=1,203)
Local breeds: Number	167	1	115	115	59	457
%	33.3	2.0	44.2	69.7	26.1	38.0
Improved breeds: Number	329	48	144	50	167	738
%	65.7	94.1	55.4	30.3	73.9	61.3
Non-responses (%)	1.0	3.9	0.4	0.0	0.0	1.1
Reasons for preference (%):						
Local breeds:						
Disease resistance	37.27	70.83	42.48	40.00	37.71	45.3
Easy to manage	62.73	29.17	57.52	60.00	64.29	54.7
Improved breeds:						
High productivity	100.0	87.69	83.58	97.37	99.23	93.6
Early maturity	0.0	12.31	16.42	2.63	0.77	6.4

Source: Follow-up Socio-economic Survey, 2002.

3.3.2 Adoption of improved livestock breeds and crop varieties

Although the level of adoption of improved livestock breeds and crop varieties in Unguja still remains low, access to improved livestock breeds and crop varieties has improved slightly over the past three years. In 1999, only 4% and 2% of the farmers acquire crossbred cattle and exotic cattle respectively from Government farms (Tambi *et al.*, 1999). In 2002, 26% and 11% of the sample farmers acquired crossbred and exotic cattle

respectively from Government farms (Table 18). Sources of improved livestock breeds for some livestock species like poultry have also increased. There is now a private wholesaler of improved poultry chicks in Zanzibar town. This source was not reported by any of the farmers interviewed during the 1999 survey (Tambi *et al*., 1999). The farmers interviewed mentioned two factors that limit adoption of improved cattle breeds in Unguja. These are inadequate finances and unavailability of the breeds. Like three years ago inadequate finances (purchasing power) is currently the most important limiting factor to increased adoption of improved livestock breeds according to 52% of the sample farmers (Table 19). In 1999, 46% of the farmers indicated inadequate finances as the major limiting factor to increased adoption of improved cattle breeds.

Table18: Sources of improved livestock species in Unguja, Zanzibar

% of farmers who acquire improved livestock from this source:	District					All districts (n=1,203)
	Central (n=501)	North-A (n=51)	North-B (n=260)	South (n=165)	West (n=226)	
Exotic cattle:						
Neighbors and other farmers	4.8	0.0	3.5	4.8	11.9	5.0
Government farm	11.2	0.0	19.2	9.7	14.2	10.9
Livestock market	0.6	0.0	1.2	0.0	0.9	0.5
Crossbreed cattle:						
Neighbors/other farmers	31.7	7.8	18.1	12.7	31.4	20.4
Gov't farm	16.0	27.5	28.1	29.7	30.5	26.3
Livestock market	16.6	0.0	16.5	3.0	22.6	11.7
Improved sheep and goats:						
Neighbors/other farmers	3.4	39.2	1.2	13.9	0.4	11.6
Gov't farm	8.4	27.5	6.9	22.4	2.2	13.5
AI Centre	0.0	0.0	1.5	1.2	0.0	0.6
Livestock market	0.8	3.9	0.4	0.6	0.4	1.2
Improved poultry:						
Neighbors and other farmers	1.4	7.8	2.7	4.2	11.1	5.4
Government farm	1.0	3.9	10.4	0.6	15.5	6.3
ZAPOCO	22.0	103.9	6.9	26.7	99.6	51.8
Wholesaler in Zanzibar town	23.8	133.3	7.7	46.7	125.7	67.4

Source: Follow-up Socio-economic Survey, 2002.

Table 19: Important constraints to the acquisition of improved livestock breeds in Unguja, Zanzibar

% Farmers who consider this as a constraint	District					Total (n=1,203)
	Central (n=501)	North-A (n=51)	North-B (n=260)	South (n=165)	West (n=226)	
Inadequate finances	41.3	79.7	65.1	31.4	43.7	52.2
Unavailability	1.6	2.0	2.8	1.0	1.8	1.8

Source: Follow-up Socio-economic Survey, 2002.

For those farmers who are able to acquire improved livestock breeds, the average price paid for crossbred cattle is Tshs 254,000 (US$ 254) (±89,000 Tshs) (± 89 US$) with a minimum price of Tshs 100,000 (US$ 100) and a maximum of Tshs 700,000 (700 US$). For exotic cattle breeds the average price is Tshs 269,000 (269 US$) (± Tshs 100,000) (± 100 US$) (Table 20). The average price for improved poultry is Tshs 552 (US$ 0.55) (±

Tshs 242 (±0.24 US$). The prices for cross bred cattle are higher than the prices reported by farmers in 1999. However, the prices of exotic cattle are lower than the prices reported in 1999. In 1999, the average price paid for crossbred cattle was Tshs 182,000 (US$ 228) (± 90,000 Tshs) (± 112.5 US$). For exotic cattle breeds and improved poultry the average prices were Tshs 287,000 (359 US$) (± Tshs 95,000) (± 119 US$) and Tshs 528 (US$ 0.7) (± Tshs 304) (±0.4 US$) respectively (Tambi *et al.*, 1999).

Table 20: Average prices paid by farmers for improved livestock breeds in Unguja, Zanzibar, 2002

	District					All districts	
	Central	North-A	North-B	South	West	Amount	%
Pure-bred cattle:							
Average price	242	350	256	178	321	269	
(1,000 Tshs/head)	(242)	(350)	(256)	(178)	(321)	(269)	
Standard deviation	106	118	96	77	102	100	
	(106)	(118)	(96)	(77)	(102)	(100)	
Maximum	500	500	400	300	500	500	
	(500)	(500)	(400)	(300)	(500)	(500)	
Minimum	100	150	100	100	100	100	
	(100)	(150)	(100)	(100)	(100)	(100)	
Number of farmers	31	6	35	8	42	122	10.14%
Crossbreed cattle:							
Average price	251	276	243	106	181	254	
(1,000 Tshs/head)	(251)	(276)	(243)	(106)	(181)	(254)	
Standard deviation	75	92	85	118	78	89	
	(75)	(92)	(85)	(118)	(78)	(89)	
Maximum	500	450	500	700	500	700	
	(500)	(450)	(500)	(700)	(500)	(700)	
Minimum	100	120	100	100	100	100	
	(100)	(120)	(100)	(100)	(100)	(100)	
Number of farmers	172	26	69	30	80	377	31.34%
Improved poultry:							
Average price	565	495	592	622	487	552	
	(0.57)	(0.5)	(0.59)	(0.62)	(0.49)	(0.55)	
Standard deviation	228	125	290	348	219	242	
	(0.23)	(0.13)	(0.29)	(0.35)	(0.22)	(0.24)	
Maximum	1,000	600	2500	2,000	1000	2,500	
	(1.0)	(0.6)	(2.5)	(2.0)	(1.0)	(2.5)	
Minimum	15	100	75	100	42	15	
	(0.02)	(0.10)	(0.08)	(0.1)	(0.04)	(0.02)	
Number of farmers	167	21	100	38	81	407	33.83%

Remarks. Numbers in parentheses are values in US$ (1 US$ = 1000 TSHS)
Source: Follow-up Socio-economic Survey, 2002.

3.3.3 Access to land resources

Following eradication of tsetse flies in Unguja, more land became available for crop and livestock production, particularly in the previously infested forest areas of the Island except protected areas like the Jozani forest. According to the 1999 socio-economic survey (Tambi *et al.*, 1999), 23% of the farmers interviewed during the survey reported that they had acquired new farm land in areas cleared of tsetse flies after 1997 and for those farmers who did not have land in tsetse cleared areas, 46% had plans to do so in the future. During

the follow-up survey, farmers were asked whether they continued to expand cultivated land after 1999. As indicated by the survey results in Table 21, 35% of the farmers reported that they have increased land area cultivated during the last three years, 52% cultivated almost the same land area as 1999 and 7% decreased the land area cultivated. Reasons given by those who decreased their cultivated land area include labour shortage (37%), inadequate capital (20%), old age (12%), low productivity of land (3%) and low prices for crop products (2%). For those who expanded cultivated land during the last three years, 79% have acquired the land from areas cleared of tsetse flies.

Table 21: Change in land area cultivated during the last three years (1999-2002)

Farmers who cultivated land has:	District					
	Central (n=501)	North-A (n=51)	North-B (n=260)	South (n=165)	West (n=226)	All district (1,203)
Increased (%)	35.2	29.4	38.1	40.6	33.6	35.4
Remained the same (%)	53.4	58.8	47.3	44.2	57.5	52.3
Decreased (%)	5.0	7.8	10.0	4.8	6.6	6.9
No-responses (%)	6.4	4.0	4.6	10.4	2.3	5.4

Source: Follow-up Socio-economic Survey, 2002.

3.3.4 Use and access to improved seeds

There is limited use of improved seeds for crop production in Unguja, Zanzibar. Most of the farmers interviewed used local seed varieties during the 2002 cropping season. Improved seeds were used for maize, cassava, rice, vegetables, fruits and coconuts. Rice was the crop with the largest percentage of farmers using improved seeds and North-A dominates in the use of improved rice seeds (Table 22). When asked about availability of improved seeds, only 38% of the farmers reported that improved seeds were readily available. Improved seeds are reported to be more readily available in North-B District and less available in Central District (Table 23). Most (96%) of those using improved seeds reported that they acquired them from Government Agricultural Centres (*Kilimo*). Other sources include private inputs shops (2%), individual extension officers (1%) and other farmers (1%). The average prices reported by those who purchased improved seeds are TShs 308 per kg for maize, TShs. 222 per kg. for rice, TShs. 52 per bundle for cassava and TShs. 163 per seedling for coconuts (Table 24)

Table 22: Use of Improved seeds in Unguja in 2002

Farmers who used improved (%):	District					All district (n=1,203)
	Central (n=501)	North-A (n=51)	North-B (n=260)	South (n=165)	West (n=226)	
Maize	5.6	0.0	6.1	17.4	2.0	6.2
Rice	43.0	58.3	54.5	41.3	56.0	50.6
Cassava	5.6	0.0	6.1	4.3	6.0	4.4
Coconuts	1.9	8.3	3.0	0.0	4.0	3.4
Vegetables	27.1	8.3	12.1	17.3	16.0	16.2
Fruit trees	7.4	16.7	9.1	4.3	6.0	9.1

Source: Follow-up Socio-economic Survey, 2002

Table 23: Availability of improved seed varieties in Unguja, 2002

Farmers who said seeds are (%)	District					All districts (n=1,203)
	Central (N=501)	North-A (N=51)	North-B (n=260)	South (n=165)	West (n=226)	
Readily available	27.4	52.9	42.3	30.9	35.0	37.7
Not readily available	59.9	27.5	32.7	52.7	50.4	44.6
No-responses	12.7	19.6	25.0	16.4	14.6	17.7

Source: Follow-up Socio-economic Survey, 2002

Table 24: Average prices paid by farmers for improved seed varieties in Unguja, 2002

Crop	District					All districts (n=1,2030
	Central (n=501)	North-A (n=51)	North-B (n=260)	South (n=165)	West (n=226)	
Maize						
Average	250.0	250.0	334.6	423.1	283.3	308.2
Std. deviation	522.5	353.6	249.5	420.8	40.8	317.4
Maximum	3000	500	1000	2000.0	300.0	3000
Minimum	200	200	200	200	200	200
Observations (n)	34	2	11	26	6	79
Rice						
Average	170.45	247.83	275.9	214.29	201.8	222.0
Std. deviation	197.17	114.3	275.9	231.57	165.6	196.9
Maximum	500	500	600	800	500	800
Minimum	100	150	150	100	100	100
Observations (n)	44	23	83	14	57	221
Cassava						
Average	32.26		214.29	12.5		51.81
Std. deviation	179.61		566.95	35.36		156.384
Maximum	1000		1500	100		520
Minimum	50		50	50		50
Observations (n)	31		7	8		46
Coconut						
Average	60.0	200.0	290.0	100.0	166.7	163.3
Std. deviation	100.0	0.0	194.9	133.3	158.1	117.3
Maximum	300	200	500	300	500	500
Minimum	150	200	100	100	100	100
Observations (n)	25	1	5	10	9	50

Source: Follow-up Socio-economic Survey, 2002

3.3.5 Integration of crops and livestock

Besides increase in land area available for crop and livestock production increased after tsetse eradication as evidenced by the 1999 economic assessment survey and the follow-up survey, the proportion of farmers using land to integrate crops and livestock has also increased relative to the period before tsetse intervention in 1985/86. This has enabled a more efficient use of land resources as crop residues and by-products are fed to livestock and as animal manure is in turn used to enrich the soil. According to the 1999 Economic Assessment survey, 13% of the farmers were feeding crop by-products to livestock while 8% were feeding concentrates and 4% were providing mineral supplements to their animals (Tambi *et al.*, 1999). The results of the 2002 follow-up Survey show that the use

of crop by-products, concentrates have increased while the use of mineral supplements has decreased during the past three years. Among the farmers interviewed in 2002, approximately 22% were feeding crop by-products, 20% feeding concentrates and only 3% used mineral supplements (Table 25). North-A District has the largest number of farmers feeding crop by-products, concentrates and mineral supplements. Regarding use of improved (established) fodder, 8% of the farmers were feeding improved fodder to cattle, with the proportion varying from less than 1% in South District to approximately 16% in West District (Table 25). According to the 1999 Economic Assessment Survey, only 4% of the farmers used improved fodder as cattle feed (Tambi *et al.*, 1999). This suggests that farmers in Unguja are increasingly feeding improved fodder to cattle.

Whereas crop residues and by-products are fed to cattle, animal manure as another form of crop-livestock integration is in turn used to fertilize crop fields. According to the 1999 Economic Assessment Survey, 59% of the farmers allowed cattle to fertilize farm plots and 51% grew crops on the plots fertilized by cattle (Tambi et al., 1999). In 2002, 63% of the interviewed farmers applied and/or allowed cattle to fertilize farm plots and 54% are growing crops on the fertilized field plots (Table 26). These results suggest that use of manure as another form of crop-livestock integration is increasingly gaining prominence. The future potential of this practice is also promising according to the intentions of using manure by 31% of the sample farmers.

Table 25: Feed stuffs fed to livestock in Unguja, Zanzibar, 2002

% of farms using these feed stuffs	District					All
	Central (n=501)	North-A (n=51)	North-B (n=260)	South (n=165)	West (n=226)	Districts (n=1,203)
Improved fodder	6.1	1.5	15.8	0.5	14.8	7.9
Crop by-products	5.2	40.0	22.2	25.0	16.7	21.8
Concentrates	22.9	20.0	14.9	29.1	12.5	19.8
Mineral Supplements	1.7	0.0	2.9	0.0	5.6	3.4

Source: Follow-up Socio-economic Survey, 2002

Table 26: Use of animal manure for crop production

	District					Total (n=1,203)
	Central (n=501)	North-A (n=51)	North-B (n=260)	South (n=165)	West (n=226)	
Farmers who apply and/or allow animals to fertelize crop fields						
Number	385	47	134	110	79	755
%	76.8	92.2	51.5	66.7	34.9	62.8
Farmers who grow crops on land fertelized with animal manure						
Number	346	33	100	102	67	648
% of farmers who fertilized fields	90.0	70.2	74.6	92.7	84.8	85.8
% of total sample	73.2	47.7	32.6	58.1	27.9	53.9
Farmers who intend to use animal manure in future						
Number	106	18	123	42	82	371
% of total	21.2	35.3	43.9	47.3	36.3	30.8

Source: Follow-up Socio-economic Survey, 2002

3.3.6 Adoption of animal power

Animal power is becoming an important ingredient in the adoption of more intensive systems of livestock and crop production. Animal power used for farm work and for transport of farm produce also enhances the adoption process. In Unguja, the use of animal traction for land preparation is still low although it has increased during the past three years. In 1999, only 3% of the farmers reported using oxen for ploughing (Tambi *et al.*, 1999). The percentage of farmers using oxen has increased from 3% in 1999 to 5% in 2002 (Table 27). Furthermore, the use of animal power for transport purposes is increasingly becoming popular. While 20% and 16% of the farmers were respectively using animals to transport their own products and commercial transport in 1999, the percentage of farmers using animals for own and commercial transport has increase slightly to 21% and 18% in 2002, respectively (Table 27). The future potential of the use of animal power in Unguja also appears to be very encouraging as 60% and 30% of the sample farmers indicated their intentions to use animals for ploughing and transport purposes, respectively.

Table 27: Use of farm animals for traction and transport, 2002

Farmers who use animal power for:	District					All districts (n=1,203)
	Central (n=501)	North-A (n=51)	North-B (n=260)	South (n=165)	West (n=226)	
Plowing: Number	34	4	13	7	4	62
%	6.8	7.8	5.0	4.2	1.8	5.1
Own transport: Number	154	11	45	15	62	287
%	30.7	21.6	17.3	9.1	27.4	21.2
Commercial transport: Number	111	9	50	13	34	217
%	22.2	17.6	19.2	7.9	15.0	18.0
Farmers who intend to use animal power in future for:						
Plowing: Number	332	19	125	106	144	726
	66.3	37.3	48.1	64.2	63.7	60.3
Transport: Number	136	36	99	32	57	360
%	27.1	70.6	38.1	19.4	25.2	29.9

Source: Follow-up Socio-economic Survey, 2002

3.3.7 Dairy intensification

A good indicator of intensification in dairy production is the degree to which dairy farmers use improved cattle breeds and how well they manage the animals through better feeding and disease control. A good dairy processing and marketing system also offers more access to market outlets as well as the purchase of inputs, thus enhancing intensification of dairy production. In Zanzibar, intensification of dairy production is constrained by inadequate finances for purchasing improved dairy cattle breeds; use of poor quality feed stuffs consisting mostly of local pastures; low adoption of improved feeding methods involving the use of crop residues and by-products, concentrates and mineral supplements; and the absence of a reliable and efficient dairy marketing system. The following sections provide information about the current status of dairy cattle productivity, dairy feeding and breeding practices in Unguja.

3.3.7.1 Dairy cattle productivity

To establish productivity parameters for intensified dairy production, a sample of 60 farmers who specialized in raising improved cattle (did not keep indigenous cattle at all) was selected from a total of 290 farmers who reported that they were raising improved cattle. According to Table 28, age at first calving for 53 out of the 60 dairy farmers averaged 2.6 (± 1.0 sd) years with a minimum of 1.5 years and a maximum of 4.0 years. The calving interval averaged 13.3 (± 3.3 sd) months with a minimum of 12 months and a maximum of 18 months. Daily milk production averaged 8.3 liters with a rainy season production of 9.1 liters and a dry season production of 7.5 liters. Maximum potential milk production is 20.0 liters. On average, cows stayed in milk for 7.2 months, the longest being 11 months and the shortest being 3.5 months. When compared with the values of the productivity parameters reported in 1999 (Tambi *et al*., 1999), productivity of improved dairy cattle has improved during the past three years. According to Tambi *et al*. (1999), age at first calving for 39 dairy farmers averaged 3.1 years with a minimum of 1.7 years and a maximum of 7.6 years while calving interval averaged 14.5 months with a minimum of 12 months and a maximum of 22 months. The same survey reported an average daily milk production of 8.2 liters with a rainy season production of 8.9 liters and a dry season production of 7.4 liters.

Table 28: Production and reproduction parameters for dairy cattle in Unguja, Zanzibar, 1999

Parameter	Dairy farmers responding		Average	Std. de.	Maximum	Minimum
	Number	%				
Age at first calving (years)	53	88.3	2.6	1.0	4.0	1.5
Calving interval (months)	47	78.3	13.3	3.3	18	12.0
Lactation length (months)	51	85.0	7.2	1.4	11.0	3.5
Milk production (liters/cow/day):						
Rainy season	48	80.0	9.1	4.1	19	3
Dry season	42	70.0	7.5	3.3	20	1.5
Average	48	80.0	8.3	3.8	20	1.5

Source: Follow-up Socio-economic Survey, 2002

3.3.7.2 Dairy cattle feeding practices

Among the 60 dairy farmers, 92% zero-grazed their cattle while 3% practised semi-zero-grazing. The remaining 5% grazed their improved dairy cattle on the rangelands. According to the 1999 Socio-economic survey (Tambi *et al*., 1999) 87% of project dairy farmers studied zero-grazed their cattle, 4% practiced semi-zero grazing and 17% grazed their cattle on the rangeland. The increase in the proportion of farmers practicing zero-grazing from 87% in 1999 to 92% in 2002 suggests that more farmers are increasingly intensifying dairy cattle feeding. A range of feed materials used for feeding improved dairy cattle was reported by farmers who practiced zero-grazing (Table 29). Most (77%) of the farmers practicing zero-grazing still depend on natural pasture as a major dairy cattle feed. Farmers using crop by-products, improved fodder, concentrates and mineral supplements accounted for 63%, 60%, 80% and 53% of the farmers practicing zero-grazing (Table 29). Improved fodder used include Elephant grass, Guatemala and Glericidia while

concentrates used include maize bran, sunflower cake and pollads. According to Tambi et al. (1999) crop by-products, improved fodder, concentrates and minerals supplements respectively were used by 56%, 57%, 80% and 56% of the farmers who practiced zero-grazing. Comparison of the situation in 1999 and the 2002 situation suggest that while use of crop by-products and improved fodder has increased since 1999, the proportion of farmers using concentrates and mineral supplements has remained more or less the same. For farmers who fed concentrates, the amount fed per day averaged 0.7 kg per cow per day with a minimum of 0.5 kg and maximum of 4 kg. per cow per day. In most cases, concentrates are fed to cows in milk during milking. The amount of mineral supplements (salts) given to cattle are also low, averaging less than 1 gm per cow per day.

For those farmers who use natural grass as a major feed for improved dairy cattle, the average cost incurred is 53.5 per kg with a minimum cost of TShs. 20 per kg. and a maximum of TShs. 140 per kg. The cost associated with the use of natural pastures is usually the cost of labour for collection of the grass and the amount of feed collected is usually measured using *polos* (one *polo* equivalent to 50kg). The cost of concentrates range from an average of TShs. 67.4 per kg of maize bran to TShs. 124.5 per kg of sunflower cake while cost of mineral supplements averaged TShs. 1,625 per kg (Table 30). The cost of concentrates and mineral supplements depend on the source. Maize bran and rice pollard were readily available locally and their prices were relatively lower than sunflower cake, wheat pollard and minerals which are usually obtained from Tanzania Mainland.

Table 29: Feeding systems and feed stuffs fed to dairy cattle in Unguja, Zanzibar, 2002

Feeding system and feeding material used	Dairy farmers using	
	Number	%
Zero grazing	55	91.6
Semi-zero grazing	2	3.4
Extensive free range	3	5.0
Feed stuffs:		
Natural pasture	46	76.8
Crop by-products	38	63.3
Concentrates	48	80.0
Mineral supplements	32	53.4
Improved (planted) fodder	36	60.0

Source: Follow-up Socio-economic Survey, 2002

Table 30: Cost of feed staffs used, 2002

Feed staff	Cost per kg			
	Average	Std. deviation	Minimum	Maximum
Natural grass	53.5	9.4	20.0	140.0
Maize bran	67.4	56.7	40.0	200.0
Sunflower cake	124.5	14.7	100.0	180.0
Pollards	51.6	5.4	40.0	70.0
Mineral supplement	1,625.0	530.3	1,000.0	2,500.0

Source: Follow-up Socio-economic Survey, 2002

3.3.7.3 Dairy cattle breeding practices

The main methods of breeding dairy cattle reported by the 60 dairy farmers are use of Artificial Insemination (AI) (72%) and use of improved bulls by 68% of the dairy farmers. Use of local bulls was not very popular among the improved dairy keepers as was reported by only 25% of the farmers (Table 30). Farmers who did not own improved bulls acquired improved bull services from neighbours and other farmers. For those who used local bull services, 10% used own bulls and 15% obtained services from their neighbours. Most (67%) of the farmers who used AI obtained the services from the government AI Center (Table 30).

The average cost incurred for improved bull services was estimated at TShs. 304 with a maximum of TShs. 8,000 and a minimum expenditure of TShs. zero (free of charge). Local bull services cost an average of TShs. 82 with a maximum of TShs. 3000 and minimum of zero while AI services were obtained at an average cost of TShs. 1187 with a maximum of TShs. 8,000 and a minimum of TShs. 500 (Table 30).

Table 30: Breeding methods for dairy cattle and sources of breeding materials in Unguja, Zanzibar, 2002

Breeding system and type of breeding material	Dairy Farmers responding			
	Number	%		
Use of improved bulls	41	68.3		
Use of artificial insemination	43	71.7		
Use of local bulls	15	25.0		
Source of improved bull:				
Own bull	9	15.0		
Neighbours and other farmers	35	58.3		
Source of AI:				
AI Centre	40	66.7		
Extension staff	3	5.0		
Source of local bull:				
Own bull	6	10.0		
Neighbours and other farmers	9	15.0		
Cost of (TShs):	Average	Std. dev.	Maximum	Minimum
Improved bull service	304	644	8000	0 (free)
AI Services	1187	974	8000	500
Local bull services	82	298	3000	0 (free)

Source: Follow-up Socio-economic Survey, 2002

3.3.8 Milk processing and marketing at the farm level

3.3.8.1 Milk processing

In Unguja, Zanzibar, milk processing is currently not well developed. Industrial processing of milk by the Zanzibar Dairy Corporation stopped in 1995 following the closure of the milk processing plant in Zanzibar town. The plant was producing pasteurised milk,

yoghurt, cream, butter and ghee[2]. Limited processing of milk is undertaken at the farm level by simply fermenting raw milk. In 2002, about 2% of the sample farmers fermented milk and most of the fermented milk was consumed at home. Less than 1% of the farmers fermented milk for sale. Thus most of the milk in rural Unguja is marketed in its raw form, neither processed nor pasteurized.

3.3.8.2 Milk marketing

Sixty two percent of the sample farmers marketed milk in 2002. As pointed out above, most of the milk is sold in its raw form. Table 31 shows the quantities of milk sold per household per day in 2002. Quantities sold ranged from 1 to 74 litres with an average of 6.2 litres per farm per day. Farms in Central and West districts marketed larger quantities of milk than the other three districts (Table 31).

When asked whether they faced problems of disposing off their milk or not, 93% of the farms which sold milk indicated that they had no problems of disposing off their milk. The few farmers who had problems experienced the problems during the rainy season. None of the farmers experienced problems of disposing off milk during the dry season. The unsold milk is either consumed at home or given away to relatives and neighbours.

Since there is no organized system of milk collection in most rural areas in Unguja, Zanzibar, farmers sell their milk through different market outlets that exist in their locality. Six alternative channels through which farmers sell their milk were reported in Central and West Districts. Other districts had less than six alternative milk marketing channels (Table 32). Most (78%) of the milk is sold to vendors (middlemen). In most cases the vendors collect the milk from the farmers and sell directly to consumers in urban areas. Zanzibar town is the main urban market for milk and other dairy products in Unguja. Vendors used bicycles and public transport to ferry milk from the producers to consumers in urban areas. Organized milk collection centres operate in Central District only and there is only one well established milk collection centre in the district. Institutions which purchase milk directly from farmers include the Tanzania Communication Company Limited (TCCL), Bank and Tanzania Posts. These operate in the Central and West Districts. Three types of containers are used for handling milk: plastic containers, metal (aluminium) containers and bottles. Bottles such as empty beer bottles are normally used by producers who sell small quantities of milk directly to consumers. Plastic and metal (aluminium) type of containers are used by vendors and producers who sell relatively large quantities (above 4 litres) of milk. Plastic containers and bottles are not hygienically recommended because they cannot be cleaned easily.

---
[2] The processing plant has been recently sold to a private investor but it has not yet resumed production.

Table 31: Quantities of milk sold in litres per farm per day, 2002

	District					All Districts
	Central	North-A	North-B	South	West	
% of farmers selling milk	66.7	51.0	76.9	45.5	69.0	61.8
Average quantity sold	7.8	4.7	5.8	4.3	8.4	6.2
Std deviation	8.4	3.6	4.7	2.6	86	5.6
Maximum quantity sold	74.0	17.0	32.0	10.0	49.0	74.0
Minimum quantity sold	1.0	1.0	1.0	1.0	1.0	1.0

Source: Follow-up Survey, 2002.

Table 32: Proportion of milk passing through different marketing channels in Unguja, Zanzibar

Market outlet	District					All Districts
% milk sold to:	Central	North-A	North-B	South	West	
Household consumers	5.0	34.9	2.1	13.5	6.6	9.7
Vendors/hawkers	83.8	43.6	97.9	84.9	90.5	78.2
Kiosks/retail shops	3.0	14.2	0.0	0.8	1.2	3.6
Milk collection centre	2.5	0.0	0.0	0.0	0.0	3.2
Hotels/restaurant	3.0	7.3	0.0	0.8	0.1	2.3
Institutions	2.7	0.0	0.0	0.0	1.6	3.0

Source: Follow-up Survey, 2002.

Prices received by farmers from the different outlets varied slightly. Farmers who sold their milk to institutions, kiosks and hotels received relatively higher prices than farmers who sold to other customers (Table 33). Irrespective of the market outlet, there is no significant difference in milk prices between the dry and wet season (Table 33).

Table 33: Prices of milk received by farmers during wet and dry season, 2002

Market outlet	Price received by farmers (Shs/litre)	
	Wet season	Dry season
Household consumers	120-270	130-270
Vendors/hawkers	130-200	140-200
Kiosks/retail shops	140-350	140-350
Milk collection centre	205	205
Hotels/restaurant	150-300	150-300
Institutions	350	350

Source: Follow-up Survey, 2002.

3.4 Animal disease and other constraints to dairy intensification in Unguja, Zanzibar

Prior to the eradication of tsetse flies in Unguja, Trypanosomosis was the main constraint to dairy intensification and livestock development as whole in the Island. Following eradication of tsetse flies in 1997, farmers are increasingly intensifying dairy production as evidenced by the increase in the proportion of farmers raising improved cattle, use of improved seeds, increased use of crop by-products as animal feed, use of manure for crop production and use of animal power for ploughing and transport activities. However, there are still other constraints which slow down the rate at which farmers intensify their production. These constraints include low adoption of improved dairy cattle due to high

costs of improved dairy heifers and low financial ability among resource poor farmers, inadequate feeding, poor quality feed stuffs consisting mainly of local grass with low use of concentrates and mineral supplements, absence of organized milk marketing system as discussed in the previous sections.

The other major constraint to dairy intensification that has not been discussed in the previous sections is existence of animal diseases other than trypanosomosis. Although tsetse and trypanosomosis have been eliminated after eradication of tsetse flies, there are still other diseases that constrain dairy development in Unguja. When farmers interviewed were asked to rank major animal diseases in their order of importance, most of them reported East Coast Fever (84%), Helminthiasis (34%), Lumpy skin disease (28%), as important diseases constraining dairy production and livestock production as a whole (Table 34). In the 1999 Socio-economic Survey (Tambi et al, 1999), similar diseases were reported as important animal diseases constraining livestock production although the order of importance has slightly changed. For example, Lumpy Skin Disease and Helminthiasis which were ranked second and third in the 1999 survey were ranked third and second respectively in the 2002 follow-up survey.

Table 34: Farmers' ranking of the importance of animal diseases currently affecting livestock in Unguja

Diseases	District					Total (n=1,203)
	Central (n=501)	North-A (n=51)	North-B (n=260)	South (n=165)	West (n=226)	
East Coast Fever	84.6	84.3	83.5	83.0	84.5	84.0
Helminthiasis	36.5	35.3	38.8	23.0	37.2	34.2
Lumpy Skin Disease	24.6	33.3	37.3	15.2	29.6	28.0
Pneumonia	0.4	45.1	8.1	4.2	8.8	13.3
Heart Water	13.6	5.9	13.8	5.5	16.8	11.1
Foot and Mouth Disease	4.8	0.0	8.1	2.4	13.7	5.8
Other Diseases	7.8	43.1	20.4	7.2	19.0	19.4

Source: Follow-up Survey, 2002/03

To examine the effect of the disease which was ranked first, the proportion of farmers with affected cattle and died from the disease considered most important (ECF) were estimated. Approximately 41% of the sample farmers reported that their cattle were affected and died from ECF with the largest proportion and smallest proportion of farmers reporting being in North-A and South Districts, respectively (Table 35). Forty one (8%) of the 488 farmers who reported cattle deaths due to ECF infection were raising improved dairy cattle (Table 35). With regard to disease management most (57%) of the farmer treated their animals after being infected as compared to those who took preventive measures (22%) (Table 35). However, the low proportion improved dairy cattle keepers who reported deaths due to ECF infection may suggest effective disease control measures among farmers raising improved dairy cattle. The cost of ECF prevention and treatment in 2002 averaged TShs. 6,209 with a minimum of TShs. 750 and maximum of TShs. 300,000 per farm.

Table 35: Effects of East Coast Fever (ECF) in Unguja, 2002

	District					All districts (n=1203)
	Central (n=501)	North-A (n=51)	North-B (n=260)	South (n=165)	West (n=226)	
% Sample farmers with cattle dead from ECF	36.3	70.6	62.6	33.3	43.4	40.6
Farmers with improved dairy cattle dead as % of above	16.5	2.8	6.2	3.6	13.3	8.4
% farmers using preventive measures	20.4	19.6	26.5	18.2	22.6	21.5
% farmers who treated animals	60.5	56.9	51.2	61.2	54.4	56.8
**Cost of ECF control and treatment:**						
Average cost	6,547	6,092	6,488	2,898	9,019	6,209
Std. deviation	17,663	7,508	9,911	3,251	18,889	11,444
Maximum	300,000	32,000	75,000	20,000	200,000	300,000
Minimum	2,600	1,200	1,700	750	3,100	750

Source: Follow-up Survey, 2002/03

3.5 Assessment of overall domestic production, consumption needs and current market for dairy products in Zanzibar

3.5.1 Domestic milk production

Projections of milk production made in 1999 using three different performance scenarios (low, medium and high) indicate that total milk production would increase by 19% by the year 2005 with the rate of increase doubling by 2010 and more than tripling by 2015. Under the medium performance scenario, total milk production was projected to increase from 6.1 million liters in 1999 to 11.5 million liters by year 2015. Under the optimal production conditions assumed under the high performance scenario, domestic milk production in Zanzibar was projected to more than double from 11.2 million liters in 1999 to 24.4 million liters by the year 2015 (Table 36). Comparison of these projections with secondary data from the Ministry of Agriculture, livestock, and Natural Resources indicate that milk production has increased from 5,720,000 litres in 1999 to 6,069,000 litres in 2001, an increase of 3% per annum. With the 3% growth rate milk production in 2002 is estimated at 6,252,000 litres and would increase to 6,832,000 litres in 2005. This production level is below the projected quantity of milk using the medium performance senario but above the projected quantity using the low performance scenario (Table 36).

3.5.2 Assessment of total consumption needs (demand) for milk in Zanzibar

Based on the estimated consumption needs of 25 litres of milk per capita per year and the total population of 984,625 (Population Census, 2002) (Annex 6), total milk consumption needs in Zanzibar in the year 2002 are estimated at 24,615,625 litres. Comparison of total consumption needs (demand) with the estimated milk production of 6,252,000 litres in 2002 suggest that domestic production was able to meet only 25% of demand for milk in Zanzibar. With a population growth rate of 3.1%, total milk consumption needs (demand) would grow from 24,615,625 litres in 2002 to 26,976,578 litres in 2005. When these consumption needs are compared with the projected quantities in Table 36 it is clear that Zanzibar will continue to experience a deficit of milk and will continue to rely on imports unless concerted efforts are made to increase domestic production. Annex 7 summarizes

quantities of imported products during the past three years (2000-2002). The figures in Annex 6 show that Zanzibar imported more than 10 different dairy products from eight different countries with the United Arab Emirates being the major source of dairy products consumed in Zanzibar during the past three years.

Table 36: Projections for milk production per capita in Unguja, Zanzibar, 1986-2015

Year	Low scenario	Medium scenario	High scenario
	Total (1,000 liters)	Total (1,000 liters)	Total (1,000 liters)
1999	3,619	6,119	11,167
2005	4,321	7,743	14,965
2010	5,009	9,421	19,099
2015	5,807	11,462	24,376

Assumptions

1. Total cattle population estimated to grow at 3% , 4% and 5% per year for low, medium and high scenarios respectively
2. Proportion of indigenous cattle is estimated to be 94%, 92% and 90% for low, medium and high scenarios respectively
3. Cattle herd structure is composed of 41.23% cows for both indigenous and improved cattle.
4. Proportion of improved cattle is estimated at 6%, 8% and 10% for low, medium and high scenarios respectively.
5. Calving rates for indigenous cattle are assumed to be 55%, 60% and 65% for low, medium and high scenarios respectively
6. Calving rates of improved cattle are assumed to be 67%, 76% and 85% for low, medium and high scenarios respectively
7. Lactation lengths for indigenous cattle are estimated at 180, 200 and 220 days per year for low, medium and high scenarios respectively
8. Lactation lengths for improved cattle are estimated at 280, 305 and 320 days per year for low, medium and high scenarios respectively

3.5.3 Assessment of the current market for milk and milk products in Unguja, Zanzibar

Section 3.3.8 examines aspects of processing and marketing of milk at the farm level. This section reports market related information obtained from interviews with household consumers and owners or managers of hotels, restaurants and kiosks.

3.5.3.1 Dairy products purchased and consumed

Results from the interviews with household consumers and owners of hotels, restaurants and kiosks provide further evidence that most of the dairy products consumed in Zanzibar are imported. Of the 15 hotel owners or managers interviewed, only 4 (27%) purchased locally produced dairy products for their customers while only 5 out of the 20 kiosk owners reported that they sell locally produced dairy products. With regard to household consumers, 16 out of the 30 (53%) sample households consumed only locally produced dairy products and the remaining 14 (47%) consumed both locally produced and imported dairy products. Most (60%) of the households purchased locally produced milk directly from dairy cattle producer while 37% and 3% purchased their milk from vendors and kiosks, respectively.

When asked if locally and imported dairy products are readily available throughout the year, almost all the hotel owners/managers and household consumers who use locally produced dairy products indicated that it is difficult to get locally produced milk during the dry season. Therefore they are compelled to reduce the quantities consumed or use imported milk products during dry season. Almost all consumers of imported dairy products acknowledged that they are readily available throughout the year. Major imported dairy products that were reported by the household consumers and owners of hotels and kiosks include powdered milk, condensed milk, UHT milk, yoghurt and cheese.

3.5.3.2 Preferences for milk and milk products

Household consumers were asked to indicate the type of milk products they would prefer. They were asked to choose between locally produced and imported milk products as well as between raw milk and processed milk. Similarly, owners of hotels and kiosks were asked to indicate whether they prefer to purchase locally or imported products for their customers. It is interesting to note that most (80%) of the household consumers highly prefer locally produced products. However, owners and managers of hotels, restaurants and kiosks seem to prefer imported products. Table 37 summarizes reasons given by both household consumers and owners or managers of hotels, restaurants and kiosks for and against preference of local and imported dairy products. Although the number of reasons in favour of local products compared to the reasons in favour of imported products is the same, there are more reasons why locally produced products are not preferred (Table 37). This is largely due to the fact that almost all locally produced milk is sold in its raw form (unprocessed) while imported products are sold as processed products with proper packaging.

When the sample households were specifically asked to indicate whether they prefer raw or processed milk, 63% of them reported that they prefer raw milk. The major reason for their preference is that raw milk is tastier than processed milk because the nutrients in the raw milk have not been disturbed through processing. The remaining 37% that prefer processed milk gave the reason that processed milk is of high hygienic quality compared to raw (unprocessed) milk because the milk is pasteurised and properly packaged to avoid contamination.

Table 37: Reasons given by sample households for and against local and imported dairy products

Reasons in favour of:	Reasons against:
Locally produced dairy products	*Locally produced dairy products*
• They can be purchased on credit<br>• They are in raw form and nutrients have not be destroyed<br>• They can be transformed into other products<br>• Their prices are low during wet season<br>• We promote our local farmers when we buy them	• Supply is not constant (Low supply in dry season)<br>• Sometime they deteriorate before boiling<br>• Hygiene of the products not assured<br>• Less preferred by children<br>• Not preferred by tourists<br>• They have high butter fat content and not suitable for people with fat problems
Imported dairy products	*Imported dairy products*
• Hygiene is assured as they meet quality standards<br>• They can be stored for long period<br>• They are available throughout the year<br>• They are preferred by children especially sweetened products<br>• People with fat problems can get products with low butter fat content	• They are very expensive compared with local products<br>• Their nutrient content has been altered<br>• They are sometimes sold beyond their expiry dates especially in rural areas hence possibility of health hazards to consumers

Source: Market survey, 2002/03

3.5.3.3 Quantities of milk and milk products consumed

Table 38 shows the quantities of major milk products purchased and consumed in the sample hotels and restaurants in Unguja. The major dairy products consumed in the sample hotels are raw milk (locally produced), UHT milk, yoghurt, powdered milk and cheese. Average quantities consumed range from 4.0 kg of cheese per day (435.4 kg. per season) to 23.4 litres of UHT milk per hotel per day (2,458.5 litres per season). The maximum amount of 7,500 litres/season of UHT milk reported was consumed in Kiwengwa Stand hotel. The same hotel consumed the maximum amount of yoghurt (2250kg/season) while the maximum amount of powdered milk (6,000 kg/season) and cheese (2400 kg/season) were consumed in Vacanze hotel (Table 38). The quantity of milk product consumed by each hotel per season depends on the type and number of visitors or tourists. Average number of visitors per hotel in 2002 was 238 visitors with a maximum of 1200 visitor and a minimum of 14 visitors. The maximum number of visitors was recorded in Serena Inn and the minimum number was recorded in Paradise Beach Bangalow.

Table 38: Zanzibar: Quantities of major dairy products consumed in hotels

Dairy product	Quantity consumed per hotel per season		
	Average	Maximum	Minimum
Raw milk (litres)	826.9	1312.5	270.0
UHT milk (litres)	2458.5	7500.0	375.0
Yoghurt (kg)	708.3	2250.0	30.0
Powdered milk (kg)	869.4	6000.0	30.0
Cheese (kg)	435.4	2400.0	19.0

Source: Market survey, 2002/03

Dairy products consumed by households include raw milk (locally produced), UHT milk, milk powder, yoghurt and cheese. Unlike hotels, the quantities of UHT milk, yoghurt and cheese consumed are extremely small and very few households reported their consumption. Table 39 shows quantities of each product consumed per household per month during wet and dry season. Average quantities consumed during the dry season are slightly lower than quantities consumed during wet season although the difference is not significant.

Table 39: Zanzibar: Quantities of dairy products consumed by households during wet and dry season

Dairy product	Quantity consumed per household per month					
	Wet season			Dry Season		
	Average	Maximum	Minimum	Average	Maximum	Minimum
Raw milk (litres)	27.9 (30)	60.0	4.5	26.2	60.0	4.5
UHT milk (litres)	15 (1)	15	15	15.0	15.0	15.0
Yoghurt (kg)	4.5 (4)	7.5	1.5	0.0	0.0	0.0
Powdered milk (kg)	1.5 (14)	2.5	0.5	1.3	2.0	0.5
Cheese (kg)	0.25 (1)	0.25	0.25	0.25	0.25	0.25

Figures in brackets are number of households consuming the product
Source: Market Survey, 2002/03

Prices paid for the various dairy products during the wet and dry seasons are summarized in Table 40. Prices of locally produced dairy products such as yoghurt are significantly lower than imported yoghurt. The price of UHT milk significantly higher than locally produced raw milk (almost three times as high). This is probably due to value adding through processing, packaging, and freight and other charges associated with importation. High price was one of the reasons given by the households interviewed for not preferring imported dairy products.

Prices of dairy products do not vary significantly between the wet and dry season. For locally produced dairy products there is slight difference between the wet season and dry season prices. On the other hand, there is no seasonal difference in the price of imported dairy products (Table 40).

Table 40: Zanzibar: Quantities of dairy products consumed by households during wet and dry season

Dairy product	Price of dairy product per litre or kg					
	Wet season			Dry Season		
	Average	Maximum	Minimum	Average	Maximum	Minimum
Raw milk (local)	333.3	450.0	250.0	335.0	450.0	250.0
Imported UHT milk	1070.0	1400.0	800.0	1070.0	1400.0	800.0
Imported yoghurt	1045.0	1250.0	900.0	1045.0	1250.0	900.0
Local yoghurt	733.3	1000.0	600.0	738.4	1100.0	600.0
Cheese (Imported)	9831.0	12500.0	6500.0	9831.0	12500.0	6500.0
Imported Milk powder	2267.0	2800.0	2000.0	2267.0	2800.0	2000.0

Source: Market Survey, 2002/03

4.0 Socio-economic Impact of Tsetse Eradication in Unguja, Zanzibar

In an attempt to determine the impact of tsetse control/eradication in Unguja, Zanzibar, an analytical framework for assessing the economic impact of the intervention was developed in 1999 (Tambi *et al*., 1999). This framework is designed to answer three important questions. The first relates to the public investment nature of the intervention (tsetse control/eradication). Does or will the intervention generate sufficiently large benefits to justify the investment costs incurred? Secondly, who are the main beneficiaries of the eradication intervention? To what extent do not only livestock and crop producers benefit from the absence of trypanosomosis, but also consumers from the increase in supply of crop and livestock products? Finally, what lessons can effectively be drawn from how the control/eradication intervention was implemented?

To measure the economic impact of a disease control/eradication intervention the framework compares the value of the outcome with the intervention in relation to the value of the outcome without the intervention. Returns or benefits derived from the intervention represent the additional earnings realised if the intervention takes place compared to what would or would have happened in its absence. Benefits and costs of the intervention are measured as the incremental changes between the intervention and the non-intervention scenario. Benefit-cost analysis then compares the value of the benefits with the value of the costs as a guide to determine whether the intervention is economically superior to the alternative scenario. The intervention (tsetse eradication) will be considered to have positive impact if the benefit-cost ratio is greater than one. This means that the discounted benefits should outweigh the discounted costs.

There are generally two types of costs associated with a disease control/eradication intervention: (i) Costs incurred to control/eradicate the disease that would not have been incurred in the absence of the intervention; and (ii) any livestock production revenues foregone that are no longer earned due to the control/eradication measures. There are also two types of benefits from disease control/eradication: (i) Increased revenue from improved productivity, i.e. revenue due to avoided production losses from mortality and morbidity, as well as (ii) savings in control costs avoided after achieving eradication. Incremental benefits are estimated as the difference between production value obtained “with” intervention versus the “without” scenario. The “with” scenario represents what is actually obtained with the intervention and so is usually based on available data. The “without” scenario, on the other hand, describes what was happening “before” the intervention or would have happened “after” had the intervention not taken place.

With regard to what is actually happening with the intervention (after tsetse control/eradication), the results of the 1999 economic assessment survey (Tambi et al., 1999) and the most recent follow-up survey (2002) show that livestock productivity in Unguja has increased since the eradication of tsetse flies as evidenced by reduced mortality rates, shorter calving intervals, increased milk yields and reduction in some of the constraints on livestock production and crop-livestock integration. However, there are still some constraints on livestock production and crop-livestock integration which prevent farmers from obtaining the full benefits of tsetse eradication. As pointed out earlier in this report constraints on livestock production include problems of diseases other than trypanosomosis (particularly ECF, Helminthiasis and Lumpy Skin Disease), low adoption

of improved cattle due to low financial ability to acquire improved cattle and inadequate feeding including low use of concentrates and mineral supplements. Due to these constraints production of meat, milk, hides and skins, manure and workforce from animals is still low. With regard to constraints on crop-livestock integration, there is still low use of manure for crop production, low use of crop by-products as animal feed and animal traction for plowing and transport although the proportion of farmers using manure, crop by-products and animal traction has increased since the eradication of tsetse flies in 1997.

Since constraints on livestock production and crop production have not been eliminated, it is not wise at this time (only 5 years after eradication) to use criteria like benefit-cost ratio and net present value for making a very crucial decision on whether tsetse control/eradication in Unguja has had positive economic impact or not. Resource poor smallholder farmers usually take time to adopt improved technologies especially if more than one technology is essential for increased production. For intensified livestock production, the potential of improved cattle to produce meat, milk, hides and skins, manure and animal power for cultivation and transport can be fully exploited if farmers adopt improved cattle with improved feeding practices and animal health services. Due to the constraints mentioned earlier 60% of the sample who have adopted improved cattle breeds with a high production potential like cross breeds have adopted improved feeding practices. On the other hand, 94% of the interviewed farmers are raising indigenous cattle which have a low potential for meat, milk and manure production. There is a limit these farmers can increase production even if they adopt improved feeding and animal health care.

Although it is not the appropriate time to assess the impact of tsetse control/eradication using criteria such as benefit-cost ratio, comparison of the performance of livestock and crop production before tsetse control/eradication with the post-tsetse (1997-2002) performance suggest that the eradication of tsetse and typanosomosis in 1997 has resulted in significant gains including increased milk production, manure and power for ploughing and transport activities as pointed out in the previous sections. Of particular importance is the fact that some of the milk produced is consumed at home. On average, about 20% of the milk produced per farm is consumed at home as raw milk and about 1% is consumed as fermented milk. This suggests that the increase in milk production following tsetse and typanosomosis eradication has also contributed to improvement of the nutritional status of rural households in Unguja, Zanzibar.

Another evidence of the positive socio-economic impact of tsetse and trypanosomosis eradication is the increase in the average household income and the proportion of farmers who moved from low-income to high-income strata since 1999. The average household income has increased by almost 30% from 41,232 TSHS in 1999 to 53,502 TSHS in 2002. Besides the increase in average household income, it is interesting to note that a large proportion of farmers have moved from low-income to high-income strata. For example, the proportion of farmers with household income below 25,000 TSHS per month has declined from 30% in 1999 to 12% in 2002 (Table 41). Although it is difficult to isolate the effects of tsetse eradication from other factors since several factors may have contributed to the increase in household income, it is still logical to attribute the increase in household income to tsetse and trypanosomosis eradication. A correlation analysis carried out to ascertain if there is any association between the household income and the

increases in milk yields, milk sales, use of manure and animal power for cultivation and transport that have occurred after eradication shows that there is strong positive correlation between the household income and all the variable. This strong correlation suggests that the increase in the household income between 1999 and 2002 is associated with tsetse eradication.

Table 41: Comparison of average household income and proportion of households by income categories

Household income	Year	
	1999	2002
Average (TSHS)	41,232	53,502
% farmers within this income group:		
Less than 25,000 TSHS	30.4	12.1
25,000 to 50,000 TSHS	47.6	49.6
50,001 to 100,000 TSHS	18.8	31.0
100,001 to 200,000 TSHS	2.2	4.8
Greater than 200,000 TSHS	0.9	0.7

4.1 Implications of the results for agriculture and livestock development in Unguja, Zanzibar

In general the results of the economic assessment survey of 1999 and the follow-up survey of 2002 both suggest that tsetse eradication has stimulated livestock production as well as increased crop-livestock integration. The results of the 1999 survey have shown that cattle herd sizes have increased between 1985/86 and 1999, suggesting that tsetse control and eradication measures undertaken between 1985/86 and 1997 have stimulated livestock growth. Similarly, the results of follow-up survey of 2002 show that cattle herd sizes have continued to grow between 1999 and 2002. Furthermore, the results of both surveys indicate that the growth in cattle herd sizes is accompanied by increase in milk yields, sale of milk, manure production and use of manure for crop production, and animal traction for cultivation and transport of agricultural products.

Although evidence from the survey results suggests that tsetse eradication has stimulated livestock and agricultural production in Unguja-Zanzibar, the current rate of adoption of livestock technologies is not large enough for attaining growth that will meet the demand for milk and other livestock products. For example, the estimated milk production of 6,252,000 litres in 2002 could meet only 25% of the consumption needs (demand) of 24,615,625 litres (Table 36). At a population growth rate of 3.1%, total milk consumption needs (demand) is projected to grow from 24,615,625 litres in 2002 to 26,976,578 litres in 2005. Even with the optimistic cattle growth rate of 5%, milk production is expected to be 14,965,000 litres in the year 2005. This suggests that Zanzibar will continue to experience a deficit of milk products and will continue to rely on imports unless concerted efforts are made to increase domestic production. Therefore, there is need for further intervention (following eradication of tsetse flies and trypanosomosis which have stimulated growth in livestock and agricultural production) to remove constraints and accelerate adoption of technologies which are necessary for intensification such as use of crossbred animals which have relatively high production potential, use of improved seed varieties, use of improved breeding methods, disease control and adequate animal nutrition.

The need for further intervention to accelerate growth in agriculture and livestock production is justified by the fact that household income is still low although it has increased by 30% from an average of 41,232 TSHS per month in 1999 to 53,502 TSHS per month in 2002. This underlines the need for intervention and justifies streamlined efforts and increased investments in livestock production for poverty reduction in rural areas of Unguja, Zanzibar.

From the results of the survey, the following are identified as areas that require further intervention in order to accelerate adoption and growth in livestock production:

(i) The results of both the 1999 economic assessment survey and the 2002 follow-up survey revealed that most of the farmers in Unguja-Zanzibar keep indigenous cattle with a low potential for meat and milk production (capable of producing a maximum of 3 litres of milk per cow/day). Furthermore, the results of the two surveys indicate that the acquisition of improved livestock breeds such as crossbred animals to replace indigenous cattle is mainly constrained by low financial ability among the resource poor smallholder farmers as indicated by 46% of the farmers interviewed in 1999 and 52% of those interviewed during the follow-up survey in 2002. The cost of crossbred cattle heifers, for example, has increased by 48% from an average of 182,000 TSHS in 1999 to 269,000 TSHS in 2002.

Although a heifer in trust scheme has already been introduced in some villages, there is need to scale up this effort in order to speed up intensification by involving more stakeholders including the government of Zanzibar, CBOs and NGOs interested in dairy development.

(ii) Efforts to promote adoption of improved cattle through heifer in trust schemes should go hand in hand with upgrading of the existing indigenous stock through selection and use of improved bulls and AI. The use of AI and improved bulls is also important for sustaining the genetic make up of improved stock. Although the use of improved bulls and AI is increasingly becoming popular among the farmers raising improved cattle, only 14% and 22% of all the sample farmers used improved bulls and AI respectively.

Interventions that will promote use of AI include equipping the Government AI Centre which is currently a major provider of AI services in Unguja, Zanzibar as well as building the capacity of staff in the provision of these services. The use of improved bull services can be promoted by encouraging farmers to establish bull centres. A system of rotating the bulls among the Centres which will be established should be put in place to avoid possibilities of inbreeding which are likely to occur if a bull stays in one centre for a long time.

Effective use of AI and bull services requires knowledge on timely heat detection. For this reason farmers should be trained on heat detection and record keeping.

(iv) Although tsetse flies and trypanosomosis have been eradicated, animal diseases other than trypanosomosis are still constraining livestock production in Unguja,

Zanzibar. The major diseases reported by the farmers interviewed in their order of importance are East Coast Fever (84%), Helminthiasis (34) and Lumpy Skin Disease (28). Others include Mastitis, Foot and Mouth Disease, Heart Water and Pneumonia as ranked by farmers in declining order of importance. The impact of these diseases is reduction of livestock productivity. For example 41% of the sample farmers interviewed reported that their cattle were affected and died from ECF.

The following are the possible interventions to reduce the impact of the diseases in order to increase and sustain livestock productivity in Zanzibar:

- Strengthening animal health services in the rural areas by involving the private sector in the provision of veterinary services together with AI services.
- Establishment of a revolving fund for small loans to private veterinarians to purchase basic facilities, veterinary drugs and vaccines.
- Promoting the use of botanicals for animal diseases treatment

(v) Although the proportion of improved cattle keepers practicing zero-grazing has increased from 87% in 1999 to 92% in 2002, most (77%) of the farmers practicing zero grazing still depend on natural grass as their major feed resource. Only 60% of the farmers used improved (planted) fodder such as Elephant grass, Guatemala and Glericidia. Also the results of the 1999 economic assessment survey and the follow-up survey of 2002 indicate that the proportion of farmers supplementing natural grass or improved fodder with concentrates has increased but the feeding regimes are still very low.

The International Atomic Energy Agency is currently promotion establishment of improved fodder in some villages. This should be extended to other villages in the island with emphasis on establishing mixtures of grasses and legumes to improve their nutritive value.

Other interventions which should go hand in hand with promotion of improved fodder establishment include the following:

- Farmer training on proper feeding management including the importance of supplementary (concentrates, mineral) feeding
- Low cost feed formulation
- Feed conservation techniques for use during the dry season when natural grass supply is inadequate

(vi) The results of the survey indicate that there is currently no serious problem of marketing milk in rural areas of Unguja island. However, the fact that the number of farmers raising improved cattle with a high potential for milk production has increased after tsetse intervention and that most farmers interviewed intend to increase milk production in the future suggest that milk output may increase above the levels that can be sold locally in the rural areas. Therefore it is necessary to develop a milk marketing system through which milk can be efficiently sold to distant markets especially urban areas where the demand for milk is high.

Interventions that can improve the marketing system may include the following:

- **Farmers organizations:** grass-root level farmers' or community-based organizations and networks must be promoted and strengthened to market milk in rural areas where private traders do not operate. They can also compete with private traders in areas where they are currently operating. The farmer organizations will not only improved milk marketing but also increase farmers' bargaining power with buyers of their milk as well as in purchasing inputs in bulky.
- **Farmer empowerment:** farmers should be empowered through knowledge to acquire the capacity to analyze their constraints, to identify opportunities and influence on their organizations.
- **Establishment of milk collection centres in rural areas:** Milk collection centres for bulking from individual farmers and sold to distant/urban markets by farmer organizations are necessary for improving the milk marketing system. Individual traders can also buy milk from the centres instead of collecting milk from scattered individual farmers.

(vii) The results of the market survey show that consumers especially tourist hotels prefer processed products not only because they are of high hygienic quality but also because they are assured of constant supply throughout the year. With regard to milk handling, most farmers use bottles and plastic containers for milking and marketing milk, a practice which is likely to lower the hygienic quality of milk because such containers are difficult to clean.

The following are some of the intervention geared towards improvement in the quality of milk to enable locally produced milk compete with imported milk which is currently perceived as of high quality standard by some consumers.

- Training on hygienic handling of milk including promoting use of aluminium containers for milking and marketing milk
- Processing: The large scale milk processing plant owned by the government has recently been sold to a private operator but it is still closed. There is need to promote small scale milk processing in rural areas. Promotion of milk processing will not only contribute to milk quality improvement but will also increase shelf life of the products and even the their supply.

5.0 Summary and conclusion

The primary purpose of this follow-up survey was to collect data on the current livestock and agriculture situation in Unguja, Zanzibar for assessing changes that have occurred since the last economic assessment survey in 1999. The approach adopted consisted of a review of the relevant recent documents on livestock and agriculture development in Zanzibar, collection of data on relevant parameters from these documents; informal discussions with key informants including government officials, design of a farm level survey using a formal questionnaire and design of a market level survey using check lists. The data collected were coded, entered in access database and analysed using the Stata software.

Based on the results of the 2002 follow-up survey and the results of the 1999 economic assessment survey, there is the general conclusion that the livestock and agriculture situation in Unguja, Zanzibar has improved since the eradication of tsetse and trypanosomosis in 1997. Whereas the 1999 economic assessment survey showed that the livestock and agriculture situation in Unguja has improved significantly from what it was before the initiation of tsetse/trypanosomosis control/eradication in 1985/86, the follow-up survey of 2002 indicate that livestock and agriculture production have continued to improved during the past three years (1999-2002). This suggests that eradication of tsetse and trypanosomosis has opened opportunities for increased livestock and agricultural production in Unguja, Zanzibar.

At the macro-level, improvement in livestock and agricultural production is evidenced by increased growth in livestock and agriculture. The contribution of agriculture to overall GDP increase from 34% in 1999 to 39% in 2001. The relative increase in the agricultural sector contribution to the overall GDP during the last three years is due to an increase in both crop and livestock production. Whereas production of crops such as paddy, cassava and bananas has respectively increased by 57%, 14% and 91%, production of milk, beef and chicken have increased by 6%, 7% and 8% respectively between 1999 and 2002.

At the farm (micro-) level, land areas cultivated for most crops in 2002 and 1999 do not vary significantly from those cultivated in 1999 although relatively more cultivated land in 2002 was allocated to the crops given top priority as cash crops and food crops. With the exception of coconut, cloves, rice and yams whose land areas declined, land areas under cassava, sweet potatoes, yams and maize increased slightly or remained the same while land areas for vegetables and plantain/banana increased substantially.

Whereas yields achieved by farmers in 2002 for cassava, rice, maize, coconut and vegetables increased from their 1999 levels, yields for bananas, sweet potatoes, yams and cloves declined from their 1999 levels. This suggests that productivity of cassava, rice, maize, coconut and vegetables has increased while that of bananas, sweet potatoes, yams and cloves has declined during the past three years. The increase in the productivity of cassava, rice, maize, coconut and vegetables may be attributed to the use of improved seed varfieties and manure for crop production. Although most of the farmers interviewed used local seed varieties for most of the crops during the 2002 cropping season, improved seeds were used for maize, rice, vegetables, fruits and coconuts. With regard to livestock, not only has the relative proportion of farms raising cattle and small ruminants increased in

2002 compared to 1999, the number of farms with improved cattle breeds has also increased. Evidence for increased intensification of livestock and agricultural activities in Unguja is provided by the increased crop-livestock integration through more farmers using manure for their crop production while in turn using crop by-products to feed their animals. The use of animal power for ploughing and transport activities has also increased during the past three years.

Although it is currently inappropriate to use criteria such as benefit-cost ratio to assess the impact of tsetse control/eradication, comparison of the performance of livestock and crop production before tsetse control/eradication with the post-tsetse (1997-2002) performance suggest that the eradication of tsetse and typanosomosis in 1997 has resulted in significant gains including increased milk production, manure and power for ploughing and transport activities. Of particular importance is the fact that some of the milk produced is consumed at home. On average, about 20% of the milk produced per farm is consumed at home as raw milk and about 1% is consumed as fermented milk. This suggests that the increase in milk production following tsetse and typanosomosis eradication has also contributed to improvement of the nutritional status of rural households in Unguja, Zanzibar. Another evidence of the positive socio-economic impact of tsetse and trypanosomosis eradication is the increase in the average household income and the proportion of farmers who moved from low-income to high-income strata since 1999. The average household income has increased by almost 30% from 41,232 TSHS in 1999 to 53,502 TSHS in 2002. Although it is difficult to isolate the effects of tsetse eradication from other factors since several factors may have contributed to the increase in household income, it is still logical to attribute the increase in household income to tsetse and trypanosomosis eradication. A strong correlation was observed between household income and milk yields, milk sales, use of manure and animal power for cultivation and transport. This correlation suggests that the increase in the household income between 1999 and 2002 is associated with tsetse eradication in the Unguja island.

Although no attempt is made in this report to use criteria such as benefit-cost ratio to assess the economic impact of tsetse eradication in Unguja, Zanzibar, the use of such criteria economic impact assessment in future would require accurate data on total population of important livestock species (cattle, goats, sheep and donkeys). Therefore a livestock census needs to be undertaken to obtain accurate information that will be used to assess economic impact.

Despite the observed increase in livestock production and productivity following eradication of tsetse and trypanosomosis, assessment of the aggregate production and demand for livestock products indicate that demand falls short of supply of livestock products such as milk. Comparison of current (2002) production as well as projections with current and future demand indicates that the domestic consumption needs are significantly higher than domestic supply. This suggests that Zanzibar will continue to experience a deficit of milk and will continue to rely on imports unless concerted efforts are made to increase domestic production. However, continued reliance on food imports cannot be sustained and could impact negatively to both livestock and agricultural production. Therefore deliberate efforts must be made by Zanzibar authorities in collaboration with private sector and other stakeholders to tackle constraints which are affecting the livestock and agricultural sector. These constraints include poor livestock and

crop extension services, processing and marketing of crop and livestock products, diseases and low usage of inputs and improved crop and livestock varieties. Although the IAEA has intervened by promoting a heifer in trust scheme and establishment of improved fodder, these efforts need to be extended to other villages in the island. Further interventions by the government and other parties interested in livestock development that would be required to improve and sustain livestock production include:

- Promoting the use of AI and bull services
- Strengthening animal health service in the rural areas by involving the private sector in the provision of veterinary services together with AI services.
- Establishment of a revolving fund for small loans to private veterinarian to purchase basic facilities, veterinary drugs and vaccines.
- Promoting the use of botanicals to treat some of the diseases
- Farmer training on proper feeding management including the importance of supplementary (concentrates, mineral) feeding
- Low cost feed formulation
- Feed conservation techniques for use during the dry season when natural grass supply is inadequate
- Promoting farmers' organizations
- Farmer empowerment.
- Establishment of milk collection centres in rural areas
- Training on hygienic handling of milk including promoting use of aluminium containers for milking and marketing milk
- Promotion of small scale milk processing in rural areas.

6.0 Documents Reviewed

Tambi, N. E; Maina, W.O and Mdoe, N.S.Y (1999). Livestock and Agriculture development in Zanzibar, Pre- and Post-Tsetse eradication. Report Prepared for IAEA, September 1999.

URT (United Republic of Tanzania). Zanzibar Statistical Database, 1980-2001. Ministry of Finance and Economic Affairs, Zanzibar, September 2002.

URT (United Republic of Tanzania) Statistical Abstracts. Department of Statistics, Ministry of State Planning and Investment, Zanzibar. 1997 and previous years.

RGZ (Revolutionary Government of Zanzibar), 1998. Five year post tsetse eradication development programme in Zanzibar. Ministry of Agriculture, Livestock and Natural Resources, Commission of Livestock and Agriculture, Zanzibar.

RGZ (Revolutionary Government of Zanzibar), 1999. Agriculture Sector Review. Report prepared by Food and Agriculture Organisation of the United Nations. April, 1999.

URT (United Republic of Tanzania), 1998. Five year post-tsetse eradication livestock development programme in Zanzibar. Agriculture and Livestock Commission, Zanzibar.

UNDP/FAO, 1993. Animal Disease Control Project, Zanzibar. The URT Project findings and recommendations. Rome, 25 pp.

WHO, (1999). World Health Organization, Division of Control of tropical Diseases (CTD). Internet Home Page of 12 June 1999. http://www.who.int/ctd/html/trypanoburtre.html

Wizara ya Kilimo, Mifugo na Mali Asili (2000) Ripoti ya Mwaka 1998. February 2000.

Wizara ya Kilimo, Mifugo na Mali Asili (2001) Ripoti ya Mwaka 1998. February 2001.

Wizara ya Kilimo, Mifugo na Mali Asili (2001) Ripoti ya Mwaka 1998. February 2002.

ANNEX 1

Table A 1.1 Names of villages sampled

North A	North B	Central	South	Western
Moga	Muwanda	Kisomanga	MuyuniA	Mbuzini
Mkokotoni	DongeKipange	Ndijani	Kibuteni	Mwakaje
Pale	Donge Mtambile	Umbuji	Kitogani	Dole
	Kitope	Bambi	Muyuni B	Fuoni
	Kilombero	Ghana	Muyuni C	Kizimbani
	Mahonda	Koani*	Makunduchi	Mfenesini
	Mangapwani	Dunga*	Pete	Kianga
	Zingwezinge	Mchangani	Muungoni	Kimara
	Fujoni	Tunduni		Mwachealale
	Kiomba mvua	Kikungwi		Bumbwi sudi
	Mkadini	U/Kae Pwani		Kama*
		Bungi		Michungwa miwili*
		U/Kaebona		
		Charawe		
		Cheju		
		Jendele		
		Pagali		
		Mgeni Haji		
		Kiboje		
		Machui		
		Ubago*		

* New villages which were not sampled in 1999 Economic Assessment Survey

ANNEX 2

QUESTIONNAIRE FOR THE ECONOMIC IMPACT ASSESSMENT OF TSETSE CONTROL AND ERADICATION IN ZANZIBAR ISLAND: FOLLOW-UP SURVEY 2002

QUESTIONNAIRE NUMBER _______________________ DATE____________________

DISTRICT____VILLAGE_________________

FARMER NAME /ID (Optional) ____

NAME OF ENUMERATOR ____

Household Characteristics

1. Sex: Male________________ Female_______________ 2. Age___________________

3. Religion: Muslim__________ Christian______________ Other (specify) ____________

4. Ethnic group:_____________ 5. Length of stay in this village ______________________

6. Education: None__________ Adult education_________ Primary _________________
Secondary______ High School ___________ University________________

7. Household Size and Composition: Children below 12 years ________________________

Children 12-18 years____________ Adults: Males__________ Females ________________

Total household size____

8. Major occupation: Farming________ Business________ Wage employment ___________

Other (specify)____

9. Secondary occupation: Farming ________ Business _______ Wageemployment ________

Other (specify)____

10. Household income (Tshs per month) ____

Livestock

11. Indicate the type and number of livestock you have been raising from when you started and now.

Type of Livestock	No. of animals you had three years ago (1999)	No. of animals you have now	No. you intend to have in the future	Reasons for the increase/decrease in numbers
Indigenous cattle				
Cross-breed cattle				
Pure breed cattle				
Local sheep & Goats				
Improved sheep & goats				
Local poultry				
Improved poultry				
Donkeys				
Rabbits				
Others (specify)				

12 Indicate whether the following improved breeds are more readily available now than 1999.

	Availability			Source	Approximate price per head
	More now	less now	No difference		
Pure breed cattle					
Cross breed cattle					
Improved sheep and goats					
Improved poultry					
Other (specify)					

13. If you were to choose between raising local breeds of animals and improved breeds which one would you prefer? Local breeds _____ Reasons ____
____
Improved breeds _____ Reasons ____
____

14. Have you ever wanted to have improved breeds but did not have access to them?
Yes ____ No _____ If yes give reasons ____
____

15. Has the availability of improved livestock species increased _______ decreased _____ or remained the same _______ since 1999?

16. Indicate the current structure and composition of the cattle herd

**Category**	**Number in 1999**	**Number now**
Cows (dry and in milk)		
Bulls (mature males)		
Heifers (females > 2 years old not yet calved)		
Steers (males 2 to 4 years old		
Oxen (males > 4 years old)		
Female calves (< 2 years old)		
Male calves (< 2 years old)		
**Total herd size**		

17. List in order of importance the diseases that affect your animals: 1) ___________________
2) ______________________ 3) ________________________ 4) ________________________

18. Are there any animal diseases of importance that were present in 1999 that are no longer present today?
Yes ____________________ No _______________________
If yes list them 1) ____________________ 2) ___________________ 3) ___________________

19. Do you experience any animal diseases today that were not present in 1999? Yes
_________________________________ No _________________________________
If yes list them 1) ____________________ 2) ___________________ 3) ___________________

20. Have your animals suffered from trypanosomosis since 1999? Yes __________ No _________
If yes, when did you experience this problem? ________________

21 Please provide the following information if your animals suffered from trypanosomosis after 1999.

**Type of animal**	**Number of animals in that particular year:**		
	**Total number available**	**Number affected by trypanosomosis**	**Number that died from trypanosomosis**
Indigenous cattle			
Crossbreed cattle			
Pure breed cattle			
Sheep and Goats			

Donkeys			
Others (specify)			

22. Have you ever attempted to prevent or treat your animal(s) against trypanosomosis since 1999?
Yes _________________________________ No ____________________________________

If yes which of the following methods did you use for prevention 1) bush clearing __________ 2) use of traps ___________________ 3) use of chemical products _____________________ 4) moving away from infested areas _________ 5) keeping trypanotolerant animals ________ 6) keeping other animal species _____________ other (specify) ________________________

Which of the following methods did you use for treatment 1) Trypanocidal drugs __________ 2) Ethnoveterinary products _________________ 3) Other (specify) ___________________

23. About how much money did you spend to 1) Prevent trypanosomosis _______________
2) Treat against trypanosomosis ____

24. Indicate number of animals which died from a major disease other than trypanosomosis in the past three years.

**Type of animal**	**Disease**	**Number affected**	**Number that died**
Indigenous cattle			
Crossbreed cattle			
Pure breed cattle			
Sheep and Goats			
Donkeys			
Others (specify)			

25. Please provide information about control measures and costs incurred in controlling diseases other than trypanosomosis during the past three years

Disease	Preventive measure/treatment	Amount of money spent
ECF		
Mastitis		
Helms		
Others		

26. Has the number of animals you raise changed since 1999? Increased from _______ to ________
Decreased from __________ to ______________
Remained the same at ____
Give reasons for your answer ____
____

27. Indicate whether as a result of the eradication of tsetse you intend to Increase ______ reduce _______ or continue to have the same ______ number of animals ____________ in the future. Give reasons for each
____
____

Dairy production
28. Has the number of dairy cattle enterprise increased since 1999? Yes ______ No. ________

29. Indicate the following about milk production, sales and consumption.

	**No. of dairy**	**Total milk production**	**Quantity fed to**	**Quantity consumed**	**Quantity sold**	**Quantity given away as**

	animals		calves	at home		gifts
In 1999						
Now (2002)						
In future						

30. What are the reasons for the increase or decrease in the number of dairy animals you now have
____
_
____
_

31. Are improved dairy cattle readily available? Yes ____________ No ________________
If yes from where are they acquired _______________________ and what is the approximate price per head
____

32. Indicate which of the following breeding methods is/are used for the dairy herd

Breeding system	Source	Cost per service
Artificial insemination		
Local bulls		
Improved bulls		

Please provide the following information

	Cow 1	Cow 2	Cow 3	Cow 4
33. How old was this cow when it had the first calf?				
34. After having the first calf how many months did it take to have a second calf?				
35. How many calves since the first calf has the cow had?				
36. After having a calf how many months did the cow continue to produce milk?				
37. During this period what was the average quantity of milk per day it produced during the **Dry season?**				
**Rainy season?**				
38. Has the cow had any abortion?				
39. If yes in (39) how many times has it aborted during its life				

40. Please provide the following information about dairy animals if they have been affected by trypanosomosis since 1999.

Type of animal	Number of animals in that particular year:		
	Total number available	Number affected by trypanosomosis	Number that died from trypanosomosis
Indigenous dairy cattle			
Crossbreed dairy cattle			
Pure breed dairy cattle			
Dairy goats			

41. Indicate which of the following types of feeding system(s) is/are used for dairy animals

1) Zero grazing ___________ 2) semi zero grazing _____________ free grazing _____________

42. What feed materials are used for feeding dairy animals 1) _______________ 2) __________ 3) ________________________ 4) ________________________ 5) ______________________

43 Are there any feed materials used now that were not used in 1999? Yes ______ No __________
If yes, list them 1) ___________ 2) ___________________ 3) ________________________

44. Indicate source, type of feeding materials and costs associated with different feed resources for your dairy animals

**Feed materials**	**Source**	**Availability**	**F**
Collected from the forest/bush<br>Grasses<br>Legumes<br>Others	---------------<br>------------------------------	---------------<br>------------------------------	--<br>--
Home grown<br>Elephant grasses<br>Glericidia<br>Others	---------------<br>------------------------------	---------------<br>------------------------------	--<br>--
Purchased (specify where purchased)<br>Bran<br>Cakes<br>Pollads<br>Others (specify)-<br>----------------------------------<br>----------------------------------	---------------<br>------------------------------<br>------------------------------<br>---------------	---------------<br>---	--<br>--

45. Do you use animal manure for crop production? Yes ________________ No __________
If yes how long have you been using animal manure ___________________________________
If you are not using animal manure do you intend to use it in the future Yes _____ No ______

46. Indicate the following information about the collection and use of manure.

	**Quantity used for crops presently**	**Quantity used for pasture improvement presently**	**Quantity given away (as gifts etc) presently**	**Present quantity sold and Price**		**Quantity for other uses (e.g. Biogas production) at the present**
				**Quantity**	**Price**	
Cattle						
Sheep & Goats						
Poultry						

47. Do you practise rotational grazing? Yes ___________________ No _________________
If yes do you let the animals fertilize plots/paddocks? Yes ____________ No _____________

If yes do you grow crops on the fertilized areas Yes ________________ No ____________

48. Do you use any of your farm animals for traction? Yes____________ No____________
If yes how long have you been using animal traction _________________________________ If you are not using animals for traction do you intend to do so in the future Yes ___ No ___

49. Indicate the number of acres ploughed in your own farm using animal traction ________

50. Do you rent any of your farm animals for traction? Yes___________ No_____________
If yes indicate the price per day per animal _________ or price per acre per day___________

51. Which of the following do you use any of your farm animals for:
1) Own transport ___________ 2) Commercial transport ___________ (if used for commercial purposes, please give approximate amount of money earned per month ________

52. How long have you been using farm animals for own transport _____________________
commercial transport ________________ If you are not using farm animals for transport do you intend to use them in the future ? Yes _____________________ No _______________

53. Have any of the animals you use for animal power suffered from trypanosomosis since 1999?
Yes ______ No ______ If yes what did you lose as a result of their being sick? ____________________
____

Milk marketing and processing

54. Provide the following information about the use of milk produced in your farm.

Use	Quantity in local units (e.g. bottle) per day		Quantity in litres per day	
	Wet season	Dry season	Wet season	Dry season
Calf feeding				
Consumed as raw milk at home				
Fermented and consumed at home				
Sold as fresh milk				
Sold as fermented milk				
Given away				
Wasted				

55. Are you able to sell all surplus milk produced in your farm all year round? Yes _____No. _____
If no, in what particular season/month do you face problems of milk disposal? Season/month ___________

56. What do you do with the milk, which could not be sold? ___________________________________
57. Do you normally deliver to the buyer or the buyer collects? __________________________________

58. What are your main milk handling containers during marketing?
____

59. Provide the following information about your market outlets for raw milk.

	Wet season			Dry season		
Market outlet	Quantity sold/day	Distance from home	Price per unit (specify unit of measure e.g. bottle or litre)	Quantity sold/day	Distance from home	Price per unit (specify unit of measure e.g. bottle or litre)
Household consumers						
Hawker						
Vendor						

Local market						
Kiosk/retail shop						
Collection centre/Farmer organization						
Processor						
Hotels/restaurants						
Institutions (eg. Schools)						
Others (specify)						

60. Which market outlet do you prefer most and why? ________________________________
____

61. If you deliver milk to your customers away from home, what means of transport do you use?
____

62. Do you process milk? Yes _____ No. _____________
If yes, what products do you make? Sour milk ______ Yoghurt ______ Ghee ____ Butter ___ Others (specify) _______________________

63. If you process milk, provide the following information about processed products

Milk product	Made in which season? (Wet, dry or both?)	Market outlet or customer	Quantity sold per day	Price per unit (specify unit)
Sour milk				
Yoghurt				
Ghee				
Butter				
Others (specify)				

Crop production

64. Indicate the four most important food crops: 1)_______________ 2)________________
3) ______________________________ 4) _______________________________________

65. Indicate the four most important cash crops: 1) _______________ 2) _______________
3) ______________________________ 4) ____

66. Indicate the crops for which you use improved seed varieties 1) ____________________
2) ___________________ 3) _________________________ 4) _______________________

67. Are improved seeds readily available now than three years ago? Yes ________________ No
____
If yes, indicate the source and approximate prices per kg. of improved seed used for major crops

Crop	Source of improved seed	Cost per kg. of seed purchased
Rice		
Maize		
Cloves		
Coconut		
Legumes		
Cassava		
Others		

68. Types of crops, acreage cultivated and total production

**Type of crop**	**Acreage**	**Total**	**Acreage**	**Total**	**Price**	**Acreage**	**Production in the**

	cultivated in 1999/00	production in 1999/00	cultivated now	production now	per unit now	in the future	future
Cassava							
Rice							
Maize							
Banana/ Plantain							
Sorghum							
Sweet potatoes							
Yams							
Cloves							
Coconut							
Tania							
Vegetables							
Others							

69. Has the land area you cultivate increased since 1999? __________, decreased __________ or remained the same in the last three years (since 1999)? Give reasons for the increase or decrease ________
____

70. Do you intend to increase _________, decrease __________ or maintain the same land areas you cultivate in the future? Give reasons for your decision ______________________
____

ANNEX 3

Table A 3.1. Enumerators used during the survey.

No.	Enumerator name	Qualification	Year of experience
1	Abdulla Moh'd abdulla	Certificate in Animal health & production	19
2	Moh'd Ali Ameir	Diploma in Range Management	11
3	Fatma w. suleiman	Diploma in Animal Health	9
4	Maryam R. Mzee	Certificate in Animal health & Production	11
5	Abubakar Hassan Kisoma	Diploma in Animal Production	14
6	Silima Hassan	Diploma in Crop Production	22
7	Salum Mwinyi Rehani	Diploma in Crop Production	14
8	Mussa Salum Abdulla	Diploma in Crop Production	7
9	Juma Omar	Diploma in Lab. tech. Chemistry	22
10	Abdul Rahim Hamid	Diploma in Animal Production	22
11	Said Kassim Yussuf	Certificate in AI. ,& Animal Health	25
12	Abeid Khamis Ramad'n	Diploma in Animal Health	21
13	Faki Kessi	Diploma in Animal Health	7
14	Ishaka Abdul Wakil	Msc. Agronomy	18
15	Remi Abdulla Bachoo	Diploma in Poultry Production	
16	Ussi Ameir	Diploma in Dairy Production	
	**Name of field supervisor**	**Qualification**	
1	Khalfan M. Saleh	Diploma in Ecology	15
2	Dr.Waridi Abdulla Mussa	MSc. in Veterinary Medicine	16

ANNEX 4

Instructions and explanations to enumerators accompanying questionnaire for the economic impact assessment of tsetse control and eradication in Zanzibar Island: Follow-up socio-economic survey 2002

Prior to completing each questionnaire, the enumerator must establish whether the person being interviewed is the household head or the major decision maker. If none of these is available the questionnaire should not be completed for that household. The enumerator is expected to have properly understood each question in the questionnaire.

Upon meeting the farmer the enumerator should explain the purpose of the survey and request the farmer's permission to have a discussion with him/her. Seek for his/her patience and cooperation during the exercise. Do not read each question to the farmer. Rather, endeavor to have a friendly, yet constructive dialogue with the farmer while tactively trying to fill in the responses. Do not make any promises to the farmer. During the interview if there are any answers that do no respond adequately to the question(s) being asked, probe further until you establish that the question has been adequately responded to. If the farmer does not seem to understand the question as it is, devise an alternative way of phrasing the question such that the message gets to the farmer.

The first ten questions pertain to the household head or the household decision maker. They seek to obtain general information on household characteristics and are quite straight forward.

Questions 11 to 63 deal with the livestock enterprise as a whole. These questions seek to establish the status of livestock development at the farm level from when the farmer began livestock production to the present. They also seek to explore the future potential of livestock development with particular emphasis on the use of improved livestock genetic resources, especially cattle, small ruminants and poultry. As well, they seek to establish livestock productivity parameters, animal disease situation with emphasis on tsetse and trypanosomosis, feeding and management practices.

Questions 11 is intended to explore changes in livestock numbers since the last socio-economic survey in 1999 and reasons for the changes. The enumerator should complete the table in **question 11** paying particular attention to the numbers of each livestock species in 1999 and the current situation. The enumerator should use his/her personal judgement to determine whether what the farmer intends to have in the future is realistic. It should be guided by his/her present resources.

Questions 12 to 15 seek to establish the changes in availability, sources, costs and the use of improved livestock species that have occurred since 1999.

Questions 16 is intended to explore changes in the cattle herd structure since the last socio-economic survey in 1999.

Questions 17 to 21 seek information about important livestock diseases constraining livestock development in Zanzibar as there might be livestock species that have been affected since the last survey in 1999. Questions 17 to 19 attempt to explore changes in the economically important major livestock diseases experienced by farmers since the last survey in 1999. **Questions 20 to 23** specifically seek to establish the status of trypanosomosis, its effect on livestock, control methods undertaken and expenditure on diseases control since the last survey. **Questions 24 to 25** seek to obtain information about cattle deaths caused by diseases other than trypanosomosis and costs of their control.

Questions 28 to 44 are specific to the dairy enterprise because dairy production offers more possibilities for increased intensification and therefore contributing to food security. These questions seek to establish the changes in productivity of dairy animals since the last survey in 1999. They also seek to establish the implications of tsetse eradication on the current status of dairy production and the future potential for development. For **question 29** the enumerator should establish the type and size of containers used by the farmer for measuring milk. For question **33** the enumerator should specify the age of the cow during its first calving in years or months which ever is applicable. For **question 42** the enumerator should establish that the farmer is using the feed material on a regular basis and not just once or occasionally. **Question 44** seeks detailed information about quantities and costs of different feed materials used. In this question, the

enumerator should clearly indicate the units of measure for different feed resources. For example the farmer may be paying someone collecting grass from the bush/forest in terms of "mzigo" or bale. Attempts can be made to convert this into kg. of green grass. Cost of home grown feed (established fodder) can be expressed in terms of TShs. per acre or hectare and this should include establishment costs together with husbandry costs including harvesting and transportation costs from the farm to the homestead.

Questions 45 to 53 are meant to establish crop-livestock interactions through the use of manure for crop production and pasture improvement, use of crop by-products for animal feed and the use of animal power. To complete the table in **question 46**, the enumerator should first of all establish whether the manure is used in fresh form of dry. Then ask for the type of containers used by the farmer, make an assessment of its weight when filled with manure and find out the number of fully filled containers used for each purpose

Questions 54 to 63 seek information about use of milk, milk marketing outlets and processing of milk at the household level. For **questions 54, 59 and 63** the enumerator should attempt to capture seasonal differences (wet and dry season) and type and size of containers used by the farmer for measuring milk sold to different customers. The units of measure used by different farmers should be clearly indicated when filling the questionnaire.

Questions 64 to 70 deal with crop production and seek to establish changes in crop production that have occurred since the past survey in 1999 and future potential of the crop production system at the farm level. They also attempt to establish the implications of tsetse eradication on current crop production and the future potential. Allusion is made to the fact that as tsetse become eradicated, increased intensification will call for the use of the improved varieties. Therefore it is necessary to establish farmers' access to and use of improved plant genetic material in **questions 66 and 67**. For question 68 the enumerator should first of all establish the type and size of container used by the farmer for measuring the output from each crop. The enumerator should also use his/her judgement to establish the size of the plot by physical inspection where possible. It should be stated whether the plot size is in acres or hectares. **Questions 69 and 70** are intended to explore the extent to which farmers are increasingly and will continue to take advantage of the tsetse cleared areas for crop production.

ANNEX 5

CHECK LISTS/QUESTIONNAIRES FOR THE MARKET LEVEL SURVEYS

A 5.1. QUESTIONNAIRE FOR HOTELS AND RESTAURANTS

Name of Hotel/Restaurant (Tick one)………………………………………….
Location: ……………… Region: ………………..District: ………………….
Capacity (total # of visitors): ……………………….. Date: ………………

Milk Products

1. Sources of milk /milk products;

(i) INTERNAL (Locally)
(a) From individual farmers (retailers): ….. (b) From big farm (private):…….
(c) Government farm ….. (d) Own farm: ……. (e) Others (specify):……..

(ii) EXTERNAL (Imported)
(a) From Europe …. (b) East and Central Africa….. (c) South Africa …..
(d) Middle East …. (e) America ….. (f) Others (specify) ……

2. Price per unit; (Tshs or US$)
(i) From internal sources (price/litre, kg) …………… (Tshs)
(ii) From external sources (price/litre, kg) …………... (US$) = ……. (Tshs)

3. Type(s) of milk products sold;
(a) Imported;
(i) Fresh …. (ii) Powder….(iii) Yoghurts ….(iv) Condensed …. (v) Cheese ….
(vi) Others (specify) ….
(b) Locally;
(i) Fresh (boiled) …. (ii) Yoghurts …. (iii) Others (specify) ….

4. How is milk consumed;
(i) In tea …. (ii) Fresh (Boiled) …. (iii) Yoghurts ….
(v) Mixed with other foods (fruits etc) …. (vi) Others (specify) ….

5. How many visitors per season does your hotel serve? ………visitors

6. Quantities of milk consumed per day;

	Types and quantities (lit, kg)					
Local	Fresh …….	Yoghurts ……..	-	-	-	
Imported	Fresh …….	Yoghurts……..	Powder ……..	Condensed ……..	Cheese ……..	Others ……..<br>(Specify)

A 5.2. QUESTIONNAIRE FOR KIOSKS AND MILK BARS

Name (if any):…………………………….……………. (Kiosk/Bar) (Tick one)
Location: ……………… Region: ………………. District: …………. Date:……………

Milk Products

1. Sources of milk/milk products;

(i) INTERNAL (Locally)
(a) From individual farmers (retailers) …….. (b) From a big farm (private)…….
(c) Government farm ……. (d) Own farm ……. (e) Others (specify) ………..

(ii) EXTERNAL (Imported)
(a) From Europe …… (b) East and Central Africa …… (c) South Africa ……
(d) Middle East …….. (e) America ……. (f) Other (specify) …………………

2. Quantity of milk/yoghurts sold per day (average); …………………. (litres)

3. Price per unit (Tshs);

(i) From internal sources ………………….. (Tsh/litre, kg)
(ii) From external sources (price/litre, kg) = US$ …….. … = ……… . Tshs.

4. Type(s) of milk/milk products sold;

(a) Imported;
(i) Fresh … (ii) Powder … (iii) Yoghurts … (iv) Condensed … (v) Others (specify) …
(b) Locally;
(i) Fresh (boiled) … (ii) Yoghurts … (iii) Others (specify) …

5. How is milk consumed;

(i) In tea … (ii) Boiled (fresh) … (iii) Yoghurts …
(iv) Mixed with other foods (fruits etc) … (vi) Others (specify) …

6. How do you feel about milk business?

(i) Very profitable ….. (ii) Profitable …… (iii) Less profitable ……

A 5.3. QUESTIONNAIRE FOR HOUSEHOLD CONSUMERS

Name of the respondent:…………………………………………………………………..
Location: ……………… Region: ………………………… District: ………………….
Sex: (Tick one) (i) Male: …… (ii) Female: …… Age: ……(years)

Milk and milk products

1. Source of products;

Imported;
(i) From shops … (ii) Supermarkets … (iii) Restaurants … (iv) Kiosks …
(v) Others (specify) …

Locally;
(i) Retail middlemen … (ii) Direct from the farms … (iii) From Kiosks …
(iv) From shops … (v) Restaurant … (iv) Other (specify) …

2. Availability of the products; (tick)

**Dry season**	**Available**	**No difference**	**Deficit**
Imported			
Locally			

Wet Season	Available	No difference	Deficit
Imported			
Locally			

4. Average quantities consumed per day;

**Quantities consumed/day (in lt or kg) during dry season**						
Imported	Fresh ……	Yoghurts…..	Cheese….	Powder….	Condensed….	Others (specify)...
Locally	Fresh ……	Yoghurts…..	-	-	-	
**Quantities consumed/day (in lt or kg) during wet season**						
Imported	Fresh …..	Yoghurts….	Cheese….	Powder….	Condensed …	Others (specify)...
Locally	Fresh …..	Yoghurts…..	-	-	-	

5. Prices of dairy products in dry and wet seasons;

**Prices for dairy products in Tshs during dry season**						
Imported	Fresh…..	Yoghurts…..	Cheese….	Powder….	Condensed …	Others (specify)
Locally	Fresh…..	Yoghurts….	-	-	-	
**Prices for dairy products in (Tshs) during dry season**						
Imported	Fresh…..	Yoghurts….	Cheese….	Powder….	Condensed	Others (specify)
Locally	Fresh…..	Yoghurts….	-	-	-	

6. Tastes and preferences;

Local products;
(a) Highly preferred …….. (b) Moderately preferred …… (c) Less preferred ……

Imported products;
(a) Highly preferred …….. (b) Moderately preferred …… (c) Less preferred ……

Raw products;
(a) Highly preferred …….. (b) Moderately preferred …… (c) Less preferred ……

Processed;
(a) Highly preferred …….. (b) Moderately preferred …… (c) Less preferred ……

7. Form(s) in which milk/milk products are consumed;

(a) Fresh (boiled milk) …………. (b) In tea ………… (c) Yoghurts ………….
(d) Mixed with other foods (e.g. fruits) ………. (e) Others (specify) ………….

ANNEX 6

Table A6.1. Population of Zanzibar by Region in 2002

Region	Population	growth rate
South Unguja	94502	2.1
North Unguja	136953	2.5
Urban West	391002	4.5
North Pemba	186013	2.2
South Pemba	176153	2.3
Total znz	984625	3.1
TB	33584607	2.9
Tz	34569232	2.9

Source: Population Census, 2002

ANNEX 7

Table A 7.1. Imports of milk and milk products in Zanzibar, 2000-2002

Dairy product	Source	Quantity	Dairy product	Source	Quantity	Dairy product	Source	Quantity
Nido milk	Holland	36,325 bags	Milk powder	Switzerland	3,506 catoons	Milk powder	Holland	6,772ba[illegible]
Sona Milk	U.A.E	4,708 catoons	Milk powder	U.A.E	6,896 catoons	Milk powder	Indonesia	11,973 catoon[illegible]
Cream milk	Switzerland	2,280 catoons	Nido milk powder	U.A.E	60 catoons	Nido milk powder	Switzerland	41,075 catoon[illegible]
Nido milk powder	U.A.E	5boxes	Evaporated milk	Switzerland	11,448 catoons	Evaporated milk	U.S.A	4,416 catoon[illegible]
U.H.T.liquid milk	U.A.E	5boxes	Tinned milk	U.A.E	155 catoons	Tinned milk	U.A.E	120 Pkt[illegible]
Nido milk powder	South Africa	6,048catoons	Supper milk	U.A.E	1,290bags	Supper milk	U.A.E	1,550cato[illegible] s
Milk powder boxes	U.A.E	60 catoons	Zain milk	U.A.E	11,138 boxes	Zain milk	U.A.E	50 catoo[illegible]
Evaporated milk box	U.A.E	25 catoons	Infant milk	U.S.A	50boxes	Infant milk	South Africa	38,040 catoon[illegible]
Conserned milk	U.A.E	50 catoons	Liquid milk	Switzerland	1048 catoons	Liquid milk	U.A.E	200 catoo[illegible]
Evaporated milk	U.A.E	130 catoons	Sona milk	U.S.A	177 catoons	Sona milk	U.A.E	80 catoo[illegible]
Milk powder bags	U.A.E	100 boxes	Cream milk	Nertheland	5724bags	Cream milk	U.A.E	20 catoo[illegible]
Luna milk	U.A.E	15 catoons	Baby milk powder	U.A.E	100 catoons	Baby milk powder	U.A.E	7 catoo[illegible]
Coconut milk	U.A.E	100catoons	Fresh milk	U.A.E	10boxes	Fresh milk	U.A.E	150 catoo[illegible]
			Lund milk			Lund milk	U.A.E	125 catoo[illegible]

ANNEX 8.

Terms of Reference for Second Socio-economic Survey in Zanzibar

The primary purpose of your mission was to assess the changes in the livestock sector which have occurred since the last study to get a measure for the impact of the tsetse eradication. In order for greater benefit to be derived from the work particular attention was paid to the dairy sector on Unguja, past, present and future potential for development. This included:

- An analysis of the current market for dairy products on Unguja and the potential development of a local market for dairy products taking into account the situation of the dairy sector in East Africa.

- Evaluation the potential for the development of the dairy sector on Unguja taking into account the economics and availability of feed resources and their availability on the island and on the mainland;

- Identification of other constraints for the development of the dairy sector;

- Making an estimate on the impact of disease control on the dairy sector and assessing its sustainability.
