# Supplementary material for: Sterile Insects to Enhance Agricultural Development: The Case of Sustainable Tsetse Eradication on Unguja Island, Zanzibar, Using an Area-Wide Integrated Pest Management Approach
Source: PLoS Negl Trop Dis. 2014 May 29;8(5):e2857. doi: 10.1371/journal.pntd.0002857 (PMC4038469; doi:10.1371/journal.pntd.0002857)
Supplement: Supporting Information S3 — Livestock and agriculture development in Zanzibar, pre- and post- tsetse eradication. International Atomic Energy Agency confidential report by N. E. Tambi, W. O. Maina, and S. Y. N. Mdoe, in the framework of project RAF5040, Vienna, December 1999. (PDF) [file pntd.0002857.s003.pdf]

LIVESTOCK AND AGRICULTURE DEVELOPMENT IN ZANZIBAR, PRE- AND POST-TSETSE ERADICATION

PROJECT: RAF 5 040

Report prepared for IAEA by:

Tambi, N. Emmanuel,[1] Maina, W. Onesmus[1] and Mdoe, S.Y. Ntengua[2]

[1] **OAU/IBAR-ILRI, P.O. Box 30709 Nairobi, Kenya.**
[2] **Department of Agricultural Economics, Sokoine University, Morogoro, Tanzania.**

December, 1999

Table of contents

		Page
**Table of contents**		2
**List of Tables**		6
**List of Figures**		9
**Summary**		10
**1.0**	**Introduction**	12
1.1	Objectives	13
**2.0**	**Methodology**	14
2.1	Study Area	14
2.2	Field Visit	15
2.3	Sample Design	15
2.4	Development of the questionnaire	15
2.5	Questionnaire administration	16
2.6	Data compilation and analysis	17
**3.0**	**Results**	18
3.1	Household socio-economic characteristics	18
3.1.1	Age and sex distribution	18
3.1.2	Education and religious affiliation	19
3.1.3	Household size and structure	20
3.1.4	Occupational distribution	20
3.1.5	Household income	21
3.2	Livestock and agriculture development in Ugunja, pre-tsetse control/eradication, 1985/86	22
3.2.1.	Livestock production	22
3.2.1.1.	Livestock production systems	22
3.2.1.2.	Livestock numbers	22
3.2.1.3.	Livestock farms	23
3.2.1.4.	Livestock species raised and their production and reproduction parameters	25
3.2.1.4.1	Cattle	25

3.2.1.4.1	Cattle herd structure	26
3.2.1.4.2	Small ruminants	26
3.2.1.4.3	Poultry	27
3.2.1.4.4	Donkeys	27
3.2.1.5	Dairy production, processing and marketing	27
3.2.1.6	Livestock disease situation	28
3.2.1.6.1	Tsetse and Trypanosomosis in Unguja	28
3.2.1.6.2	Tsetse and typanosomosis control measures	30
3.2.1.6	Tsetse and trypanosomosis eradication using the SIT in Unguja	32
3.2.1.6.4	Farmer awareness of typanosomosis	33
3.2.1.6.4.1	Impact on cattle production	34
3.2.1.6.4.2	Impact on milk production	36
3.2.2	Crop production	38
3:2.2.1	Crop production systems	38
3.2.2.1.1	Tree crop production	38
3.2.2.1.2.	Food crop production	38
3.3.3.	Exports and imports of livestock and agricultural products	41
3.2	Livestock and agriculture development in Ugunja, post-tsetse eradication, 1999.	43
3.3.1	Livestock production	43
3.3.1.1	Livestock production systems	43
3.3.1.2	Livestock species raised	44
3.3.1.2.1	Cattle	44
3.3.1.2.2	Sheep and goats	45
3.3.1.2.3	Chickens	45
3.3.1.2.4	Donkeys and rabbits	46
3.3.1.3	Dairy Production	46
3.3.1.3.1	Dairy production with indigenous cattle	46
3.3.1.3.2	Dairy production with improved cattle	47
3.3.2	Crop production	48
3.3.2.1	Types of crops produced	48
3.3.2.2	Acreage cultivated	49
3.3.2.3	Quantities of crops harvested and yields	50
3.3.3	Implications for agricultural development and marketing	51

3.3.3.1. Intensification of livestock and crop production 53
3.3.3.1.1. Adoption of improved livestock breeds and crop varieties 54
3.3.3.1.2 Access to land resources 56
3.3.3.1.3 Integration of crops and livestock 57
3.3.3.1.4 Adoption of animal power 58
3.3.3.1.5 Dairy intensification 59
3.3.3.1.6. Processing and marketing of milk and dairy products 61

3.3.4 Predictive scenarios for livestock and agricultural development through 2005, 2010 and 2015 63
3.3.4.1 Meat 63
3.3.4.2 Milk 65
3.3.4.3 Eggs 66
3.3.4.4 Projections for livestock production systems 67
3.3.4.5 Implications of the projections in livestock products 67
3.3.4.6 Crop production projections, 1999 to 2015 67

3.3. Issues requiring attention by the Zanzibar authorities and other parties 69

4.0 Framework for economic impact assessment of tsetse control/eradication 73

4.1. Why a framework for impact assessment 73
4.2. Previous work 73
4.3. Important considerations for developing a framework 77
4.3.1. Direct losses 77
4.3.2. Indirect losses 77
4.3.3. Direct benefits 78
4.3.3.1. Mortality losses avoided 78
4.3.3.2. Morbidity losses avoided 78
4.3.3.3. Control and treatment costs saved 78
4.3.4. Indirect benefits 79
4.3.4.1. Constraints on livestock production eliminated 79
4.3.4.2. Constraints on agricultural production eliminated 79
4.3.4.3. Constraints on livestock-crop integration 80
4.4. Application of the framework to economic analysis 80
4.4.1. Data needs for estimating mortality losses 81
4.4.2. Data needs for estimating morbidity losses 81
4.4.3. Data needs for estimating control/eradication costs 82
4.4.4. Data needs for estimating indirect losses on livestock and

agricultural production. 82
4.4.5. Other data needs 83
4.4.6. Calculations required 83
4.4.6.1. Mortality and morbidity losses 83
4.4.6.2. Economic value of livestock and agriculture production under conditions of trypanosomosis 85
4.4.6.3. Decision criteria 86

5.0. Summary and conclusion 87

References 89

Annexes 92

List of Tables

		Page
Table 1.	Sex and Age distribution of household heads	19
Table 2.	Educational level and religious affiliation of household heads	19
Table 3.	Household structure by age and sex category	20
Table 4.	Distribution of household heads by occupation	21
Table 5.	Average household income and frequency distribution of household income	21
Table 6.	Results of thė cattle census Unguja Island in 1993	23
Table 7.	Total number of cattle by type and district in Unguja 1993	23
Table 8.	Farmers raising livestock in Unguja, Zanzibar, before tsetse intervention in 1985/86	24
Table 9.	Average herd and flock sizes for cattle, sheep, goats and poultry on Unguja Island 1985/86	24
Table 10.	Calving and mortality rates at the Bambi dairy farms, 1977-1985	25
Table 11.	Milk production at farm level in Unguja, 1985/86	26
Table 12.	Milk and milk products produced by the Zanzibar Dairy Corporation, 1977-1985	27
Table 13.	Milk sales from indigenous cattle in Unguja 1985/86	28
Table 14.	Prevalence of cattle typanosomosis in Unguja Island, Zanzibar, March 1987 before the initiation of the Animal Disease Control Project	30
Table 15.	Prevalence of cattle typanosomosis in Unguja, Zanzibar, 1982 to 1984	31
Table 16.	Farmer awareness of tsetse and trypanosomosis and methods used to prevent and/or treat trypanosomosis	

	prior to eradication in Unguja, Zanzibar.	34
Table 17.	Farms, plots affected by tsetse prior to eradication in Unguja, Zanzibar	35
Table 18.	Effect of typanosomosis on cattle production before tsetse control/eradication in Unguja, Zanzibar	36
Table 19.	Effect of trypanosomosis on farms producing milk from indigenous cattle in Unguja, Zanzibar	37
Table 20.	Effect of trypanosomosis on farms producing milk from improved dairy cattle in Unguja, Zanzibar	37
Table 21.	Changes in crop production in Unguja, 1975/76 to 1996/97	39
Table 22.	Land area cultivated and quantities of crops harvested and yields/acre in 1985/86 in Unguja, Zanzibar	40
Table 23.	Value of hides and skins exported to Mainland Tanzania, 1994-1999	41
Table 24.	Slaughter cattle imported from Mainland Tanzania, 1978-1995	42
Table 25.	Value of imported milk and milk products 1992-1998	43
Table 26.	Cattle production systems practised in Unguja, Zanzibar	43
Table 27.	Proportion (%) of farms with livestock in Unguja, 1999	44
Table 28.	Cattle herd structure by age and sex category in Unguja, Zanzibar, 1999	45
Table 29.	Average herd/flock sizes for cattle, chickens, sheep and goats in Unguja, 1999	46
Table 30.	Milk production within the traditional sector in Unguja, Zanzibar, 1999	47
Table 31.	Calving and mortality rates for dairy cattle at the Bambi Research Station, 1986-1992	48
Table 32.	Food crops considered as important by farmers	48

Table 33.	Cash crops considered as important by farmers	49
Table 34.	Changes in land area cultivated before and after tsetse in Unguja, Zanzibar	49
Table 35.	Changes in food and cash crop quantities harvested before and after tsetse intervention in Unguja, Zanzibar	50
Table 36.	Changes in food and cash crop yields obtained before and after tsetse intervention in Unguja, Zanzibar	51
Table 37.	Average herd/flock sizes for cattle, chickens, sheep and goats in Unguja, pre and post tsetse intervention	52
Table 38.	Farmers' ranking of the importance of other animal diseases	52
Table 39.	Farmer awareness of the availability of improved livestock species in Unguja, Zanzibar	54
Table 40.	Farmers' preference for improved livestock breeds and reasons	54
Table 41.	Sources of improved livestock species in Unguja, Zanzibar	55
Table 42.	Important constraints to the acquisition of improved livestock breeds in Unguja, Zanzibar	55
Table 43.	Average prices paid by farmers for improved livestock breeds in Unguja, Zanzibar	56
Table 44.	Access to land in tsetse cleared areas in Unguja, Zanzibar	57
Table 45.	Feed stuffs fed to livestock in Unguja, Zanzibar, 1999	58
Table 46.	Use of animal manure for crop production	58
Table 47.	Use of farm animals for traction and transport	59
Table 48.	Production and reproduction parameters for dairy cattle in Unguja, Zanzibar, 1999	60
Table 49.	Feeding systems and feed stuffs fed to dairy cattle in Unguja, Zanzibar 1999	60
Table 50.	Breeding methods for dairy cattle and sources of breeding	

	materials in Unguja, Zanzibar, post-tsetse eradication	61
Table 51.	Milk and milk products produced by the Zanzibar Dairy Corporation, 1986-1995	62
Table 52.	Projections for beef, poultry, lamb and mutton production in Unguja, Zanzibar, 1986-2015 (Low performance scenario)	63
Table 53.	Projections for beef, poultry, lamb and mutton productions in Unguja, Zanzibar, 1986-2015 (Medium performance scenario)	64
Table 54.	Projections for beef, poultry, lamb and mutton production in Unguja, Zanzibar, 1986-2015 (Medium performance scenario)	65
Table 55.	Projections for milk production per capita in Unguja, Zanzibar, 1986-2015	66
Table 56.	Projections of total and per capita eggs production in Ugunja, Zanzibar, 1986-2015	67
Table 57.	Projections of total and per capita production of food and cash crops in Zanzibar, 1999-2015 (Low performance scenario)	68
Table 58.	Projections of total and per capita production of food and cash crops in Zanzibar, 1999-2015 (Medium performance scenario)	69
Table 59.	Projections of total and per capita production of food and crops in Zanzibar, 1999-2015 (High performance scenario)	69

List of figures

Figure 1.	Map of Unguja Island, Zanzibar	14
Figure 2.	Framework for economic impact assessment of tsetse control/eradication.	76

SUMMARY

The purpose of this assignment was to collect baseline data on the livestock and agriculture situation in Unguja, Zanzibar before the initiation of tsetse/trypanosomosis control/eradication in 1985/86 and the current situation in 1999. While serving as inputs for a future economic impact assessment of tsetse/trypanosomosis control/eradication, the data were to be used to (i) describe the developments in livestock and agriculture that have occurred since the initiation of the intervention in 1985/86 and (ii) predict future developments in livestock and agriculture production. To enable the economic impact assessment to be carried out, a framework of analysis was to be developed.

The approach adopted consisted of a review of the relevant documents on tsetse/trypanosomosis in Zanzibar, collection of data on relevant parameters from these secondary sources; field visits involving informal discussions with key informants and other stake holders; design of a field survey using a formal questionnaire; and data compilation, analysis and report writing.

Based on the informal discussions, the secondary and primary data gathered, there is the general conclusion that the livestock and agriculture situation in Unguja, Zanzibar in 1999 has changed significantly from what it was before the initiation of tsetse/trypanosomosis control/eradication in 1985/86. The overall change is evidenced by increased growth in the livestock sub-sector relative to the crops sub-sector. Livestock contribution to GDP for example, increased from 12% in 1986 to 34% in 1997 whereas the share of crops in GDP dropped from 81% in 1986 to 59% in 1997.

Whereas areas cultivated for crops such as cassava, sweet potatoes, tania (cocoyams), maize and plantain increased or remained the same after 1985/86, yields obtained for these crops declined, suggesting a decline in productivity of land used to produce these crops. Improvements have however, been observed for some crops. Areas cultivated for cloves, yams and coconuts declined after 1985/86, but the yields obtained increased, suggesting that productivity of these crops has improved. This is not surprising especially for cloves as there has been deliberate efforts by the government to revive cloves production in Zanzibar. With regard to livestock, not only has the relative proportion of farms raising cattle and small ruminants increased in 1999 compared to 1985/86, the number of farms with improved cattle breeds has also increased, making it possible for improved cattle to supply 35% of the total milk production compared to about 10% before the intervention. Evidence for intensification of livestock and agricultural activities in Unguja is provided by the increased crop-livestock integration through more farmers using manure for their crop production while in turn using crop by-products to feed their animals. The use of animal power for ploughing and transport activities is also increasing. While several factors may have contributed to the achievement of these results, there is general consensus among people in Zanzibar that the eradication of tsetse and trypanosomosis in 1997 has resulted in significant gains in the livestock sub-sector. Zanzibar's Agricultural Sector Review of 1999 indicates that the International Atomic Energy Agency (IAEA) sponsored tsetse-

eradication project produced some of the best successful results of donor funded projects in East Africa. The information in this report provides some support to this claim.

Although the total production of livestock and agriculture has been projected to increase in the near future, per capita consumption of major livestock and crop products generally fall short of the planned targets. Current production trends indicate that the domestic consumption needs may not be met. Imports will continue to be needed to fill the deficit. However, continued reliance on food imports cannot be sustained and could impact negatively on domestic livestock and agricultural production. Deliberate efforts must be made by Zanzibar authorities in collaboration with private sector and other stakeholders to tackle constraints which are affecting the livestock and agricultural sector. These constraints include poor livestock and crop extension services, processing and marketing of crop and livestock products, diseases and low usage of inputs and improved livestock and crop varieties. Provision of affordable and smallscale oriented credit facilities could greatly improve livestock and crop production.

This report does not purport to have assessed the economic impact of tsetse eradication in Unguja, Zanzibar. Rather, it only provides data on some key parameters and proposes a framework that could be used to carry out an economic impact assessment.

1.0 INTRODUCTION

Agriculture is the most important sector of the economy of Zanzibar. In 1997, it contributed 38% (37,997 Million TShs) (US$ 62.3 million) to the GDP (99,208 Million TShs) (US$ 162.6 million) measured at current market prices), 75% of foreign exchange earnings and employed about 60% of labor force (FAO, 1999; Zanzibar, URT, Department of Statistics, 1999). The relative contribution of agriculture to GDP has been declining in recent years, from an average of 46% between 1980 and 1986 to 39% between 1987 and 1997. The relative decline in agricultural sector contribution to GDP is due to a decline in crop production and an increase in the contribution of other sectors to the economy. In 1986 for example, the share of crops in total agricultural production was 81%. In 1990 crops contributed 77% to total agricultural production whereas in 1997 the contribution was only 59%. On the other hand, the livestock sub-sector's contribution to agricultural GDP has been steadily rising over the last decade. Before 1986 livestock contributed an average of 12% to agricultural GDP. From 1987 to 1997, the contribution of livestock to agricultural GDP averaged 24%, rising from 22% in 1988 to 34% in 1997 (Annex 1). This reflects the important role that livestock has assumed in the economy of Zanzibar and the efforts being made to improve and intensify livestock production.

Efforts to improve and intensify livestock production in Zanzibar have continued to be hampered by a number of constraints, livestock disease being the most important. Up to 1996/97 trypanosomosis was the most important disease constraining livestock production. To control and eradicate the disease, a joint effort between the Food and Agriculture Organisation, United Nations Development Programme (FAO/UNDP) and the Government of Zanzibar (GoZ) was initiated from 1985/86 to 1993. This effort succeeded to a large extent in controlling fly populations and in reducing the incidence of trypanosomosis, but never managed to eradicate the disease. Long-term use of prophylactic or curative drugs for trypanosome suppression was not practicable or economical. Rather, interruption of transmission by vector eradication was considered the most viable long-term solution. In 1994 the International Atomic Energy Agency (IAEA) introduced the Sterile Insect Technique (SIT) under project URT/5/016 - Tsetse Fly Eradication on Zanzibar. In 1997, as a result of a special team effort by the Tsetse and Trypanosomosis Research Institute (TTRI) at Tanga, the Department of Livestock Development-Zanzibar, and the IAEA, tsetse and trypanosomosis were eradicated from Zanzibar.

Following eradication in 1997 there has been a need to assess the economic impact of the intervention on livestock and agriculture development. Unfortunately, the baseline data needed for such an assessment were not available at the start of the tsetse control/eradication interventions i.e. in 1985/86. This study is an attempt to retrospectively collect some information and define a framework for a future economic impact assessment of the tsetse control/eradication intervention. The study reports information collected from secondary sources and from a sample survey conducted in July-August, 1999.

1.1. Objectives

The main objectives of this study are to (i) retrospectively obtain baseline data on the livestock situation and related agricultural and other relevant economic parameters on Unguja Island of Zanzibar before initiation of tsetse/trypanosomosis intervention operations in 1985/86 and the current situation in 1999; and (ii) establish a framework for economic impact assessment of tsetse eradication and related investment into livestock and agricultural development in Unguja. Based on the information collected, the following specific objectives are to be accomplished:

(i) Summarise the status of livestock and agricultural systems in 1985/86, highlighting indicative key parameters for livestock productivity and performance and stressing the respective implications for agricultural systems and for marketing of agricultural products;

(ii) summarise the status of livestock and agriculture development in 1999, highlighting indicative key parameters that show changes in livestock numbers, productivity and performance; quantifying and qualifying the changes and assessing whether they are related to or due to the tsetse fly eradication operations that were implemented in late 1997;

(iii) prepare some predictive scenarios for the livestock and agricultural development through 2005, 2010 and 2015, taking into consideration the livestock and agricultural development over the past 15 years (1985 - 1999) and the assessed potential of livestock and agricultural development of Zanzibar and the respective development plans of Zanzibar; and

(iv) advise on issues that require attention by the Zanzibar authorities and other partners involved in livestock and agriculture development in Zanzibar.

2.0. METHODOLOGY

2.1. Study area

As part of the United Republic of Tanzania, Zanzibar is located 35 km off the eastern coast of Tanzania in the Indian Ocean (Figure 1). It comprises of two sister islands - Unguja and Pemba. Zanzibar has a total land area of 2,332 km^2 and a human population of 840,000 inhabitants. Unguja has a land area of 1,664 km^2 and a population of 493,000 inhabitants. The annual population growth rate is 2.7%.

Unguja has a warm and humid equatorial climate with the main rainy seasons occurring during the months of March to May (Masika) and from October to November (Vuli). The cold season is from June to August while the hot season (Kaskazi) is from December to February. Zanzibar has an average annual rainfall of 114 mm (1996) with a minimum of 5 mm in September and a maximum of 545 mm in May. The annual average temperature is 31°C with a low of 29°C and a high of 33°C.

Figure 1. Map of Unguja Island, Zanzibar

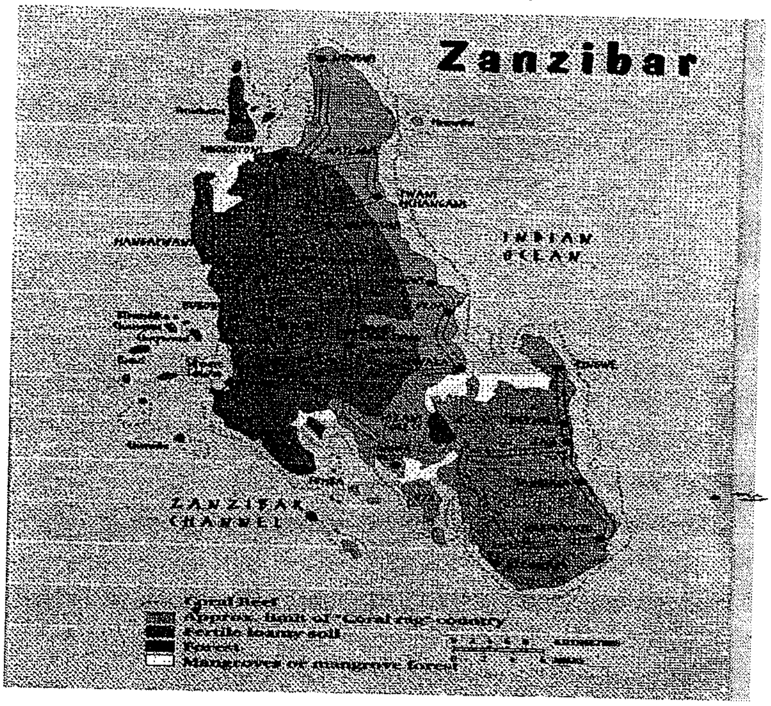

2.2. Field visit

The attempt to collect information for assessment of the economic impact of tsetse control/eradication in Zanzibar began in May 1999 when the team of three persons (agricultural economists) made the first three-week mission to Unguja, Zanzibar. Upon arriving in Unguja, the team met with the senior staff of the Commission of Agriculture and Livestock (CAL) to discuss the logistics and facilitation of the data collection exercise. Following a series of meetings, visits were made to the Ministry of Agriculture, the Agricultural Research and Training Center at Kizimbani, the Department of Statistics, Ministry of State Planning and Investment, and the Ministry of Trade, Industries and Marketing. During these visits, the team examined a wide range of documents and studies related to tsetse and trypanosomosis, the livestock and agricultural situation in Zanzibar, the general economy of Zanzibar and the institutional and policy environment governing livestock and agricultural production in Zanzibar.

The field visits were nevertheless not without problems. The first mission visit coincided with the time when most government officials were busy with annual budget preparations for presentation to Parliament. It was difficult to organize meetings with some of them. Secondary data sources available had inadequate time series data on crop production, livestock numbers and productivity as well as livestock trade. The problem of inadequate data was further compounded by the lack of baseline studies before the tsetse intervention. Where possible, for example in case of livestock numbers, growth rates estimated from available data were used for projections of their numbers and products.

2.3. Sample design

No recent livestock census was available as a sample frame from which the sample farmers could be drawn. However, a list of all the villages in each District of Unguja was available from the Commission for Agriculture and Livestock. A two stage sampling procedure was used. Prior to selecting the sample villages, the cattle population of each district was established based on the 1993 livestock census. The human population and number of households in each District were also established based on the 1995 population estimate. Of the 125 villages, 51% (64) were found to have been infested with tsetse flies before the start of intervention in 1985/86. Taking into consideration the location of each village, the levels of infestation by tsetse flies and the logistics, 50 villages (40%) were identified (Annex 2). A random sample of 1,200 farms was selected taking into account the number of infested villages, households, cattle and human populations (Annex 3). The sample size represented 1.6% of the total number of households in Unguja.

2.4. Development of the questionnaire

A preferred survey tool for establishing parameter values for economic impact assessment would have been to trace over time, the households surveyed before the intervention. This was not possible because no baseline survey was undertaken before the start of tsetse

control/eradication in 1985/86. Since farmers also did not keep records of their farm activities, it was thus necessary to rely on respondent recall of events to obtain data for the before-and-after periods. In developing the questionnaire, a number of steps were taken to handle the well-known problems associated with recall data. The questionnaire and the interview methods were designed to facilitate recall. For example, questions seeking information on events before the start of intervention in 1985/86 were followed by cross check questions and general enquiries to freshen memories, and respondents were invited to refer to particular events at a given time. Enumerators were asked to mention events of past livestock projects, particularly those which had a tsetse control component, of which there was a direct contact between an extension officer and the farmer (Annex 4). Given that farmers are more apt to recall events at the time they initiate an activity (e.g. starting a livestock or crop production enterprise) than during a specific year (e.g. 1985/86), cross check questions were also added for information at the time they started. The rationale of having a large sample size was thus to enable a greater number of those who started in 1985/86 to be interviewed.

The questionnaire contained a total of 66 closed and open ended questions to enable respondents report freely and give reasons for certain answers. It covered the general socio-economic characteristics of the respondents; the status and evolution of livestock and agricultural development at the farm level; livestock and crop production systems; land use practices; crop-livestock interactions; the animal disease situation, feeding, breeding and management practices; the pre- and post-tsetse eradication livestock and crop productivity parameters; and the future potential of livestock and agricultural development. Questions were also included to establish changes in livestock and crop production. Because dairy offers more possibilities for increased intensification, a section of the questionnaire was devoted to the dairy enterprise. The specific questions covered the pre- and post-tsetse eradication productivity parameters for dairy.

2.5. Questionnaire administration

The questionnaire was administered by 18 enumerators (Annex 5) and ~~two~~ supervisors. The enumerators, who were all employees of the Ministry of Agriculture and Livestock were recruited and trained by the team members during the first mission with the assistance of the Tsetse Monitoring Project URT/5/018 staff. During field implementation, the enumerators were organized into four groups and each was assigned a group leader. Interviews began on July 29 and ended on August 15, 1999. In each village, farmers were informed through the *Shehas* (village leaders) one or two days before the proposed date of interviews. Most interviews took place in the morning period to enable farmers attend to their farm duties. Other interviews took place in the evenings, upon the farmer's return from the farm. On average, each questionnaire was completed within 2 hours. Farmers who had been raising livestock for a long time took longer to be interviewed as was the case with those who were growing crops in areas cleared of tsetse flies.

Farmers co-operated well and were willing to spend more time with the enumerators. Their hopes and expectations were quite high upon learning of the survey. The anticipated

difficulties with memory lapse appeared not to have been serious as most farmers were able to provide information for 1985/86 when the tsetse intervention started. Recall was facilitated by events of the time since the campaign against tsetse and trypanosomosis was a major event in Unguja at that time.

Administration of the questionnaire however, was not without a few snags. Unanticipated problems cropped up for certain categories of data. For example, some respondents who thrived on fishing as a major occupation considered it as farming whereas others considered it as another form of occupation. Following the supervisors' reports, the first few days of the survey were characterized by sloppy field work by some enumerators. This necessitated a field visit by one of the team members to monitor and stream line the field activities of the enumerators and supervisors. This intervention notwithstanding, some enumerators managed to turn in questionnaires with problems that the supervisors failed to catch. This necessitated proper screening of the questionnaire prior to data extraction. Approximately 5% of the sample size was discarded following the screening exercise.

2.6. Data compilation and analysis

Prior to data extraction, questionnaires were coded by administrative district. Computer programs were prepared in the Excel software and each response was coded. During data extraction, an unanticipated problem arose from enumerator-introduced errors such as entering the value "zero" where "no response - not applicable" should have been entered. Problems associated with quantity measurements were resolved by converting them into a single unit. For example, milk reported in 750 cc bottles was converted into liters. Quantities of crops (e.g. cassava, rice, maize, tania, sweet potatoes, cloves) reported in bags, *polos* or *gunias* were multiplied by 50 kg. Quantities reported in *pakachas* (local basket made of palm leaves) were multiplied by 15 kgs while quantities of crops (e.g. tomatoes and other vegetables) reported in *susus* (local container made of palm leaves) were multiplied by 5 kg. For coconuts, two pieces were assumed to be equivalent to one kg. Crop yields were estimated by converting quantities harvested into kilograms per acre. The data were analyzed using the Excel software.

3.0. RESULTS

This section reports the information obtained from secondary sources and from interviews conducted during the survey. The survey results are combined with the secondary information to describe the livestock and agriculture situation in Unguja before the tsetse intervention in 1985/86 and the post-tsetse eradication situation in 1999. The first part presents the household socio-economic characteristics of the sample farmers. The second part describes the status of livestock and agriculture development before tsetse intervention in 1985/86, using the key parameters for livestock productivity and performance and discusses the implications for the agricultural production and marketing systems. Part three presents the current (1999) status of livestock and agriculture development, using the key parameters that show changes in livestock productivity and performance and discusses their relationships with the tsetse eradication intervention. In the fourth part, the future livestock and agriculture situation of Unguja is predicted through 2005, 2010 and 2015 under a number of scenarios taking into account the developments over the period from 1985/86 to 1999. Issues that require attention by the Zanzibar authorities and other partners involved in livestock and agriculture development in Zanzibar are discussed in the fifth part. Finally, the conclusions and recommendations are presented.

3.1. Household socio-economic characteristics

3.1.1. Age and sex distribution

About 96% of the sample households were headed by males while only 4% were headed by females (Table 1). North-A and South Districts had all male headed households while the rest of the districts had a small percentage of female household heads. The average age was 41 years with a maximum of 85 years and a minimum of 13 years.[1] Average age varied from 37 years in North-A to 46 years in North-B. Four percent of the households fell within the age category of less than 21 years while 42% fell within the category of 21 to 35 years. About one fifth of the household heads were older than 55 years. These estimates indicate that household heads in Zanzibar are generally of middle age with 79% falling within the 21 to 55 age bracket.

[1] This person lost both parents

Table 1. Sex and age distribution of household heads.

Sex and age category	District					
	Central	North-A	North-B	South	West	All districts
Sex (%): Male	98.0	100.0	93.7	100.0	86.7	95.7
Female	2.0	0.0	6.3	0.0	13.3	4.3
Total number	392	65	255	186	240	1,138
Average age (Years)	38.2	37.4	46.1	38.1	44.5	40.8
Std. de.	13.1	14.4	15.5	12.5	14.5	10.9
Maximum	75.0	70.0	85.0	85.0	85.0	85.0
Minimum	16.0	13.0	18.0	19.0	19.0	13.0
% within this age group						
Less than 21 years	3.6	9.2	1.6	2.7	0.8	3.6
21 to 35 years	49.0	50.8	30.3	48.4	31.3	42.0
36 to 55 years	34.4	26.2	39.0	38.7	44.2	36.5
Greater than 55 years	13.0	13.9	29.1	10.2	23.7	18.0
Total number	392	65	255	186	240	1,138

Source: Economic Assessment of Livestock Development on Zanzibar Survey, 1999.

3.1.2. Education and religious affiliation

Regarding formal education, the results in Table 2 suggest that about 99% of the household heads never went beyond high school level. About 18% had no formal education, 7% had acquired some form of adult education. Household heads with primary level education made up 42% while one third had secondary level education. These results suggest that in general, the level of education in Zanzibar is low, with about two thirds having at best, primary level education. The household heads are predominantly Muslims (95%) with only 4% as Christians and 1% being of other religious affiliations.

Table 2. Educational level and religious affiliation of household heads.

% households within this level of education	District					All districts
	Central	North-A	North-B	South	West	
No formal education	13.8	16.9	27.5	9.2	20.8	17.6
Adult education	6.6	6.2	9.2	4.3	7.3	6.7
Primary	44.6	46.2	39.8	39.5	41.7	42.4
Secondary	32.4	30.8	23.1	47.0	30.2	32.7
High School	2.0	0.0	0.4	0.0	0.0	0.5
University	0.5	0.0	0.0	0.0	0.0	0.1
Total number	392	65	251	186	240	1,134
Religious affiliation (%)						
Muslim	94.4	100.0	98.0	96.2	87.5	95.2
Christian	3.6	0.0	2.0	1.6	12.1	3.9
Others	2.0	0.0	0.0	2.2	0.4	0.9
Total number	392	65	255	186	240	1,138

Source: Economic Assessment of Livestock Development on Zanzibar Survey, 1999.

3.1.3. Household size and structure

Table 3 shows the household structure by age and sex category. The average household size was 8.5 persons with a maximum of 37 persons and a minimum of one person. North-A District had the largest average household size of 9.3 persons while South District had the smallest size of 7.5 persons. Each household had an average of 3 adults and 5 children.

Table 3. Household structure by age and sex category.

Household Size (Number)	District					All districts
	Central	North-A	North-B	South	West	
Average	8.0	9.3	8.9	7.5	8.6	8.5
Std.dev.	3.5	3.9	3.7	3.1	3.9	3.6
Max.	20.0	22.0	27.0	7.0	37.0	37.0
Min.	1.0	1.0	1.0	1.0	1.0	1.0
Less than 12 years	3.0	3.4	3.2	2.8	3.4	3.2
12 to 18 years	2.4	2.8	2.5	2.1	2.2	2.4
Adult Males	1.3	1.5	1.5	1.3	1.4	1.4
Adult Females	1.3	1.6	1.7	1.3	1.6	1.5

Source: Economic Assessment of Livestock Development on Zanzibar Survey, 1999.

3.1.4. Occupational distribution

Farming is the major occupation of the people of Unguja Island. About 88% of the sample household heads reported farming as their major occupation (Table 4). By definition, farming includes livestock keeping and crop agriculture. Wage employment in the public service was an occupation for only 8% of the household heads with 4% of them involved in business and other income earning activities. Among the secondary occupations, farming was reported by about 10% of the household heads followed by 9% in business and 4% in the public service. For those who reported other occupations, fishing ranked first according to 15% of the household heads. Fishing is an important part-time activity (usually carried out during the night) for most people as Unguja is surrounded by the Indian Ocean. Fishing contributes to the household nutritional needs as well as being an income generating activity.

Table 4. Distribution of household heads by occupation.

Major occupation: (%)	District					All districts
	Central	North-A	North-B	South	West	
Farming	87.8	93.9	89.0	84.4	87.5	88.5
Business	1.5	1.5	2.8	1.1	2.1	1.8
Public service	8.9	1.5	6.7	11.8	9.6	7.7
Other	1.8	4.6	0.8	2.7	0.4	2.1
Total number	392	65	255	186	240	1,138
Secondary occupation: (%)						
Farming	12.2	6.0	9.4	10.7	10.8	9.8
Business	7.4	13.9	5.9	6.5	8.8	8.5
Public service	1.0	3.1	2.4	7.5	7.9	4.4
Other	24.8	16.9	8.6	23.1	3.3	15.4
Total number	392	65	255	186	240	1,138

Source: Economic Assessment of Livestock Development on Zanzibar Survey, 1999.

3.1.5. Household income

Household income averaged TShs 41,232 (US$ 52) per month with a maximum monthly income of TShs 850,000 (US$ 1,063) and a minimum of only TShs 2,000 (US$ 2.5) (Table 5). Income is highest in West District despite the widest variation in income distribution. Approximately 97% the households reported an average monthly income of less than TShs 100,000 (US$ 125), a reflection of the generally low income level of farm households in Zanzibar.

Table 5. Average household income and frequency distribution of household income.

	District					
Household income (TSHS/Month)	Central	North-A	North-B	South	West	All districts
Average	40,425<br>(50.5)	29,078<br>(36.3)	33,214<br>(41.5)	48,508<br>(60.6)	54,933<br>(68.6)	41,232<br>(51.5)
Standard deviation	34,460<br>(43.1)	17,856<br>(22.3)	22,938<br>(28.7)	37,372<br>(46.7)	68,456<br>(85.6)	36,216<br>(45.3)
Maximum	350,000<br>(437.5)	90,000<br>(112.5)	160,000<br>(200)	400,000<br>(500)	850,000<br>(1062.5)	850,000<br>(1062.5)
Minimum	2,000<br>(2.5)	3,000<br>(3.7)	5,000<br>(6.3)	10,000<br>(12.5)	3,000<br>(3.8)	2,000<br>(2.5)
% within this income group:						
Less than 25,000 TSHS (31.3)	24.0	50.0	46.3	11.4	20.4	30.4
25,000 to 50,000 TSHS (31.3) to (62.5)	57.4	40.6	39.2	57.8	42.9	47.6
50,001 to 100,000 TSHS (62.5) to (125)	15.3	9.4	13.3	27.0	29.2	18.8
100,001 to 200,000 TSHS (125) to (250)	2.6	0.0	1.2	2.7	4.6	2.2
Greater than 200,000 TSHS (250)	0.8	0.0	0.0	1.0	2.9	0.9
Total number	392	65	255	186	240	1,138

Remarks: Numbers in parentheses are values in US$ (1 US$ = 800 TSHS)

Source: Economic Assessment of Livestock Development on Zanzibar Survey, 1999.

From the preceding estimates, farm households in Unguja can be characterized as being predominantly headed by middle-age Muslim males with less than secondary level education. The households are relatively large with more children than adults and derive a very meagre wage from farming activities. These characteristics have important implications for livestock and agriculture development on the Island, particularly for the adoption of production systems that require human, financial and other physical resources necessary for increasing productivity.

3.2. Livestock and agriculture development in Unguja, pre-tsetse control and eradication, 1985/86.

3.2.1. Livestock production

3.2.1.1. Livestock production systems

Three main livestock production systems could be distinguished in Unguja before the tsetse intervention in 1985/86. These were the extensive, semi-intensive and intensive production systems. The extensive system was characterized by free grazing of cattle, goats and sheep on communal range lands, in fields from which crops have been harvested and in coconut and clove plantations. The cattle grazing system varied according to soil type. In areas with deep soils most cattle were tethered whereas in coral rag areas, free grazing was common. Tethering was mostly done during the planting season to prevent crop damage by animals. In coral rag areas smallholders kept cattle in larger numbers than in deep soil areas where fewer numbers were raised due to the pressure on land. Goats were allowed to scavenge around the homestead and in fields after crop harvests, feeding on a variety of grasses, herds and shrubs. Monogastric livestock such as chickens, ducks and Guinea fowls scavenged around the homestead and were occasionally supplemented with kitchen waste.

The semi-intensive system was practised by very few farmers and involved crossbred as well as highly productive local stock. The animals (mostly cattle) were grazed as in the extensive system, but were supplemented with crop by-products and crop residues. The intensive system was common with commercial dairy and poultry keeping and the breeds involved were mostly exotic and crossbred. For dairy production, the cut-and-carry (zero-grazing) system was commonly practised. Crop by-products, concentrates and mineral supplements were provided. Two intensive poultry keeping systems were operational namely battery caging and deep litter.

3.2.1.2. Livestock numbers

For the two decades preceding 1985/86 only one official livestock census was conducted in 1978 in Zanzibar. At the time Unguja had a livestock population of 28,225 cattle, 13,659 goats, 351 sheep and 319 donkeys (MALNR, 1978). During the next livestock census in 1993 the cattle population was estimated at 45,750 heads (Table 6), implying

that it had increased by 62% or 4% per year. Based on this inter-census growth rate, Unguja's cattle population was estimated at 37,142 cattle in 1986. Indigenous cattle comprised of 94.3% of the total cattle population while exotic and crossbred cattle made up 1% and 4.7% respectively (Table 7). Together, West and Central Districts had 57% of all cattle in Unguja (Table 7). Only 2.5% were available in or around the urban area.

The total population of small ruminants was estimated at 14,010 in 1978. In 1993 it was estimated at 26,847 with sheep comprising an insignificant 1.4%. The annual inter-census growth rate was estimated at 6%, meaning that in 1986 there was an estimated 22,330 small ruminants in Unguja. In 1993 the poultry population in Unguja were at 324,657 chickens, 24,921 ducks and 1,334 Guinea fowls. There were 494 donkeys, having increased by 55% from 319 in 1978. Rabbits and pigs were estimated at 335 and 66 respectively in 1993.

Table 6: Results of the cattle census Unguja Island in 1993

District	Cattle	Goats	Sheep	Chickens	Ducks	Donkeys	Guinea Fowls	Rabbits	Pigs
North A	8,465	6,823	63	50,971	1,606	104	125	18	-
North B	6,849	3,248	40	45,009	2,346	141	123	14	-
Central	12,743	5,714	169	67,963	4,309	148	293	35	43
South	3,406	3,967	-	8,246	1,466	-	64	-	-
Urban	1,164	1,376	13	57,672	7,107	32	318	129	-
West	13,132	5,344	90	94,796	8,086	69	411	139	23
Total	45,750	26,472	375	324,657	24,921	494	1,334	335	

Source: Commission for Livestock, Ministry of Agriculture, Livestock and Natural Resources, Zanzibar.

Table 7. Total number of cattle by type and district in Unguja, 1993.

District	Exotic	Crossbreeds	Local	Total	%
North-A	-	20	8,456	8,456	18.5
North-B	15	123	6,711	6,849	15.0
Central	189	811	11,743	12,743	27.8
South	-	109	3,297	3,406	7.4
Urban	-	191	973	1,164	2.5
West	252	895	11,985	13,132	28.7
Total Unguja	456	2,149	43,145	45,750	100.0
%	1.0	4.7	94.3	100.0	

Source: Commission for Livestock, Ministry of Agriculture, Livestock and Natural Resources, Zanzibar.

3.2.1.3. Livestock farms

Apart from the 1978 livestock census, there is no other source known to us that contains farm level data on the livestock situation in Unguja before the tsetse intervention in 1985/86. The most appropriate means of obtaining this information therefore, was through farmer recall of events at that time. The results indicate that 31% of the 1,138 farmers surveyed in 1999 raised indigenous cattle while 2.4% raised crossbred cattle in 1985/86. About 22% raised chickens and 6% raised sheep and goats (Table 8) in 1985/86. Only 1%

had donkeys. The proportion of farms with indigenous cattle varied from 20% in South District to 44% in North-B District. Farms with crossbred cattle were mostly in North-B (17%), West (5%) and Central (3%) Districts. No farm in North-A District had crossbred cattle. Most of the farms with small ruminants were located in North-B and West Districts while those with local chickens were mostly in North-B (49%), North-A (32%) and West (25%) Districts. Over 60% of the farms with improved poultry were in South District. None was present in North-A District.

Table 8. Farmers raising livestock in Unguja, Zanzibar, before tsetse intervention in 1985/86.

% farms with:	Central (n=392)	North A (n=65)	North B (n=255)	South (n=186)	West (n=240)	All Districts (n=1,138)
Indigenous cattle	27.0	30.8	43.5	19.9	35.0	31.3
Crossbred cattle	2.8	0.0	16.7	1.1	5.0	2.4
Sheep and goats	2.8	1.5	13.4	1.6	7.9	5.6
Local chickens	12.5	32.3	48.9	9.1	24.6	20.6
Improved chickens	0.3	0.0	0.5	2.2	0.4	1.1
Donkeys	1.5	0.0	0.0	0.0	0.8	1.1

Source: Economic Assessment of Livestock Development on Zanzibar Survey, 1999.

Herd and flock sizes on the sample farms averaged 1.53 indigenous and 0.07 crossbred cattle per farm with standard deviations of 5.3 and 1.5 respectively (Table 9). Indigenous cattle averaged 1.7 per farm in Central, North-B and West Districts with no significant difference among the districts. Apart from West District which had the highest number of crossbred cattle (0.2 ± 1.6 sd), no significant difference existed among the other districts. Sheep and goats averaged 0.43 per farm with significant variation among districts. North-B had the highest (1.14 sheep/goats) while North-A had the lowest (0.03). Indigenous and improved chickens averaged 4.3 and 3.2 respectively, being highest in North-B District and lowest in South District.

Table 9. Average herd and flock sizes for cattle, sheep and goats, and poultry on Unguja Island, 1985/86.

District		Indigenous cattle	Crossbred cattle	Sheep and Goats	Indigenous poultry	Improved poultry
Central:	Number	1.73	0.09	0.23	2.40	0.26
	Std. de.	7.01	3.03	3.22	13.48	n.a.
North-A:	Number	1.28	0.0	0.03	6.51	0.0
	Std. de.	4.32	0.0	n.a.	20.06	0.0
North-B:	Number	1.74	0.02	1.14	6.65	6.71
	Std. de.	4.83	0.71	8.96	31.93	179.06
South:	Number	1.20	0.02	0.08	1.45	0.0
	Std. de.	5.02	0.70	1.00	8.44	0.0
West:	Number	1.71	0.16	0.65	4.49	4.17
	Std. de.	5.04	1.64	6.02	14.03	n.a.
Average:	Number	1.53	0.07	0.43	4.30	3.17
	Std. de.	5.26	1.52	4.80	17.59	n.a.

Source: Economic Assessment of Livestock Development on Zanzibar Survey, 1999.

3.2.1.4. Livestock species raised and their production and reproduction parameters

The major livestock species raised by stock owners in Unguja, Zanzibar were cattle, goats and poultry (including ducks and Guinea fowls). Only a limited number of sheep (about 2.5% of all small ruminants), donkeys, rabbits and pigs were raised.

3.2.1.4.1. Cattle

Cattle were the most important livestock species raised because of their meat and milk. The indigenous small-sized, small-humped East African short-horn zebu was the dominant breed, making up over 90% of the Island's total cattle population. In 1985/86 the zebu was estimated to produce about 90% of the total domestic milk with a daily yield ranging from 0.8 to 1.2 liters/cow (Grijpma *et al.*, 1982). The lactation length was estimated to be between 150 and 180 days. Data on other reproductive parameters are lacking for Unguja during the 1985/86 period but can well be assumed to be similar to those reported for mainland Tanzania. Calving intervals ranged from 24 to 26 months, age at first calving from 3 to 4 years. Calf mortality averaged 27% and the overall mortality was 7% (Kadigi, 1995). Mature weights for indigenous cattle ranged from 250 to 300 kg with a carcass weight that ranged from 100 to 110 kg.

Before the tsetse intervention in 1985/86 there were very few exotic and crossbred cattle in Unguja. Most of these were available on state farms located in Bambi, Kizimbani and Pangeni. Farm level data on their production and reproduction parameters are not available as the few farms that raised such animals did not keep any records. However, indicative estimates from the Bambi dairy farm which had a higher standard of management than the ordinary smallholder farm show that calving rates averaged 78.5% for the period 1977 to 1985 while calf mortality on average was 26% and cow mortality averaged 4.5% for the same period (Table 10).

Table 10. Calving and mortality rates at the Bambi dairy farm, 1977-1985.

Year	Calving rate (%)	Mortality rates (%)	
		Cows	Calves
1977	82.7	0	6.2
1978	61.2	0	24.4
1979	51.9	2.3	20.8
1980	77.9	3	24.5
1981	73.7	5.4	19.1
1982	88.3	4.2	50.2
1983	83.6	8.4	32.6
1984	98.2	11.5	30.9
1985	88.7	5.8	25.8
Average 1977-1985	78.5	4.5	26.1

Source: Department of Livestock, MANLR, Zanzibar.

Farm level data on the number of farms producing milk and quantities produced before the tsetse intervention were also not available from secondary sources. Recall information for

1985/86 obtained during the 1999 survey from 166 (15%) livestock farmers who milked their cattle revealed that daily milk yield averaged only 1.0 liters per cow with a standard deviation of 5.4. Milk yield on farms in West and North-B Districts were higher, averaging 1.7 and 1.5 liters respectively compared to only 0.2 liters on farms in South District (Table 11). The large standard errors could have been due to a few farms with higher yields from crossbred and exotic cattle.

Table 11. Milk production at farm level in Unguja, 1985/86.

District	% farmers producing milk	Quantity of milk produced (liters/day)	
		Quantity	Std. de.
Central (n=392)	14.3	0.8	5.4
North-A (n=65)	10.8	0.5	4.2
North-B (n=255)	24.7	1.5	4.7
South (n=186)	7.0	0.2	3.6
West (n=240)	16.3	1.7	8.9
Total (n=1,138)	14.6	1.0	5.4
Number of farmers	166		

Source: Economic Assessment of Livestock Development on Zanzibar Survey, 1999.

3.2.1.4.1.1. Cattle herd structure

Reports of the Zanzibar Ministry of Agriculture, Livestock and Natural Resources indicate that in 1978 when the livestock census was conducted, the cattle herd was composed of 42% cows, 23% calves, 21% heifers and 14% bulls. Oxen comprised of an insignificant proportion and considerable variation by region and production system. Quantitative estimates on the dynamics of the cattle herd following the 1978 livestock census are not available. By how much the structure and composition of the cattle herd had changed in 1985/86 is largely unknown. However, according to the herd structure from the 1993 livestock census which comprised of 42% cows, 25% calves, 19% heifers and 14% bulls, it can reasonably be assumed that in 1985/86 no significant change had occurred in the herd structure except for a 2% decline in the proportion of heifers and a 2% increase in the proportion of calves. Calculated calf/cow ratios for the two periods were 0.55 and 0.59 respectively.

3.2.1.4.2. Small ruminants

The small East African goat and the Black Head Persian sheep were the dominant breeds of small ruminants. The sheep are white in color with a black head and fat rump. They are very few and are available mostly on state farms. The goat has a variable body and short horn that curves backwards. Mature weights range from 25 to 30 kg. As good scavengers and tolerant of harsh conditions, they are very prolific, mature quickly and have 3 kiddings within 2 years. They reach slaughter weight in 8 to 12 months and produce a carcass yield of between 10 and 12 kg (MALNR, 1995).

3.2.1.4.3. Poultry

The people of Unguja raised mostly local chickens as well as other poultry species such as Guinea fowls and Muscovy ducks. The local chicken exhibits a lot of genetic variation with mature males weighing between 1.2 to 1.4 kg and females weighing 0.9 to 1.1 kg. Guinea fowls are grey in color and weigh about 1.5 kg at maturity. Muscovy ducks show considerable variation in color with males weighing 2-2.5 kg and females 1.3-1.7 kg at maturity (MALNR, 1995). Available breeds for broiler production are the Light Sussex while the Rhode Island Red is used for egg production.

3.2.1.4.4. Donkeys

Donkeys are of the common African grey type. They are hardy, resistant and tolerant of many common diseases and thrive on poor quality diet obtained by scavenging on local feed resources. They are mostly used for transportation of all kinds of items ranging from feeds, cash and food crops as well as water.

3.2.1.5. Dairy production, processing and marketing

At farm level, the productivity of dairy cattle in Unguja before and during the tsetse intervention in 1985/86 was quite low. According to estimates provided by Grijpma *et al.* (1982), total daily milk production was 48,000 liters in 1985 with only 7% being produced on state farms and the rest by traditional smallholders. In 1985 total milk available for processing by the dairy plant located at Mapinduzi was estimated at about 2 million liters, averaging 2.2 million liters per year between 1980 and 1985 (Table 12). Most of the milk did not come from local production, but as powder milk from the World Food Program (WFP). The milk was reconstituted and processed into dairy products for sale; the proceeds of which were intended for developing the local dairy industry. During 1985/86, about 4.8 million TShs were generated as sales from reconstituted milk powder. Since the plant relied heavily on milk powder and butter oil, and milk sourced from smallholders was low partly due to the fixed milk price and lack of an organized milk collection system, quantities of dairy products processed through this plant fluctuated significantly.

Table 12. Milk and milk products produced by the Zanzibar Dairy Corporation, 1977-1985.

Year	Milk (1,000 Litres)	Butter (Kg)	Ghee (Litres)	Yoghurt (Litres)	Cream (Litres)
1977	204	475	5,040	10,232	34
1978	641	2,370	17,035	13,885	981
1979	1,442	7,766	9,247	29,161	434
1980	1,896	7,969	8,020	41,501	16
1981	2,325	8,978	141	45,580	29
1982	2,719	8,789	7,830	11,900	177
1983	1,813	8,442	1,510	8,905	3,022
1984	2,510	8,545	1,951	67,698	49,234
1985	2,134	8,465	697	81,885	26,865

Source: Department of Livestock, MALNR, 1995

In rural areas production and marketing of milk and dairy products was mostly by smallholders through informal channels. There was no processing of the milk produced by smallholders and no formal milk marketing channels existed despite the presence of the Mapinduzi Dairy Plant. According to results of the survey, 11% (121) of the sample farms reported selling milk during 1985/86 and the average quantity sold per farm was 0.7 liters (Table 13). North-B District had the largest proportion (20.4%) of farmers selling milk followed by West District (14.6%) and lastly by South District (1.1%). West and North-B Districts reported higher than average milk sales of 1.4 litres/farm and 1.1 litres/farm respectively. Farmers in these districts also produced more milk than those in the other districts. In addition, their relative proximity to the large urban market of Zanzibar town could have contributed to more milk being sold than for the rest of the districts.

Table 13. Milk sales from indigenous cattle in Unguja, 1985/86.

District	% farmers who sold milk	Quantity of milk sold (litres/day)	
		Quantity	Std. de.
Central (n=392)	10.7	0.6	5.1
North-A (n=65)	6.2	0.3	4.6
North-B (n=255)	20.4	1.1	4.3
South (n=186)	1.1	0.1	11.0
West (n=240)	14.6	1.4	8.8
Total (n=1,138)	10.6	0.7	6.8
Number of farmers	121		

Source: Economic Assessment of Livestock Development on Zanzibar Survey, 1999.

3.2.1.6. Livestock disease situation

Before and during the tsetse intervention in 1985/86, livestock in Unguja suffered from a variety of diseases. Trypanosomosis was the most important disease in terms of the losses it caused. Ticks and tick-borne diseases such as East Coast Fever (ECF), Babesiosis and Heartwater were of equal importance. Foot and Mouth Disease, Lumpy Skin Disease, Contagious Caprine Pleuropneumonia (CCPP), Newcastle Disease and African Swine Fever also caused important losses in livestock. Apart from trypanosomosis and ECF for which there exist some data on their impacts on cattle in Unguja, information on the effects of the other animal diseases is unavailable for 1985/86. Mortality from ECF was estimated to vary from 6% to 29% for zebu cattle while for crossbred cattle, mortality was as high as 31% (URT, 1987). Control of ECF and other tick borne diseases was hampered by lack of an effective dipping system and shortages of acaricides. The devastating effects of tsetse and trypanosomosis on cattle in Unguja before the intervention in 1985/86 warrants that the disease be examined more closely.

3.2.1.6.1. Tsetse and Trypanosomosis in Unguja, Zanzibar.

The tsetse fly is a vector of a parasitic disease -- trypanosomosis. It belongs to the genus *Glossina*. The causative organism of trypanosomosis is a parasitic protozoa found in the

blood and tissues of its host. The thirty species or sub-species so far identified are specific to tropical Africa (covering some 11 million km^2) and are classified under three major groups -- *fuscus* (forest), *palpalis* (riverine) and *morsitans* (savannah) (Jordan, 1986; Putt *et al.*, 1980). Fly species within each group are responsible for animal and/or human trypanosomosis (sleeping sickness). However, the rate of infection with trypanosomosis varies from one specie to another and from one locality to the other; the establishment of trypanosome infection being influenced by the ecology, the parasite and its host, and the fly itself (Molyneux, 1977). Overall, infection rates are lower in the *fuscus* and *palpalis* groups than in the *morsitans* group (Jordan, 1986).

Vast areas of the African continent that are inhabited by human and livestock populations are infested with the tsetse fly. Unguja island of Zanzibar was infested with the tsetse fly for over a century. The historical evidence indicates that the island was infested only with animal trypanosomosis caused by *Glossina austini* and not human trypanosomosis. Although first diagnosed in Unguja in 1908, animal trypanosomosis had existed as far back as 1880 (Turner, 1984). The incidence however, assumed greater significance due to the changing socio-economic circumstances that accompanied the abolition of slave trade in 1897.

Until the first tsetse fly *G. austeni* was caught in a diary herd at Chukwani in 1945, it was believed during the first half of the century that trypanosomosis in Unguja was caused by *Stomoxys*. The tsetse surveys conducted by the East African Trypanosomosis Research Organisation between 1945 and 1950 revealed that the tsetse fly was widely present in western Zanzibar, notably in Mwera, Mbusini, Donge, Mwangini, Mangapwani and Kizimbani. Of the 8,000 cattle surveyed for trypanosomosis at the time, 17% diagnosed positive *with T. congolense* or *T. vivax*. The comprehensive tsetse survey conducted between 1948 and 1951 and subsequent mapping of the areas showed an island-wide presence of *G. austeni* with major concentrations in the Jozani forest in the eastern part of the island (Figure 1 Annex 6). The incidence of trypanosomosis was greatest at that time in the wet season when flies were widely dispersed.

Other studies conducted between 1981 and 1983 showed that trypanosomosis was highly prevalent in known endemic areas and even in areas higtherto considered to be free from the disease. In Mangapwani, infection rates were as a high as 60% and 43% in Kinyasini in the north and Muyuni in the south of the island (FAO, 1987, pp. 16-17). Surveys of cattle conducted between 1985 and 1987 revealed a peak prevalence rate of 39% in Jozani forest in November 1985 and 63% in Mgeni Haji in January 1987 (Figure 1 Annex 6). By March 1987, the distribution of tsetse was as shown in Table 14. Cases of trypanosomosis were identified in several villages in North-B, West and Central Districts. Prevalence rates in excess of 30% were also reported in several villages in North-B, Central and South Districts. Other trials conducted between 1985 and 1987 showed that goats, sheep and donkeys were affected as well. Goats, sheep and donkeys sampled in trypanosomosis endemic areas showed that 13%, 6% and 8% were trypanosomosis positive respectively (Hafidh *et al.*, 1987, p. 20). Although data are inadequate, it is highly believed by the

Zanzibar authorities that antelopes and bush pigs are possible reservoirs for trypanosomosis.

Table 14. Prevalence of cattle trypanosomosis in Unguja Island, Zanzibar, March 1987 before the initiation of the Animal Disease Control Project.

District	Prevalence rate			Areas with identified cases of trypanosomosis
	1-10%	11-30%	> 30%	
North "B"	Kitope<br>Mahonda	Zingwesingwe<br>Kiomba-mvua<br>Mkadini	Misufini<br>Mangapwani<br>Fujoni	Makoba<br>Mgambo<br>Pangeni
West				Mfenesini
Central	Tunguu<br>Jendele<br>Umbuji	Chwaka<br>Mgeni Hai<br>Unguja-Ukuu	Mchangani<br>Tunduni	Ukongoroni<br>Charawe<br>Donga Bweni
South			Pete<br>Muugoni<br>Muyuni A, B & C	

Source: Compiled from Hafidh et al., 1987. Appendix 5.

3.2.1.6.2. Tsetse and trypanosomosis control measures

The first major public attempt to control tsetse and trypanosomosis in Unguja started in the 1950s. The control method focused on tsetse reclamation in the high risk areas of Mangapwani in western Zanzibar. However, as trypanosomosis remained a recurring problem of unknown magnitude and economic importance, the need for better understanding of the tsetse and trypoanosomosis situation became imperative as a basis for determining the feasibility and desirability of putting in place an eradication program for *G. austeni*. Based on the recommendations of a number of consultancy missions by Jordan and USAID in 1978 and FAO in 1981 on the control and eradication of *G. austeni* from Zanzibar (Le Roux, 1981), a number of trypanosomosis surveys were commissioned between 1981 and 1983. In 1981 it was shown that the incidence of trypanosomosis on the island was 5%. The subsequent surveys of 1982 and 1983 revealed an overall trypanosomosis prevalence of 3.1% and 4.3% respectively with a prevalence of 10.6% in Mangapwani and 13.6% in Muungoni where small but long-standing foci of fly were present (Table 15). Late in 1982 personnel from the Tsetse and Trypanosomiasis Institute (TTRI) in Tanga, Tanzania thoroughly surveyed known areas of disease in Jozani forest with the view to setting up a herd trial for the use of the sterile insect technique. The puparia collected from Jozani forest were used to start a colony of *G. austeni* at Tanga for eventual mass production. Based on results of the trials and surveys conducted at Jozani forest and Mangapwani between March and October 1983, major decisions for launching an island-wide campaign for the eradication of trypanosomosis were arrived at.

Prior to a request by the Zanzibar Ministry of Agriculture and Livestock for external assistance, control efforts were made under Project URT/73/025 -- Livestock Production, Phase I-- from 1976 to 1978. This project focused mostly on tick control, training and general husbandry at smallholder level. Project URT/78/028 -- Livestock Production,

Phase II -- concentrated on veterinary assistance, breeding, forage production and pasture improvement from 1979 to 1981. Project DP/URT/81/017 -- Livestock Development Phase III -- which was implemented from 1982 to 1986 concentrated on the baseline activities established during Phases I and II, aiming, overall, at raising the productivity of smallholder farms through training in integrated crop and livestock farming (1982-83), training in artificial insemination (1984-85) and the development of disease diagnostic facilities (1985-86) (FAO, 1987).

Given the major challenge from trypanosomosis, the Zanzibar Ministry of Agriculture and Livestock in 1985 requested for assistance from FAO to collect background information on trypanosomosis that could be used to formulate a strategy for its eradication. Two years of data collection by FAO provided background information for the formulation of a pilot trial for the control of *G. austeni* (Bouvry, 1986). The trial was implemented under FAO TCP Project AG:TCP/URT/6758 on the Island of Unguja to test the feasibility and identify a method for the control and possible eradication of trypanosomosis. The technique, which was later to be applied to the entire island, involved the treatment of livestock, mainly cattle, with sequential application of the residual pour-on acaricide deltamethrin.

Following a Government of Tanzania (GoT) request for UNDP assistance in early 1987 to control animal diseases, notably ECF, tsetse and trypanosomosis and other animal diseases, the Animal Disease Control Project, Zanzibar (Project AG:DP/URT/86/022) was negotiated and became operational in March 1988. Phase I covered the period from March 1988 to September 1991. Phase II, which included the island of Pemba, began in October 1991 and ended in December 1993. Building on the results of the pilot trial for the control of *G. austeni*, the Animal Disease Control Project succeeded in reducing the apparent density of *G. austeni* to zero level after application of five, six or more insecticide treatments. Routine post treatment monitoring of both fly and disease in animals in the Jozani and Ufufuma forest areas using "sticky targets" and treatment of trypanosome infected animals showed that the feasibility of using insecticide-treated livestock for the control and possible eradication of *G. austeni* was not only effective but farmer friendly.

Table 15. Prevalence of cattle trypanosomosis in Unguja, Zanzibar, 1982 to 1984.

District	Cattle population	Cattle density (no./km^2)	Area surveyed	Prevalence of cattle trypanosomosis
North A	6,330	27	Mokotoni	0%
North B	2,763	13	Mangapwani<br>Pangeni	10.6%<br>0%
West and Urban	10,152	34	Mtoni<br>Mbweni	0%<br>0%
Central	6,013	13	Bambi<br>Tunguu	6.8%<br>2.8%
South	2,013	6	Muungoni<br>Makunduchi	13.6%<br>0%

Source: Compiled from figures reported in Turner, 1984.

3.2.1.6.3. Tsetse and trypanosomosis eradication using the Sterile Insect Technique (SIT) in Unguja.

The SIT is an *auticide* genetic method of pest control which aims at suppressing the reproduction potential of the female pest such that its control or its total eradication is achieved within a given geographical area. The SIT is based on the release of sufficient males of the pest species, bred and sterilzed in the laboratory, yet capable of copulating or inseminating, into a population of this species so that they compete with the fertile males for the females to be fertilized. The sterilized male population is increased at a rate such that the reproductive potential of the fertile females is severely impaired to permit an eradication of the pest within the area concerned. Once eradicated, efforts (e.g. creation of barrier systems) to sustain the achieved eradication must be put in place.

Initial application of the SIT was in the control and eradication of the screw-worm fly (Baumhover *et al.*, 1955; Knippling, 1960) and the Mediterranean fruit fly (Langley *et al.*, 1972; Spharim, 1974). The first application of the SIT to tsetse eradication in Africa was in 1981 when the technique was used in the Sideradougou area of Burkina Faso. Eradication of the tsetse fly by the SIT method is facilitated by the particular biological characteristics of the tsetse fly. For an insect, the tsetse's reproduction rate of one larva every ten days per reproductive female coupled with the female's ability to mate only once in the first days after hatching and to remain sterile for life following insemination by a sterile male is exceedingly good for eradication. Areas cleared of tsetse flies using the SIT need to either have effective natural barriers or be protected from renewed invasion through insecticide-impregnated traps placed at close intervals along the path of invasion. Unguja has been a very favorable site for the use of the SIT because of its unique status as an island and the presence of only one species of tsetse -- *G. austeni* -- which do not transmit human trypanosomosis.

Before the initiation of the tsetse eradication program using the sterile insect technique in 1994, recurrent measures were undertaken by UNDP/FAO to limit the number and distribution of tsetse and thus decrease the trypanosomosis challenge in the highly infested areas of Zanzibar. The measures typically involved the application of "pour-ons" -- pyrethroid and deltamethrin insecticides -- to eliminate flies from areas of intensive land use and the use of cloth screens impregnated with pyrethroid and deltamethrin insecticides, placed along transects at a density of 30 to 40 per km^2 to reduce the fly density. The use of "pour-ons" led to the reclamation of some 700 km^2 by the end of 1992 within the less infested areas. The method however, was less successful in eliminating flies from the forests and dense thickets in the south of the island. In an area of 8 km^2 of the Jozani forest the use of impregnated screens reduced the fly density by about 85-90%. In the northern part of the island, screens placed at 50 per km^2 succeeded in reducing the fly population by 90% and the fly density from 3 to 0.3 flies/trap/day by April 1993 (UNDP/FAO, 1998, p. 6). While these control methods were on-going, infected animals were treated by dipping with deltamethrin acaricide. In Unguja as a whole, the use of chemoprophylactic methods to reduce fly population and the treatment of animals using

chemotherapeutic drugs reduced the disease incidence and therefore led to an overall improvement to the livestock industry.

The drastic reduction in apparent fly density achieved under the UNDP/FAO Animal Disease Control Project by the end of 1993 provided a fertile ground for the application of the sterile insect technique (SIT) as a method for final eradication of *G. austeni*. In January 1994 the SIT was introduced under the International Atomic Energy Agency (IAEA) Project URT/5/016 which then officially took over from the UNDP/FAO Animal Disease Control Project URT/91/006 in January 1994.

3.2.1.6.4. Farmer awareness of trypanosomosis and its impact.

To determine the extent to which farmers were aware of tsetse and trypanosomosis and how well they co-operated in the fight against the disease, the 1999 survey found that 87% of the sample farmers knew something about tsetse and trypanosomosis before the intervention in 1985/86 (Table 16). About 11% know nothing about the disease while the rest failed to respond. There were no significant differences among the districts in the proportion of farmers who knew something about the disease. Forty eight percent of the farmers sought for measures to prevent and/or treat against trypanosomosis while 44% did nothing about the disease. For those who took measures to prevent the disease, insecticide application was the sole method of prevention while for those who treated their animals, use of trypanocidal drugs (32%) such as Berenil and Samorin was the common method of treatment. Less than 1% combined the use of ethnoveterinary drugs with other methods of treatment. It should be noted that although a few farmers made individual efforts to fight against the disease, prevention and treatment was largely carried out free of charge for farmers by the IAEA and FAO/UNDP tsetse control projects which eventually led to final eradication of tsetse and trypanosomosis from Zanzibar.

Table 16. Farmer awareness of tsetse and trypanosomosis and methods used to prevent and/or treat trypanosomosis prior to eradication in Unguja, Zanzibar.

	District					
	Central (n=392)	North-A (n=65)	North-B (n=255)	South (n=186)	West (n=240)	All districts (n=1,138)
Farmers who are aware of tsetse and trypanosomosis (%)						
Yes	91.10	83.10	87.10	91.90	82.50	87.1
No.	8.20	15.40	11.80	1.10	15.80	10.5
Non-responses	0.70	1.50	1.10	7.00	1.70	2.4
Farmers who prevent and/or treat Trypanosomosis: (%)						
Yes	42.5	32.3	47.1	76.3	44.2	48.5
No.	50.5	60.0	43.9	13.4	52.1	44.0
Non-responses	7.0	7.7	9.0	10.3	3.7	7.5
Farmers who use this method of prevention:						
Bush clearing	0.0	0.0	4.0	0.0	0.0	4.0
Use of traps	0.0	0.0	0.0	0.0	0.0	0.0
Use of insecticides	41.4	32.3	47.1	73.7	43.8	47.7
Avoidance of infested animals	0.0	0.0	0.0	0.0	0.0	0.0
Use of trypanotolerant animals	0.0	0.0	0.0	0.0	0.0	0.0
Raising other animals species	0.0	0.0	0.0	0.0	0.0	0.0
Non-responses	58.6	63.7	48.9	26.3	56.2	50.7
Farmers who use this method of treatment:						
Use of trapanocidal drugs	27.4	27.7	43.5	23.7	38.8	32.2
Use of Ethnoveterinary drugs	0.0	0.0	0.0	0.0	0.4	0.4
Others	0.0	0.0	0.0	0.5	0.4	0.5
Non-responses	72.6	72.3	56.5	75.8	60.4	67.5

Source: Economic assessment of livestock development on Zanzibar survey, 1999.

3.2.1.6.4.1. Impact on cattle production

Although adequate information is available on the prevalence of trypanosomosis in each of the Island's districts, exactly how many farms were affected before the intervention is largely unknown. On average, 54% of the sample farmers reported their farm plots to have been infested by tsetse in 1985/86 (Table 17). The proportion of farms infested varied significantly among the districts, being quite high in South District (86%) and as low as 8% in North-A District. For the infested farms, only 2.3% reported to have abandoned their plots due to tsetse flies. For those who continued to use the infested plots, 51% used them to raise livestock whereas 49% used them for crop production.

Table 17. Farms plots affected by tsetse prior to eradication in Unguja, Zanzibar.

	District					All districts (n=1,138)
	Central (n=392)	North-A (n=65)	North-B (n=255)	South (n=186)	West (n=240)	
Farm plots infested by trypanosomosis (%)						
Yes	66.30	7.70	45.50	85.50	63.80	53.8
No	30.10	90.80	43.10	7.50	34.20	41.1
Farm plots abandoned due to trypanosomosis (%)						
Yes	1.8	0.0	1.6	3.2	2.5	2.3
No	92.3	90.8	89.4	76.9	85.4	87.0
Farm plots used for raising livestock (%)						
Yes	66.10	6.20	40.80	79.60	62.10	51.0
No.	14.00	24.60	16.50	5.40	0.80	12.3
Farm plots used for crop production (%)						
Yes	65.10	7.70	30.90	83.90	59.60	49.4
No.	15.10	12.30	13.30	1.60	0.00	10.6

Source: Economic assessment of livestock development on Zanzibar survey, 1999.

The effect of tsetse infestation was the transmission of animal trypanosomosis to cattle which greatly reduced their productivity and eventually led to deaths when not treated. According to the survey results, 10.2% of the sample farmers reported their cattle to have been infected by trypanosomosis. Among the farms with infected cattle, 42% lost cattle (Table 18). Compared to the entire sample, 4.3% of the farms lost cattle due to trypanosomosis. The number of cattle infected with trypanosomosis was estimated at 0.48, which is 31% of the estimated average herd size of 1.53 cattle per farm. Of the infected cattle, about 76% were reported to have died as a result of trypanosomosis. When compared with the average herd size, it was estimated that 23.5% of the cattle died as a result of trypanosomosis. This estimate falls within the range of 17 to 25% reported by the GURT/FAO/IAEA (1994) Tsetse Fly Eradication on Zanzibar, URT/5/016 Project Document). The amount of weight loss incurred by the infected cattle could not be established as farmers did not weigh their animals. Of the 29 farms sampled with crossbred cattle, 21% reported having infected cattle. The number of crossbred cattle lost due to trypanosomosis could not also be established from the survey results.

Table 18. Effect of trypanosomosis on cattle production before tsetse control/eradication in Unguja, Zanzibar.

Effect of trypanosomosis	District					All districts (n=1,138)
	Central (n=392)	North-A (n=65)	North-B (n=255)	South (n=186)	West (n=240)	
Number of cattle farms affected	30	15	34	8	29	116
% of cattle farms affected	7.6	23.1	13.3	4.3	12.1	10.2
Number of affected farms losing cattle	20	15	7	3	4	49
Farms losing cattle as % of affected cattle farms	66.7	100.0	20.6	37.5	13.8	42.2
Farms losing cattle as % of total cattle farms	5.1	23.1	2.7	1.6	1.7	4.3
Average herd size	1.73	1.28	1.74	1.20	1.71	1.53
Number of cattle affected	0.55	0.02	0.60	0.77	0.54	0.48
Cattle affected as % of herd size	31.80	1.56	34.48	64.17	31.58	31.37
Number of cattle dead	0.26	0.00	0.22	0.52	0.44	0.36
Dead cattle as % of affected cattle	48.00	0.00	36.80	67.50	81.00	76.20
Dead cattle as % of total cattle	15.02	0.00	12.64	43.33	25.73	23.53
Number of farms with crossbred cattle	11	0	4	2	12	29
Farms with crossbred cattle affected	4	0	1	0	1	6
Affected farms as % of farms with crossbred cattle	36.4	0.0	25.0	0	8.3	20.7

Source: Economic assessment of livestock development on Zanzibar survey, 1999.

3.2.1.6.4.2. Impact on milk production

As far as the effect of trypanosomosis on milk production is concerned, 114 farms (10%) with milking cows (mostly indigenous) reported a reduction in milk production from the pre-infection to the post-infection period. Milk production dropped from an average of 1.0 liter per cow before infection to 0.44 liters per cow after infection, a reduction equivalent to 56% (Table 19). For the 10 farms that produced milk from crossbred dairy cattle, milk production was reported to have decreased from an average of 7.8 liters per cow before infection to 2.4 liters per cow post-infection, or a reduction equivalent to 69% (Table 20).

Table 19. Effect of trypanosomosis on farms producing milk from indigenous cattle in Unguja, Zanzibar.

Cattle farms with lactating cows affected by trypanosomosis	District					All districts (n=1,138)
	Central (n=392)	North-A (n=65)	North-B (n=255)	South (n=186)	West (n=240)	
Yes	8.9	4.6	14.1	6.5	15.8	10.0
No.	76.3	67.7	62.4	80.6	75.0	72.4
Non-responses	14.8	27.7	23.5	12.9	9.2	17.6
Milk production:						
Before infection (liters/cow)	0.80	0.50	1.50	0.20	1.70	1.00
After infection (liters/cow)	0.29	0.25	0.67	0.09	0.71	0.44
Change in production (liters/cow)	-0.51	-0.25	-0.83	-0.10	-0.99	-0.56
% Change	-63.6	-50.0	-55.3	-52.6	-58.3	-56.0

Source: Economic assessment of livestock development on Zanzibar survey, 1999.

Table 20. Effect of trypanosomosis on farms producing milk from improved dairy cattle in Unguja, Zanzibar.

Dairy farms with lactating cows affected by trypanosomosis	Dairy farmers responding	
	Number	%
Yes	10	18.5
No.	41	76.0
Non-responses	3	5.5
Milk production:		
Before infection (liters/cow)	7.8	
After infection (liters/cow)	2.4	
Change in production (liters/cow)	-5.4	
% Change	-69.2	

3.2..2. Crop production

3.2.2.1. Crop production systems

3.2.2.1.1. Tree crop production

The main tree crops are cloves and coconuts. Minor tree crops are mangoes and citrus trees. Tree crops are normally intercropped with other trees and food crops. Time series data on tree crop production were only available for cloves. Cloves has been the major cash crop of Zanzibar. Its production has been declining during the last two decades (Annex 7 Tables 1 and 3). Average annual production for the ten year period before tsetse intervention in 1985/86 was 6,275 tons (Table 21). Average production for the period between 1985/86 and 1996/97 was 6,065 tons, a decline equivalent to 3% (Table 21). The decline in cloves production is attributed to ageing of clove trees, diseases, and low clove prices leading to replacement of cloves with other cash crops.

3.2.2.1.2. Food crop production

Roots and tubers such as cassava, sweet potatoes, yams and tania (cocoyams) are the dominant food crops in Zanzibar. Other food crops are cereals (maize, rice and sorghum), banana, legumes (cowpeas, pigeon peas and green gram), and vegetables (Annex 7 Tables 1 to 4). Rice is normally grown in pure stand (monocropped). The other food crops can be grown as sole crops or intercropped.

Cassava is the main root crop in Zanzibar. The crop is normally intercropped and typical crop mix comprises of cassava intercropped with sweet potatoes. Estimated area under cassava and cassava output have been fluctuating. Average annual cassava production for ten years before 1985/86 season was 251,049 tons (Table 12). Average production for the period between 1985/86 and 1996/97 was 147,971 tons. Area under cassava production averaged 55,231 hectares and 36,565 hectares for the period before and after 1985/86, respectively. Pests and disease attack is the major constraint to cassava production in Zanzibar. The major pests are mites and cassava mealy bug and major diseases are brown streak, mosaic and cassava blight.

Sweet potatoes is the second most important root crop in Zanzibar. It is either grown as a sole crop or intercropped with other crops such as cassava. Area under sweet potatoes and output have been fluctuating especially for the period 1985/86 to 1996/97. Average area under sweet potatoes for ten years before 1985/86 season was 14,197 hectares per annum and 9,495 hectares per annum for the period 1985/86 to 1996/97. Annual sweet potato output averaged 38,717 tons for ten year period before 1985/86 and 23,121 tons for the period between 1985/86 and 1996/97 (Table 21). The major constraints to sweet potato production in Zanzibar are shortage of planting materials and poor storage facilities. Pest incidences are low and sweet potato weevil is the only common pest, which can be avoided through early planting.

Yams and cocoyams (other root crops) production has been fluctuating, showing a general declining trend in acreage and output over the period 1985/86 to 1996/96. Area under yams and cocoyams averaged 4,948 hectares per annum for ten years before 1985/86 and 2,635 hectares per annum between 1985/86 and 1996/97. Annual output averaged 19,404 tons for ten years before 1985/86 and 9,955 tons between 1985/86 and 1996/97. These crops are drought resistant and there are no major pests and diseases affecting them.

Table 21. Changes in crop production in Unguja, Zanzibar, 1975/76 to 1996/97.

Type of crop	Before tsetse intervention (Average annual for the period 1975/76 to 1985/86)		During tsetse control/eradication (Average annual for the period 1986/87 to 1996/97)		% change	
	Hectares	Output	Hectares	Output	Hectares	Output
Cloves	n.a.	6,275	n.a.	6,065	n.a.	-3.3
Cassava	55,231	251,049	36,565	147,971	-33.8	-41.1
Maize	9,796	3,561	5,823	1,021	-40.6	-71.3
Rice	28,372	19,360	22,590	15,826	-20.4	-18.3
Banana	17,973	81,695	8,191	33,356	-54.4	-59.2
Sorghum	7,075	1,929	2,376	333	-66.4	-82.7
Legumes	10,231	1,881	8,693	1,308	-15.0	-30.5
Sweet potato	14,197	38,717	9,495	23,121	-33.1	-40.3
Other roots and tubers	4,948	19,404	2,635	9,955	-46.7	-48.7
Total land area under agricultural production (Km^2):						
Unguja	63,770		52,264		-18.0	
Zanzibar	131,570		122,566		-6.8	

Source: Averages and percentages are computed from Ministry of Agriculture statistics, Zanzibar.

Rice is one of the most important food items in Zanzibar. It is produced under rainfed and irrigation conditions with rainfed rice being more important than irrigated rice in terms of acreage and output. Total area under rice and rice output remained almost constant for five year period before 1985/86 and fluctuated slightly between 1985/86 and 1996/96. Area under rice production averaged 28,372 hectares for ten year period before 1985//86 and averaged 22,590 hectares per annum between 1985/86 and 1996/97. Output averaged 19,360 tons per annum for ten year period before 1985/86 and averaged 15,826 tons per annum between 1985/86 and 1996/97 (Table 21). Constraints to rice production include pests and diseases, and poor post harvest technologies which lead into substantial losses.

Maize is an important crop for roasting and for making thick porridge. Area under maize production averaged 9,796 hectares per annum for ten year period before 1985/86 and 5,823 for the period 1985/86 to 1996/97. Annual maize output averaged 3,561 tons for ten year period before 1985/86 and 1,021 tons between 1985/86 and 1996/97. The major constraint to maize production is insect and disease attack. Armyworms, stem borer are the major insects while major diseases are maize streak and white leaf blight.

Sorghum is a drought resistant food crop commonly grown in coral and sub-coral rag areas. Area under sorghum averaged 7,075 hectares per annum for ten year period before 1985/86 and 2,376 hectares per annum between 1985/86 and 1996/7. Output averaged 1,929 tons per annum for ten year period before 1985/86 and 333 tons between 1985/86 and 1996/97.

Banana and plantains are widely grown in Zanzibar. Production of banana and plantains is largely affected by diseases, the major disease being Black Sigatoka. Both area under banana production and output have been fluctuating. Area under banana and plantains averaged 17,739 hectares per annum for ten year period before 1985/86 and averaged 8,191 between 1985/86 and 1996/97. Annual output averaged 81,695 tons and 33,356 tons for ten year period before 1985/86 and for the period 1985/86 to 1996/97.

Legumes such as cowpeas, pigeon peas and green gram are grown as sole crops or intercropped. They are susceptible to pests and diseases both in the field and in storage conditions. Area under legume production averaged 10,231 hectares per annum for ten year period before 1985/86 and averaged 8,693 hectares per annum for the period 1985/86 to 1996/97. Output averaged 1,881 tons per annum for ten years period before 1985/86 and 1,308 between 1985/86 and 1996/97.

Farm level data on land area cultivated for food and cash crops and quantities harvested were obtained from a sample of farmers for the period before tsetse intervention in 1985/86. In general, the total land area cultivated by each household was small as most crops were intercropped with two or more other crops on the same piece of land. The average size of land cultivated before tsetse intervention ranged from 0.3 acres for plantains to 2.0 acres for coconuts (Table 22 and Annex 8 Tables 1 and 3). Annual quantities of crops harvested are summarised in Table 22. For 44% and 26% of the sample farmers for example, the average quantities of cassava and sweet potatoes were 826 kg and 900 kg respectively.

Table 22. Land area cultivated and quantities of crops harvested and yields/acre in 1985/86 in Unguja, Zanzibar.

Type of crop	Land area cultivated	Quantities harvested (kg)	Yield (kg/acre)	% of farmers responding (1,138)
Cassava	1.4	826	597	43.5
Rice	1.1	538	492	24.7
Maize	0.9	429	477	9.0
Banana	1.0	44	44	31.0
Plantain	0.3	13	44	1.3
Sweet potatoes	1.0	899	885	26.1
Yams	0.8	420	545	5.5
Cloves	1.3	224	170	4.0
Coconut	2.0	1,134	580	15.5
Tania	1.0	365	370	5.0
Vegetables	0.5	134	260	4.1

Source: Averages and percentages are computed from Ministry of Agriculture statistics, Zanzibar.

To estimate the productivity of crops, yields obtained per acre were calculated from the quantities harvested from areas cultivated and are also summarised in Table 22. Crop yields before tsetse intervention were generally low, averaging about 0.6 metric tonnes per hectare for cassava, 0.9 metric tonnes for sweet potatoes and 0.5 metric tonnes for rice, maize and yams. These yields are significantly lower than the yields estimated from aggregate level data (see Table 21) and could be due to the inability of farmers to accurately estimate quantities of crop harvested since no records were being kept.

3.2.3. Exports and imports of livestock and agricultural products

Agricultural exports of Zanzibar comprise mainly of crop products. The main export crops have been the traditional crops such as cloves and copra. Following trade liberalization policy in the mid 1980s, agricultural exports have been diversified to include non-traditional crops such as chillies, citrus fruits, mangoes and spices such as ginger, turmeric, black pepper and cinnamon. Except for exportation of hides and skins to mainland Tanzania (Table 23), Zanzibar is a net importer of livestock products. Zanzibar imports various livestock products from Tanzania and abroad. Main livestock products imported into Zanzibar are live animals, meat, and milk and milk products. However, information on these imports is very scanty, probably due to changes in the ministry/department with mandate to keep such data. In the past, all trade statistics used to pass through the Ministry of Trade, Marketing and Industry. Following trade liberalization policy, most export and import transactions are handled by the customs department.

The problem of availability of data on livestock trade is compounded by the fact that statistical abstracts prepared by the Ministry of Statistics of the Ministry of Planning and Investment lump livestock products such as meat and milk together with other food products into one commodity class "food". Thus, the discussion on imports of live animals and milk and milk products is based on scanty information obtained from the Department of Livestock, Ministry of Agriculture and Natural Resources, and the Ministry of Trade, Marketing and Industry.

Table 23. Value of hides and skins exported to Mainland Tanzania 1994-1999

Year	Value in (1,000TShs)	Value in US$
1994	6,270	12,058
1995	7,950	12,885
1996	8,400	13,816
1997	5,400	8,852
1998	5,265	8,100
1999	2,950	3,686

Source: Ministry of Trade, Marketing and Industry.

Table 24 presents the number of slaughter cattle imported from Mainland Tanzania and total cattle slaughtered in Zanzibar for the period before tsetse intervention in 1985/86 and for the period prior to final eradication in 1997. There is a general declining trend in the number of slaughter cattle imported from Mainland Tanzania. On average, Zanzibar

imported more slaughter cattle during the pre-tsetse control period (1978-1985) than during the tsetse control period (1986-1995). Imported slaughter cattle during the tsetse control period contributed about 34% of the total cattle slaughtered compared 71% of the cattle slaughtered in Zanzibar during the pre-tsetse control period (Table 24), suggesting an increase in slaughter cattle obtained from the domestic herd during the tsetse control periods. Despite the absence of slaughter cattle data for the post-tsetse control and eradication period (after 1997) one would expect more slaughter cattle from the domestic herd and eventually achieve the government policy objective of reducing dependency on slaughter animals from mainland Tanzania.

Table 24. Slaughter cattle imported from Mainland Tanzania, 1978-1995.

Year	Cattle from mainland	Total cattle slaughtered	Imported cattle as % of total cattle slaughtered
1978	7,722	8,707	88.7%
1979	4,780	7,624	62.7%
1980	5,608	7,793	71.9%
1981	4,809	6,741	71.3%
1982	3,632	5,968	60.8%
1983	4,514	6,677	67.6%
1984	8,132	9,781	83.1%
1985	4,266	7,531	56.6%
1986	3,611	6,979	51.7%
1987	530	5,186	10.2%
1988	440	5,828	7.5%
1989	953	4,275	22.3%
1990	598	4,998	11.9%
1991	1,168	5,433	21.5%
1992	3,429	7,250	47.3%
1993	4,557	8,331	54.7%
1994	4,028	8,560	47.1%
1995	2,729	8,385	32.5%
Average 1978-85	5,433	7,603	71.5%
Average 1986-95	2,204	6,523	33.8%

Source: Department of Livestock, Ministry of Agriculture, Livestock and Natural Resources (MALNR), various issues.

Apart from beef production, the government of Zanzibar has the objective of achieving self-sufficiency in the production of milk and milk products. Self-sufficiency in milk and milk products is yet to be achieved despite recent increases in domestic milk production (Survey results show that milk production in Zanzibar has increased by 167% between pre-tsetse control/eradication (1985/86) and post-tsetse eradication in 1999. Available data indicate that Zanzibar is still importing milk and milk products to bridge the gap between domestic milk production and demand (Table 25).

Table 25 Value of imported milk and milk products 1992-1998

Year	Value of milk (1,000 TShs)	Value in US$
1992	16,633	55,443
1993	16,633	41,376
1994	98,555	189,529
1995	103,959	168,491
1996	135,383	222,669
1997	267,946	439,256

Source: Ministry of Trade, Marketing and Industry

3.3. Livestock and agriculture development in Unguja, post-tsetse eradication, 1999.

This section examines the current (1999) status of livestock and agriculture development in Unguja taking into account the key parameters that show changes in livestock productivity and performance. It also examines how these changes are related to the tsetse eradication intervention.

3.3.1. Livestock production

3.3.1.1. Livestock production systems

According to the recent description of the livestock production systems provided in the recent Agriculture Sector Review (1999), no major change appears to have occurred in the production systems practised before the tsetse intervention in 1985/86. The dominant livestock production system in Unguja is still the extensive system as indicated by 86% of the sample farmers (Table 26). Under this system, ruminant livestock still depend on low quality indigenous pastures and post-harvest crop residues left in the fields. Poultry rely on grass, insects, grains and kitchen leftovers under this system. About 14% of the sample farmers raise livestock under more intensive systems with more of them found in West and South Districts. Those who practise the semi-intensive system feed a variety of crop by-products and crop residues such as maize stovers, rice husks, bean and groundnut haulms, cassava and banana peels. Livestock under the intensive system are fed improved fodder, crop residues, concentrates and mineral supplements under a zero-grazing system.

Table 26. Cattle production systems practised in Unguja, Zanzibar, 1999.

% farmers practising this feeding system:	District					All districts (n=1,138)
	Central (n=392)	North-A (n=65)	North-B (n=255)	South (n=186)	West (n=240)	
Extensive free range	92.3	98.5	87.5	74.7	75.4	85.7
Intensive (zero-grazing)	5.1	0.0	2.4	0.0	14.6	7.4
Semi-intensive	1.8	0.0	5.5	16.1	7.5	6.2

Source: Economic Assessment of Livestock Development on Zanzibar Survey, 1999.

3.3.1.2. Livestock species raised

Currently, livestock producers in Zanzibar raise cattle, goats, sheep, chickens, ducks, Guinea fowls, donkeys, rabbits and pigs. As was the case before tsetse intervention in 1985/86, cattle, goats and chickens are the most important species in terms of numbers and the proportion of farms raising them.

3.3.1.2.1. Cattle

In the recent survey, 77% of the sample farmers reported raising indigenous cattle, with proportions varying from just 20% in North-A to 95% in Central and South Districts (Table 27). Farms with crossbred cattle make up 18% of the sample, with proportions ranging from 1% in North-A District to 31% in West District. In 1993 the proportion of farms raising cattle was estimated at about 50%. When compared to the current estimate, it can be said that more farmers have become involved in cattle production.

The average number of cattle per farm is estimated at 2.28 (± 4.36 sd) indigenous cattle and 0.59 (± 2.51 sd) crossbred cattle (Table 29). Farms in North-B District have more indigenous cattle (2.6 cattle per farm) compared to farms in South District (1.9 cattle per farm) even though the proportion of farms with cattle is greater in the South than in the North. On average, farms in West and Central Districts have three to four times the number of crossbred cattle in North-B and South Districts.

Table 27. Proportion (%) of farms with livestock in Unguja, 1999.

Livestock species:	Central (n=392)	North-A (n=65)	North-B (n=255)	South (n=186)	West (n=240)	All Districts (n=1,138)
Indigenous cattle	94.6	19.9	35.0	95.2	89.2	77.2
Crossbred cattle	21.9	1.1	5.0	16.7	31.2	18.1
Sheep and goats	14.3	1.6	7.9	13.4	19.6	13.1
Local poultry	55.1	9.1	24.6	48.9	69.2	47.6
Improved poultry	3.8	2.2	0.4	0.5	2.5	2.1
Donkeys	2.8	0.0	0.8	0.0	3.7	1.9
Rabbits	2.3	0.0	0.0	0.0	1.3	1.1

Source: Economic Assessment of Livestock Development on Zanzibar Survey, 1999.

Given the current number of 2.28 cattle per farm and assuming that there are 26,500 livestock farms in Unguja (MALNR, 1998), the 1999 cattle population is estimated at 60,420 cattle. This compares higher than the 57,888 cattle projected for 1999 using an inter-census growth rate of 4%. With an estimated number of 0.59 crossbred cattle per farm, and assuming that 18% of the 26,500 livestock farms keep improved cattle, the total number of improved cattle in 1999 is estimated at 2,814 cattle. According to the 1993 livestock census, exotic and crossbred cattle made up 5.7% (2,605 cattle) of the 45,750 cattle available in Unguja. Assuming an annual growth rate of 4% (estimated from the inter-census populations), the number of improved cattle would be estimated at 3,296 in 1999, which is about 15% greater than the estimate from the survey.

3.3.1.2.1.1. Cattle herd structure

Presently, the cattle herd structure consists of 41% cows, 22% calves, 15% heifers, 9% bulls while steers and oxen account for the remaining 13% (Table 28). Compared to the herd structure in 1993, there appears to be no major change in the proportion of cows. Since the 1993 census did not provide separate classes for mature males, it may well be assumed that bulls included steers and oxen. If that is the case, then there has been a significant change in the proportion of mature males between the two periods. There has also been a decline in the proportion of calves and heifers. As well, the calf/cow ratio has declined from 0.59 in 1993 to 0.53 in 1999.

Table 28. Cattle herd structure by age and sex category in Unguja, Zanzibar, 1999.

Age and sex category	Livestock census, 1993 (% of herd size)	Economic assessment of livestock development survey, 1999	
		Number of cattle per category	% of average herd size
Cows	42.0	0.94	41.23
Calves	25.0	0.50	21.93
Heifers	19.0	0.35	15.40
Bulls	14.0*	0.21	9.21
Steers		0.17	7.46
Oxen		0.11	4.82
Total	100.0	2.28	100.0

* Assumed to include steers and oxen.
Source: Economic Assessment of Livestock Development on Zanzibar Survey, 1999.

3.3.1.2.2. Sheep and goats

The proportion of farms with goats is estimated at 13%, which is close to 11% estimated during the 1993 livestock census (RGZ, Agricultural Sector Review, 1999). Most of the farms with goats are in West (20%), Central (14%) and South (13%) Districts (Table 27). The average number of goats and sheep per farm is 0.80 (± 3.69 sd) with farms in North-A District having fewer numbers (0.51) than farms in North-B District (1.13). The estimated number of 0.8 would give a total population of 21,200 sheep and goats.

3.3.1.2.3. Chickens

About 48% of the sample farmers raise indigenous chickens while 2% raise improved chickens (Table 27). Each farm has an average of 9 (± 12 sd) and 7 (± 198 sd) indigenous and improved chickens respectively. The number of improved chickens vary significantly among the farms because of the existence of a few large-scale commercial poultry producers. Based on these estimates, the number of chickens in Unguja are estimated at 435,130 chickens.

3.3.1.2.4. Donkeys and rabbits

Only 2% of the sample farmers reported having donkeys and 1% having rabbits.

Table 29. Average herd /flock sizes for cattle, chickens, sheep and goats in Unguja, 1999.

District	Average number of animals per farm				
	Indigenous cattle	Crossbred cattle	Goats and sheep	Indigenous chickens	Improved chickens
Central	2.29 (6.54)*	0.96 (3.51)	0.75 (3.65)	8.91 (11.82)	8.87 (206.24)
North-A	2.38 (3.07)	0.0 (0.0)	0.51 (4.77)	9.18 (9.74)	7.69 (2.00)
North-B	2.64 (4.57)	0.26 (3.34)	1.13 (4.30)	10.95 (12.07)	13.03 (349.90)
South	1.91 (4.09)	0.34 (1.30)	0.68 (3.11)	5.61 (7.93)	0.67 (45.86)
West	2.16 (3.53)	1.38 (4.38)	0.92 (2.61)	11.75 (19.75)	5.42 (190.86)
Average	2.28 (4.36)	0.59 (2.51)	0.80 (3.69)	9.28 (12.26)	7.14 (198.20)

Source: Economic Assessment of Livestock Development on Zanzibar Survey, 1999.
* Standard deviations in parentheses.

3.3.1.3. Dairy production

Currently, the dairy sub-sector in Unguja is still largely based on indigenous cattle producing about 65% of total production while the remaining 35% comes from improved cattle breeds raised under more intensive conditions (RGZ, Agricultural Sector Review, 1999). This represents a major change given that in 1986 indigenous cattle were estimated to produce about 90% of the total domestic milk in Unguja.

3.3.1.3.1. Dairy production with indigenous cattle

Recent estimates by the Zanzibar Commission for Agriculture and Livestock (1998) indicate that milk yield by the local zebus raised under the extensive system ranges between 1.5 and 2.5 liters/cow/day. Total annual milk production per cow varies from 200 to 400 liters per lactation with a lactation length ranging from 150 to 180 days and a calving interval of 24 to 26 months. According to the recent survey, 58% of the sample farmers are currently milking indigenous cattle and produce an average of 2.4 liters/cow/day with a standard deviation of 2.5 liters (Table 30).

When compared with estimates provided for the period 1985/86, the current estimates indicate that major changes have, in fact, occurred in the performance of the traditional dairy sector in Unguja. First, the relative proportion of farms milking indigenous cattle increased from 15% to 58% (Table 30), suggesting that over time, more farmers have become involved in traditional dairy production. Secondly, milk production within this sector has also increased, from 1.0 liters/farm to 2.4 liters/farm, an increase equivalent to 167%. This is a reflection of two things. One, whereas other factors may have contributed, the absence of tsetse and trypanosomosis is largely responsible. Before the start of control in 1985/86, an estimated 10.2% of the farms were affected by trypanosomosis with 4.3% of them losing an average of 24% of their cattle and lactating cows losing 56% of their milk. The other fact is an increasing proportion of farms (16%) now having access to new

grazing land (released from tsetse eradication) and an increasing proportion of farms (13%) now feeding crop residues and by-products to cattle.

Table 30. Milk production within the traditional sector in Unguja, Zanzibar, 1999

District	% of farms producing milk		Quantity of milk produced (liters/farm)		
	Before tsetse intervention, 1985/86	After tsetse eradication, 1999	Pre-tsetse eradication, 1985/86		% change in milk production
Central (n=392)	14.3	50.8	0.8 (5.4)*	2.5 (3.0)	213.0
North-A (n=65)	10.8	65.5	0.5 (4.2)	1.9 (1.5)	460.0
North-B (n=255)	24.7	61.1	1.5 (4.7)	2.8 (2.6)	26.7
South (n=186)	7.0	56.7	0.2 (3.6)	2.9 (3.2)	1350.0
West (n=240)	16.3	64.5	1.7 (8.9)	2.0 (2.1)	17.6
Total (n=1,138)	14.6	58.2	1.0 (5.4)	2.4 (2.5)	166.7
Number of farmers	166	662			298.8

Source: Economic Assessment of Livestock Development on Zanzibar Survey, 1999.
* Standard deviations in parentheses

3.3.1.3.2. Dairy production with improved cattle

Recently, the Zanzibar Agricultural Sector Review (1999) estimated that there are about 2,077 smallholder dairy farmers who raise some 6,000 improved cattle under semi-intensive and intensive (zero-grazing) production systems. Taking in to consideration the estimate of 2,605 improved cattle from the 1993 livestock census, the current estimate represents a more than two fold increase in the number of improved cattle in Unguja. Based on research results obtained at the State farms in Bambi and Kizimbani on improved dairy cattle, the Commission for Agriculture and Livestock (1998) estimates that crossbred dairy cattle provide an average of 7 liters/cow/day with a potential of 14 liters whereas for exotic dairy cattle, daily production averages 12 liters with a potential of 27 liters. The lactation length for crossbred cattle is estimated at between 280 and 300 days while the calving rate averages 73%. Calf and cow mortality are estimated at 19.6% and 8.6% respectively (Table 31). Compared to the period before tsetse intervention, the calving rate has decreased by about 6%. Calf mortality has also declined by about half, suggesting an improvement in performance due to reduced disease incidence. While the decline in calving rate could have been due to a drop in the management standards on state farms, the general reduction in the incidence of disease apparent with the control of trypanosomosis could have accounted for the improvement in calf mortality rate.

Table 31. Calving and mortality rates for dairy cattle at the Bambi Research Station, 1986 - 1992.

Year	Calving rate (%)	Mortality rate (%)	
		Cows	Calves
1986	69	7.2	15.0
1987	73	7.3	17.0
1988	77	6.7	35.9
1989	67	14.5	31.3
1990	72	8.5	15.0
1991	75	8.0	10.0
1992	76	8.3	13.0
Average, 1986-1992	73	8.6	19.6
Average, 1977-1985	78.5	4.5	26.1

Source: MALNR, Department of Livestock, 1995.

3.3.2. CROP PRODUCTION

3.3.2.1. Types of crops produced and their importance

In spite of slight differences across the five districts in the ranking of the major food crops, most farmers interviewed reported cassava, banana/plantain, sweet potatoes, rice, yams, maize and vegetables as important food crops (in declining order of importance) (Table 31). Important cash crops (in declining order of importance) as considered by the farmers are cassava, banana/plantain, sweet potatoes, coconut, vegetables, yams, cloves, maize, fruits and rice (Table 32). Almost all the food crops considered as important by the farmers are important sources of cash income. Cassava and banana/plantain appear to be the most important crops not only as food crops but also as the major cash crops in the household economy of Zanzibar. Cloves which is traditionally the major export and cash crop of Zanzibar is one of the cash crops considered important by farmers but ranked below cassava, banana, sweet potatoes, coconut, vegetables and yams (Table 33).

Table 32. Food crops considered as important by farmers.

Farmers who consider these as important (%)	District					All districts (n=1,138)
	Central (n=392)	North-A (n=65)	North-B (n=255)	South (n=186)	West (n=240)	
Cassava	90.8	95.4	97.3	79.0	90.8	90.7
Banana/plantain	80.6	80.0	64.3	57.0	72.1	70.8
Sweet potatoes	58.2	66.2	49	18.3	40.4	46.4
Rice	24.7	73.9	77.7	6.5	43.8	45.3
Yams	25.0	1.5	4.3	45.7	12.1	17.7
Maize	18.6	10.8	5.9	13.4	9.2	11.6
Vegetables	5.1	1.5	0.8	15.0	3.8	5.2
Other food crops	11.2	4.6	4.7	10.2	3.8	6.9

Source: Economic Assessment of Livestock Development on Zanzibar Survey, 1999.

Table 33. Cash crops considered as important by farmers.

Farmers who consider these as important (%)	District					All districts (n=1,138)
	Central (n=392)	North-A (n=65)	North-B (n=255)	South (n=186)	West (n=240)	
Cassava	33.4	33.9	34.9	22	50.8	35.0
Banana/plantain	30.8	26.2	22.8	19.4	37.1	27.3
Sweet potatoes	24.7	41.5	34.1	4.3	19.6	24.8
Coconut	15.3	9.2	38	11.3	29.6	20.7
Vegetables	36.7	15.4	3.5	19.4	4.6	15.9
Yams	14.8	1.5	3.9	20.4	10.4	10.2
Cloves	1.5	4.6	16.1	0	8.7	6.2
Maize	9.4	6.2	1.6	3.8	4.2	5.0
Fruits	13.8	0	1.6	6.4	1.3	4.6
Rice	1.8	4.6	1.6	0.5	4.6	2.6

Source: Economic Assessment of Livestock Development on Zanzibar Survey, 1999.

3.3.2.2. Acreage cultivated

Land area cultivated in 1999 varied from 0.7 acres for yams and vegetables to 1.9 acres for coconuts (Table 34) with West District having the largest amount of land (2.8 acres) devoted to coconuts (Annex 8 Tables 4 to 6). The land areas cultivated per household before tsetse intervention in 1985/86 and after tsetse intervention in 1999 do not vary significantly between most crops. However, relatively more land in 1999 is allocated to the crops given top priority as cash crops and food crops. Comparing the areas cultivated in 1985/86 and in 1999, land areas cultivated for coconut, cloves, rice and yams declined, with area under cloves declining more than the other crops (Table 34). Land areas cultivated for cassava, sweet potatoes, tania (cocoyams) and maize increased slightly or remained the same while land areas cultivated for vegetables and plantain/banana increased substantially (Table 34).

Table 34. Changes in land area cultivated before and after tsetse intervention in Unguja, Zanzibar.

Type of crop	Acreage cultivated (acres)		% change
	Before tsetse intervention in 1985/86	After tsetse intervention in 1999	
Coconut	2.0	1.9	5.0
Cassava	1.4	1.4	0.0
Cloves	1.3	1.0	-23.0
Rice	1.1	1.0	-9.0
Banana	1.0	1.0	0.0
Sweet potatoes	1.0	1.1	10.0
Tania	1.0	1.1	10.0
Maize	0.9	0.9	0.0
Yams	0.8	0.7	-12.5
Vegetables	0.5	0.7	40.0
Plantain	0.3	1.1	267.0

Source: Economic Assessment of Livestock Development on Zanzibar Survey, 1999.

Although the increase in land areas cultivated for some of the crops may be attributed to factors other than influence of tsetse eradication, tsetse eradication appears to have increased access to land for crop production. A large proportion (70%) of the sample farmers who acquired new land after tsetse eradication did so in tsetse cleared areas. The Central district has the largest proportion of farmers who grow crops on land acquired in tsetse cleared areas. Moreover, some farmers (46% of total sample) intend to acquire new crop land in tsetse cleared areas in future. Again, most of the farmers who intend to acquire crop land in tsetse cleared areas are in the Central District.

Comparison of national level data for the period before and after tsetse intervention is not possible because aggregate (national level) time series data on land areas cultivated for major crops in Unguja-Zanzibar were not available for the period after tsetse eradication. National level data were only available for the period before tsetse intervention in 1985/86 and tsetse control/eradication period 1986/87-1996/97 (Annex 7 Tables 2 and 4).

3.3.2.3. Quantities of crops harvested and crop yields

Tables 35 and 36 provide information on quantities of crops harvested and crop yields obtained before tsetse intervention in 1985/86 and after tsetse eradication in 1999 respectively. Except for banana, plantain, yams, cloves and vegetables, quantities harvested during the post-tsetse eradication period of the other crops declined from their pre-tsetse eradication levels. In terms of yields, post-tsetse eradication yields for cassava, rice, maize, plantain, sweet potatoes and tania declined from their pre-tsetse eradication levels.

Table 35. Changes in food and cash crop quantities harvested before and after tsetse intervention in Unguja, Zanzibar.

Type of crop	Quantities harvested (Kg)		% change
	Before tsetse intervention in 1985/86	After tsetse intervention in 1999	
Cassava	826	790	-4.4
Rice	538	443	-17.6
Maize	429	392	-8.6
Banana*	44	52	18.2
Plantain*	13	21	61.6
Sweet potatoes	899	837	-6.9
Yams	420	471	12.1
Cloves	224	249	11.2
Coconut	1,134	1,124	-1.0
Tania	365	355	-2.7
Vegetables	134	197	47.0

* Bunches
Source: 1999 survey results.

Table 36. Changes in food and cash crop yields obtained before and after tsetse intervention in Unguja, Zanzibar.

Type of crop	Crop yields (Kg/acre)		% change
	Before tsetse intervention in 1985/86	After tsetse intervention in 1999	
Cassava	597	559	-6.4
Rice	492	443	-10.0
Maize	477	457	-4.2
Banana*	44	53	20.5
Plantain*	44	19	-56.8
Sweet potatoes	885	756	-14.6
Yams	545	687	26.0
Cloves	170	252	48.2
Coconut	580	608	4.8
Tania	370	324	-12.4
Vegetables	260	285	9.6

* Bunches
Source: 1999 survey results.

3.3.3. Implications for agricultural development and marketing

A closer look at the farm level data on livestock and crop production during the pre- and post-tsetse control/eradication periods reveals a number of important changes that have occurred in the light of tsetse eradication in Zanzibar. These changes have important implications for the future development of agricultural systems and the marketing of agricultural products in Zanzibar.

With regard to the livestock sub-sector, when livestock farms and the number of animals per farm in 1999 are compared with those reported before the tsetse intervention in 1985/86, a number of important changes can be noted. First, the relative proportion of farms raising indigenous cattle, chickens, goats and sheep has increased by about two times between 1985/86 and 1999. The change is even more pronounced for farms with improved cattle increasing six fold (Table 37). The relative increase in farms raising livestock could be attributed to a number of reasons. The first is the increased desire by farmers to diversify their agricultural activities. During the interviews, farmers were unanimous about having animals as a buffer against crop failure. The second reason is the increase due to the general population increase. With an annual population increase of 2.8% and with limited employment opportunities outside agriculture, more people have had no choice but to seek employment opportunities in animal agriculture. The third reason is that with the eradication of tsetse and trypanosomosis in Unguja, farmers have perceived livestock production to be less risky because of the low disease challenge.

Secondly, the number of animals per farm in 1999 has also increased, *al beit* at different rates. The number of indigenous cattle increased by about half from 1.53 to 2.28 cattle per farm (Table 37). The number of crossbred cattle per farm has increased seven fold between 1985/86 and 1999. For sheep and goats, there has been an 86% increase in the number per farm. The number of indigenous chickens more than doubled while the number

of improved chickens per farm increased by 92%. The significant increase in the number of animals per farm could among other factors, be attributed to the relatively low mortality rate experienced in the absence of trypanosomosis in 1999 compared to 1985/86 when the disease had not been eradicated. However, disease still remains a major constraint to livestock production in Unguja because of the prevalence of other diseases. Most farmers interviewed reported East Coast Fever (88%), Lumpy Skin Disease (44%) and Helminthiasis (42%) as important diseases constraining livestock production (Table 38). Another reason for the increase in the number of animals, particularly for improved breeds is the increasing desire by farmers to intensify production.

Table 37. Average herd/flock sizes for cattle, chickens, sheep and goats in Unguja, pre- and post-tsetse intervention.

Livestock species:	Pre-tsetse control, 1985/86		Post-tsetse eradication, 1999		% change in	
	% of farms	Herd/flock size	% of farms	Herd/flock size	% of farms	Herd/flock size
Indigenous cattle	31.5	1.5 (5.26)*	77.2	2.3 (4.36)	145.1	49.0
Crossbred cattle	2.4	0.07 (1.52)	18.1	0.6 (3.13)	654.2	742.8
Sheep and goats	5.6	0.4 (4.80)	13.1	0.8 (3.69)	134.0	86.1
Indigenous chickens	20.6	3.3 (17.59)	47.6	9.3 (12.26)	131.1	181.2
Improved poultry	1.1	3.7 (n.a.)	2.1	7.1 (198.20)	91.0	92.4

n.a. not available
* Standard deviations in parentheses

Table 38. Farmers' ranking of the importance of other animal diseases.

Diseases	District					Total (n=1,138)
	Central (n=392)	North-A (n=65)	North-B (n=255)	South (n=186)	West (n=240)	
East Coast Fever	88.5	90.8	91.8	90.8	79.2	88.2
Lumpy Skin Disease	53.1	32.3	53.3	12.4	69.6	44.1
Helminthiasis	29.3	58.5	53.3	29.6	40	42.1
Foot and Mouth Disease	15.8	6.2	10.9	0	26.3	14.8
Heart Water	14	9.2	4.3	1.1	1.3	6.0
Pneumonia	6.6	1.5	0.4	6.5	1.3	3.3
Other Diseases	5.1	63.1	26.2	0.5	27.5	24.5

In terms of crop production, a number of observations can be noted in the changes observed in crop yields obtained. First, whereas areas cultivated for crops such as cassava, sweet potatoes, tania (cocoyams), maize and plantain increased or remained the same after tsetse intervention, yields obtained for these crops declined. This suggests a decline in productivity of land used to produce these crops.

Second, areas cultivated for cloves, yams and coconuts declined after tsetse intervention but yields obtained increased, suggesting that productivity of these crops has improved after tsetse eradication. This is not surprising especially for cloves as there has been deliberate efforts by the government to revive cloves production in Zanzibar (Personal

communication with Ministry of Agriculture Officials). Cloves has for a long time been the major traditional cash and export crop in Zanzibar. Available aggregate (national level) time series data for the period 1975/76-1996/97 show a general declining trend in production of the crop (Annex 7 Tables 1 and 2). Reasons given for the declining trend in cloves production include poor management of clove trees caused by low returns obtained by farmers as a result of low prices received. Other reasons have been the climatic variations, diseases as well as the insecurity of the three-acre land tenure system for clove production.One of the measures taken by the government in recent years was to restructure the marketing system of the crop as well as provision of good drying facilities to improve quality (Personal communication with Ministry of Agriculture officials). High grade cloves fetches a higher price. The measures taken by the government may have been one of the contributing factors to the increase in cloves production from 1,073 tons in 1995/96 to 11,368 tons in 1996/97 (Annex 7 Table 3).

Third, yields obtained for banana and vegetables increased after tsetse eradication while the area under banana remained the same and that under vegetable production increased, implying increase in productivity (output/acres) in the case of bananas.

3.3.3.1. Intensification of livestock and crop production

Indicators of intensification in livestock production include adoption of high yielding animal breeds accompanied by improved nutritional and health management practices. Intensification in crop production requires the adoption of high yielding crop varieties, use of inorganic and organic fertilizers and other inputs that can raise output per unit area. How well farmers adopt these practices clearly depends on the availability of opportunities and their willingness to take advantage of such opportunities. In Zanzibar, these opportunities include the eradication of major animal diseases such as trypanosomosis which has provided a more favorable environment for raising improved livestock species and has opened up new opportunities for a more optimal use of land resources through integration of crop and animal agriculture as well as the availability of improved animal breeds and crop varieties. The extent however, to which farmers capitalize on such opportunities depends on their level of awareness of the opportunities.

When farmers were asked about their knowledge of the availability of improved livestock breeds in Unguja, 31% reported that they were aware of the availability of crossbred cattle whereas only 6% were aware of exotic breeds. About 13% were aware of the availability of improved poultry breeds while less than 1% knew of improved sheep and goat breeds (Table 39). When information was sought on farmers' preferences for livestock breeds, 62% reported that they preferred improved breeds while 36% preferred local breeds (Table 40). Among the reasons given for preferring improved breeds were their high level of productivity (65%), their ability to mature early (2%) and the fact that they are a source of good genetic material (1%). The reasons given for local breeds include the fact that they are easy to manage (15%), are disease resistant (11%) and can easily be obtained (5%) at low cost.

Table 39. Farmer awareness of the availability of improved livestock species in Unguja, Zanzibar.

Farmers who are aware of The availability of improved livestock species (%):	District					All districts (n=1,138)
	Central (n=392)	North-A (n=65)	North-B (n=255)	South (n=186)	West (n=240)	
Pure-bred cattle: Yes	5.1	7.7	5.9	1.6	11.7	6.4
No	85.0	55.4	36.1	91.9	15.4	56.8
Non-responses	9.9	36.9	58.0	6.5	72.9	36.9
Crossbred cattle: Yes	38.8	27.7	33.3	25.3	30.0	31.0
No	58.0	6.1	32.2	68.3	10.0	34.9
Non-responses	3.2	9.2	34.5	6.4	60.0	22.7
Improved sheep and goats: Yes	0.0	0.0	0.4	0.0	0.0	0.4
No	80.6	52.3	27.1	90.3	15.0	53.1
Non-responses	19.4	47.7	72.6	9.7	85.0	46.9
Improved poultry: Yes	26.5	12.3	12.6	4.3	11.3	13.4
No	63.8	56.9	26.7	86.0	15.0	49.7
Non-responses	9.7	69.2	60.8	9.7	73.7	44.6

Source: Economic Assessment of Livestock Development on Zanzibar Survey, 1999.

Table 40. Farmers' preference for improved livestock breeds and reasons.

Farmers who prefer (%):	District					
	Central (n=392)	North-A (n=65)	North-B (n=255)	South (n=186)	West (n=240)	All districts (n=1,138)
Local breeds: Number	221	8	67	55	57	408
%	56.5	12.3	26.3	29.6	23.7	35.8
Improved breeds: Number	150	57	186	129	183	705
%	38.2	87.7	72.9	69.6	76.3	62.0
Non-responses (%)	5.3	0	0.8	0.8	0	2.2
Reasons for preference (%):						
Local breeds:						
Disease resistance	19.9	1.54	7.06	13	13.8	11.1
Easy to obtain	3.2	4.62	0.39	14.5	1.3	4.8
Easy to manage	40.9	6.15	19.22	8.9	1.3	15.3
Improved breeds:						
High productivity	28.5	87.69	72.16	66.6	70.8	65.2
Improved genetic material	1.1	0	0	1	0	1.1
Early maturity	1.6	0	0	2.6	0	2.1
Others	0	0	0.39	0	0.8	0.6

Source: Economic Assessment of Livestock Development on Zanzibar Survey, 1999.

3.3.3.1.1. Adoption of improved livestock breeds and crop varieties

In spite of farmers' willingness to acquire improved livestock breeds and improved crop varieties in Unguja, the level of adoption remains very low. Farmers do not at the present time have easy access to these resources nor do they have a wide range of options from which to choose. Currently, only 20% of the sample farmers report that they acquire

crossbred cattle from neighbors or other farmers whereas the percentage of farmers acquiring exotic cattle is only 5% (Table 41). Central and West Districts dominate in the number of farmers acquiring improved cattle breeds. For poultry farmers, only 7% report that they acquire improved poultry breeds from private breeding companies. The most important limiting factor to increased adoption of improved livestock breeds according to 46% of the sample farmers is inadequate finances (Table 42).

Table 41. Sources of improved livestock species in Unguja, Zanzibar.

% of farmers who acquire improved livestock from this source:	District					All districts (n=1,138)
	Central (n=392)	North-A (n=65)	North-B (n=255)	South (n=186)	West (n=240)	
Exotic cattle:						
Neighbors and other farmers	3.8	3.1	1.2	3.8	10.4	4.5
Government farm	1.5	3.1	0.0	0.0	0.4	1.7
Livestock market	0.0	0.0	3.1	0.0	0.0	3.1
Crossbreed cattle:						
Neighbors/other farmers	27.8	21.5	11.8	15.1	24.2	20.1
Gov't farm	3.8	3.1	5.1	2.7	4.2	3.8
Livestock market	0.8	1.5	14.1	0.0	0.8	4.3
Improved sheep and goats*	n.r.	n.r.	n.r.	n.r.	n.r.	n.r.
Improved poultry:						
Neighbors and other farmers	2.8	1.5	0.0	2.7	0.0	2.3
Government farm	7.1	0.0	0.0	1.6	0.0	4.4
Livestock market	15.8	9.2	3.9	0.0	0.4	7.3

Source: Economic Assessment of Livestock Development on Zanzibar Survey, 1999.
* No respondent.

Table 42. Important constraints to the acquisition of improved livestock breeds in Unguja, Zanzibar.

Farmers who consider this as a constraint	District					Total (n=1,138)
	Central (n=392)	North-A (n=65)	North-B (n=255)	South (n=186)	West (n=240)	
Inadequate finances	33.4	87.7	51.8	13.4	43.7	46.0
Unavailability	4.6	1.5	3.1	0.5	1.7	2.3
Other factors	5.1	4.6	9.0	0.5	5.4	4.9

Source: Economic Assessment of Livestock Development on Zanzibar Survey, 1999.

For those farmers who are able to acquire improved livestock breeds, the average price paid for a crossbred cattle is Tshs 182,000 (US$ 228) (± 90,000 Tshs) (± 112.5 US$) with a minimum price of Tshs 90,000 (US$ 112.5) and a maximum of Tshs 360,000 (450 US$). For exotic cattle breeds the average price is Tshs 287,000 (359 US$) (± Tshs 95,000) (± 119 US$) (Table 43). The average price for improved poultry is Tshs 528 (US$ 0.7) (± Tshs 304) (±0.4 US$).

Table 43. Average prices paid by farmers for improved livestock breeds in Unguja, Zanzibar

	District					All districts	
	Central	North-A	North-B	South	West	Amount	%
Pure-bred cattle:							
Average price (1,000 Tshs/head)	380 (475)	250 (312.5)	263 (328.7)	124 (155)	418 (522.5)	287 (358.7)	
Standard deviation	113 (141.3)	87 (108.7)	48 (60)	55 (68.7)	174 (217.5)	95 (118.7)	
Maximum	500 (625)	350 (437.5)	300 (375)	350 (437.5)	500 (625)	400 (500)	
Minimum	47 (58.7)	2 (2.5)	200 (250)	60 (75)	80 (100)	78 (97.5)	
Number of farmers	19	3	4	5	26	57	5.01%
Crossbreed cattle:							
Average price (1,000 Tshs/head)	190 (237.5)	190 (237.5)	243 (303.7)	106 (132.5)	181 (226.3)	182 (227.5)	
Standard deviation	99 (123.7)	116 (145)	86 (107.5)	62 (77.5)	89 (111.2)	90 (112.5)	
Maximum	400 (500)	· 500 (625)	450 (562.5)	200 (250)	250 (312.5)	360 (450)	
Minimum	30 (37.5)	20 (25)	100 (125)	40 (50)	40 (50)	46 (57.5)	
Number of farmers	121	12	59	34	70	296	26.01%
Improved poultry:							
Average price	390 (0.5)	790 (1.0)	384 (0.5)	614 (0.8)	461 (0.6)	528 (0.7)	
Standard deviation	260 (0.3)	690 (0.9)	168 (0.2)	234 (0.3)	165 (0.2)	304 (0.4)	
Maximum	1,500 (1.9)	2,000 (2.5)	650 (0.8)	1,500 (1.9)	600 (0.7)	1,250 (1.6)	
Minimum	40 (0.05)	350 (0.4)	45 (0.06)	350 (0.4)	40 (0.05)	165 (0.2)	
Number of farmers	62	5	7	7	26	107	9.40%

Remarks. Numbers in parentheses are values in US$ (1 US$ = 800 TSHS)
Source: Economic Assessment of Livestock Development on Zanzibar Survey, 1999.

3.3.3.1. 2. Access to land resources

According to estimates provided by the National Land Use Plan of 1993, the amount of grazing land in Unguja increased by 30% from 2,140 hectares in 1982 to 3,215 hectares in 1993. With the introduction of tsetse control measures in 1985/86 and the eventual eradication of tsetse flies in 1997, more land became available for crop and livestock production, particularly in the previously infested forest areas of the Island especially around Jozani forest. The government has however declared this forest a protected area to conserve its rich wild flora and fauna. As indicated by the survey results, 23% of the farmers reported that they have farm land in areas cleared of tsetse flies. North-B, West and Central Districts have 30%, 26% and 24% of the farmers with land in areas cleared of tsetse flies respectively. Of the total sample, 16% reported that they have acquired new

farm land in tsetse free areas (Table 44). For those farmers who did not have land in tsetse cleared areas, 46% have plans to do so in the future.

Table 44. Access to land in tsetse cleared areas in Unguja, Zanzibar.

	District					All districts (n=1,138)
	Central (n=392)	North-A (n=65)	North-B (n=255)	South (n=186)	West (n=240)	
Farmers having farm land in tsetse cleared areas after eradication						
Number	94	5	76	26	62	263
%	24.0	7.7	29.8	14.0	25.8	23.0
Farmers acquiring new land in tsetse cleared areas						
Number	86	2	38	10	48	184
%	22.0	3.1	15.0	5.4	20.0	16.2
Farmers intending to acquire land in tsetse cleared areas in future						
Number	237	11	93	80	103	524
%	60.4	17.0	36.5	43.0	43.0	46.0

Source: Economic Assessment of Livestock Development on Zanzibar Survey, 1999.

3.3.3.1.3. Integration of crops and livestock

Not only has the land area available for crop and livestock production increased in 1999, the proportion of farmers using land to integrate crops and livestock has also increased relative to the period before tsetse intervention in 1985/86. This enables a more efficient use of land resources as crop residues and by-products are fed to livestock and as animal manure is in turn used to enrich the soil. Among the sample farmers, 13% are feeding crop by-products to livestock while 8% are feeding concentrates and 4% providing mineral supplements (Table 45) to their animals. West District has the largest number of farmers feeding crop by-products, concentrates and mineral supplements. Only 4% of the farmers are feeding improved fodder to cattle, with the proportion varying from less than 1% in South District to 9% in West District.

Rotational grazing as another form of crop-livestock integration is also gaining prominence in more intensive production systems in Unguja. This practice is common among 59% of the sample farmers who allow cattle to fertilize farm plots and among 51% who grow crops on plots fertilized by cattle (Table 46). The future potential of this practice is also promising according to the intentions of 29% of the sample farmers.

Table 45. Feed stuffs fed to livestock in Unguja, Zanzibar, 1999.

% of farms using these feed stuffs	District					All
	Central (n=392)	North-A (n=65)	North-B (n=255)	South (n=186)	West (n=240)	Districts (n=1,138)
Improved fodder	5.1	4.6	1.2	0.5	9.2	4.1
Crop by-products	11.0	1.5	14.9	5.9	33.3	13.3
Concentrates	4.1	0.0	2.7	0.0	18.3	8.4
Mineral supplements	2.3	0.0	2.0	0.0	8.3	4.2
Other	0.8	0.0	1.2	0.0	0.0	1.0

Source: Economic Assessment of Livestock Development on Zanzibar Survey, 1999.

Table 46. Use of animal manure for crop production.

	District					Total (n=1,138)
	Central (n=392)	North-A (n=65)	North-B (n=255)	South (n=186)	West (n=240)	
Farmers who allow animals to fertelize crop fields						
Number	312	46	110	120	81	669
%	79.6	70.8	43.1	64.5	33.7	58.8
Number of years using animal manure for crop production	6.1	2.8	6.4	7.3	6.2	5.8
Std. De.	5.8	1.5	7.4	5.9	4.6	5.0
Farmers who grow crops on land fertelized with animal manure						
Number	287	31	83	108	67	576
%	92.0	67.4	75.5	90.0	82.7	86.1
% of total	73.2	47.7	32.6	58.1	27.9	50.6
Farmers who intend to use animal manure in future						
Number	106	32	113	42	32	325
%	27.0	49.2	44.3	22.6	13.3	28.5

Source: Economic Assessment of Livestock Development on Zanzibar Survey, 1999.

3.3.3.1.4. Adoption of animal power

Animal power is becoming an important ingredient in the adoption of more intensive systems of livestock and crop production. Animal power used for farm work and for transport of farm produce also enhances the adoption process. In Unguja, the use of animal traction for land preparation is unfortunately limited as only 3% of the sample farmers reported using oxen for ploughing. For transport purposes however, animal power is more popular among 36% of the sample farmers who use animals to transport their own products and among 16% of those who use them for commercial transport (Table 47). The future potential of the use of animal power in Unguja however, appears encouraging as 27% of the sample farmers indicated their intentions to use animals for ploughing and 28% for transport purposes.

Table 47. Use of farm animals for traction and transport.

Farmers who use animal power for:	District					All districts (n=1,138)
	Central (n=392)	North-A (n=65)	North-B (n=255)	South (n=186)	West (n=240)	
Plowing: Number	14	1	7	1	14	37
%	3.6	1.5	2.7	0.5	5.8	3.2
Own transport: Number	102	5	45	16	60	228
%	26.0	7.7	17.7	8.6	25.0	20.0
Commercial transport: Number	80	8	45	16	31	180
%	20.4	12.3	17.7	8.6	13.0	15.8
Farmers who intend to use animal power in future for:						
Plowing: Number	226	18	49	4	7	304
	57.6	27.7	19.2	2.2	2.9	26.7
Transport: Number	97	48	90	31	51	317
%	24.7	73.8	35.3	16.7	21.2	27.7

Source: Economic Assessment of Livestock Development on Zanzibar Survey, 1999.

3.3.3.1.5. Dairy intensification

A good indicator of intensification in dairy production is the degree to which dairy farmers use improved cattle breeds and how well they manage the animals through better feeding and disease control. A good dairy processing and marketing system also offers more access to market outlets as well as the purchase of inputs, thus enhancing intensification of dairy production. In Zanzibar, intensification of dairy production is constrained by inadequate numbers of improved dairy cattle breeds; poor quality feed stuffs consisting mostly of local pastures; low adoption of improved feeding methods involving the use of crop residues and by-products, concentrates and mineral supplements; and the absence of a reliable and efficient dairy marketing system.

To establish productivity parameters for intensified dairy production, a sample of 54 smallholder dairy farmers involved in an FAO/UNDP dairy project was selected out of a total of 76 farmers. Interviews were conducted with the farm operator and information was obtained on their performance over the last two years. According to Table 48, age at first calving for 39 of the dairy farmers averaged 3.1 (± 2.8 sd) years with a minimum of 1.7 years and a maximum of 7.6 years. The calving interval averaged 14.5 (± 4.9 sd) months with a minimum of 12 months and a maximum of 22 months. Daily milk production averaged 8.2 liters with a rainy season production of 8.9 liters and a dry season production of 7.4 liters. Maximum potential milk production averaged 17.5 liters. The estimated average daily milk production represents a 17% increase over the estimate reported for 1995 at the Bambi research station. In general, cows stayed in milk for 7.5 months (228 days), the longest being 11.3 months (342 days) and the shortest being 3 months (91 days). Values of these productivity parameters fall within the range of values reported for dairy cattle raised at the Bambi and Kizimbani state farms.

Table 48. Production and reproduction parameters for dairy cattle in Unguja, Zanzibar, 1999.

Parameter	Dairy farmers responding		Average	Std. de.	Maximum	Minimum
	Number	%				
Age at first calving (years)	39	72.2	3.1	2.8	7.6	1.7
Calving interval (months)	35	64.8	14.5	4.9	22.0	12.0
Lactation length (months)	39	72.2	7.5	1.7	11.3	3.0
Milk production (liters/cow/day):						
Rainy season	36	66.7	8.9	3.8	17.7	1.7
Dry season	38	70.4	7.4	3.5	17.3	1.8
Average	37	68.5	8.2	3.6	17.5	1.8

Source: Economic Assessment of Livestock Development on Zanzibar Survey, 1999.

Among the project dairy farmers, 87% zero-grazed cattle while 4% practised semi-zero-grazing. For 17%, grazing of cattle on the range lands was an alternative source of feeding for their dairy cattle. For farmers who zero-grazed, 57% fed improved fodder while 56% fed crop by-products as the main feed stuffs. About 80% of the farmers provided concentrates while 56% provided mineral supplements (Table 49).

Table 49. Feeding systems and feed stuffs fed to dairy cattle in Unguja, Zanzibar, 1999.

Feeding system and type of feeding material:	Dairy farmers responding	
	Number	%
Zero grazing	47	87.0
Semi-zero grazing	2	3.7
Extensive free range	9	16.7
Feed stuffs:		
Improved fodder	31	57.4
Crop by-products	30	55.6
Concentrates	43	79.6
Mineral supplements	30	55.6
Other	5	9.3

Source: Economic Assessment of Livestock Development on Zanzibar Survey, 1999.

The main method of breeding dairy cattle is by use of improved bulls for 57% of the sample farmers, use of Artificial Insemination (AI) (56%) and use of local bulls (19%) (Table 50). Farmers acquired improved dairy cattle mainly from the State dairy farms located at Bambi, Kizimbani and Mtoni. For those who used AI, the services were mostly obtained from the AI Center (54%). For 44% of the farmers, improved dairy cattle were obtained from their neighbors and other farmers while 28% used their own bulls for crossing. Private organizations provided dairy cattle to only 4% of the dairy farmers.

The average expenditure incurred per head of improved dairy cattle was estimated at 324,000 TShs with a maximum of 600,000 and a minimum expenditure of 200,000 TShs (Table 50).

Table 50. Breeding methods for dairy cattle and sources of breeding materials in Unguja, Zanzibar, post-tsetse eradication.

Breeding system and type of breeding material:	Dairy farmers responding	
	Number	%
Use of improved bulls	31	57.4
Use of artificial insemination	30	55.6
Use of local bulls	10	18.5
Source of improved dairy cattle:		
State farm and Artificial Insemination Center	29	53.7
Own bull	15	27.8
Neighbors and other farmers	24	44.4
Private organizations	2	3.7
Other source	1	1.8
Expenditure for improved dairy cattle (1,000 TSHS/head)		
Average	324	
Standard deviation	110	
Maximum	600	
Minimum	200	

Source: Economic Assessment of Livestock Development on Zanzibar Survey, 1999.

3.3.3.1.6. Processing and marketing of milk and dairy products

Presently in Zanzibar, the only dairy plant with a daily capacity of 10,000 liters is non-operational, having stopped functioning in 1995. The plant was operated by the Zanzibar Dairy Corporation (ZADACO) which was established under the Companies Act (Companies Decree, Cap 153) in October 1987 to take over the functions of the Mapinduzi dairy plant following its closure that year. Between 1986 and 1995 ZADACO supplied an average of 1,134 tons of reconstituted powder milk per year and processed 4,208 kgs of butter and 33,204 liters of yoghurt per year (Table 51). Milk supply peaked in 1992 but by 1995 only 248 tons were supplied, due to the closure of the plant in that year. Most of the milk and dairy products supplied by the plant came from dry powder milk procured under the World Food Program. Milk supply to the plant from smallholders was very low partly due to the fixed price and the absence of an organized milk collection system. Milk supply to the plant fluctuated considerably.

At the present time, milk processing and marketing within the traditional sector is still very rudimentary. Milk is marketed in rural areas in its raw form, neither processed nor pasteurized. The main market for milk and other dairy products is in the urban area of Zanzibar town. Since there is no organized system of milk collection in rural areas where the milk is produced and transport facilities are inadequate, smallholder producers have difficulty accessing this market with the small quantities (average 2-3 litres) available for sale. In some rural areas there are milk vendors (middlemen) who purchase milk from producers and sell directly to consumers. The differential between the established producer price and the informal market price makes it difficult for farmers to capitalize on the high demand coming from the urban area.

Table 51. Milk and milk products produced by the Zanzibar Dairy Corporation, 1986-1995.

Year	Product				
	Milk (1,000 litres)	Butter (Kg)	Ghee (Litres)	Yoghurt (Litres)	Cream (Litres)
1986	1,831	6,923	343	62,888	20,336
1987	1,958	8,769	20	0	23,885
1988	1,534	8,146	11	4,333	21,339
1989	1,118	5,809	72	171,816	16,071
1990	1,447	2,092	3	20,288	0
1991	0	0	0	0	0
1992	1,912	3,013	372	25,545	8,037
1993	1,043	4,512	590	18,380	0
1994	444	2,280	834	13,290	0
1995	248	532	122	15,496	0
Average	1,134	4,208	237	33,204	8,966

Source: Mapinduzi Dairy Farm

Notes: 1990 figure does not include data for December

Production in 1995 was up to September only

3.3.4. Predictive scenarios for livestock and agricultural development through 2005, 2010 and 2015.

The overall goal of Zanzibar's agricultural policy is to promote sustainable development of the agriculture sector. To achieve this goal, a number of policies have been outlined. These policies include among others, increased livestock and agricultural productivity, liberalization and rationalization of production and processing of export crops, and reforms in the agricultural pricing and marketing system. Taking into account the past performance of the livestock and agricultural sub-sectors over the last 15 years (1985 - 1999) and the available opportunities for intensifying animal and crop production, the future production of livestock and crop products in Zanzibar is projected for the years 2005, 2010 and 2015 under three scenarios of low, medium and high performance. The current period (1999), which serves as the baseline for comparison, is an extension of the outcome of the government's policies put in place some years ago. The future performance of the livestock and agriculture sub-sectors is measured in terms of the output of meat, milk, eggs, and food and cash crops. Output projections under each scenario are based on a series of assumptions and data on key parameter values obtained from various sources over the period from 1985/86 to 1999.

3.3.4.1. Meat

Under the low performance scenario, a comparison of the total domestic meat production in 1999 with the production in 1986 shows a 50% increase in total meat within 14 years. In 2005, 2010 and 2015, total meat production is projected to be 20%, 41% and 65% greater than the base year 1999 production respectively (Table 52). Growth in per capita production during the same period is expected to be lower at 4% by 2005 (3.35 kg), 7% by 2010 (3.46 kg) and 11% by 2015 (3.58 kg) respectively, owing to a projected rapid increase in human population.

Currently, chicken accounts for 55% of total domestic meat production followed by beef with 40% while lamb and mutton account for the remaining 5%. Projected share of the projected production of chicken and beef are expected to decline marginally to 53% and 38% by 2015 while the production of lamb and mutton is expected to increase by about 8%.

Table 52. Projections for beef, poultry, lamb and mutton production in Unguja, Zanzibar, 1986-2015 (Low performance scenario)

Year	Beef		Lamb and Mutton		Poultry meat		All meats	
	Total (Mtonnes)	Per capita (kg)	Total (Mtonnes)	Per capita (kg)	Total (Mtonnes)	Per capita (kg)	Total (Mtonnes)	Per capita (kg)
1999	641	1.27	88	0.17	896	1.78	1,625	3.22
2005	765	1.31	123	0.21	1,070	1.83	1,958	3.35
2010	887	1.34	162	0.24	1,241	1.88	2,290	3.46
2015	1,028	1.37	214	0.29	1,439	1.92	2,681	3.58

Assumptions
1. Human population growth rate 2.5% per year
2. Cattle population estimated to grow at 3% per year
3. Cattle offtake rate estimated at 11.4% per year based on the 1986-1995 average cattle slaughters from Unguja Island only
4. Carcass weight for cattle estimated at 103 kg per head based on FAO Production Yearbook estimates for Tanzania.
5. Sheep and goats population estimated to grow at 5.73% per year based on the 1978 and 1993 inter-census annual growth rate.
6. Sheep and goats offtake rate estimated at 23.4% per year based on 1993 sheep and goats estimates for mainland Tanzania.
7. Carcass weight for sheep and goats estimated at 10 kg per head based on Ministry of Agriculture estimates.
8. Poultry population estimated to grow at 3%, per year
9. Offtake rate for poultry is assumed at 100% due to the short cycle nature of poultry production
10. Carcass weight for poultry is assumed at 1 kg per head based on MANLR estimates.

Under the medium performance scenario, the actual production for 1986 is retained but the baseline production is estimated under the assumption that there is less disease risk. The 1986 - 1999 differential in production is thus estimated at 83%. Total meat production is projected to about double within 16 years from 1,983 metric tonnes in 1999 to 3,835 metric tonnes in 2015 (Table 53). On a per capita basis, domestic meat production is projected to increase by only 20% between 1999 (3.74 kg) and 2015 (4.50 kg). While chicken and beef account for 53% and 42% under this scenario, these shares are expected to decline only marginally while the share of lamb and mutton are expected to about double by the year 2015.

Table 53. Projections for beef, poultry, lamb and mutton production in Unguja, Zanzibar, 1986-2015 (Medium performance scenario)

Year	Beef		Lamb and Mutton		Poultry meat		All meats	
	Total (Mtonnes)	Per capita (kg)	Total (Mtonnes)	Per capita (kg)	Total (Mtonnes)	Per capita (kg)	Total (Mtonnes)	Per capita (kg)
1999	827	1.57	111	0.21	1,045	1.96	1,983	3.74
2005	1,407	1.65	166	0.26	1,322	2.08	2,895	3.99
2010	1,274	1.75	233	0.32	1,609	2.18	3,116	4.25
2015	1,550	1.82	327	0.38	1,958	2.29	3,835	4.49

Assumptions
1. Human population assumed to grow at 3% per year based on 1988 population census estimates for Zanzibar.
2. Cattle population estimated to grow at 4% per year based on the 1978 and 1993 inter-census annual growth rate
3. Cattle offtake rate estimated at 13% per year based on slaughter cattle from Unguja Island only
4. Carcass weight for cattle estimated at 110 kg per head
5. Sheep and goats population assumed to grow at 7% per year
6. Sheep and goats offtake rate assumed to be 25% per year
7. Carcass weight for sheep and goats estimated at 11 kg per head based on MANLR estimates.
8. Poultry population estimated to grow at 4%, per year
9. Offtake rate for poultry is assumed at 100% due to the short cycle nature of poultry production

10. Carcass weight for poultry is assumed at 1.1 kg per head based on MANLR estimates.

The high performance scenario, which represents the maximum potential possible, is projected under general assumption of the availability of all opportunities and resources. Under these conditions, total meat production in 1999 is more than double that of 1986 while per capita production increases by about half. By the year 2015, total meat production would exceed current production by 135% *ceteris paribus*. However, per capita production will only be 36% greater (Table 54).

Under the high performance scenario, the current share of beef, lamb and mutton in total meat production is increased to 44% and 7% respectively while the share of chicken declines to 49%, suggesting that beef producers are currently taking more advantage of available opportunities to increase production than producers of the other livestock products. By 2015 however, both the share of beef and chicken in total meat production are expected to decrease to 41% and 45% respectively while the share of lamb and mutton is expected to double.

Table 54. Projections for beef, poultry, lamb and mutton production in Unguja, Zanzibar, 1986-2015 (Medium performance scenario)

Year	Beef		Lamb and Mutton		Poultry meat		All meats	
	Total (Mtonnes)	Per capita (kg)	Total (Mtonnes)	Per capita (kg)	Total (Mtonnes)	Per capita (kg)	Total (Mtonnes)	Per capita (kg)
1999	1,101	1.96	171	0.31	1,207	2.15	2,479	4.42
2005	1,475	2.14	303	0.44	1,618	2.35	3,396	4.93
2010	1,883	2.30	488	0.60	2,065	2.52	4,436	5.42
2015	2,403	2.47	787	0.81	2,636	2.71	5,826	6.00

Assumptions

1. Human population assumed to grow at 3.5% per year
2. Cattle population assumed to grow at 5% per year
3. Cattle offtake rate assumed to be 15% per year based on slaughters of cattle from Unguja Island only
4. Carcass weight for cattle assumed to be 120 kg per head
5. Sheep and goats population estimated to grow at 7% per year
6. Sheep and goats offtake rate estimated at 30% per year
7. Carcass weight for sheep and goats estimated at 12 kg per head based on MANLR estimates.
8. Poultry population estimated to grow at 5%, per year
9. Offtake rate for poultry is assumed at 100% due to the short cycle nature of poultry production
10. Carcass weight for poultry is assumed at 1.2 kg per head based on MANLR estimates.

3.3.4.2. Milk

Milk production under the low, medium and high performance scenarios is shown in Table 55. Under the low scenario, total domestic milk production increased by 48% from 2.45 million liters (6.7 liters per capita) in 1986 to 3.62 liters in (7.2 liters per capita) 1999. Total milk production is projected to increase by 19% by year 2005 with the rate of increase doubling by 2010 and more than tripling by 2015. Under the medium scenario, total milk production will increase from 6.1 million liters (11.5 liters per capita) in base year 1999 to 11.5 million liters (13.4 liters per capita) by year 2015. Under the optimal

production conditions assumed under the high performance scenario, domestic milk production in Zanzibar is projected to more than double from 11.2 million liters in 1999 to 24.4 million liters by the year 2015. Per capita production however, will increase by only 26% from 20 liters to 25 liters (Table 55).

Table 55. Projections for milk production per capita in Unguja, Zanzibar, 1986-2015.

Year	Low scenario		Medium scenario		High scenario	
	Total (1,000 liters)	Per capita (liters)	Total (1,000 liters)	Per capita (liters)	Total (1,000 liters)	Per capita (liters)
1999	3,619	7.18	6,119	11.50	11,167	19.92
2005	4,321	7.39	7,743	12.19	14,965	21.72
2010	5,009	7.58	9,421	12.79	19,099	23.34
2015	5,807	7.76	11,462	13.43	24,376	25.08

Assumptions

1. Human population growth rate 2.5%, 3% and 3.5% per year for low, medium and high scenarios respectively
2. Total cattle population estimated to grow at 3% , 4% and 5% per year for low, medium and high scenarios respectively
3. Proportion of indigenous cattle is estimated to be 94%, 92% and 90% for low, medium and high scenarios respectively
4. Cattle herd structure is composed of 41.23% cows for both indigenous and improved cattle.
5. Proportion of improved cattle is estimated at 6%, 8% and 10% for low, medium and high scenarios respectively.
6. Calving rates for indigenous cattle are assumed to be 55%, 60% and 65% for low, medium and high scenarios respectively
7. Calving rates of improved cattle are assumed to be 67%, 76% and 85% for low, medium and high scenarios respectively
8. Lactation lengths for indigenous cattle are estimated at 180, 200 and 220 days per year for low, medium and high scenarios respectively
9. Lactation lengths for improved cattle are estimated at 280, 305 and 320 days per year for low, medium and high scenarios respectively

3.3.4.3. Eggs

Table 56 shows the projected total egg production and per capita production respectively under low, high and medium scenarios. Total production of eggs under low scenario is projected to increase from 10.4 million eggs in 1999 to 16.8 million eggs by 2015, an increase of about 60%. During the same time period, per capita egg production is projected to increase by about 4%. In the medium scenario, total egg production is projected to increase from 13.3 million eggs in 1999 to 24.9 million eggs by 2015, an increase of about 87%. For the same period, per capita egg production is expected to increase by 16%. In the high scenario, total egg production is projected to increase from 16.4 million eggs in 1999 to 35.9 million eggs by 2105, an increase of about 118%. During the same time period per capita egg production is projected to increase by 27%.

Table 56. Projections of total and per capita eggs production in Unguja, Zanzibar, 1986-2015

Year	Low scenario		Medium scenario		High scenario	
	Total (1,000)	Per capita (No)	Total (1,000)	Per capita (No)	Total (1,000)	Per capita (No)
1999	20,950	42	26,641	50	32,918	59
2005	25,016	43	33,709	53	44,113	64
2010	29,000	44	41,013	56	56,301	69
2015	33,619	45	49,898	58	71,856	74

Assumptions

1. Human population growth rate 2.5%, 3% and 3.5% per year for low, medium and high scenarios respectively
2. Total poultry population estimated to grow at 3%, 4% and 5% per year for low, medium and high scenarios respectively
3. Offtake rate for poultry is assumed at 100% for all the three scenarios
4. Carcass weight for poultry is assumed at 1 kg, 1.1 kg and 1.2 kg for low, medium and high scenarios respectively.

3.3.4.4. Projections for livestock production systems

Considering the livestock productions in 1993, 97% was classified as extensive system while only 3% was intensive. In 1999, extensive livestock production system constituted 86% while intensive systems made up 14%. This gives an annual decline of 1.9% for the extensive system. Based on this decline rate, the extensive system will constitute 76.7%, 69.6% and 63.3% by 2005, 2010 and 2015 respectively. Since the extensive system is declining, one can reasonably assume that this is in favor of more intensive systems. Thus the projected rates for the intensive systems are 23.3%, 30.4% and 36.7% for the years 2005, 2010 and 2015 respectively.

3.3.4.5. Implications of the projections in livestock products

Even though future total and per capita meat, milk and eggs production will increase in Zanzibar as projected, domestic production in general is very low. The projected quantities do not seem to meet the consumption needs which currently are estimated at 3.9 kg of red meat, 2.5 kg of poultry meat, 25 litres of milk and 50 eggs per capita per year. Nevertheless, the projections indicate that Zanzibar will continue to experience a deficit in meat, milk and eggs consumption and will continue to rely on imports of these products unless concerted efforts are made to increase domestic production.

3.3.4.6. Crop production projections, 1999 to 2015.

Zanzibar's agricultural sector review of 1999 estimated that the contribution of food crop production to domestic food supply declined from 60% in 1970 to 42% in 1980 and further to less than 35% in the 1990's. The decline is expected to continue due the downward trend in clove prices. As the main export crop of Zanzibar the price of cloves plummeted from US$ 9,600 per tonne in the early 1980's to US$600 in 1994. This adversely affected the agricultural economy, reducing the contribution of cloves to agricultural GDP from 98% in 1989 to 74% in 1993.

Total crop production in Zanzibar shows a lot of fluctuations from one year to the other due largely to uneven rainfall distribution. Crop production is also being constrained by low usage of improved inputs and high yielding crop varieties due to low purchasing power of the farmers and lack of credit facilities. Other constraints include poor farm management practices, poor soil fertility, crop pests and diseases and lack of effective extension and research services. However, the potential for increasing productivity is great given recent government policies and measures to open up opportunities for agricultural development in Zanzibar.

Projections for the main food and cash crops in Zanzibar are shown in Tables 57 to 59. These projections have been done under three scenarios which generally assume a declining growth rate in the low scenario, a constant growth rate in the medium scenario and a positive growth rate under the high scenario. These assumptions stem from the fact that crop production in Zanzibar has at best remained constant otherwise decreased over the last ten years. The main food crops like cassava, sweet potatoes, banana and rice have relatively higher total and per capita production than cash crops such as cloves. Both total and per capita food and cash crops production only show a significant growth rate in the high scenario only where a positive growth rate is envisaged. This indicates that deliberate efforts must be made by the GoZ to increase production of the main food and cash crops so as to earn the much need foreign exchange and meet the domestic consumption needs.

Table 57. Projections of total and per capita production of food and cash crops in Zanzibar, 1999-2015 (Low performance scenario).

Crop	1999		2005		2010		2015	
	Total (MT)*	Per capita (kg)	Total (MT)	Per capita (kg)	Total (MT)*	Per capita (kg)	Total (MT)	Per capita (kg)
Cloves	5.9	10	5.5	10	5.1	10	4.8	10
Cassava	144.3	290	133.8	230	125.6	190	118.0	160
Maize	1.0	2	0.92	2	0.87	1	0.80	1
Rice	15.4	31	14.3	24	13.4	20	12.6	17
Banana	32.5	65	30.2	52	28.3	43	26.6	36
Sorghum	0.32	1	0.30	1	0.28	0.4	0.27	0.4
Legumes	1.28	3	1.2	2	1.1	2	1.0	1
Sweet potato	22.3	45	21.0	36	19.6	30	18.4	25
Other roots and tubers	9.7	19	9.0	15	8.4	13	7.9	11

* MT = metric tonnes

Assumptions:

In the low scenario, total annual production for each of the main cash and food crop is projected to decline by 1.25% per year. The agricultural sector review of 1999 for Zanzibar projected that total annual production for most food and cash crops have been declining steadily since 1980's through to 1990's. The downward trend is expected to continue.

Table 58. Projections of total and per capita production of food and cash crops in Zanzibar, 1999-2015 (Medium performance scenario).

Crop	1999		2005		2010		2015	
	Total (MT)*	Per capita (kg)	Total (MT)	Per capita (kg)	Total (MT)*	Per capita (kg)	Total (MT)	Per capita (kg)
Cloves	8.0	20	8.0	10	8.0	10	8.0	10
Cassava	148.0	280	148.0	230	148.0	200	148.0	170
Maize	1.0	2	1.0	2	1.0	1	1.0	1
Rice	15.8	30	15.8	25	15.8	21	15.8	19
Banana	33.3	63	33.3	53	33.3	45	33.3	39
Sorghum	0.3	1	0.3	1	0.3	0.5	0.3	0.4
Legumes	1.3	2	1.3	2	1.3	2	1.3	2
Sweet potato	23.1	43	23.1	36	23.1	31	23.1	27
Other roots and tubers	9.9	19	9.9	16	9.9	14	9.9	12

In the medium scenario, total annual production for each of the main cash and food crops is projected to remain the same as that of 1997. The current production levels have almost remained constant since 1997.

Table 59. Projections of total and per capita production of food and cash crops in Zanzibar, 1999-2015 (High performance scenario).

Crop	1999		2005		2010		2015	
	Total (MT)*	Per capita (kg)	Total (MT)	Per capita (kg)	Total (MT)*	Per capita (kg)	Total (MT)	Per capita (kg)
Cloves	10.0	20	38.1	60	116.4	140	355.3	370
Cassava	185.0	330	705.6	1,020	2,153.3	2,630	6,571.2	6,750
Maize	1.6	3	6.1	10	18.6	20	56.7	60
Rice	1.6	3	6.1	10	18.6	20	56.7	60
Banana	52.1	90	198.8	290	606.7	740	1,851.6	1,900
Sorghum	0.5	1	1.9	3	6.0	7	18.4	19
Legumes	2.0	4	7.8	10	23.8	30	72.6	70
Sweet potato	36.1	60	137.8	200	420.6	510	1,283.5	1,320
Other roots and tubers	15.5	30	59.3	90	181.1	220	552.6	570

In the high scenario, total annual production for each of the main cash and food crop is projected to increase by 1.25% per year.

3.3.5. Issues requiring attention by the Zanzibar authorities and other partners

It has been reported elsewhere that cattle herds without trypanosomosis are more efficient than infected cattle herds because the absence of trypanosomosis will enable herds to grow faster over time, resulting in more milk and meat production. Apart from benefits from

increased milk and meat production, there will be benefits to crop production through the increased availability of animal manure and animal draught power for traction and transportation of agricultural products.

In general, the farm level survey results suggest that tsetse eradication has stimulated livestock production as well as increased crop-livestock integration. Cattle herd sizes have increased between 1985/86 and 1999, suggesting that tsetse control and eradication measures undertaken between 1985/86 and 1997 have stimulated livestock growth. Moreover, farmers intend to keep more crossbred cattle and use more manure for crop production. The increase in land productivity for some of the crops mentioned above may be associated with intensification in the smallholder farming systems in Zanzibar. Indicators of intensification in crop production include adoption of high yielding crop varieties, use of inorganic and organic fertilizers and other inputs that can raise output per unit area. Use of improved crop varieties by smallholder farmers in Zanzibar appear to be low. Very few farmers reported the use of improved varieties for rice, vegetables, fruits and maize. None of the sample farmers reported the use of improved banana varieties. Thus, the increase in banana productivity may be associated with increased use of manure (organic matter) after tsetse intervention, suggesting more integration of livestock and crops in the farming systems after tsetse intervention. There is evidence of crop-livestock integration from the farmer level survey data in terms of use of animal manure for crop production (Table 43) and use of farm animals for traction and transport of agricultural produce (Table 44).

The rate at which the smallholder farmers in Zanzibar can intensify livestock and crop production and benefit from tsetse eradication depends on the rate at which they adopt technologies. Technologies such as use of crossbred animals which have relatively high production potential, use of improved seed varieties, use of improved breeding methods, disease control and good animal nutrition are necessary for intensification. However, a number of constraints for which farmers have limited knowledge and/or resources to address were identified from the survey results. Farmers may need external assistance to accelerate adoption of various technologies.

- Most of the farmers interviewed (77%) keep indigenous cattle which are low meat and milk producers, producing between 1 and 3 litres of milk per cow daily. Options available to the farmer for obtaining a high yielding animal are purchase of a crossbred animal to replace the indigenous animal or upgrading the indigenous cattle using artificial insemination and/or improved bulls. The acquisition of improved livestock breeds such as crossbred animals is mainly constrained by inadequate finances as indicated 46% of the farmers interviewed. Upgrading of indigenous cattle is constrained by unavailability of artificial insemination services (presently the artificial insemination unit is non-operational due to lack of liquid nitrogen) and improved bulls as majority of the farmers keeping indigenous cattle use local bulls. Currently the artificial insemination unit is non-operational due to lack of liquid nitrogen.

Interventions by the government and other parties interested in livestock development may include:

(i) Establishment of heifer in trust schemes or smallholder credit schemes. Heifer in trust schemes may be more effective than credit schemes since credit given in cash may be directed to other uses unless it is given in kind.

(ii) Provision of artificial insemination service as a strategy to improve genetic potential of dairy animals.

(iii) Establishment of improved bull centres to complement the use of AI

(iv) Training of farmers on heat detection and record keeping

- Almost all interviewed farmers are aware and acknowledgeable that tsetse flies have been eradicated in Unguja, Zanzibar. However, other diseases such as East coast Fever, Lumpy Skin Disease, Helminthiasis, Foot and Mouth Disease, Heart Water and Pneumonia as ranked by farmers in declining order of importance still constrain cattle production in rural areas. Most of the Veterinary Investigation Centers (VICs) lack the necessary drugs and vaccines

Intervention by government and/or other parties interested in livestock development

(i) Strengthening animal health service in the rural areas.

(ii) Establish a revolving fund for purchase of veterinary drugs and vaccines

(iii) Gradually involve the private sector in the provision of veterinary service

(iv) Training of farmers on characteristics of common diseases

- Most of the farmers (86%) interviewed are practising extensive free grazing whereby cattle graze on natural pastures without supplementation. The remaining 14% practice zero or semi-zero grazing whereby animals are fed on natural pastures or home grown fodder with little supplementation. Very few farmers reported the use of supplementary feeds such as concentrates, minerals, crop by-products. Cassava peels was the only crop being used as reported by some of the sample farmers.

Intervention by government and/or other parties interested in livestock development

(i) Farmer training on the use of supplementary feeds

(ii) Feed conservation techniques for use during dry season

(iii) Fodder establishment-fodder grasses mixed with legumes to improve nutritive value

- Overall the results of the survey show that the number farmers selling milk as well as average quantity of milk sold have increased after tsetse intervention. Moreover, farmers interviewed indicated their intentions to increase milk production in the future through adoption of improved cattle breeds. This will obviously increase milk production in the rural areas and the issue of establishing an organized and efficient milk marketing and processing system cannot be ignored. The only milk processing plant in Zanzibar town which could process surplus milk from farmers and increase shelf life has been closed. Milk marketing is already being regarded by the Ministry of Agriculture as a constraint to dairy development in the Island (Personal Communication with Dr. Kassim Juma).

Intervention by government and/or other parties interested in livestock development

(i) Encourage formation of farmer groups in the rural areas for input supply and milk marketing

(ii) Promote informal milk processing and marketing by private market agents

(iii) Establish milk collection centers in rural areas

The fact that household income averaged TShs 41,232 (US$ 52) per month with approximately 97% of the households reporting an average income of less than TShs 100,000 (US$ 125) is a reflection of the generally low income level of rural households in Zanzibar. This underlines the need for intervention and justifies streamlined efforts and increased investments for poverty elimination in rural areas of Zanzibar.

4.0. FRAMEWORK FOR ECONOMIC IMPACT ASSESSMENT OF TSETSE CONTROL/ERADICATION

In this section, we first explain why we need to assess the impact of a tsetse/trypanosomosis control/eradication programme. Next, we review some of the previous work on the economic impact of tsetse and trypanosomosis. This is followed by a discussion of the various components a framework should contain. The data requirements for applying the framework then follow.

4.1. Why a framework for impact assessment

To objectively evaluate the economic impact of a tsetse control/eradication programme and provide valuable and reliable information for decision-making, an analytical framework is required. However, before developing such a framework, it is essential to know why impact assessment should be carried out at all. This helps to determine the various factors to be considered, how each factor interacts with one another, what type of data should be collected for each defined parameter and how the data are to be used. It also helps to accommodate all the changes (direct and indirect) that have or are likely to occur (at farm or national level) as a result of the intervention.

Specifically, an impact assessment of a tsetse control/eradication intervention should answer three important questions. The first relates to the public investment nature of the intervention. Does or will the intervention generate sufficiently large benefits to justify the investments incurred? Answers to these questions are needed for decisions on future investments in other disease control/eradication interventions. Secondly, who are the main beneficiaries of the eradication intervention? To what extent do not only livestock and crop producers benefit from the absence of trypanosomosis, but also consumers from the increase in crop and livestock production?. Finally, what lessons can effectively be drawn from how the control/eradication intervention was or will be implemented? Providing answers to these questions requires that accurate data be collected on the relevant parameters; that appropriate procedures and methodologies be defined for analyzing the data; and that an analytical framework be developed to tie together all the relevant factors associated with the incremental changes attributable to the intervention.

4.2. Previous work

Trypanosomosis is a parasitic disease caused by the tsetse fly. The disease causes significant economic losses in livestock, notably cattle, sheep, goats, donkeys and pigs, leading to reduced meat, milk, manure and draught power. FAO (1994) estimates that annual losses in the potential of farm production due to trypanosomosis amounts to about US$ 4 billion with annual control costs ranging between US$ 600 million and US$ 1.2 million. WHO (1999) also estimates that over 55 million people are at risk of contracting human trypanosomosis with more than 300,000 people infected with "sleeping sickness". Before proposing a framework for deriving such estimates, a brief review of some of the impact assessment methods used in the past is in order.

Previous attempts to assess the impacts of the incidence of trypanosomosis on the productivity of animals have either compared the infection status of individual animals within a herd or have compared the level of risk among herds across different geographical areas. In a study of the epidemiology and incidence of trypanosomosis, Camus (1981a) tested over 3,000 animals in 191 cattle herds in the north of Ivory Coast for the rate of infection with trypanosomosis. Based on productivity data established on fertility and mortality parameters, he developed a static model for estimating losses due to trypanosomosis. After calculating the maximum extraction rates for each of four genotypes -- N'Dama, Baoule, N'Dama x Baoule and Zebu crossbreeds -- he then estimated the average annual losses per animal due to trypanosomosis as the difference between the potential production value of trypanosomosis-infected and non-infected cattle herds. Using this method, Camus (1981b) estimated the annual total losses attributable to trypanosomosis in northern Ivory Coast to be 92 million Francs CFA. While the work of Camus provided the basis for a methodology for economic assessment of trypanosomosis control, it failed to account for the dynamics of the cattle herd and its evolution over time. Far from taking a holistic view, the model only partially considered the outputs of the cattle sub-sector, neglecting the yet important output -- milk. Camus' framework therefore generated values that were grossly underestimated.

Brandl (1988) assessed the economic impacts of trypanosomosis control in cattle by the use of a herd simulation model. The model was superior to that of Camus by its very dynamic nature. The model simulated the development of a herd in a time sequence, on the basis of initial herd structure, productivity and management parameters as well as the physical and monetary outputs. With 24 age classes for female and male animals, 17 input parameters for each age class are specified for each of two scenarios involving *with tsetse control* and *without tsetse control*. Herd growth, milk offtake and animal offtake were estimated *ex ante*, and the profitability of tsetse control in terms of the benefits and costs associated with the use of the sterile insect technique to eradicate tsetse in the Sideradougou area of southern Burkina Faso were assessed. Brandl then compared a scenario involving tsetse eradication using the sterile insect technique with three other scenarios involving no tsetse control but with low, medium and high prevalence levels of trypanosomosis infection. Results of the herd simulation model showed that *without tsetse control*, the cattle population in the Sideradougou area would increase by less than 1% per year and between 2% and 5% per year *with tsetse control*. Based on these growth rates, he estimated that the presence of trypanosomosis would reduce milk off-take by 38% and animal offtake by 5% to 3.1%. By applying results of the herd model to alternative control methods, Brandl (1988) concluded that for smaller areas of application, the sterile insect technique would only become profitable with medium and high losses due to trypanosomosis and with calculation periods of over 15 years.

Putt *et al.* (1980) have provided one of the most comprehensive economic assessments of the costs and benefits of tsetse control operations in northern Nigeria. By using statistics of trypanocidal treatments augmented by the expert opinions of veterinarians and local stock owners, they estimated the costs and benefits of tsetse eradication under two levels

of assumptions. The first assumption was that tsetse eradication projects are only justifiable if the constraints they remove are of sufficient importance that the opportunities they create are picked up by people and secondly, that people would apply the same agricultural and livestock techniques after tsetse clearance as they did before. This assumption however applied strictly for rare situations like Northern Nigeria where after tsetse was cleared, people maintained the same traditional livestock production techniques. The more likely situation is that after tsetse has been cleared in an area, people will likely adopt some mixed farming practices, limited integration of crop and livestock production and sedentarization of livestock. This situation as pointed out by Putt *et. al* (1980 pp 443-444) is more likely to occur for most of Africa and the survey findings in Zanzibar certainly reflect this likely situation.

Drawing from results of field studies conducted under contrasting disease and production situations across Africa, Swallow (1999) developed a dynamic framework for measuring the impacts of trypanosomosis on agriculture. Following the classification of Putt *et al.* (1980), Swallow distinguished between the direct and indirect impacts of the incidence of trypanosomosis and those of trypanosomosis risk. He showed that the direct impacts of the incidence of trypanosomosis could be measured from morbidity, mortality, work efficiency of animals and the costs associated with the treatment of trypanosomosis-diseased animals whereas the direct impacts of trypanosomosis risk could be derived from the management activities that livestock farmers undertake because of the risk to which their animals are, or could be, exposed to. The indirect impacts on the other hand, of trypanosomosis incidence, could be measured as the changes that occur in human settlement, crop production and land use attributable to the reduced productivity of existing animals while the indirect impacts of trypanosomosis risks could be measured as the changes in human settlement and crop production that occur because of the livestock management practices followed by farmers who raise livestock under trypanosomosis risk. Applying the framework, Swallow (1999) concluded that trypanosomosis directly constrains the productivity of cattle, sheep and goats by reducing birth rates, increasing abortion rates, and increasing mortality rates among young animals; making the production of meat, milk and animal traction by existing animals less efficient. In mixed crop-livestock production systems Swallow (1999) notes, the impact of trypanosomosis on the number and work efficiency of oxen has repercussions on crop production with estimated reductions in total agricultural production varying from 2% to 10%.

The important point about the preceding studies is that a framework for economic impact assessment should account for all the effects, direct as well as indirect, attributable to tsetse and trypanosomosis. Such a framework is provided in Figure 2. It suggests that the impact of tsetse/trypanosomosis should be viewed and assessed in a wider context of the farming system and economy, being cognisant of the varied and complex interrelationships among the system's physical, biological and socio-economic components.

Figure 2. Framework for economic impact assessment of tsetse control and eradication

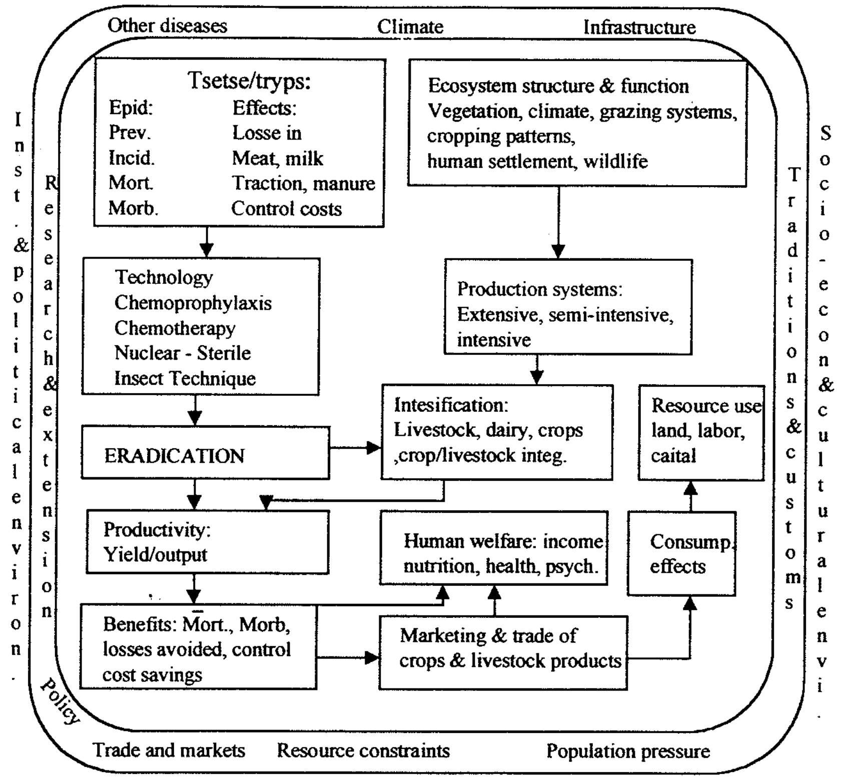

Biophysical environment

Fig. 2. Framework for economic impact assessment of tsetse control/eradication.

4.3. Important considerations for developing a framework

The type of effects to be quantified are the following:

4.3.1. Direct losses

For Zanzibar where human trypanosomosis is not an issue, the direct economic effects of animal trypanosomosis arise from:

(i) mortality losses incurred;

(ii) morbidity losses experienced; and

(iii) the costs of control (treatment and prophylaxis) and/or eradication.

The consequences of the direct losses are sometimes difficult to value in economic terms. It may be easy to value the death of an animal but it may not be easy to quantify and value the effects of poor performance due to increased rates of abortion or poor growth rates due to anaemia. Trypanosomosis-infected animals are sometimes subject to the influences of other animal diseases as well as malnutrition, making it difficult to isolate the productivity effects of trypanosomosis. Also, the death of an animal and the magnitude with which its productivity is affected by trypanosomosis may well depend on the level of trypanosomosis risk to which it is exposed and whether or not it is trypanotolerant or well managed.

The direct costs of treating animals by chemotherapy, protecting them from infection by chemoprophylaxis or total elimination of the vector by mechanical or nuclear means provides a good indication of the direct consequences of trypanosomosis. However, the absence of proper diagnosis and optimal treatment methods sometimes make it difficult to obtain accurate estimates of the benefits and costs. Occasionally, costs become overstated when there is blanket treatment of herds in which not all animals are infected and understated when infected animals go untreated. These facts should be taken into consideration by any framework designed to assess economic impact.

4.3.2. Indirect losses

What is of greater significance than the direct losses due to trypanosomosis but more difficult to value in economic terms are the indirect losses which arise from:

(i) Constraints on the production of cattle, sheep, goats and donkeys (including some wildlife);

(ii) constraints placed on land use for crop and livestock production; and

(iii) limitations placed on crop-livestock integration.

These constraints are due to peoples' perceptions of the risk of their animals contracting trypanosomosis and their adoption of production systems that fail to make use of the natural resources available in tsetse infested areas. In areas of high challenge, livestock keeping is highly precluded and therefore the indirect consequences of trypanosomosis are most evident. Because land use in areas of high trypanosomosis risk is avoided (mostly when human sleeping sickness is concerned) and only restricted to periods of low risk, optimal use of natural resources is reduced, thus making the indirect economic losses far more important than losses due to mortality and morbidity.

Successful control/eradication of tsetse infestations removes the direct losses and permits more efficient utilization of natural resources, thus giving rise to a number of direct and indirect benefits. Among the direct benefits are the mortality and morbidity losses avoided and the savings in control and treatment costs. These benefits clearly depend on the level of trypanosomosis challenge because areas with low challenge are usually well utilized by livestock whereas the high challenge in other areas often precludes livestock and even crop production. The benefits to be quantified and valued include:

4.3.3. Direct benefits

4.3.3.1. Mortality losses avoided

Complete eradication of tsetse from an area clears the cattle and other livestock species of the trypanosome parasite thus eliminating the possibility of an animal getting sick and eventually dying from trypanosomosis. The product gained by preventing the death of an animal constitutes the benefit arising out of the mortality avoided as the disease is eradicated. The value of a lost animal to the livestock owner due to death or premature slaughter is to be measured in terms of the market price that the owner would have obtained under conditions of good health.

4.3.3.2. Morbidity losses avoided

Livestock infected with the trypanosome parasite experience a reduction in productivity due to a loss in condition, lowered milk production, decreased fertility, abortion and a reduction in work force. Because of the varying response of individual animals to infection, these productivity parameters are to be quantified and valued with care. Farm level data on these parameters may be difficult to obtain. However, they can be estimated under experimental conditions.

4.3.3.3. Control and treatment costs saved

Several methods have been used in Zanzibar to control and eradicate tsetse and trypanosomosis. Bush clearing was widely practised during the 1950s and 1960s, particularly in Mangapwani. During the 1970s and 1980s, a combination of various

control methods involving insecticide impregnated screens, bait-oxen fly-rounds and biconical traps were used. Chemoprophylactic measures were applied and infected cattle were treated with trypanocidal drugs. Following the suppression of apparent fly densities by the various control methods, the Sterile Insect Technique was used to finally eradicate the disease in 1997. With eradication, these control measures are no longer being undertaken, leading to important benefits from the savings in control costs.

4.3.4. Indirect benefits

Indirect benefits occur when eradication of tsetse and trypanosomosis removes constraints on land, making it available for use by people. The changes that occur in the use of the land constitute the benefits, which only become evident when (i) the land is used for livestock production; (ii) the land is arable and is used for crop production; and (iii) interactions occur between livestock and crop production activities leading to mutual benefits.

4.3.4.1. Constraints on livestock production eliminated

Eradication of tsetse flies from an area creates opportunities for increased carrying capacity as livestock utilize the fodder and water resources which otherwise would not be accessible to animals in the presence of tsetse flies. It also avoids the losses in livestock that would have been incurred in their attempt to utilize the resources in the tsetse infested areas. These benefits are best measured in terms of the additional meat, milk, hides and skins, manure and work force from animals resulting from the additional carrying capacity. The magnitude of the benefits however, depend on (i) the presence of fodder and water resources in the tsetse free areas and (ii) the extent to which people move cattle and other livestock into the area to utilize the resources. To determine the additional carrying capacity, data is needed on (i) total land made available following tsetse eradication; (ii) the proportion of land that is suitable for use by animals; (iii) percentage of stock owners making use of the tsetse free area; and the number of animals making use of the resources in the area. Taking all of these factors into account may not truly reflect the value of the benefit realized from an animal that makes use of the additional carrying capacity. What matters in the final analysis is data on the value of the animal at sale.

4.3.4.2. Constraints on agricultural production eliminated

The extent to which agricultural production is constrained by tsetse flies in Unguja is limited because of the absence of human sleeping sickness, the risk of which often keeps people away from cultivating land in the infested areas. There is no evidence in Unguja that people have abandoned the cultivation of arable land because of tsetse flies. Rather, those agricultural production activities that go together with raising cattle, donkeys and small ruminants have been constrained because of the fear of these animals contracting trypanosomosis. Quantifying the benefits of eliminating constraints on agricultural production therefore depends on the ability to isolate the avoided losses in crop production.

4.3.4.3. Constraints on livestock-crop integration eliminated

Elimination of tsetse and trypanosomosis from an area enhances greater integration between livestock and agricultural production as cattle, sheep, goats and donkeys are allowed to remain in the arable areas being cultivated. As the animals make use of crop residues and by-products, they in turn provide manure and other animal products (e.g. meat, milk, hides and skins, animal power) to the farmer. As animal manure improves soil quality, crop yields increase, leading to greater agricultural output. This depends however, on (i) the symbiotic relationship between stock owners and farmers and how they are willing to co-exist with each other; and (ii) how well farmers and stock raisers adopt technologies that promote increased use of crop residues and by-products as animal feed, manure for soil fertility improvement and animal traction for plowing and transport. To value these benefits, data are needed on farm sizes, proportion of farmers integrating livestock and agricultural activities, percentage of farmers adopting animal traction for plowing and traction.

4.4. Application of the framework to economic analysis

The economic impact of a disease control/eradication intervention is assessed in relation to the value of the outcome without the intervention. Returns or benefits derived from the intervention represent the additional earnings realised if the intervention takes place compared to what would or would have happened in its absence. Benefits and costs of the intervention are measured as the incremental changes between the intervention and the non-intervention scenario. Benefit-cost analysis then compares the value of the benefits with the value of the costs as a guide to determine whether the intervention is economically superior to the alternative scenario.

There are generally two types of costs associated with a disease control/eradication intervention: (i) Costs incurred to control/eradicate the disease that would not have been incurred in the absence of the intervention; and (ii) any livestock production revenues foregone that are no longer earned due to the control/eradication measures. There are also two types of benefits from disease control/eradication: (i) Increased revenue from improved productivity, i.e. revenue due to avoided production losses from mortality and morbidity, as well as (ii) savings in control costs avoided after achieving eradication. Incremental benefits are estimated as the difference between production value obtained "with" intervention versus the "without" scenario. The "with" scenario represents what actually obtains with the intervention and so is usually based on available data. The "without" scenario, on the other hand, describes what was happening "before" the intervention or would have happened "after" had the intervention not taken place.

4.4.1. Data needs for estimating mortality losses

It is useful to spell out the data variables in more detail as a guide for compiling sufficient data for assessing economic impact.

Total cattle population (same for sheep, goats and donkeys if important in the system)
The following variables for calves, immature cattle and adult cattle:
Cattle population under risk: types, numbers, by production system
% of cattle affected by trypanosomosis
% of cattle dead (mortality) due to trypanosomosis (calves, immature and adult cattle)
% of cattle dead from other animal diseases (calves, immature and adult cattle)
Prevalence: in calves, immature and adult cattle
Incidence: in calves, immature and adult cattle
Mortality: in calves, immature and adult cattle
Life weight (kg/head)
Milk yield (liters/per cow per year)
Beef yield (kg carcass/head/year)
% of breeding females
Calving rate (%)
Manure production (kg/head/year)
Hectares worked (hours/draught animal/year)
Off-take rates (%) in calves, immature and adults per year
Farm gate prices for beef, milk, lamb and mutton, draught animal, manure

4.4.2. Data needs for estimating morbidity losses

Total cattle population (same for sheep, goats and donkeys if important in the system)
The following variables for calves, immature cattle and adult cattle:
Cattle population under risk: types, numbers, by production system
% of cattle affected by trypanosomosis
Prevalence: in calves, immature and adult cattle
Incidence: in calves, immature and adult cattle
% of life weight reduction due to trypanosomosis
% of milk reduction due to trypanosomosis
% reduction in work force due to trypanosomosis
% reduction in manure due to trypanosomosis
Age at first calving (months)
Calving interval (months)
Calving rate (%)
Calf/cow ratio
Lactation length (days)
Abortion rate (%)
Farm gate prices for beef, milk, lamb and mutton, draught animal, manure

4.4.3. Data needs for estimating control/eradication costs

Capital investment costs

Buildings (administrative and factory)
Radiation equipment and accessories
Equipment (office, laboratory, factory, etc.)
Airplanes
Vehicles
Traps and targets

Recurrent costs

Labor costs (salaries, wages, allowances)
Building running and maintenance (water, electricity, etc.)
Vehicle and airplane running and maintenance (fuel, spare parts, lubricants, etc.)
Quantities used of insecticides, chemicals and drugs and their prices
Trap maintenance and checking
Packaging materials
Administrative costs (e-mail, telephone, fax, etc.)

4.4.4. Data needs for estimating indirect losses on livestock and agriculture production

Total national land area
% of total land area infected by tsetse flies
% of land area abandoned due to tsetse flies
% of total land area cleared of tsetse flies
% of cleared land that is arable
% of cleared land fit for grazing
% of cleared land used by cattle (and other livestock species)
% of cleared land cultivated for crops
Number of farmers acquiring new land tsetse cleared areas
Number of farmers cultivating cleared area
Number of farmers grazing cattle in cleared area
% of cattle farms with infected cattle
% of cattle farms with infected cattle losing cattle due to trypanosomosis
% of herd size infected by trypanosomosis (indigenous and improved cattle by age and sex structure)
% of infected cattle dead due to trypanosomosis (indigenous and improved cattle by age and sex structure)
% of farms with milking cows (indigenous and improved cattle)
% of farms with milking cows infected by trypanosomosis (indigenous and improved cattle)

% of farms with infected milking cows having a reduction in milk production (indigenous and improved cattle)
% of farms using crop residues and by-products as animal feed
% of farms feeding concentrates and mineral supplements to cattle
% of farms using animal manure for soil fertility improvement
% of farms using animal power for plowing and transport
% of adult cattle used for traction (oxen)
Crop area prepared per animal per year
Crop yields per hectare
Farm-gate prices of crops cultivated
Reduction in traction due to the disease per traction animal per
% of farms adopting improved breeds of cattle (and other livestock species)

4.4.5. Other data needs

Discount rate (%)
Exchange rate (TSHS per 1US$)
Elasticities of demand and supply for various livestock products and crops
Shadow prices of livestock products and crops
Quantities of livestock products and crops imported and exported
Value of livestock products and crops imported and exported
Total human population
Gross Domestic Product (GDP)
Agricultural GDP
Livestock GDP

4.4.6. Calculations required

4.4.6.1. Mortality and morbidity losses

Beef loss:

Land area covered by cattle at risk of trypanosomosis
Proportion of land covered by cattle at risk, %
Cattle at risk of trypanosomosis, %
Herd in trypanosomosis area at year start
Number of breeding females
Number of calves born during the year
Herd size affected by trypanosomosis during the year
Number of calves affected by trypanosomosis
Number of calves dead due to trypanosomosis
Number of adult males in the herd
Number of immature cattle in the herd
Number of immature cattle with trypanosomosis
Number of immature cattle dead due to trypanosomosis

Number of adult cattle in the herd
Number of adult cattle with trypanosomosis
Number of adult cattle dead due to trypanosomosis
Total cattle dead due to trypanosomosis
Calf beef loss due to trypanosomosis mortality
Immature cattle beef loss due to trypanosomosis mortality
Adult cattle beef loss due to trypanosomosis mortality
Total beef loss due to trypanosomosis mortality
Number of calves morbid from trypanosomosis
Calf beef loss from morbidity
Number of immature morbid cattle
Immature beef loss due to morbidity
Number of adult cattle morbid from trypanosomosis
Adult cattle beef loss due to morbidity
Total beef loss due to trypanosomosis morbidity
Total herd beef loss due to trypanosomosis
Farm gate value of beef loss
National value of beef loss

Milk loss:

Number of milking cows with trypanosomosis
Survival rate of milking cows with trypanosomosis
Number of milking cows surviving trypanosomosis
Quantity milk reduction from trypanosomosis morbidity
Number of milking cows dead due to trypanosomosis
Quantity of milk lost from dead cows
Total milk loss due to trypanosomosis
Farm gate value of milk loss
National value of milk loss

Animal traction loss

Number of adult cattle used for traction
Reduction in traction days due to trypanosomosis
Traction hectares reduced due to trypanosomosis
Farm gate value of traction hectares lost
National value of traction hectares lost

Manure loss

Quantity of manure lost due to dead calves, immature and adult cattle
Quantity of manure from calves reduced due to trypanosomosis

Quantity of manure from immature reduced due to trypanosomosis
Quantity of manure from adult cattle reduced due to trypanosomosis
Total manure loss due to trypanosomosis
Farm gate value of manure loss
National value of manure loss

Total loss due to trypanosomosis:

Total mortality and morbidity losses
Total cost of trypanosomosis control/eradication
Total farm gate value of output loss from trypanosomosis
Total national cost of trypanosomosis
Trypanosomosis loss per head of cattle
Trypanosomosis loss per hectare in trypanosomosis area

4.4.6.2. Economic value of livestock and agriculture production under conditions of trypanosomosis

Livestock

Total calves net of trypanosomosis mortality
Calves net of total mortality
Number of calves for disposal
Immature cattle net of trypanosomosis mortality
Immature cattle net of total mortality
Number of immature cattle for disposal
Adult cattle net of trypanosomosis mortality
Adult cattle net of total mortality
Number of adult cattle for disposal
Total number of cattle for disposal
Beef production from calves
Beef production from immature cattle
Beef production from adult cattle
Total beef production
Gross national value of beef production
Milking cows net of trypanosomosis mortality
Milking cows net of total mortality
Total milk production
Gross national value of milk production
Number of hectares of animal traction
Total national income from animal traction
Total manure production
Total national value of manure production
Quantity of hides produced

Total national value of hides
Net cattle population at year end
Herd growth, heads of cattle
National value of herd growth
Total gross national value of output
Total value of cattle at year 1 start and year X end
Total costs of production
Total net farm income from cattle in trypanosomosis area
Productivity per cattle head in trypanosomosis area
% change in herd size
Year-end carrying capacity, ha/head
% change in carrying capacity
Carrying capacity in trypanosomosis area

Crops

Total number of crop farms cultivated in tsetse cleared areas
Total land area cultivated in tsetse cleared areas
Total quantity (of each crop) produced in tsetse cleared areas
Value of crops produced in tsetse cleared areas
Quantity of forage, crop residues and by-products produced in tsetse cleared areas
Value of forage, crop residues and by-products produced in tsetse cleared areas
Crop productivity per ha in trypanosomosis cleared areas

4.4.6.3. Decision criteria

Benefit-cost ratio
Net present value
Internal rate of return
Total welfare gains
Producer welfare gains
Consumer welfare gains

5.0. Summary and conclusion

The purpose of this assignment was to collect baseline data on the livestock and agriculture situation in Unguja, Zanzibar before the initiation of tsetse/trypanosomosis control/eradication in 1985/86 and the current situation in 1999. While serving as inputs for a future economic impact assessment of tsetse/trypanosomosis control/eradication, the data were to be used to (i) describe the developments in livestock and agriculture that have occurred since the initiation of the intervention in 1985/86 and (ii) predict future developments in livestock and agriculture production. To enable the economic impact assessment to be carried out, a framework of analysis was to be developed.

The approach adopted consisted of a review of the relevant documents on tsetse/trypanosomosis in Zanzibar, collection of data on relevant parameters from these secondary sources; field visits involving informal discussions with key informants and other stake holders; design of a field survey using a formal questionnaire; and data compilation, analysis and report writing.

Based on the informal discussions, the secondary and primary data gathered, there is the general conclusion that the livestock and agriculture situation in Unguja, Zanzibar in 1999 has changed significantly from what it was before the initiation of tsetse/trypanosomosis control/eradication in 1985/86. The overall change is evidenced by increased growth in the livestock sub-sector relative to the crops sub-sector. Livestock contribution to GDP for example, increased from 12% in 1986 to 34% in 1997 whereas the share of crops in GDP dropped from 81% in 1986 to 59% in 1997.

Whereas areas cultivated for crops such as cassava, sweet potatoes, tania (cocoyams), maize and plantain increased or remained the same after 1985/86, yields obtained for these crops declined, suggesting a decline in productivity of land used to produce these crops. Improvements have however, been observed for some crops. Areas cultivated for cloves, yams and coconuts declined after 1985/86, but the yields obtained increased, suggesting that productivity of these crops has improved. This is not surprising especially for cloves as there has been deliberate efforts by the government to revive cloves production in Zanzibar. With regard to livestock, not only has the relative proportion of farms raising cattle and small ruminants increased in 1999 compared to 1985/86, the number of farms with improved cattle breeds has also increased, making it possible for improved cattle to supply 35% of the total milk production compared to about 10% before the intervention. Evidence for intensification of livestock and agricultural activities in Unguja is provided by the increased crop-livestock integration through more farmers using manure for their crop production while in turn using crop by-products to feed their animals. The use of animal power for ploughing and transport activities is also increasing. While several factors may have contributed to the achievement of these results, there is general consensus among people in Zanzibar that the eradication of tsetse and trypanosomosis in 1997 has resulted in significant gains in the livestock sub-sector. Zanzibar's Agricultural Sector Review of 1999 indicates that the IAEA sponsored tsetse-eradication project produced some of the

best successful results of donor funded projects in East Africa. The information in this report provides some support to this claim.

Although the total production of livestock and agriculture has been projected to increase in the near future, per capita consumption of major livestock and crop products generally fall short of the planned targets. Current production trends indicate that the domestic consumption needs may not be met. Imports will continue to be needed to fill the deficit. However, continued reliance on food imports cannot be sustained and could impact negatively to both livestock and agricultural production. Deliberate efforts must be made by Zanzibar authorities in collaboration with private sector and other stakeholders to tackle constraints which are affecting the livestock and agricultural sector. These constraints include poor livestock and crop extension services, processing and marketing of crop and livestock products, diseases and low usage of inputs and improved crop and livestock varieties. Provision of affordable and smallscale oriented credit facilities could greatly improve livestock and crop production.

This report does not purport to have assessed the economic impact of tsetse eradication in Unguja, Zanzibar. Rather, it only provides data on some key parameters and proposes a framework that could be used to carry out an economic impact assessment.

References

Baumhover, A.H., A.J. Graham, B.A. Bitter, D.E. Hopkins, W.D. New, F.H. Dudley and R.C. Bushland, 1955. "Screw-worm control trhough release of steilized flies". *Journal of Economic Entomology*, 48: 462.

Bouvry Stratford M. 1986. Cattle trypanosomiasis situation on Zanzibar (URT/81/017) FAO Field document No. 7. Zanzibar, 21 pp.

Brandl, E.F. 1988. *Economics of trypanosomiasis control in cattle*. Farming Systems and Resource Economics in the Tropics, Vol. 1. Wissenschaftsverlag Vauk Kiel, Germany.

Camus, E. 1981a. Epidemiologie et incidence clinique de la trypanosomose dans le nord de la Cote d'Ivoire. Rev. Elev. Med. Vet. Pays Trop.., 34 (3): 289-295.

Camus, E. 1981b. Evaluation economique des pertes provoquees par la trypanosomose sur quatre types genetiques bovins dans le nord de la Cote d'Ivoire. Rev. Elev. Med. Vet. Pays Trop.., 34 (3): 297-300.

FAO (1994). Opening Address of Y. Cheneau in: A systematic approach to tsetse and trypanosomosis control; Proceedings of the FAO Panel of Experts; held in Rome, Italy, from 1 to 3 December 1993. FAO Animal and Prod. Health Paper, 121, 1-3.

FAO (Food and Agriculture Organisation), 1987. Livestock Development Phase III. Project findings and recommendations. UNDP/FAO Project DP/URT/81/017. Rome 49 pp.

GURT/FAO/IAEA (Government of United Republic of Tanzania/Food and Agriculture Organisation/International Atomic Energy Agency), 1994. Tsetse Fly Eradication on Zanzibar. Project Document URT/5/016, Zanzibar.

Hafidh, H.S., Khamis, I.S., Salum, S.H. and Stratford, M.B. 1987. Background data for the control of cattle trypanosomiasis on Zanzibar, Tanzania. Report prepared for the Ministry of Agriculture and Livestock, Zanzibar. FAO Project URT/81/017 Field document No. 10. 43 pp.

IAEA/FAO (International Atomic Energy Agency/Food and Agriculture Organisation), 1997. Eradicating the Tsetse Fly on Zanzibar Island.

Jordan. A.M. 1986. *Trypanosomiasis control and African rural development*. Longman Publishers, London. pp. 223-242.

Kadigi, R.J. 1995. Industry review of ruminant livestock. Ministry of Agriculture, Dar es Salaam, Tanzania.

Knippling, E.F. 1960. "The eradication of the screw-worm fly". *Sci. Amer.*, 203: 54-61.

Langley, P.A., H. Maly and F. Ruhm, 1972. "Application of the sterility principle for the control of the Mediterranean fruit fly (Ceratis capitata): Pupal metabilism in relation to mass-rearing techniques". *Ent. Exp. Appl.*, 15: 23-34.

URT (United Republic of Tanzania) Statistical Abstracts. Department of Statistics, Ministry of State Planning and Investment, Zanzibar. 1997 and previous years.

Molyneux, D.H., 1977. Vector relationships in the trypanosomatidae. Advances in Parasitology, 15, 1-82.

Muller, P. and Nagel, P. 1994. Tsetse fly eradication on Zanzibar Island. Environmental surveys. FAO Project. URT/5/016-07-01. Rome, 97 pp.

Putt. S.N.H., A.P.M. Shaw, R.W. Matthewman, D.M. Bourn, M. Underwood, A.D. James, M.J. Hallam and P.R. Ellis. 1980. *The social and economic implications of trypanosomiasis control: A study of its impact on livestock production and rural development in northern Nigeria.* Study No. 25. 549 pp.

RGZ (Revolutionary Government of Zanzibar), 1998. Five year post tsetse eradication development programme in Zanzibar. Ministry of Agriculture, Livestock and Natural Resources, Commission of Livestock and Agriculture, Zanzibar.

RGZ (Revolutionary Government of Zanzibar), 1999. Agriculture Sector Review. Report prepared by Food and Agriculture Organisation of the United Nations. April, 1999.

Schonefeld, A.A. 1988. Pilot trial for the control of *Glossina austeni* on the Island of Zanzibar. FAO Project TCP/URT/6758. Assistance in Trypanosomiasis Control, Zanzibar, Rome, 33 pp.

Spharim, Y. 1974. Economic feasibility of establishing a pilot plant for control of the Mediterranean fruit fly by distribution of sterile males. IAEA-SM-186/22: 151-158. International Atomic Energy Agency, Vienna.

Swallow, M.B. 1999. Impacts of trypanosomosis on African Agriculture. Paper presented at the International Scientific Council for trypanosomiasis Research and Control, Maputo, Mozambique, Sept. 29 - Oct. 4, 1997.

Turner, D.A. 1984. The distribution of the tsetse fly, Glossina austeni Newstead on Zanzibar (Unguja) Island, Republic of Tanzania: A report of a survey carried out between November 1983 and February 1984. IAEA Report.

URT (United Republic of Tanzania), 1998. Five year post-tsetse eradication livestock development programme in Zanzibar. Agriculture and Livestock Commission, Zanzibar.

UNDP/FAO, 1993. Animal Disease Control Project, Zanzibar. The URT Project findings and recommendations. Rome, 25 pp.

WHO, (1999). World Health Organization, Division of Control of tropical Diseases (CTD). Internet Home Page of 12 June 1999. http://www.who.int/ctd/html/trypanoburtre.html

Wizara ya Kilimo, Mifugo na Mali Asili (1999) Ripoti ya Mwaka 1998. February 1999.

Zanzibar Integrated Land and Environmental Management Project (1995). National Land Use Plan.

ANNEX 1

Table A 1.1. Total, per capita and agricultural Gross Domestic Product in Zanzibar, 1980 to 1997.

Year	GDP at current prices						GDP at constant 1976 prices		
	Total<br>Million TShs	Per capita<br>TShs	Agricultural<br>Million TShs	Agric. as % of GDP	Livestock as % of agric. GDP	Crops as % of agric. GDP	Total<br>Million TShs	Per capita	Agricultural<br>Million TShs
1980	1,431	2,849	654	45.7			624	1,242	285
1982	2,229	4,206	1,201	53.9			784	1,479	423
1984	2,870	5,134	1,613	56.2			685	1,225	385
1986	4,767	8,927	1,816	38.1	12.2	81.0	734	1,375	280
Average annual[1]	2,756	4,998	1,272	46.0			726	1,329	334
1988	9,412	16,421	4,433	47.1	22.2	72.0	768	1,340	362
1990	16,921	27,903	7,919	46.8	15.3	77.5	815	1,344	381
1992	34,698	54,965	13,012	37.5	22.7	69.2	880	1,394	330
1994	56,537	84,766	19,279	34.1	28.9	64.5	942	1,228	321
1996	84,225	110,775	32,342	38.4	32.6	61.2	1,038	1,365	398
1997	99,208	126,266	37,997	38.3	33.8	59.4	1,089	1,386	417
Average annual[2]	41,437	59,432	16,184	39.0	24.0	67.3	893	1,350	349

[1] Includes all the years from 1980 to 1986

[2] Includes all the years from 1987 to 1997

ANNEX 2.

Table A 2.1. Names of villages sampled.

North A	North B	West	South	Central	
Moga	Mangapwani	Mfenesini	Pete	Charawe	Ghana
Mkokotoni	Fujoni	Mwakaje	Muungoni	U. Kaeboni	Tunduni
Pale	Zingwezingwe	Bumbwisudi	Kitogani	U. Kaepwani	Mgeni haji
	Kiomba mvua	Mwachealale	Muyuni A	Bungi	Pagali
	Mkadini	Mbuzini	Muyuni B	Kikungwi	Bambi
	Kitope	Fuoni	Muyuni C	Ndijani	Cheju
	Kilombero	Kimara	Kibuteni	Kiboje	Jendele
	Muwanda	Kizimbani	Makunduchi	Machui	Kisomanga
	Donge Mtambile	Dole		Mchangani	Umbuji
	Donge Kipande	Kianga			
	Mahonda				

ANNEX 3

Table A 3.1. Number of sampled villages and households.

Districts	Cattle population (1993)	Human population (1995)	Number of villages (1987)	Number of Households (1995)	Number of villages infested with tsetse flies, 1985/86	Number of villages to be sampled	Number of households to be sampled
North A	8,465	70,000	27	11,290	1	3	68
North B	6,849	44,000	23	7,097	13	11	266
Central	12,743	60,500	36	9,758	31	18	414
South	3,406	27,500	17	4,435	10	8	198
Urban	1,164	203,000	1	32,742	0	0	0
West	13,132	72,000	21	11,613	9	10	254
All Districts	45,750	477,000	125	76,935	64	50	1,200

ANNEX 4

Instructions and explanations to enumerators accompanying questionnaire for the economic impact assessment of tsetse control and eradication in Zanzibar Island

Prior to completing each questionnaire, the enumerator must establish whether the person being interviewed is the household head or the major decision maker. If none of these is available the questionnaire should not be completed for that household. The enumerator is expected to have properly understood each question in the questionnaire.

Upon meeting the farmer the enumerator should explain the purpose of the survey and request the farmer's permission to have a discussion with him/her. Seek for his/her patience and cooperation during the exercise. Do not read each question to the farmer. Rather, endeavor to have a friendly, yet constructive dialogue with the farmer while tactively trying to fill in the responses. Do not make any promises to the farmer. During the interview if there are any answers that do no respond adequately to the question(s) being asked, probe further until you establish that the question has been adequately responded to. If the farmer does not seem to understand the question as it is, devise an alternative way of phrasing the

question such that the message gets to the farmer. Also, if the respondent fails to remember the answer to a question relating to the period before or during tsetse intervention, refer to particular events at that time. Ask if an extension agent ever visited his/her farm at that time and see if he/she can remember.

The first ten questions pertain to the household head or the household decision maker. They seek to obtain general information on household characteristics and are quite straight forward.

Questions 11 to 55 deal with the livestock enterprise as a whole. These questions seek to establish the status of livestock development at the farm level from when the farmer began livestock production to the present. They also seek to explore the future potential of livestock development with particular emphasis on the use of improved livestock genetic resources, especially cattle, small ruminants and poultry. As well, they seek to establish livestock productivity parameters, animal disease situation with emphasis on tsetse and trypanosomosis, feeding and management practices.

Questions 11 and 12 are intended to establish the proportion of farmers who were involved in livestock production before and after the tsetse control and eradication program and the respective livestock numbers during those periods. They also want to explore reasons for changes in livestock numbers. The enumerator should complete the table in **question 12** paying particular attention to the numbers of each livestock species at the time the farmer began the livestock enterprise and the current situation. The enumerator should use his personal judgement to determine whether what the farmer intends to have in the future is realistic. It should be guided by his present resources.

Questions 13 to 15 seek to establish the availability, sources, costs and the use of improved livestock species.

Questions 16 to 20 are self explanatory.

Questions 21 to 31 are intended to establish farmers' knowledge of tsetse and trypanosomosis, their control strategies, costs, risk avoidance strategies and the potential for development in view of tsetse eradication. **Questions 21 to 23** attempt to determine the proportion of farmers who were affected by tsetse and trypanosomosis, how they responded to the disease and when the last case was observed. Bearing in mind that tsetse was eradicated in 1997 and laboratory results indicate that no case of animal trypanosomosis has been detected so far since then, any response by a farmer to part two of **question 23** after 1997, the farmer should be noted and questioned further. Such farmers will need to be followed up later. Recognizing that no data exist in Unguja on the incidence, morbidity and mortality due to trypanosomosis **question 24** seeks to establish these parameters. **Question 30** seeks to establish the proportion of farmers who have moved into or are making use of areas cleared of tsetse.

Questions 31 to 47 are specific to the dairy enterprise because dairy production offers more possibilities for increased intensification and therefore contributing to food security. These questions seek to establish the pre- and post-tsetse eradication productivity parameters for dairy. They also seek to establish the implications of tsetse eradication on the current status of dairy production and the future potential for development. For **question 32** the enumerator should establish the type and size of containers used by the farmer for measuring milk. For question 35 the enumerator should specify the age of the cow during its first calving in years or months which ever is applicable. For **question 45** the enumerator should establish that the farmer is using the feed material on a regular basis and not just once or occasionally.

Questions 47 to 54 are meant to establish crop-livestock interactions through the use of manure for crop production and pasture improvement, use of crop by-products for animal feed and the use of animal power. To complete the table in **question 48**, the enumerator should first of all establish whether the manure is used in fresh form of dry. Then ask for the type of containers used by the farmer, make an assessment of its weight when filled with manure and find out the number of fully filled containers used for each purpose

Questions 56 to 66 deal with crop production and seek to establish the past, present and future potential of the crop production system at the farm level. They also attempt to establish the implications of tsetse eradication on current crop production and the future potential. Allusion is made to the fact that as tsetse become eradicated, increased intensification will call for the use of the improved varieties. Therefore it is necessary to establish farmers' access to and use of improved plant genetic material in **questions 60 and 61. Questions 62 to 66** are intended to explore the extent to which farmers are already and will take advantage of the tsetse cleared areas for crop production. For question 57 the enumerator should first of all establish the type and size of container used by the farmer for measuring the output from each crop. The enumerator should also use his/her judgement to establish the size of the plot by physical inspection where possible. It should be stated whether the plot size is in acres or hectares.

ANNEX 5

Table A 5.1. Enumerators used during the survey.

No.	Enumerator name	Qualification	Year of experience
1	Mashavu Yahya Ali	Certificate in agriculture & livestock Production (CALP)	6
2	Abdulla Moh'd abdulla	Certificate in Animal health & production	16
3	Moh'd Ali Ameir	Diploma in Range Management	
4	Fatma w. suleiman	Diploma in Animal Health	
5	Maryam R. Mzee	Certificate in Animal health & Production	8
6	Abubakar Hassan Kisoma	Diploma in Animal Production	11
7	Silima Hassan	Diploma in Crop Production	19
8	Salum Mwinyi	Certificate in agriculture & Livestock Production	11
9	Amos Lamek	Diploma in horticulture	19
10	mussa Salum Abdulla	Certificate in agriculture & Livestock Production	4
11	Daudi Ramadhani	Certificate in agriculture & Livestock Production	18
12	Juma Omar	Diploma in Lab. tech. Chemistry	19
13	Sudi Nahoda Hassan	Msc. Agronomy	15
14	Abdul Rahim Hamid	Diploma in Animal Production	19
15	Said Kassim Yussuf	Certificate in AI. ,& Animal Health	22
16	Abeid Khamis Ramad'n	Diploma in Animal Health	18
17	Juma Ramadhan Juma	Certificate in agriculture & Livestock Production	4
18	Ishaka Abdul Wakil	Msc. Agronomy	15
	**Name of field supervisor**	**Qualification**	
1	Khalfan M. Saleh	Diploma in tsetseControl	12
2	Dr.Waridi Abdulla Mussa	Doctor of veterinary Medicine	13

ANNEX 6

Fig. Prevalence of trypanosomosis in Jozani forest (south district) and Mgeni Haji (central district) of Unguja, Zanzibar.

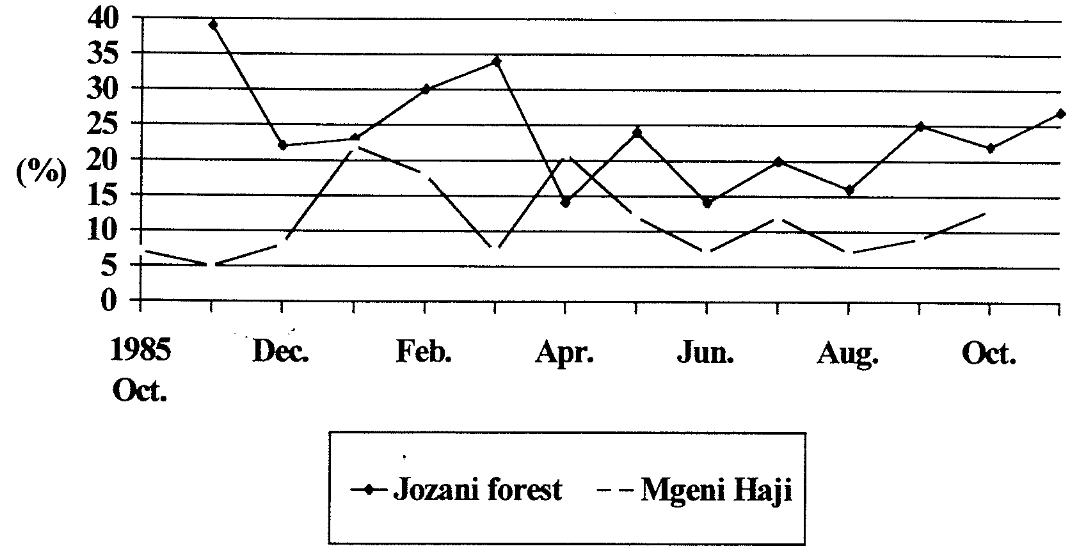

ANNEX 7

Table A 7.1. Crop production in Unguja, Zanzibar, pre-tsetse control - 1975/75 to 1985/86 (tons)

Year	Cloves	Cassava	Maize	Rice	Banana	Sorghum	Legumes	Sweet potato	Other roots and tubers
1975/76	10,159	299,268	4,639	22,137	107,182	3,427	530	63,111	4,246
1976/77	4,441	257,300	3,615	25,914	103,336	2,607	1,605	45,893	8,215
1977/78	769	209,422	3,252	24,755	99,950	1,069	1,174	32,459	20,075
1978/79	12,571	187,791	2,979	10,192	77,713	1,188	1,042	31,118	10,379
1979/80	6,482	269,159	3,431	22,464	67,873	1,557	2,356	17,847	16,569
1980/81	7,497	289,356	4,774	20,166	60,718	2,298	2,894	41,029	31,896
1981/82	6,150	200,768	3,596	18,580	36,013	1,990	2,036	34,892	23,478
1982/83	7,226	319,137	3,463	18,357	117,543	1,893	2,768	43,417	27,600
1983/84	4,698	231,796	2,164	14,645	54,623	1,875	1,619	34,405	22,094
1984/85	8,900	275,800	5,249	20,118	113,635	2,452	2,956	62,046	28,727
1985/86	136	221,748	2,011	15,635	60,064	870	1,715	19,671	20,170
Average 1975-86	6,275	251,049	3,561	19,360	81,695	1,929	1,881	38,717	19,404

Source: Department of Agriculture, Zanzibar

Table A 7.2. Land area under food crop production in Unguja, Zanzibar, pre-tsetse control - 1975/76 to 1985/86 (hectares)

Year	Cassava	Maize	Rice	Banana	Sorghum	Legumes	Sweet potato	Other roots and tubers
1975/76	65,839	12757	32467	23580	12562	2915	23143	934
1976/77	56606	9942	38007	22734	9560	8849	16829	2318
1977/78	46073	8942	36292	21989	3921	6500	11903	5294
1978/79	41314	8206	14948	17097	4356	2356	11410	2682
1979/80	59215	9433	32948	14932	5708	13278	6544	4282
1980/81	63658	13129	29576	13358	8425	16528	15044	7931
1981/82	44169	9889	26957	7923	7296	11737	12794	5796
1982/83	70217	9523	26924	25862	6941	15544	15919	6879
1983/84	50996	5952	21479	12019	6875	8947	12615	5692
1984/85	60676	14459	29507	25000	8992	16616	22750	7428
1985/86	48,785	5,529	22,992	13,214	3,192	9,275	7,213	5,201
Average 1975-1985	55,231	9,796	28,372	17,973	7,075	10,231	14,197	4,948

Table A 7.3. Crop production in Unguja, Zanzibar, during tsetse control and eradication, 1986/87 to 1996/98 (tons)

Year	Cloves	Cassava	Maize	Rice	Banana	Sorghum	Legumes	Sweet potato	Other roots and tubers
1986/87	12,035	156,799	1,768	8,112	54,240	460	1,833	30,651	14,610
1987/88	2,027	174,131	1,184	20,361	109,098	335	1,828	14,034	14,856
1988/89	12,703	113,840	1,268	20,570	22,800	438	937	14,232	7,357
1989/90	881	208,236	1,087	17,959	27,726	392	2,296	34,447	10,668
1990/91	1,705	179,352	1,392	12,186	46,672	563	2026	30,732	13340
1991/92	16,052	175,826	1,236	11,258	23,184	443	1,208	34,407	9,022
1992/93	1,757	149,122	645	15,580	18,476	445	991	34,600	11,216
1993/94	1,774	168,364	812	11,690	20,472	272	1,403	26,011	14,280
1994/95	5,344	141,128	1,093	11,719	16,626	139	941	12,853	6,201
1995/96	1,073	133,940	522	18,607	21,824	113	916	15,432	6,180
1996/97	11,368	26,948	229	26,045	5,796	68	10	6,941	1,776
Average 1985-97	6,065	147,971	1,021	15,826	33,356	333	1,308	23,121	9,955

Source: Department of Agriculture, Zanzibar

Table A 7.4. Land area under food crop production in Unguja, Zanzibar, during tsetse control and eradication, 1986/87 to 1996/97 (hectares)

Year	Cassava	Maize	Rice	Banana	Sorghum	Legumes	Sweet potato	Other roots and tubers
1986/87	34,496	4,861	16,245	11,933	1,687	10,279	11,239	3,612
1987/88	43,533	7,397	25,451	27,275	2,795	11,248	5,848	3985
1988/89	28,460	7,925	23,966	5,700	3,647	5,663	5,930	1,986
1989/90	52,059	6,793	22,448	6,932	3,264	16,289	14,353	2,810
1990/91	44,838	8,733	23,571	11,668	4,688	14,764	12,805	3,553
1991/92	43,957	7,722	16,705	5,796	3,689	8,446	14,336	2,365
1992/93	37,281	4,030	19,616	4,619	3,708	6,698	14,418	2,999
1993/94	42,091	5,074	27,044	5,118	2.263	9,833	10,838	3,857
1994/95	35,282	6,834	19,532	4,157	1,158	6,097	5,356	1,670
1995/96	33,485	3,263	21,363	5,456	938	6,251	6,430	1,661
1996/97	6,737	1,430	32,557	1,449	563	62	2,892	490
Average 1985-1998	36,565	5,823	22,590	8,191	2,376	8,693	9,495	2,635

Source: Department of Agriculture, Zanzibar

ANNEX 8

Table A 8.1. Land area (acres) cultivated for important food and cash crops after tsetse intervention in 1999 in Unguja, Zanzibar.

Type of crop	District					All districts
	Central	North-A	North-B	South	West	
Coconut	1.7	1.5	1.5	1.8	2.8	1.9
Cassava	1.3	1.6	1.6	1.1	1.4	1.4
Plantain	2.9	1.0	1.0	0.7	0.0	1.1
Sweet potatoes	0.9	1.5	1.5	0.7	1.0	1.1
Tania	1.2	1.2	1.2	0.9	1.0	1.1
Banana	1.2	0.8	0.8	1.2	1.0	1.0
Cloves	1.2	0.6	0.6	n.a	2.5	1.0
Rice	1.0	1.0	1.0	0.8	1.1	1.0
Maize	1.2	0.5	0.5	1.1	1.1	0.9
Yams	0.7	0.5	0.5	0.8	0.9	0.7
Vegetables	1.0	0.6	0.6	0.9	0.5	0.7

Table A 8.2. Quantities of food and cash crops harvested from cultivated areas before tsetse intervention in 1985/86 in Unguja, Zanzibar.

Type of crop	District					All Districts	
	Central	North-A	North-B	South	West	Average	% of farmers responding (n=1,138)
Cassava	632	829	849	578	1,240	826	43.5
Rice	554	374	432	750	581	538	24.7
Maize	451	300	358	363	674	429	9.0
Banana	66	24	30	40	58	44	31.0
Plantain	5	46	4	10	n.a	13	1.3
Sweet potatoes	519	1,017	1,019	559	1,380	899	26.1
Yams	626	50	388	387	648	420	5.5
Cloves	200	244	448	n.a	227	224	4.0
Coconut	2,825	428	778	418	1219	1,134	15.5
Tania	346	510	378	357	233	365	5.0
Vegetables	259	10	85	32	284	134	4.1

Source: Source: Averages and percentages are computed from Ministry of Agriculture statistics, Zanzibar.

Table A 8.3. Food and cash crop yields obtained before tsetse intervention in 1985/86 in Unguja, Zanzibar (Kg/acre)

Type of crop	District					All Districts	
	Central	North-A	North-B	South	West	Average	% of farmers responding (n=1,138)
Cassava	522	528	524	503	912	597	43.5
Rice	583	378	366	600	528	492	24.7
Maize	410	600	377	370	695	477	9.0
Banana*	55	34	32	34	61	44	31.0
Plantain*	29	73	13	26	0	44	1.3
Sweet potatoes	590	969	842	717	1,190	885	26.1
Yams	763	100	377	472	953	545	5.5
Cloves	200	387	193	0	86	170	4.0
Coconut	2,004	245	314	299	445	580	15.5
Tania	449	785	353	322	175	370	5.0
Vegetables	341	20	185	60	861	260	4.1

* Bunches

Source: Averages and percentages are computed from Ministry of Agriculture statistics, Zanzibar.

Table A 8.4. Land area (acres) cultivated for important food and cash crops after tsetse intervention in 1999 in Unguja, Zanzibar.

Type of crop	District					Total
	Central	North-A	North-B	South	West	
Coconut	1.7	1.5	1.5	1.8	2.8	1.9
Cassava	1.3	1.6	1.6	1.1	1.4	1.4
Plantain	2.9	1.0	1.0	0.7	0.0	1.1
Sweet potatoes	0.9	1.5	1.5	0.7	1.0	1.1
Tania	1.2	1.2	1.2	0.9	1.0	1.1
Banana	1.2	0.8	0.8	1.2	1.0	1.0
Cloves	1.2	0.6	0.6	n.a	2.5	1.0
Rice	1.0	1.0	1.0	0.8	1.1	1.0
Maize	1.2	0.5	0.5	1.1	1.1	0.9
Yams	0.7	0.5	0.5	0.8	0.9	0.7
Vegetables	1.0	0.6	0.6	0.9	- 0.5	0.7

Table A 8.5. Quantities of food and cash crops harvested from cultivated areas after tsetse intervention in 1999 in Unguja, Zanzibar.

Type of crop	District					All Districts	
	Central	North-A	North-B	South	West	Average	% of farmers responding (n=1,138)
Cassava	611	711	978	487	1164	790	85.6
Rice	502	430	426	295	560	443	40.5
Maize	416	150	418	315	661	392	17.6
Banana	74	29	37	47	74	52	63.6
Plantain	24	57	3	21	n.a	21	4.4
Sweet potatoes	508	1177	1281	215	1005	837	50.6
Yams	434	525	410	360	628	471	13.5
Cloves	320	100	528	n.a	297	249	5.2
Coconut	2379	492	782	547	1420	1,124	23.0
Tania	351	450	363	231	380	355	13.9
Vegetables	429	177	73	49	258	197	16.0

Table A 8.6. Food and cash crop yields obtained after tsetse intervention in 1999 in Unguja, Zanzibar (Kg/acre)

Type of crop	District					All Districts	
	Central	North-A	North-B	South	West	Average	% of farmers responding (n=1,138)
Cassava	477	434	596	443	826	559	85.6
Rice	512	413	410	369	491	443	40.5
Maize	353	300	836	297	630	457	17.6
Banana*	63	35	45	41	75	53	63.6
Plantain*	8	57	3	32	0	19	4.4
Sweet potatoes	577	801	871	299	1,005	756	50.6
Yams	611	1,050	820	429	714	687	13.5
Cloves	267	159	838	0	120	252	5.2
Coconut	1,442	328	521	297	514	608	23.0
Tania	290	369	298	269	396	324	13.9
Vegetables	452	305	126	55	561	285	16.0

* Bunches

Source: 1999 Survey results.

ANNEX 9

Terms of Reference

The main duties of the mission team were to:

(i) Obtain appropriate information on the livestock situation and related agricultural and other relevant economic parameters on Unguja Island of Zanzibar before initiation of tsetse/trypanosomosis intervention operations in 1985/86 and 1999 and

(ii) establish parameters and sampling procedures to enable future impact assessments of tsetse eradication on livestock and agriculture development as well as well-being of people on Unguja.

These objectives were to be accomplished within two missions to Unguja, Zanzibar.

First Mission:

During the first mission the team was expected to :

- Prepare a questionnaire to retrospectively collect relevant information on the livestock and agriculture situation in 1985/86 before initiation of tsetse intervention operations, taking into account some available data and reports.
- Prepare a questionnaire for assessing the current status and future potential of livestock and agriculture development in Zanzibar, taking into account current livestock and agricultural practices, as well as, the potential for intensified systems, with particular reference to the potential for genetically upgraded cattle breeds. Incorporate into the data compilation procedure measures for data quality assurance.
- Prepare a time frame for the data compilation and the economic impact exercise and advise on additional action to be taken by the Government, IAEA and other collaborators.
- Collaborate closely with the local authorities to identify and provide all necessary instructions to enumerators who will interview farmers, cattle owners and other people in Zanzibar in connection with the data collection exercise.
- Define an appropriate data evaluation procedure and a convenient mode of data presentation and of evaluation of relevant current and anticipated developments.

Second Mission

During the second mission the team was expected to:

- Screen the completed questionnaires and, when necessary, interview selected farmers, livestock owners and other people, in order to obtain any complementary information.
- Summarise the status of livestock and agricultural systems in 1985/86. Highlight indicative key parameters for livestock productivity and performance and stress the respective implications for agricultural systems and for marketing of agricultural products.
- Summarise the status of livestock and agriculture development in 1999. Highlight indicative key parameters that show changes in livestock numbers, productivity and performance. Quantify and

qualify the changes and assess whether they are related to or due to the tsetse fly intervention and eradication operations that were implemented in late 1997.

- Prepare some predictive scenarios for the livestock and agricultural development through 2005, 2010 and 2015, taking into consideration the livestock and agricultural development over the past 15 years (1985 - 1999) and the assessed potential of livestock and agricultural development of Zanzibar and respective development plans of the Zanzibar authorities.

- Advise on issues that require attention by the Zanzibar authorities and other partners involved in livestock and agriculture development in Zanzibar.
